# Organocatalytic atroposelective synthesis of axially chiral styrenes

**DOI:** 10.1038/ncomms15238

**Published:** 2017-05-03

**Authors:** Sheng-Cai Zheng, San Wu, Qinghai Zhou, Lung Wa Chung, Liu Ye, Bin Tan

**Affiliations:** 1 Department of Chemistry, South University of Science and Technology of China, Shenzhen 518055, China

## Abstract

Axially chiral compounds are widespread in biologically active compounds and are useful chiral ligands or organocatalysts in asymmetric catalysis. It is well-known that styrenes are one of the most abundant and principal feedstocks and thus represent excellent prospective building blocks for chemical synthesis. Driven by the development of atroposelective synthesis of axially chiral styrene derivatives, we discovered herein the asymmetric organocatalytic approach via direct Michael addition reaction of substituted diones/ketone esters/malononitrile to alkynals. The axially chiral styrene compounds were produced with good chemical yields, enantioselectivities and almost complete *E*/*Z*-selectivities through a secondary amine-catalysed iminium activation strategy under mild conditions. Such structural motifs are important precursors for further transformations into biologically active compounds and synthetic useful intermediates and may have potential applications in asymmetric synthesis as olefin ligands or organocatalysts.

## Abstract (web summary)

Many methods exist for the atroposelective synthesis of axially chiral biaryls, but axially chiral styrenes are much less explored. Here the authors report an organocatalytic procedure for the synthesis of configurationally stable, axially chiral styrenes with high enantio- and diastereoselectivities.

Axially chiral compounds in which the chirality originates from highly sterically hindered rotation along a chiral axis rather than a stereogenic centre with four different substituents have received much attention from chemists because of their widespread appearance in biologically active compounds and useful chiral ligands in asymmetric catalysis. Among the well-known axially chiral structures, most of the chiral axis is between two aromatic moieties as named biaryl atropisomers. Owing to the importance of this structural motif, the catalytic atroposelective construction of axially chiral biaryls has been intensively investigated^1^,^2^,^3^,^4^,^5^,^6^ and could be accessed by enantioselective oxidative/cross coupling of two aryl counterparts,^7^,^8^,^9^,^10^,^11^,^12^,^13^ asymmetric construction of an aromatic ring^14^,^15^,^16^,^17^,^18^,^19^,^20^ and kinetic resolution/desymmetrization of biaryl compounds (Fig. 1a, left)^21^,^22^,^23^,^24^,^25^,^26^,^27^,^28^,^29^,^30^,^31^,^32^,^33^,^34^,^35^,^36^. However, in sharp contrast, the axially chiral styrenes bearing a chiral axis between a simple alkene and an aromatic ring have been rarely studied with respect to asymmetric synthesis and applications^37^,^38^. Although this type of atropisomer was firstly proposed to demonstrate a new concept of the memory of chirality by Kawabata *et al*.^39^ in 1991, the consequent investigations on synthesis or application of axially chiral styrenes remain underexplored^40^,^41^,^42^ (Fig. 1a, right). The main reasons are presumably attributed to the relatively low-rotation energy to racemization and the difficulty to control the enantioselectivity.

Inspired by elegant reports concerning the synthesis of axially chiral styrene-type derivatives^43^,^44^, Gu and co-workers^45^ developed an efficient enantioselective construction of axially chiral vinyl arenes from aryl bromide and hydrazones by using palladium catalysis, providing a pioneering example for the atroposelective catalytic synthesis of axially chiral styrene-type derivatives. It is well-known that simple styrenes are one of the most abundant and principal feedstock, and thus represent excellent prospective building blocks for chemical synthesis. Most interestingly, chiral olefins have been utilized to generate metal–olefin complexes for enantioselective transformations^46^,^47^,^48^. Therefore, the development of enantioselective synthetic approach to axially chiral styrenes becomes very attractive and highly desirable. Iminium activation^49^,^50^ has been successfully utilized to control the enantioselectivity at the *β*-position on the *α*,*β*-unsaturated aldehyde (enal). Alkynals are known to be a challenging class of electrophiles in asymmetric catalysis because they would adopt different structures and geometry^51^. As such, in contrast to these widely reported iminium activated enal systems, the organocatalytic enantioselective transformation involving alkynal remains underexplored^52^,^53^,^54^,^55^,^56^. In this context, Wang and co-workers reported the elegant work^53^ involving a domino iminium-allenamine process to control the stereoselectivity far from the *β*-position on the alkynal. To further extend the utility of alkynal in asymmetric synthesis and particularly perform a direct enantioselective reaction of nucleophilic addition to alkynal, we envision that the chiral allenamine intermediate *in situ* generated from the initial Michael addition of nucleophile to an iminium ion would then transfer the chiral information to control the axial chirality of the styrene derivatives (Fig. 1b).

In this scenario, several challenges would be encountered: (1) the atroposelective construction of axially chiral styrene compounds has rarely been investigated in asymmetric catalysis, (2) the selection of appropriate nucleophile to allow a compromise between reactivity and selectivity in the organocatalytic nucleophilic addition and the steric hindrance required to possess enough rotation energy to maintain the integrity of the chiral axis, (3) the choice of a suitable chiral organocatalyst to efficiently control *E*/*Z* selectivity since in only one isomer may exist axial chirality. As part of our ongoing interest in asymmetric organocatalysis on construction of axially chiral compounds^15^,^27^,^57^,^58^, we describe herein the first organocatalytic atroposelective synthesis of axially chiral styrene compounds via direct Michael reaction of substituted diones/ketone esters/malononitrile to alkynals, providing a powerful and straightforward synthetic route toward substituted styrene derivatives. Such structural motifs are important chiral components for further transformations into biologically active natural products and pharmaceutical compounds and may have potential application in asymmetric catalysis as olefin ligands or organocatalysts.

## Results

### Discovery of configurationally stable axially chiral styrenes

**Figure 2:**
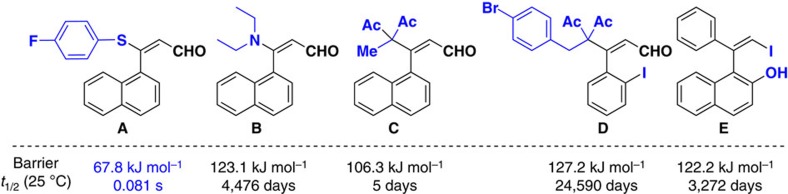
Computed rotation barriers of several styrenes and their corresponding half-life. The above computed rotation barriers along the axial C-CAr bond and their corresponding *t*_1/2_ (25 °C) were estimated by SMD M06-D3/6-31+G*//M06-D3/6-31G* method. (See the computational details and Supplementary Figs 1–4).

Motivated by Kawabata’s pioneering discovery, we synthesized the compound **A** through Michael addition reaction and imagined that in such compound may exhibit axial chirality due to the restricted rotation between double bond and the directly attached naphthyl group (Fig. 2). Disappointedly, compound **A** displays no axial chirality based on the chiral stationary high-performance liquid chromatography (HPLC) analysis and computed relatively low-rotation barrier (67.8 kJ mol^−1^), corresponding to a first-order half-life (*t*_1/2_) of 0.081 s. To further increase the rotation barrier for an axially chiral styrene compound, we synthesized three styrene-type compounds **B**, **C**, **D** and **E** with more bulky nucleophiles. We are pleased to find that these compounds show apparently axial chirality and much larger rotation barriers (Fig. 2). Notably, the compound **D** possess high rotational energy barrier around 127.2 kJ mol^−1^, clearly indicating that the substituted styrene-type compounds might be stable for asymmetric synthesis and other transformations. On the base of racemization experiments and kinetics of racemisation of an enantiomer, the rotational barrier of compound **D** was 117.9 kJ mol^−1^ under the solvent of chloroform at 61 °C (for details, see Supplementary Note 1).

### Optimization of reaction conditions

After confirmation of the axially chiral styrenes (Fig. 2, compounds **B**, **C**, **D**, **E**), we turned our attention to develop an atroposelective synthesis of chiral styrenes. Our initial investigations focused on evaluating the reaction between 3-(4-bromobenzyl)pentane-2,4-dione **1a** and 2-iodophenylpropiol-aldehyde **2a** in chloroform (CHCl_3_) at room temperature in the presence of the secondary amine catalyst^59^,^60^,^61^,^62^, **C1**. Despite its high steric hindrance, the desired axially chiral styrene-type **3a** was isolated with 74% yield and complete *Z*/*E-*selectivity control (>20:1), accompanying with moderate enantioselectivity (65% ee) (Table 1, entry 1). This proof-of-principle result clearly suggests that the axial chirality of styrene-type compound could be controlled very well by using a chiral secondary amine as organocatalyst. Having thus proven the efficiency of the secondary amine catalyst in our system, we next moved to investigate the substituent and protecting group effects on the catalyst (Table 1, entries 2–7). As shown in Table 1, the electron property on the aromatic ring and the steric size of the silyl ether group have very strong influences on the reactivity and enantioselectivity. Catalyst **C5** displayed the best results in terms of the enantioselectivity (90% ee) (Table 1, entry 5). Upon optimizing the reaction conditions through variations of the solvent, temperature, catalyst loading and additive (Table 1, entries 8–13 and Supplementary Tables 1–4), we identified the following protocol as optimal: when **1a** (0.05 mmol) was treated with **2a** (0.055 mmol) in the presence of catalyst **C5** (5 mol%) in DCM at 0 °C for 24 h under the addition of LiOAc, the axially chiral styrene-type **3a** was obtained in 96% isolated yield with 94% ee (Table 1, entry 13).

### Substrate scope

After an acceptable optimal reaction condition established, we turn our attention to the substrate scope investigation. Firstly, we have investigated the substituted alkynals and the results were summarized in Table 2. Different substituents (I, CF_3_, SO_2_Ph, ^*i*^Pr, ^*t*^Bu) on the phenyl ring of the alkynal substrates were tolerated to produce the desired axially chiral products with moderate to good results (**3a**–**3c**, **3j**–**3l**). Different substituents on the naphthyl rings, such as electron-donating groups (–OMe), electron-neutral and electron-withdrawing groups (–Br), were found to be suitable to give the corresponding products **3d–3f** in 75–99% yields with 87–92% ee. Moreover, the phenyl group at the substrate **2a** could be replaced with 9-phenanthryl or 1-pyrenyl substituent without affecting the chemical yields, albeit with a little bit lower enantioselectivities (**3g** and **3h**). It should be pointed out that the aldehyde with two different *ortho* substituents on the aryl ring is not an appropriate substrate for this transformation under the current standard condition (Table 2, product **3i**).

To further explore the scope of this transformation, we then evaluated the use of various diones as nucleophiles (Tables 3, **1c–1n**). Most reactions reached completion within 24 h and gave axially chiral products in moderate to good yields (49–99%) with excellent enantioselectivities (90–95% ee). For the use of substituted benzyl pentanedione substrates, the position and electronic properties of substituents (Ph, 4-ClPh, 3-BrPh, 4-MePh, 4-OMePh) appeared to have a very limited effect on chemical yields and stereoselectivities as expected (Table 3, products **3m–3r**). Encouraged by these results, we expanded the generality of the reaction by using 3-allylpentane-2,4-dione, 3-chloropentane-2,4-dione, 2-methylcyclohexane-1,3-dione as reactants (Table 3, products **3s–3u**). The desired products can be obtained with good enantioselectivities, demonstrating the broad generality of this approach for the synthesis of axially chiral styrene derivatives. To our delight, the reaction performed very well with ketone ester (**1l**, **1m**) albeit with unsatisfactory dr value. Furthermore, we were pleased to find that the reactions proceeded smoothly with excellent yields and good enantioselectivities using 2-benzylmalononitrile (**1n**) as nucleophile (Table 2, **3j–3l**; Table 3, **3x**).

### Preparative scale synthesis of **3n** and **3y**

**Figure 3:**
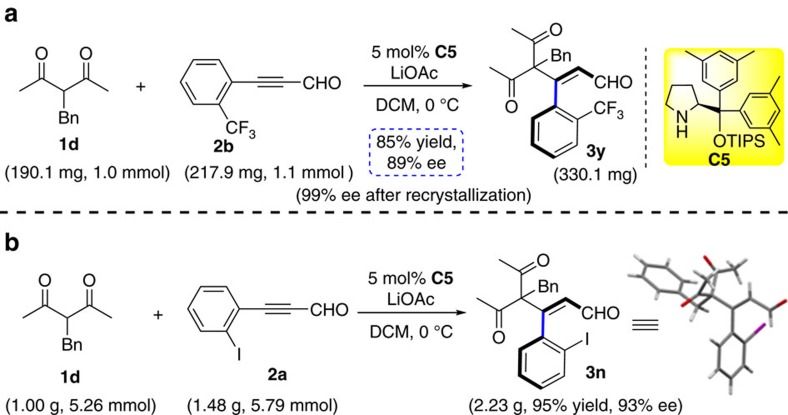
Preparative synthesis of 3y and 3n. (**a**) Preparative synthesis of **3y** under the corresponding standard conditions. (**b**) Gram-scale preparation of **3n**.

To demonstrate the utility of this transformation, preparative scale synthesis of products **3n** and **3y** were carried out. As displayed in Fig. 3, there was almost no change in chemical yield and stereoselectivity. An indication for the configurational stability of the product was obtained by heating a solution of **3a** in dichloroethane (DCE) at 40 °C for 24 h. HPLC analysis showed that the enantioselectivity was unaffected. Thus, the obtained axially chiral styrene-type compounds may have potential application in asymmetric synthesis. The absolute configuration of **3n** was determined to be *S* by X-ray diffraction analysis (CCDC 1507490, see Supplementary Fig. 5) and those of other products were assigned by analogy.

### Synthetic application

**Figure 4:**
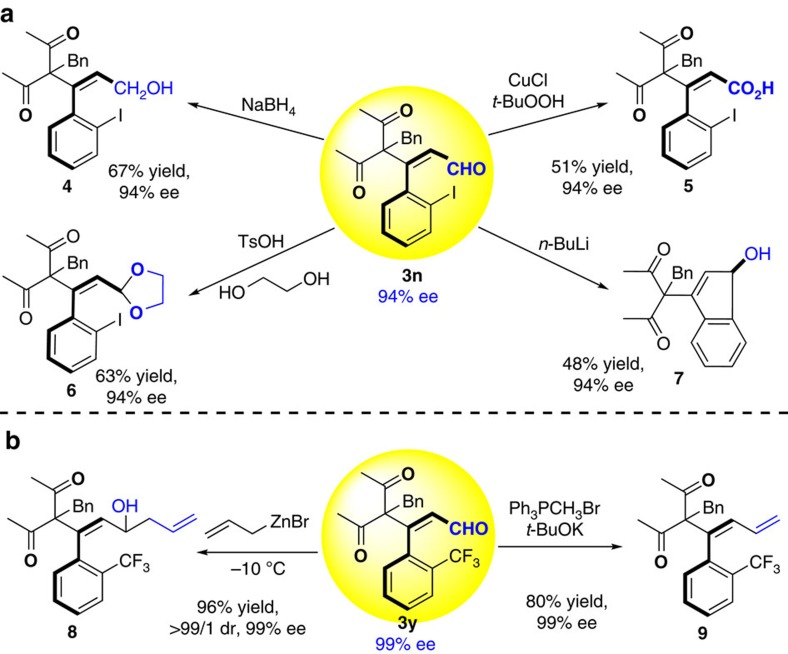
Versatile chemical transformations of axially chiral 3n and 3y. (**a**) Synthetic transforamtion of enantioenriched **3n**. (**b**) Synthesis of diene via simple trasformation of **3y**.

With the atroposelective synthesis of axially chiral styrene-type derivatives established, we turned to the demonstration of further synthetic utility through derivatization of the obtained products (Fig. 4). Gratifying, the obtained enal **3n** could be selectively reduced to produce alcohol **4** or oxidized to carboxylic acid **5** under mild conditions^63^,^64^ without any erosion of enantioselectivity. The aldehyde can be protected with glycol and the resulting acetal **6** maintains the axial chirality with same enantioselectivity^65^. Furthermore, by treatment of **3y** with Wittig reagent, the desired 1,3-diene **9** could be easily obtained with the same enantiomeric excess. Thus, the aldehyde group is not necessary for this type of axially chiral compound. It should be worth highlighting that the axial chirality of the styrene can be easily transferred into carbon stereogenic compound **7** under the treatment with *n*-BuLi and the resulting product remains the same enantiomeric excess (Fig. 4a)^66^. Finally, the expected 1,5-diene **8** with complete diastereocontrol (dr>99:1) was easily achieved by allylation of aldehyde with allylzinc bromide reagent^67^,^68^, probably providing a new type of axially chiral diene ligand for asymmetric catalysis.

## Discussion

We have developed the first organocatalytic approach to atroposelective synthesis of axially chiral styrene derivatives via direct nucleophilic addition of substituted diones to alkynals. The axially chiral styrene compounds were produced with good chemical yields and enantioselectivities through a secondary amine-catalyzed iminium activation strategy under mild reaction conditions. Moreover, several transformations were investigated to demonstrate synthetic utilities to more functional axially chiral styrene-type compounds. Considering the importance of the axially chiral styrenes, we further anticipate that this promising strategy will motivate the design of other related processes. The application of this strategy to a broader substrate scope and mechanistic investigations are currently underway in our group.

## Methods

### General information

Reagents were purchased at the highest commercial quality and used without further purification, unless otherwise stated. Analytical thin layer chromatography (TLC) was performed on precoated silica gel 60 F254 plates. Flash column chromatography was performed using Tsingdao silica gel (60, particle size 0.040–0.063 mm). Visualization on TLC was achieved by use of ultraviolet light (254 nm). NMR spectra were recorded on a Bruker DPX 400 spectrometer at 400 MHz for ^1^H NMR, 100 MHz for ^13^C NMR and 376 MHz for ^19^F NMR in CDCl_3_ or acetone-*d*_6_ with tetramethylsilane (TMS) as internal standard. Chemical shifts are reported in p.p.m. and coupling constants are given in Hz. Data for ^1^H NMR are recorded as follows: chemical shift (p.p.m.), multiplicity (s, singlet; d, doublet; t, triplet; q, quartet; m, multiplet), coupling constant (Hz), integration. Data for ^13^C NMR are reported in terms of chemical shift (*δ*, p.p.m.). High-resolution mass spectra were recorded on a LC-TOF spectrometer (Micromass). Enantiomeric excess was determined on Agilent HPLC using DAICEL CHIRAL column. Racemic compounds were obtained by using diisopropylamine as catalyst. For NMR analysis of the compounds in this article, see Supplementary Figs 6–97. For characterization of structurally-novel chemical compounds, see Supplementary Notes 2 and 3. For the procedure of versatile transformations, see Supplementary Note 4.

### Computational details

For each of the five compounds **A**–**E**, a conformational search was first performed using Macromodel (via mixed MCMM/Low-mode sampling method and OPLS_2005 force field). Next, the lowest-energy conformation and the conformations within 3 kcal mol^−1^ range in energy were used to fully optimize minima and the rotation transition states by density functional theory (DFT) method (Supplementary Figs 1–4). Afterward, harmonic vibrational frequency calculations (at 298.15 K) were performed to ensure one imaginary frequency for the optimized transition states and no imaginary frequency for the optimized minima. All the optimization and frequency calculations were performed at M06-D3/BS1 level (BS1: 6–31G* basis set for C, H, N, O, F, S Br atoms was used, and Lanl2dz basis set with its ECP were used for I atoms) in gas phase. Single-point energy calculations were performed at M06-D3/BS2 level (BS1I: 6–31+G** basis set for C, H, N, O, F, S Br atoms was used, and Lanl2dz basis set with its ECP were used for I atom, with ultrafine integral grid) by using SMD solvation model (solvent: dichloromethane). For each compound, the lowest-free energy conformation of minimum or transition state was used to discuss the rotation pathway. All the DFT calculations were carried out by Gaussian 09 software package^69^. The *t*_1/2_ values were computed based on transition state theory and first-order kinetic (*k*=(*k*_B_*T*/*h*)*EXP*(−*Δ*G*/*RT*); *t*_1/2_*=*ln(2)/*k*). The 3D model of each optimized structure was generated with CYL view^70^.

### General procedure for atroposelective synthesis of axially chiral styrenes 3

(*S*)-2-bis(3,5-dimethylphenyl)((tri-isopropylsilyloxy)methyl)pyrrolidine **C5** (5 mol%) and LiOAc (0.05 mmol) were added to a solution of alkynal **2** (0.11 mmol, 1.1 equiv) in methylene chloride (1 ml). After the mixture was cooled to 0 °C, **1** (0.10 mmol, 1.0 equiv) was added and maintained at 0 °C for 24 h. After the reaction was complete (monitored by TLC), the mixture was concentrated under reduced pressure and purified by flash chromatography on silica gel and eluted with PE/EA (8/1 to 3/1) to afford the corresponding axially chiral styrene products **3**.

### Data availability

The X-ray crystallographic coordinates for structures reported in this article have been deposited at the Cambridge Crystallographic Data Centre (CCDC), under deposition number of CCDC 1507490. These data can be obtained free of charge from The Cambridge Crystallographic Data Centre via http://www.ccdc.cam.ac.uk/data_request/cif. All other data is available from the authors upon reasonable request.

## Additional information

**How to cite this article:** Zheng, S.-C. *et al*. Organocatalytic atroposelective synthesis of axially chiral styrenes. *Nat. Commun.* **8,** 15238 doi: 10.1038/ncomms15238 (2017).

**Publisher’s note**: Springer Nature remains neutral with regard to jurisdictional claims in published maps and institutional affiliations.

## Supplementary Material

Supplementary Information Supplementary Figures and Supplementary Tables

Peer Review File

## Figures and Tables

**Figure 1:**
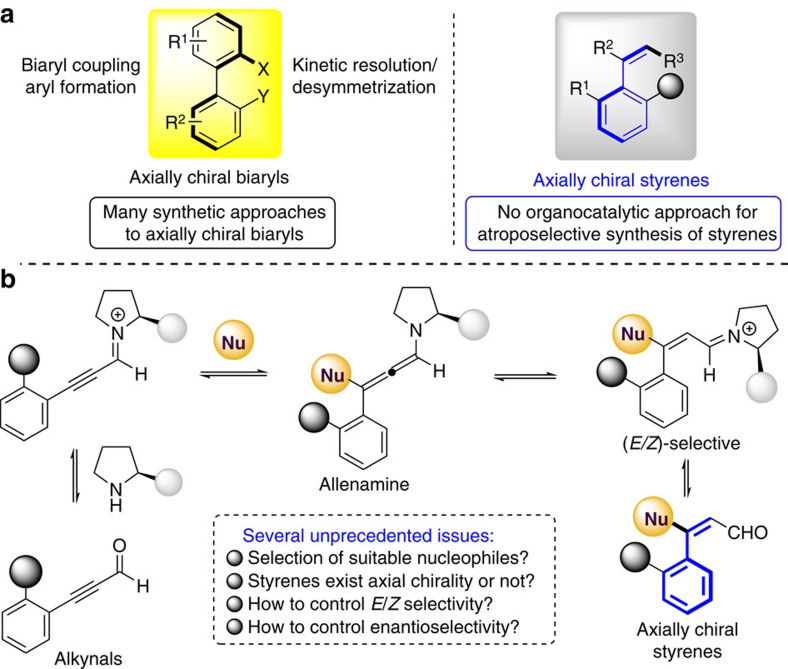
Background introduction of axially chiral styrenes and our strategy. (**a**) The existing approaches for atroposelective synthesis of axially chiral biaryls and styrenes. (**b**) Our strategy for atroposelective synthesis of axially chiral styrenes.

**Table 1:**
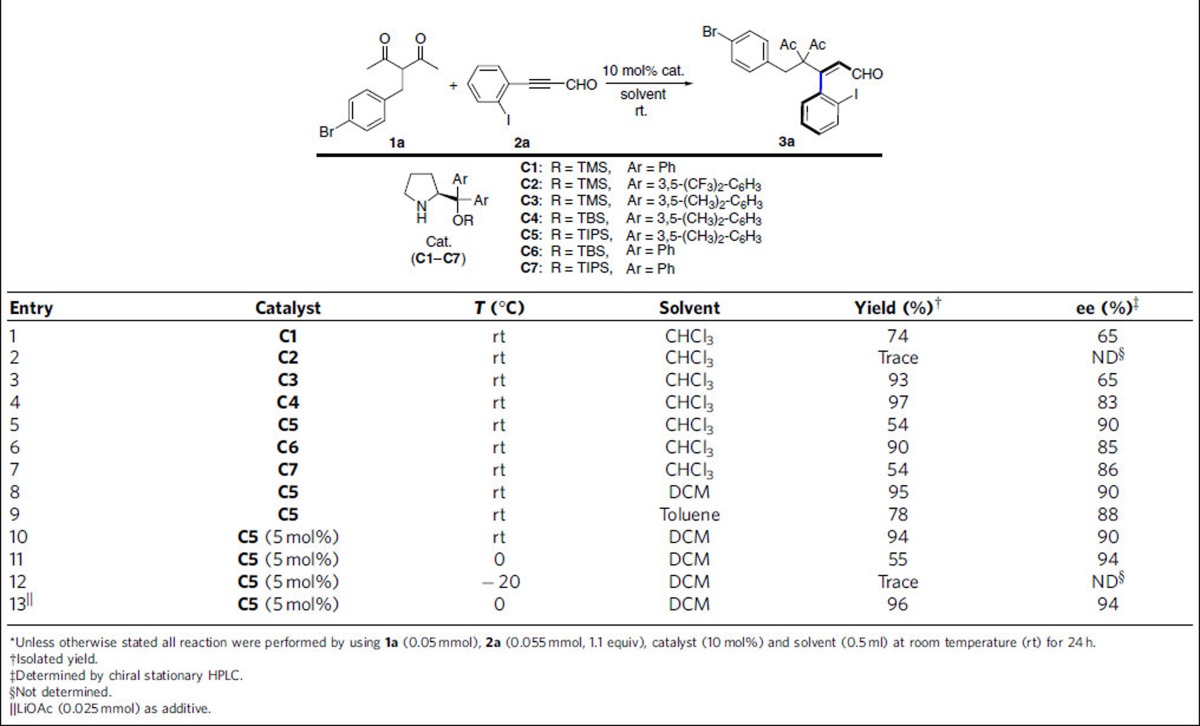
Optimization of the reaction conditions^*^.

**Table 2:**
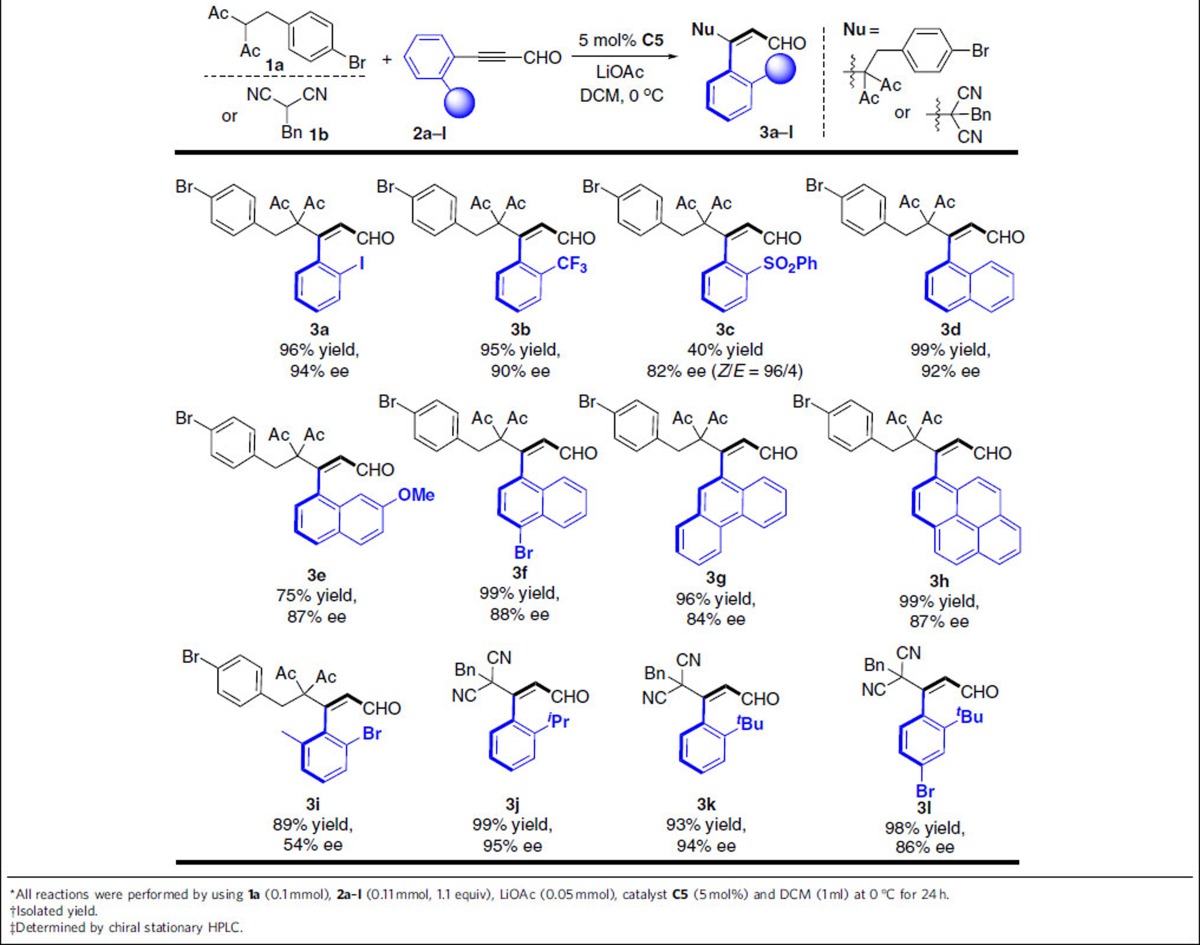
The substrate scope with respect to alkynals^*^^†^^‡^.

**Table 3:**
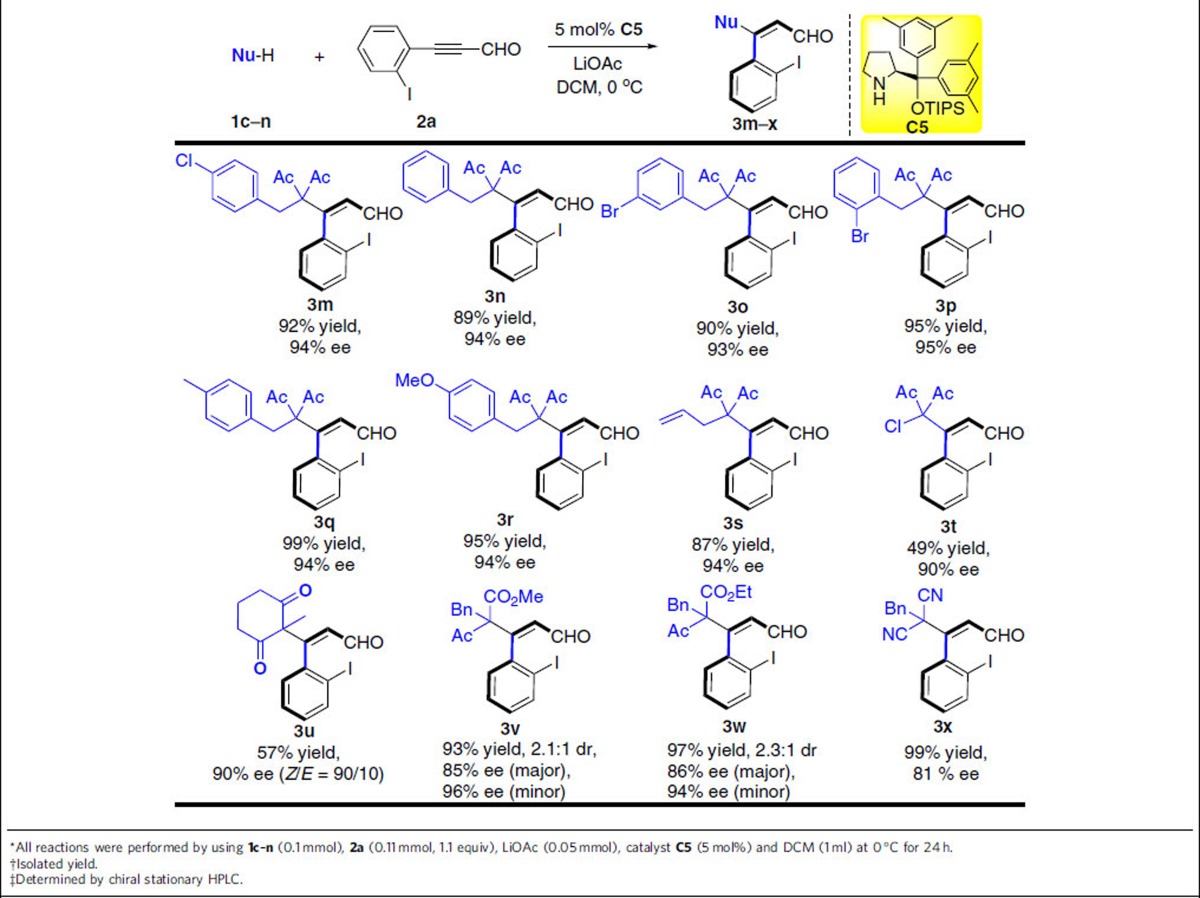
The substrate scope with respect to different nucleophiles^*^^†^^‡^.
