# Erratum: Organocatalytic atroposelective synthesis of axially chiral styrenes

**DOI:** 10.1038/ncomms16119

**Published:** 2017-06-27

**Authors:** Sheng-Cai Zheng, San Wu, Qinghai Zhou, Lung Wa Chung, Liu Ye, Bin Tan

Nature Communications 8: Article number:15238; DOI: 10.1038/ncomms15238 (2017); Published: 05 03 2017; Updated: 06 27 2017

An incorrect version of the Supplementary Information was inadvertently published with this Article. The HTML has now been updated to include the correct version of the Supplementary Information.

## Supplementary Material

Supplementary Information

