# Supplementary material for: Organocatalytic atroposelective synthesis of axially chiral styrenes
Source: Nat Commun. 2017 May 3;8:15238. doi: 10.1038/ncomms15238 (PMC5418600; doi:10.1038/ncomms15238)
Supplement: Supplementary Information — Supplementary Figures and Supplementary Tables [file ncomms15238-s1.pdf]

## Supplementary Figure 1

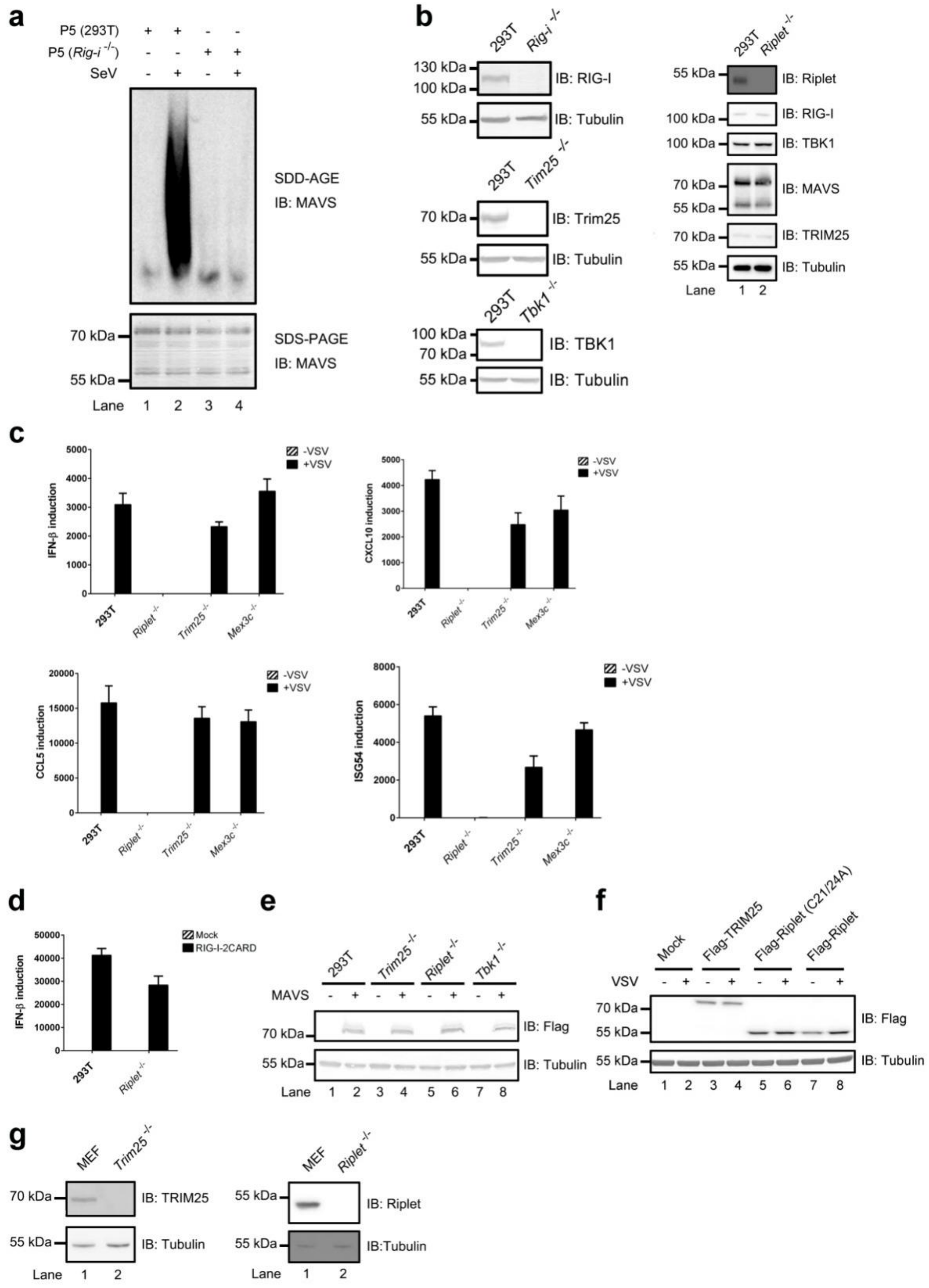


**Supplementary Figure 1 | Reconstitution of a cell-free assay for RIG-I and MAVS activation in antiviral signaling, Related to Fig. 1.**

(**a**) HEK293T cells (wild type or *Rig-i* $^{-/-}$) were homogenized and subjected to

subcellular fractionation to get P5 fractions, which were subjected to SDD-AGE and SDS-PAGE followed by immunoblotting analysis with anti-MAVS antibody.

(**b**) Immunoblotting to show the expression levels of RIG-I, TRIM25, TBK1, Riplet and MAVS in various knockout cell lines used in Fig. 1d.

(**c**) HEK293T cell lines (WT and knockout lines) were infected with VSV for twelve hours. The cells were then harvested and subjected to qPCR analysis for the expression of *IFN*, *CXCL10*, *CCL5* and *ISG54*. All data are presented as the mean values based on three independent experiments, and error bars indicate s.d.

(**d**) pcDNA3-flag-RIG-I-2CARD were transfected into HEK293T cells (WT and Riplet$^{-/-}$). Thirty six hours after transfection, The cells were harvested for qPCR analysis for IFN expression

(**e**) Immunoblotting to show the expression levels of Flag-MAVS as indicated in Fig. 1e.

(**f**) Immunoblotting to show the expression levels of TRIM25, Riplet (WT and C21/24A) as indicated in Fig. 1f.

(**g**) Immunoblotting to show the expression levels of TRIM25 and Riplet in different MEF cell lines.

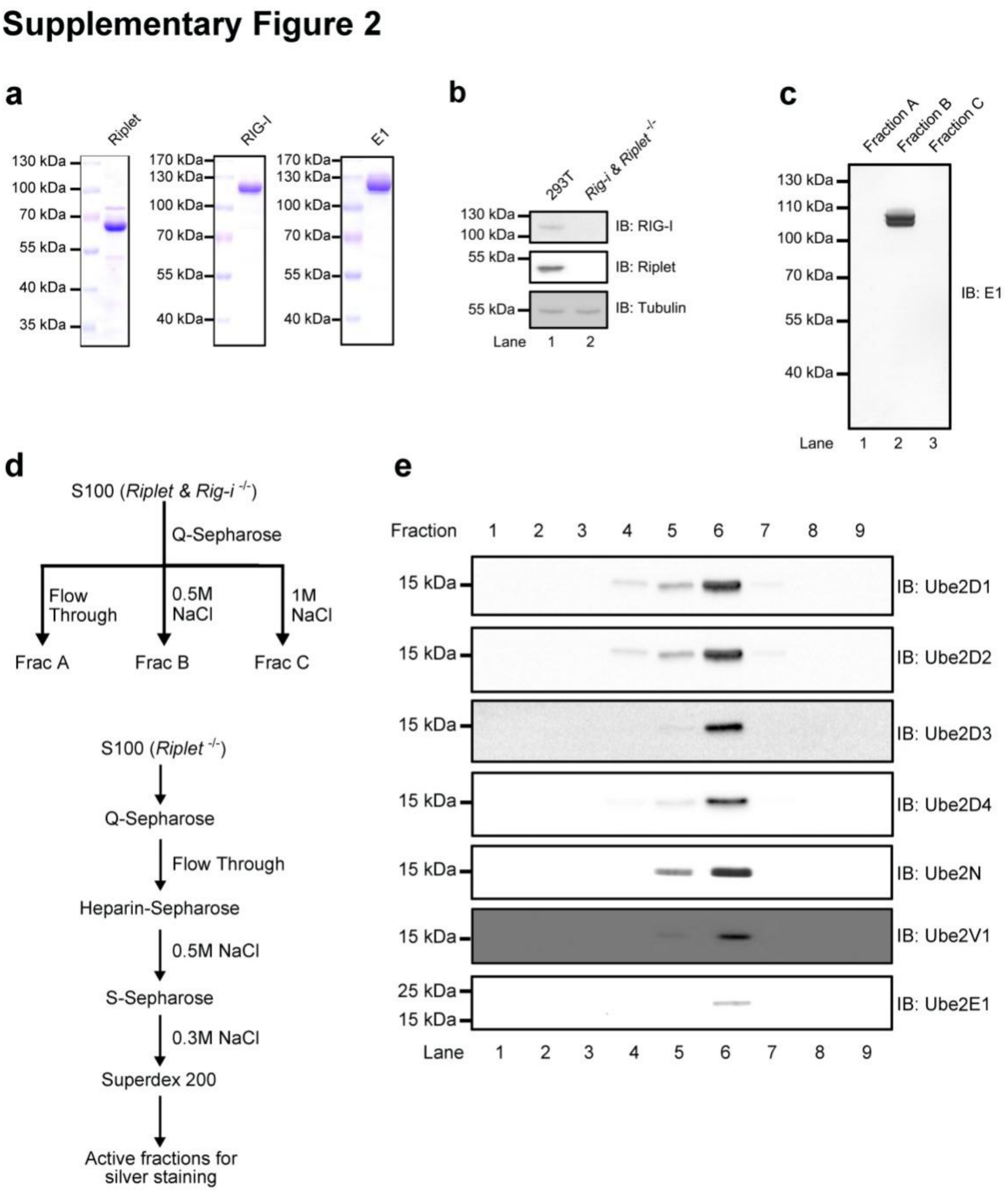


**Supplementary Figure 2 | Purification of multiple E2s required for RIG-I and MAVS activation in the cell-free assay, Related to Fig. 2.**

(**a**) His-tagged Riplet, RIG-I and E1 recombinant proteins were expressed in E.coli or insect cells and purified for cell-free assay. Proteins were separated

by SDS-PAGE and visualized by commassie blue staining.

(**b**) Immunoblotting to show the expression levels of RIG-I and Riplet in HEK293T cells (WT and *RIG-I*$^{-/-}$ & *Riplet*$^{-/-}$ double knockout cell line).

(**c**) Fractions (A, B and C) from HiTRAP mono-Q column were immunoblotted with anti-E1 antibody.

(**d**) Scheme of fractionation strategy to purify essential components shown in Fig. 2d.

(**e**) Fractions used in Fig. 2d were separated by SDS-PAGE, which was followed by Immunoblotting with antibodies as indicated.

## Supplementary Figure 3

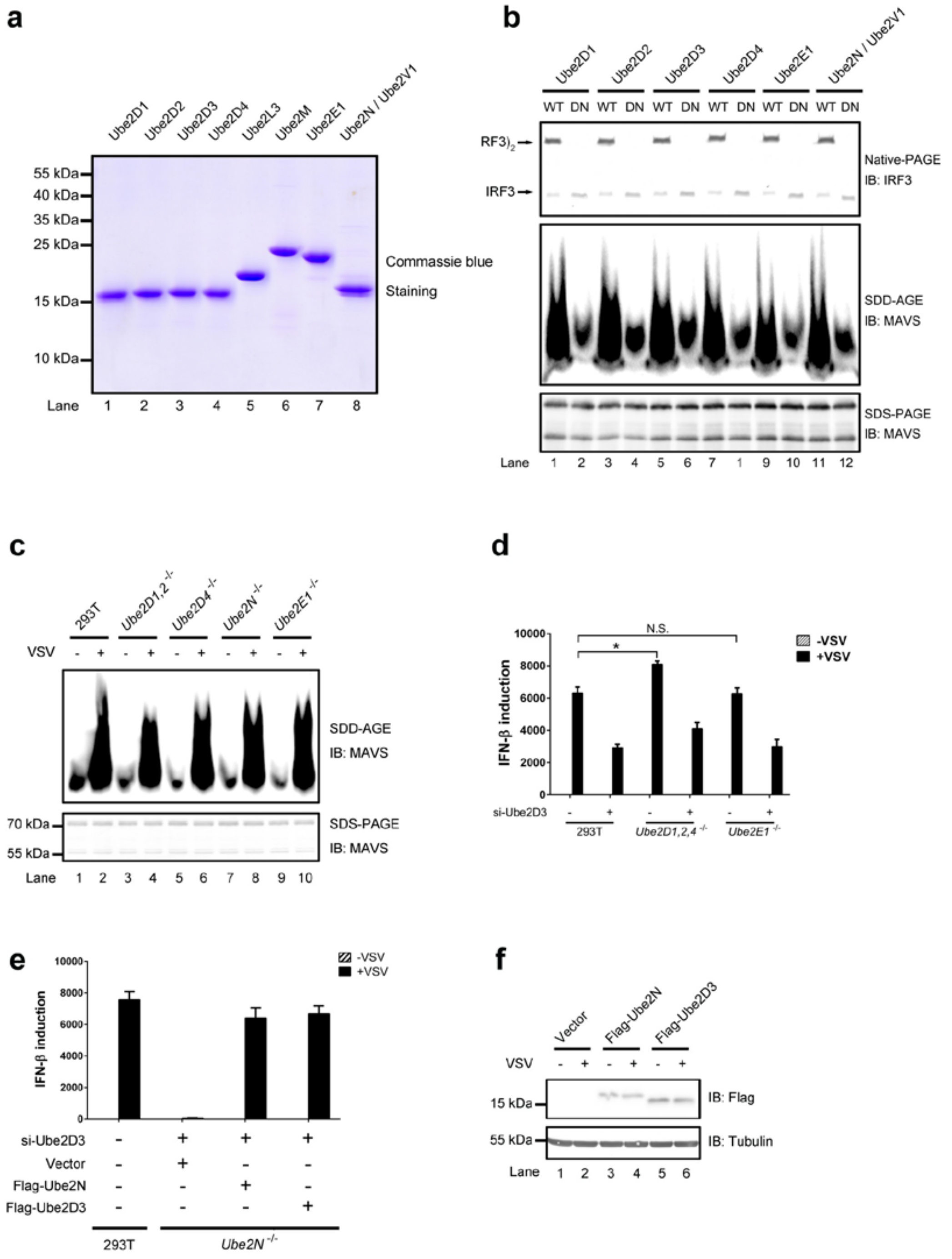


**Supplementary Figure 3 | Ub2D3 and Ube2N are required for RIG-I and MAVS activation in HEK293T cells, Related to Fig. 3.**

(**a**) Recombinant proteins used in the cell-free assay were separated by SDS-PAGE and stained by commassie blue.

(**b**) Wild type (WT) or mutant form (DN) of various E2s (Ube2D1/2/3/4, Ube2E1 and Ube2N) were used in the cell-free assay as described in Fig. 3a.

(**c**) HEK293T cells (wild type and various knockout lines) were infected with VSV. Twelve hours post virus infection, the cells were harvested for SDD-AGE to examine MAVS aggregation.

(**d-e**) The cells were treated as described in Fig. 3e and harvested for measuring IFN induction by qPCR. All data are presented as the mean values based on three independent experiments, and error bars indicate s.d.

(**f**) Immunoblotting to show the expression levels of Flag-Ube2N and Flag-Ube2D3 as indicated in Fig. 3f.

## Supplementary Figure 4

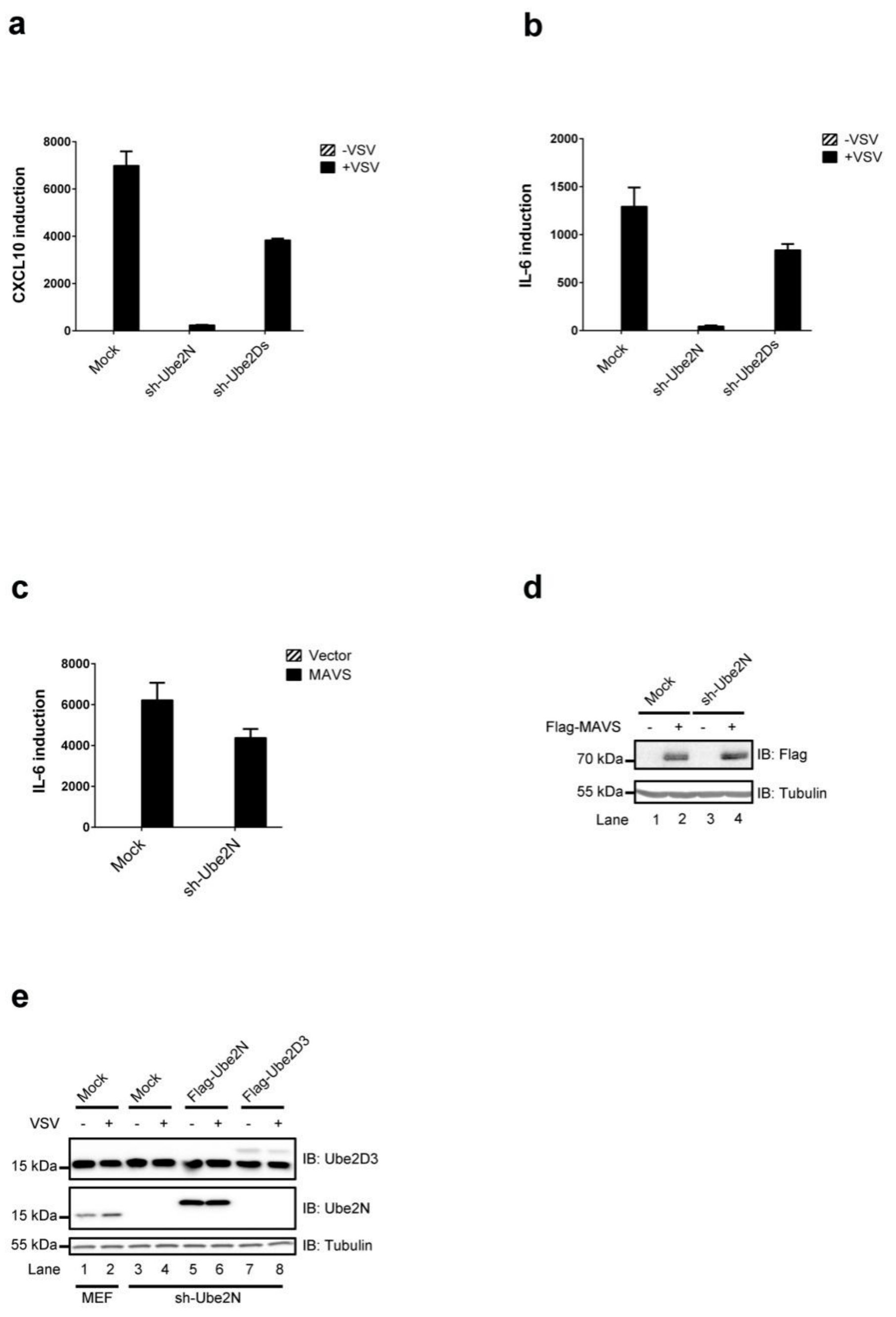


**Supplementary Figure 4 | Ube2N is required for RIG-I and MAVS activation in MEF cells, Related to Fig. 4.**

(**a-b**) MEF cells were treated as described in Fig. 4a and the production of CXCL10 (a) and IL6 (b) was measured by qPCR. All data are presented as the

mean values based on three independent experiments, and error bars indicate s.d.

(**c**) MEF cells were treated as described in Fig. 4d and IL6 production was measured by qPCR.

(**d**) Immunoblotting to show the expression levels of Flag-MAVS as indicated in Fig. 4d.

(**e**) Immunoblotting to show the expression levels of Flag-Ube2N and Flag-Ube2D3 as indicated in Fig. 4f.

## Supplementary Figure 5

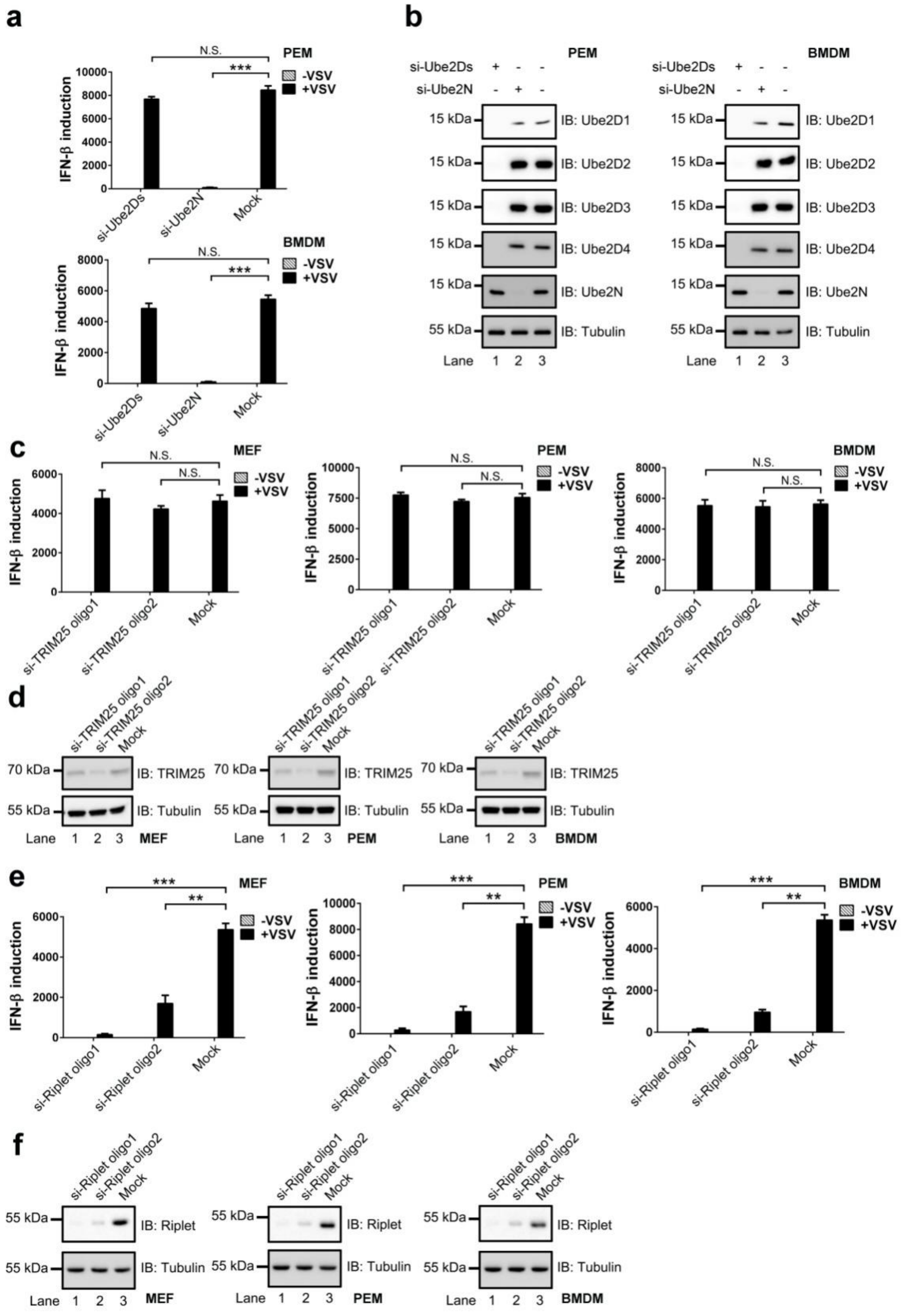


**Supplementary Figure 5 ｜ Ube2N is required for interferon production in mouse primary macrophages**

(**a-b**) PEM cells (up panel) and BMM cells (down panel) were transfected with or without si-Ube2Ds and si-Ube2N oligoes as indicated, forty-eight hours after

transduction, the cells were infected with VSV. Six hours post infection, the cells were harvested and IFN β production was measured by qPCR. Immunoblotting were also performed to examine knockdown efficiency (b), Right panel indicates data from PEM cells and left panel indicates data from BMM cells. *P<0.05 and***P<0.001. NS indicates no statistically significant difference.

(**c-f**) MEF cells (left panel), PEM cells (middle panel) and BMM cells (right panel) were transfected with or without si-TRIM25 (c) or si-Riplet (e) oligoes. Forty eight hours after transfection, the cells were infected with VSV. Six hours after viral infection, the cells were harvested for measuring IFN induction by qPCR. *P<0.05 and***P<0.001. Immunoblotting to show the knock down efficiency was performed (d, f).

## Supplementary Figure 6

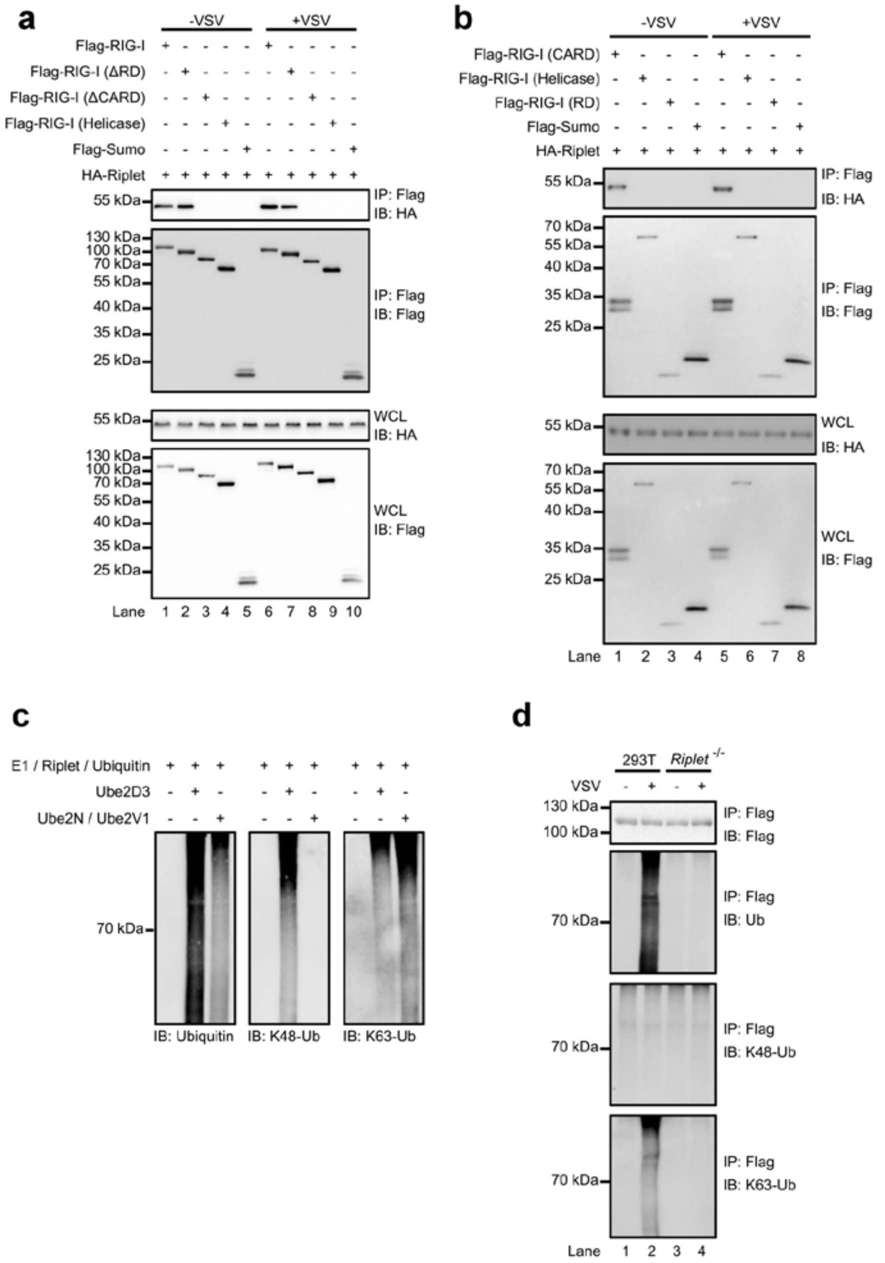


**Supplementary Figure 6 | RIG-I associates with Riplet and ubiquitination of RIG-I in response to viral infection is dependent on Riplet.**

(**a-b**) pcDNA3-flag-sumo, pcDNA3-flag-RIG-I (full length and different truncations) and pcDNA3-HA-Riplet were transfected into HEK293T cells as

indicated. Twenty four hours after transfection, the cells were infected with or without VSV. The cells were harvested twelve hours post infection and lysed for IP with M2 beads. IP products and whole cell lysate (WCL) were subjected to immunoblotting.

(**c**) The ubiquitination assay was performed with or without purified components (E1, Riplet, Ubiquitin, Ube2D3 and Ube2N/Ube2V1) as indicated. After one hour incubation, immunoblotting was performed with different ubiquitin antibodies as indicated.

(**d**) pcDNA3-flag-RIG-I was transfected into HEK293T cells (WT and *Riplet*$^{-/-}$) Twenty four hours after transfection, the cells were infected with or without VSV. The cells were harvested twelve hours post infection and lysed for IP with M2 beads. IP products and whole cell lysate (WCL) were subjected to immunoblotting.

## Supplementary Figure 7

a

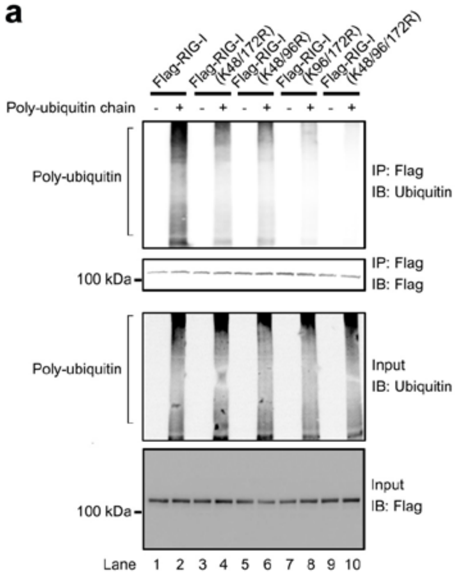


**Supplementary Figure 7 | Ube2N-Riplet pair specifically synthesized K63-linked polyubiquitin chains and K96 and K172 sites are required for RIG-I to bind polyubiquitin chains.**

(**a**) pcDNA3-flag-RIG-I (WT or with different point mutations) were transfected

into HEK293T cells. Thirty six hours after transfection, the cells were harvested and lysed for the first IP with M2 beads and elute with 3X flag peptide. IP products were incubated with or without NEM-stopped ubiquitin in vitro assay mixture for one hour at 30℃, then the second IP were performed with M2 beads. The 2$^{nd}$ products were boiled with 1XSDS loading buffer and subjected to immunoblotting with antibodies as indicated.

## Supplementary Figure 8

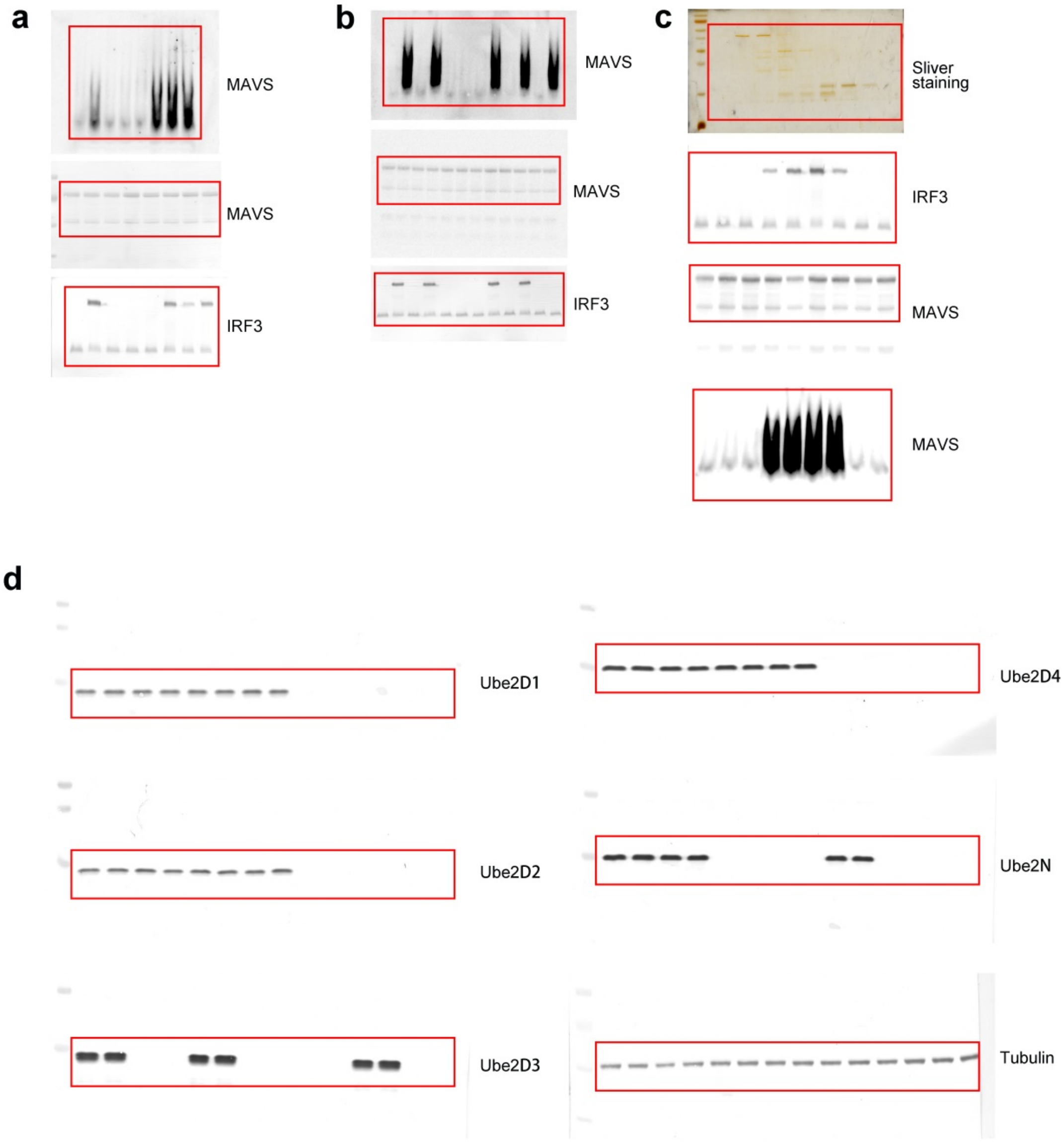


**Supplementary Figure 8 | Full blot images.**

(**a**) For Fig. 1a. (**b**) For Fig. 1d. (**c**) For Fig. 2d. (**d**) For Fig. 3e.

## Supplementary Figure 9

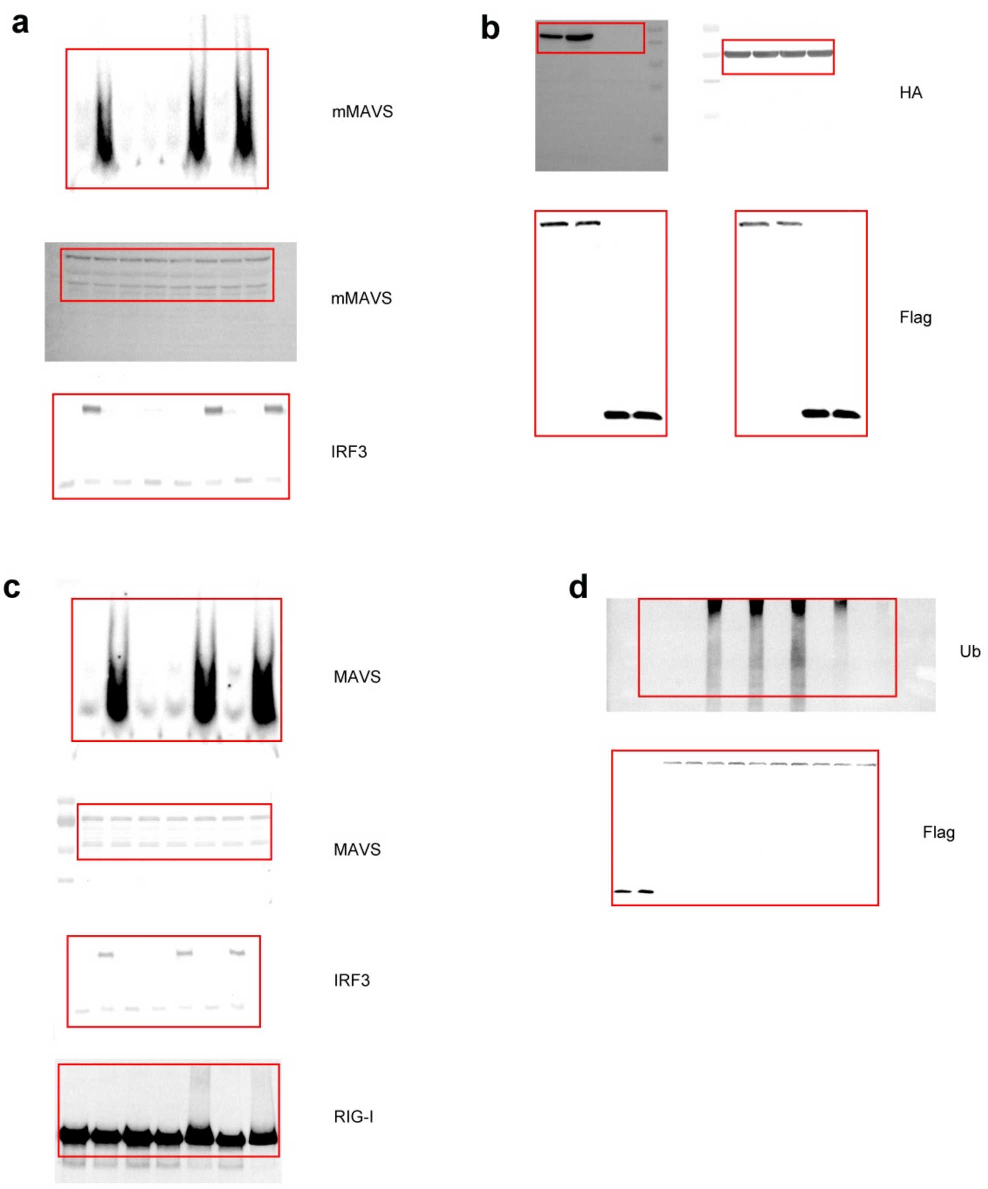


**Supplementary Figure 9 | Full blot images.**

(**a**) For Fig. 4e. (**b**) For Fig. 5a. (**c**) For Fig. 6a. (**d**) For Fig. 7d.

**Supplementary Table 1. Peptides identified by mass spectrometry related to Fig. 2d**

| Proteins | Number of peptides identified | Peptide Sequence |
|---|---|---|
| UbeD1 | **3** | IAFTTK` |
| | | ELSDLQR |
| | | SQWSPALTVSK |
| UbeD2 | **5** | VAFTTR |
| | | IYKTDR |
| | | ELNDLAR |
| | | IHKELNDLAR |
| | | SQWSPALTISK |
| UbeD3 | **6** | VAFTTR |
| | | IYKTDR |
| | | ELSDLAR |
| | | TDRDKYNR |
| | | INKELSDLAR |
| | | SQWSPALTISK |
| UbeD4 | **3** | VAFTTK |
| | | ELTDLQR |
| | | SQWSPALTVSK |
| UbeM | **1** | DINELNLPK |
| UbeN | **1** | TNEAQAIETAR |
| UbeE1 | **1** | GDNIYEWR |
| UbeL3 | **4** | NAEEFTK |
| | | NAEEFTKK |
| | | ADLAEEYSK |
| | | ADLAEEYSKDR |

**Supplementary Table 2. Sequence of primers, si-RNA, sg-RNA used in this study**

| Primer Name | Sequence(5' to 3') | Purpose |
|---|---|---|
| RIG-I-For | CACCGTCGCTGCTCGGTGGTCATGC | CRISPR/ Cas9 knock-out cell line |
| RIG-I-Rev | AAACGCATGACCACCGAGCAGCGAC | |
| RIG-I(mouse)-For | CACCGACAGAATGACAGCGGAGCAG | |
| RIG-I(mouse)-Rev | AAACCTGCTCCGCTGTCATTCTGTC | |
| TBK1-For | CACCCATAAGCTTCCTTCGTCCAG | |
| TBK1-Rev | AAACCTGGACGAAGGAAGCTTATG | |
| Riplet-For | CACCGGGCGTCGCGGGCGCCCCAC | |
| Riplet-Rev | AAACGTGGGGCGCCCGCGACGCCC | |
| Riplet(mouse)-For | CACCGACCTGTGGGTCTCGAAGCGT | |
| Riplet(mouse)-Rev | AAACACGCTTCGAGACCCACAGGTC | |
| TRIM25-For | CACCGGTCGTGCCTGAATGAGACG | |
| TRIM25-Rev | AAACCGTCTCATTCAGGCACGACC | |
| TRIM25(mouse)-For | CACCGGACGACCCACGTCTCATCC | |
| TRIM25(mouse)-Rev | AAACGGATGAGACGTGGGTCGTCC | |
| Mex3c-For | CACCGTTTCGAGATGCACGAATCA | |
| Mex3c-Rev | AAACTGATTCGTGCATCTCGAAAC | |
| TRIM4-For | CACCGCGGCTGCCTGCACCGCAACT | |
| TRIM4-Rev | AAACAGTTGCGGTGCAGGCAGCCGC | |
| Ube2D1-For | CACCGACTGGCAAGCCACTATTATG | |
| Ube2D1-Rev | AAACCATAATAGTGGCTTGCCAGTC | |
| Ube2D2-For | CACCGGACCTGCTGAACACTGTGC | |
| Ube2D2-Rev | AAACGCACAGTGTTCAGCAGGTCC | |
| Ube2D3-For | CACCGACATTGTGCTGGAGGGTCA | |
| Ube2D3-Rev | AAACTGACCCTCCAGCACAATGTC | |
| Ube2D4-For | CACCGGCTTGAACGGGTAATCTGT | |
| Ube2D4-Rev | AAACACAGATTACCCGTTCAAGCC | |
| Ube2E1-For | CACCGCTGGTTGGAAGACGAAGATG | |
| Ube2E1-Rev | AAACCATCTTCGTCTTCCAACCAGC | |
| Ube2N-For | CACCGTAACGGGCGTTGCTCTCATC | |
| Ube2N-Rev | AAACGATGAGAGCAACGCCCGTTAC | |
| Ube2D1-For | AAAGGATCCATGGCGCTGAAGAGGATTCAG | cDNA cloning |
| Ube2D1-Rev | TTTCTCGAGTTACATTGCATATTTCTGAGTCCAT | |
| Ube2D2-For | AAAGGATCCATGGCTCTGAAGAGAATCCAC | |
| Ube2D2-Rev | TTTCTCGAGTTACATCGCATACTTCTGAGTCCATTC CCGAGC | |
| Ube2D3-For | AAAGGATCCATGGCGCTGAAACGGATTAAT | |
| Ube2D3-Rev | TTTCTCGAGTCACATGGCATACTTCTGAGTCCATTC CCGAGA | |

| | | |
|---|---|---|
| Ube2D4-For | AAGGATCCATGGCGCTAAAGCGCATCCAGAAGGAATTAACCGACTTGCAGAGAGATCCT | |
| Ube2D4-Rev | TTTCTCGAGTTACATAGCATATTTTTGTGTCCACTCTCTT | |
| Ube2N-For | AAAGGATCCATGGCCGGGCTGCCCCGCAG | |
| Ube2N-Rev | TTTCTCGAGTTAAATATTATTCATGGCATATAGCC | |
| Ube2D1,2,3,4-C85A-For | TAAACAGTAATGGAAGTATTGCTCTCGATATTCTGAGGTCACAATG | |
| Ube2D1,2,3,4-C85A-Rev | AGCAATACTTCCATTACTGTTTATG | |
| Ube2E1-C131A-For | TTAATAGTCAAGGTGTTATTGCTTTGGACATATTGAAGGATAATTG | |
| Ube2E1-C131A-Rev | AGCAATAACACCTTGACTATTAATATTAC | |
| Ube2N-C87A-For | TAGACAAGTTGGGAAGAATAGCTTTAGATATTTTGAAGGATAAG | |
| Ube2N-C87A-Rev | AGCTATTCTTCCCAACTTGTCTAC | |
| Ube2M-For | AAAGGATCCATGATCAAGCTGTTCTCGCTGAAG | |
| Ube2M-Rev | TTTGCGGCCGCTTATTTCAGGCAGCGCTCAAAG | |
| Ube2E1-For | TTTGGATCCATGTCGGATGACGATTCGAGG | |
| Ube2E1-Rev | TTTCTCGAGTTATGTAGCGTATCTCTTGGTCC | |
| Riplet-For | TTTGAATTCGCGGGCCTGGGCCTGGGCTCCG | |
| Riplet-Rev | TTTCTCGAGTTACACCTTTACTTGCTTTATTATCAGG | |
| RIG-I-For | TATTCTAGAATGACCACCGAGCAGCGACGCAG | |
| RIG-I-Rev | CGCCTCGAGTCATTTGGACATTTCTGCTGGATC | |
| TRIM25-For | ATATCTAGAATGGCAGAGCTGTGCCCCCTGG | |
| TRIM25-Rev | ATACTCGAGCTACTTGGGGGAGCAGATGGAGAG | |
| ube2d1-shRNA-For | CCGGAAGATTGCTTTCACAACAAAACTCGAGTTTTGTTGTGAAAGCAATCTTTTTTTG | Si-RNA and sh-RNA |
| ube2d1-shRNA-Rev | AATTCAAAAAAAGATTGCTTTCACAACAAAACTCGAGTTTTGTTGTGAAAGCAATCTT | |
| ube2d3-shRNA-For | CCGGAACAGTAATGGCAGCATTTGTCTCGAGACAAATGCTGCCATTACTGTTTTTTTG | |
| ube2d3-shRNA-Rev | AATTCAAAAAAACAGTAATGGCAGCATTTGTCTCGAGACAAATGCTGCCATTACTGTT | |
| ube2d4-shRNA-For | CCGGAACAGTAATGGCAGCATTTGTCTCGAGACAAATGCTGCCATTACTGTTTTTTG | |
| ube2d4-shRNA-Rev | AATTCAAAAAAACAGTAATGGCAGCATTTGTCTCGAGACAAATGCTGCCATTACTGTT | |
| Ube2n-shRNA-For | CCGGAAGTACGTTTCATGACCAAAACTCGAGTTTTGGTCATGAAACGTACTTTTTTTG | |
| Ube2n-shRNA-Rev | AATTCAAAAAAAGTACGTTTCATGACCAAAACTCGAGTTTTGGTCATGAAACGTACTT | |
| UBE2D3-siRNA-For | CAGTAATGGCAGCATTTGT | |

| | | |
|---|---|---|
| UBE2D3-siRNA-Rev | ACAAATGCTGCCATTACTG | |
| TRIM25-siRNA-h-1 | ggaaaagaaaUccaagaaa | |
| TRIM25-siRNA-h-2 | ggUggagcagcUacaacaa | |
| TRIM25-siRNA-m-1 | gcaacagUgUgUgcaggaU | |
| TRIM25-siRNA-m-2 | ggcagaggUUgagcUcaUU | |
| Riplet-siRNA-m-1 | ccUgaggacccUcUccUaU | |
| Riplet-siRNA-m-1 | ggacgaccUgagcUgcaUU | |
| GAPDH-For | AGAAGGCTGGGGCTCATTTG | qPCR |
| GAPDH-Rev | AGGGGCCATCCACAGTCTTC | |
| IFN-β-For | CAGCAGTTCCAGAAGGAGGA | |
| IFN-β-Rev | AGCCAGGAGGTTCTCAACAA | |
| ISG54-For | CTGAACCGAGCCCTGCCGAAC | |
| ISG54-Rev | GCTGCCTCGTTTTGCCCTTTGAG | |
| CXCL10-For | TGGCATTCAAGGAGTACCTC | |
| CXCL10-Rev | TTGTAGCAATGATCTCAACACG | |
| CCL5-For | ATCCTCATTGCTACTGCCCTC | |
| CCL5-Rev | GCCACTGGTGTAGAAATACTCC | |
| Actin (mouse)-For | TGACGTTGACATCCGTAAAGACC | |
| Actin (mouse)-Rev | AAGGGTGTAAAACGCAGCTCA | |
| IFNα (mouse)-For | ATTTTGGATTCCCCTTGGAG | |
| IFNα (mouse)-Rev | TATGTCCTCACAGCCAGCAG | |
| IFNβ (mouse)-For | CCCTATGGAGATGACGGAGA | |
| IFNβ (mouse)-Rev | CTGTCTGCTGGTGGAGTTCA | |
| CXCL10 (mouse)-For | GGTCTGAGTGGGACTCAAGG | |
| CXCL10 (mouse)-Rev | GTGGCAATGATCTCAACACG | |
| IL-6(mouse)-For | TCCATCCAGTTGCCTTCTTG | |
| IL-6(mouse)-Rev | GGTCTGTTGGGAGTGGTATC | |
