# Supplementary material for: Erratum: Organocatalytic atroposelective synthesis of axially chiral styrenes
Source: Nat Commun. 2017 Jun 27;8:16119. doi: 10.1038/ncomms16119 (PMC5490258; doi:10.1038/ncomms16119)
Supplement: Supplementary Information [file ncomms16119-s1.pdf]

Supplementary Figures

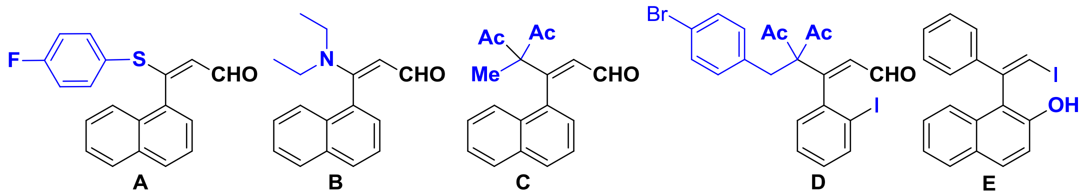

	A	B	C	D	E
Barrier	*67.8 kJ/mol*	*123.1 kJ/mol*	*106.3 kJ/mol*	*127.2 kJ/mol*	*122.2 kJ/mol*
$t_{1/2}$ (25 °C)	*0.081 s*	*4476 days*	*5 days*	*24590 days*	*3272 days*

The above computed rotation barriers along the axial C-C_{Ar} bond and their corresponding $t_{1/2}$ (25 °C) were estimated by SMD M06-D3/6-31+G*//M06-D3/6-31G* method.

Supplementary Figure 1. Initial investigations of the computed rotation barrier for the axial hirality of styrene-type compounds.

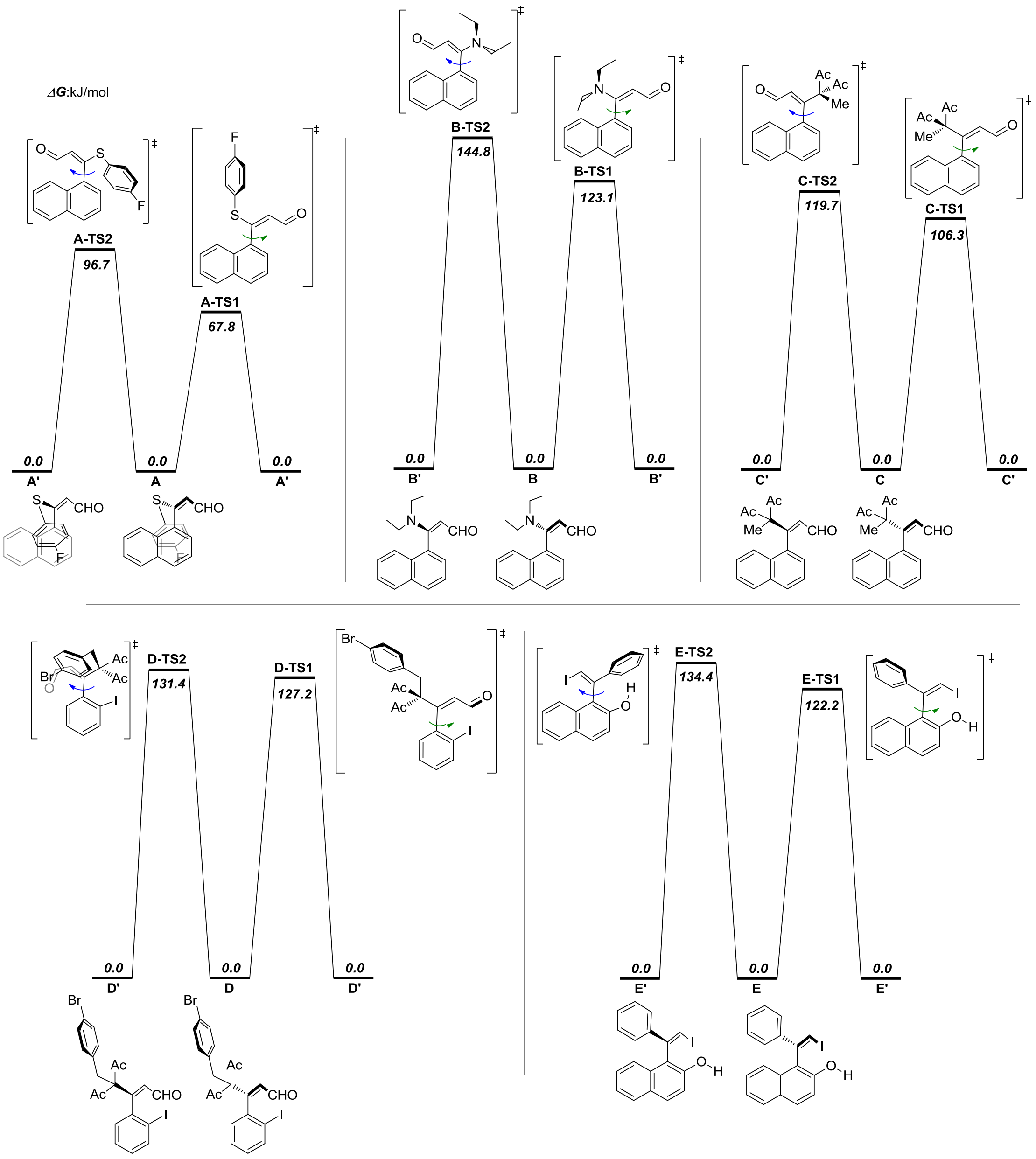

Supplementary Figure 2. Two rotation pathways to interconvert two axial chiral enantiomers for compounds **A**-**E**. Computed relative free energies in DCM by the SMD M06 method (in kJ mol^{-1}) are given.

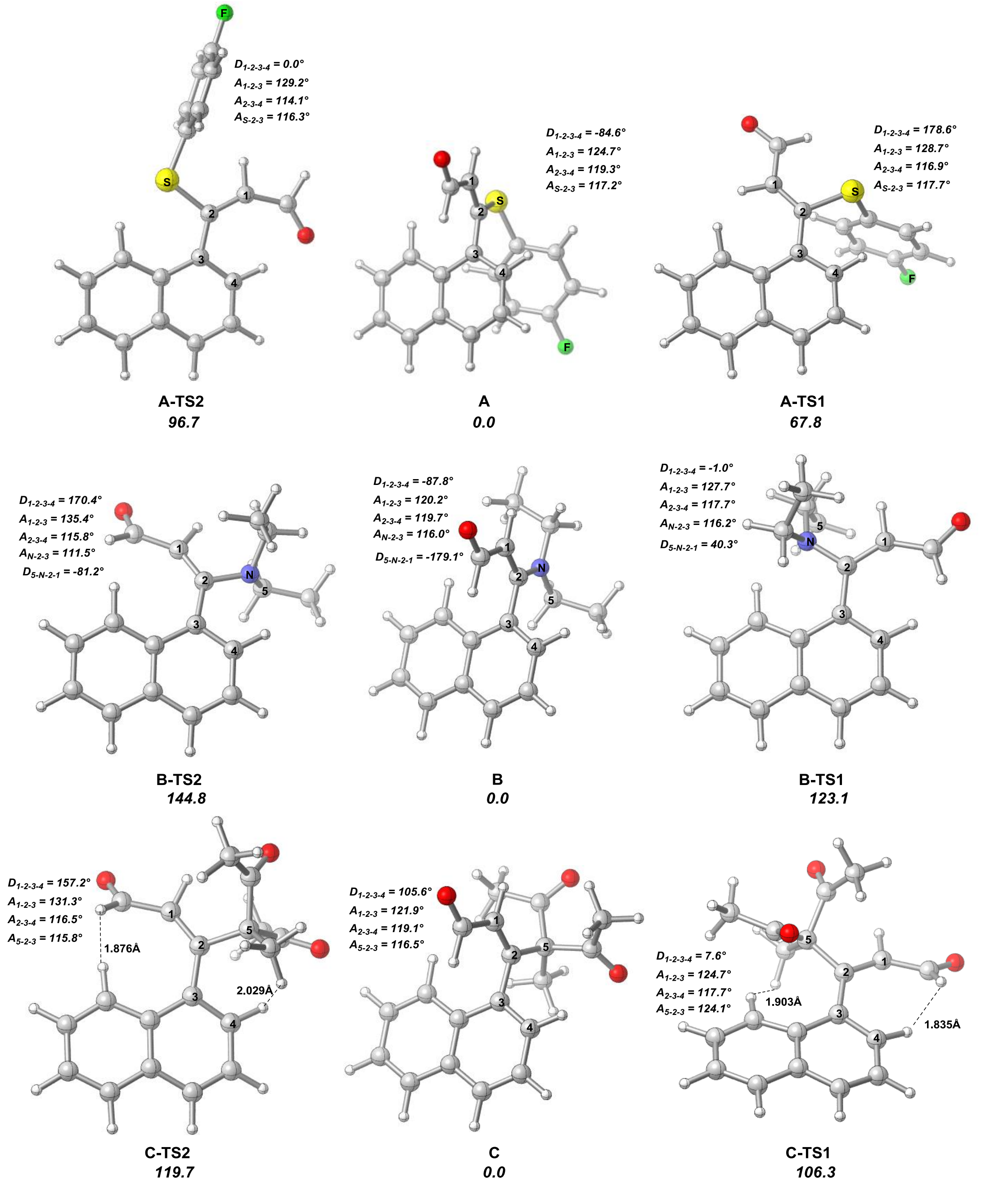

Supplementary Figure 3. Optimized structure and rotation transition states with selected structural parameters for compounds **A**, **B** and **C** at the M06/6-31G* level. Bold italic numbers are relative free energies in DCM solution (in kJ mol^{-1}).

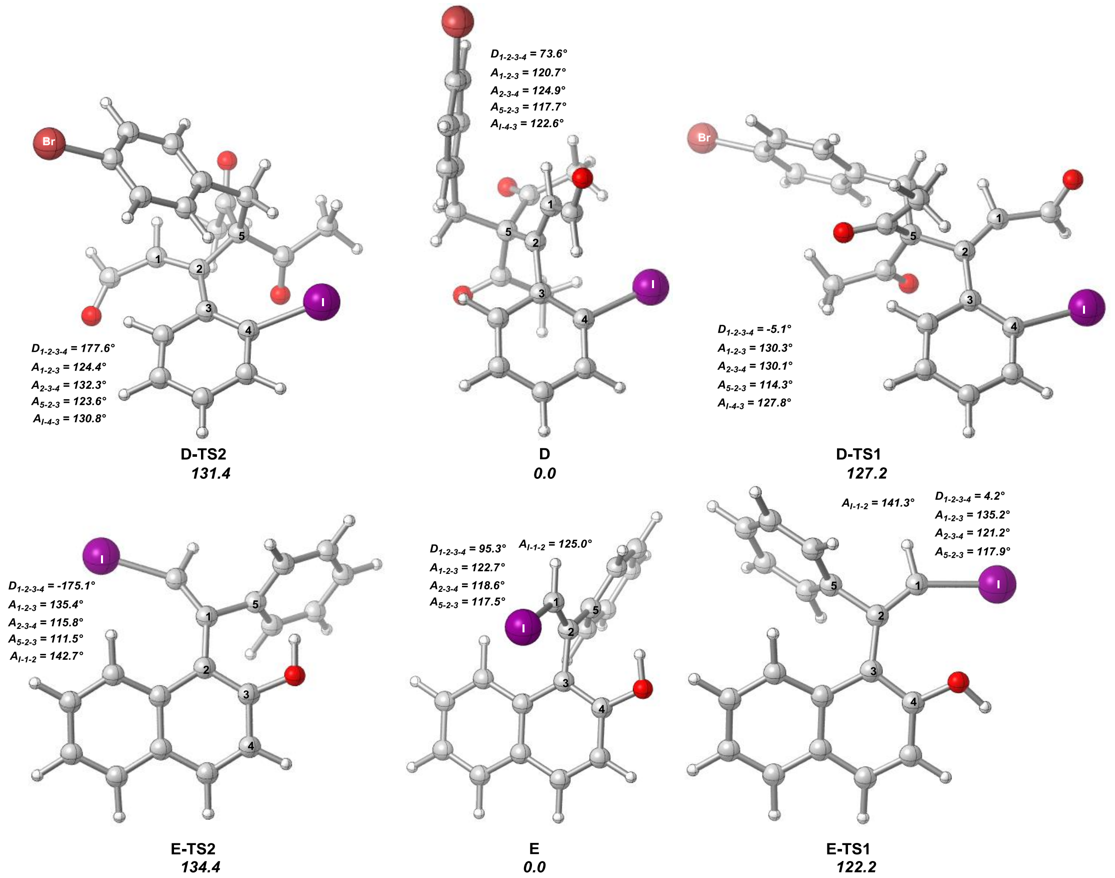

Supplementary Figure 4. Optimized structure and rotation transition states with selected structural parameters for compounds **D** and **E** at the M06/6-31G* level. Bold italic numbers are relative free energies in DCM solution (in kJ mol^{-1}).

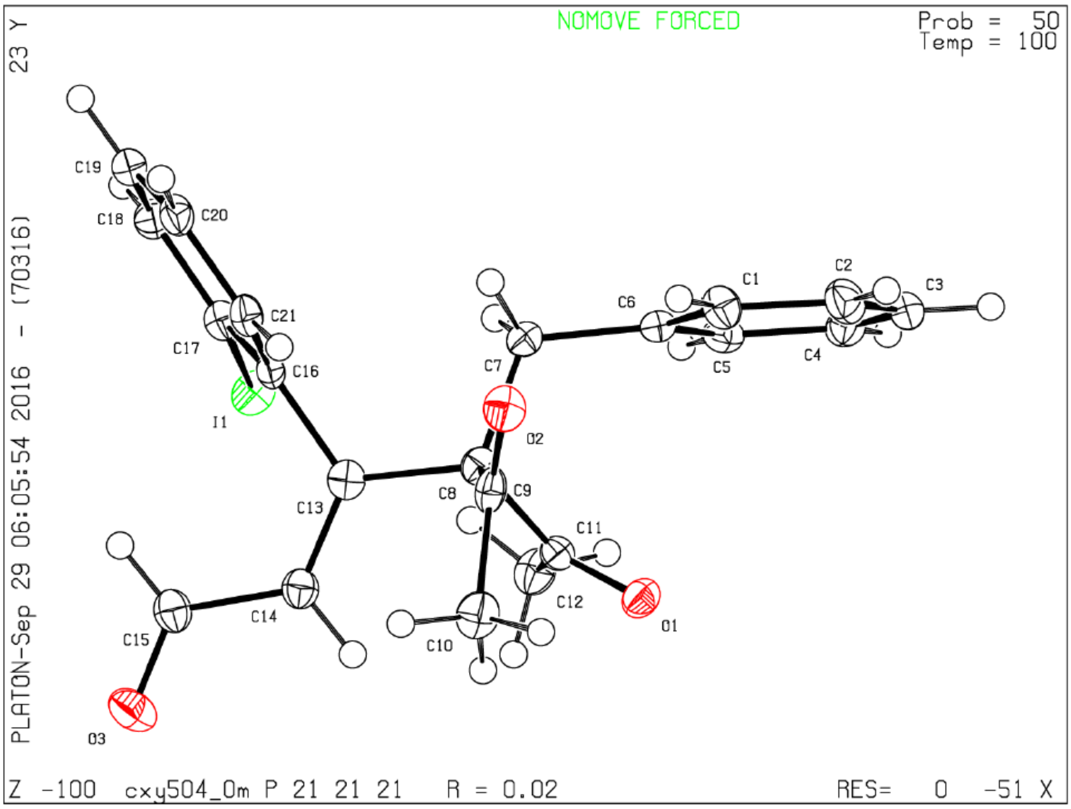

Supplementary Figure 5. X-ray Structure of **3n**.

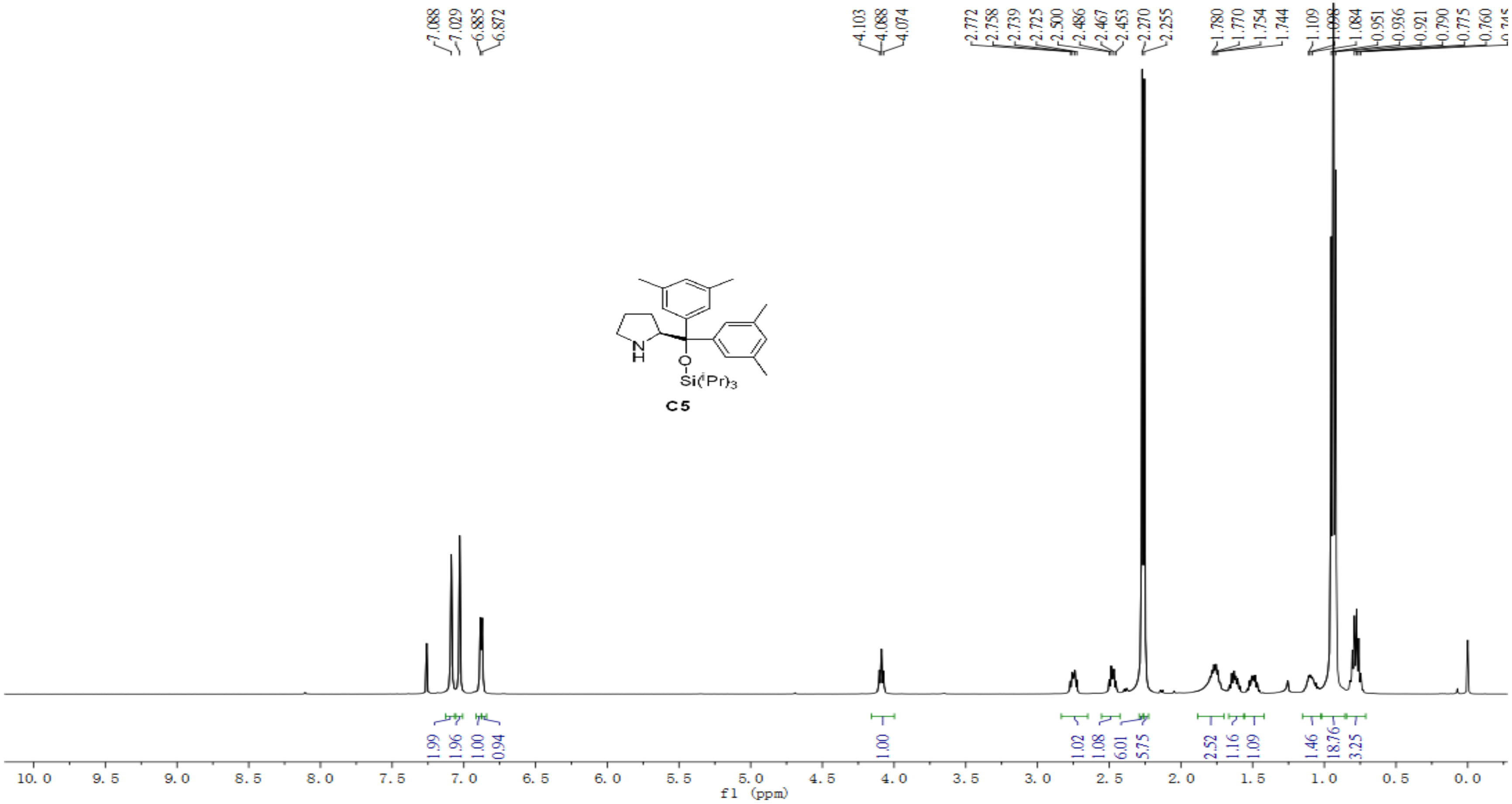

Supplementary Figure 6. ^{1}H NMR of **C5**

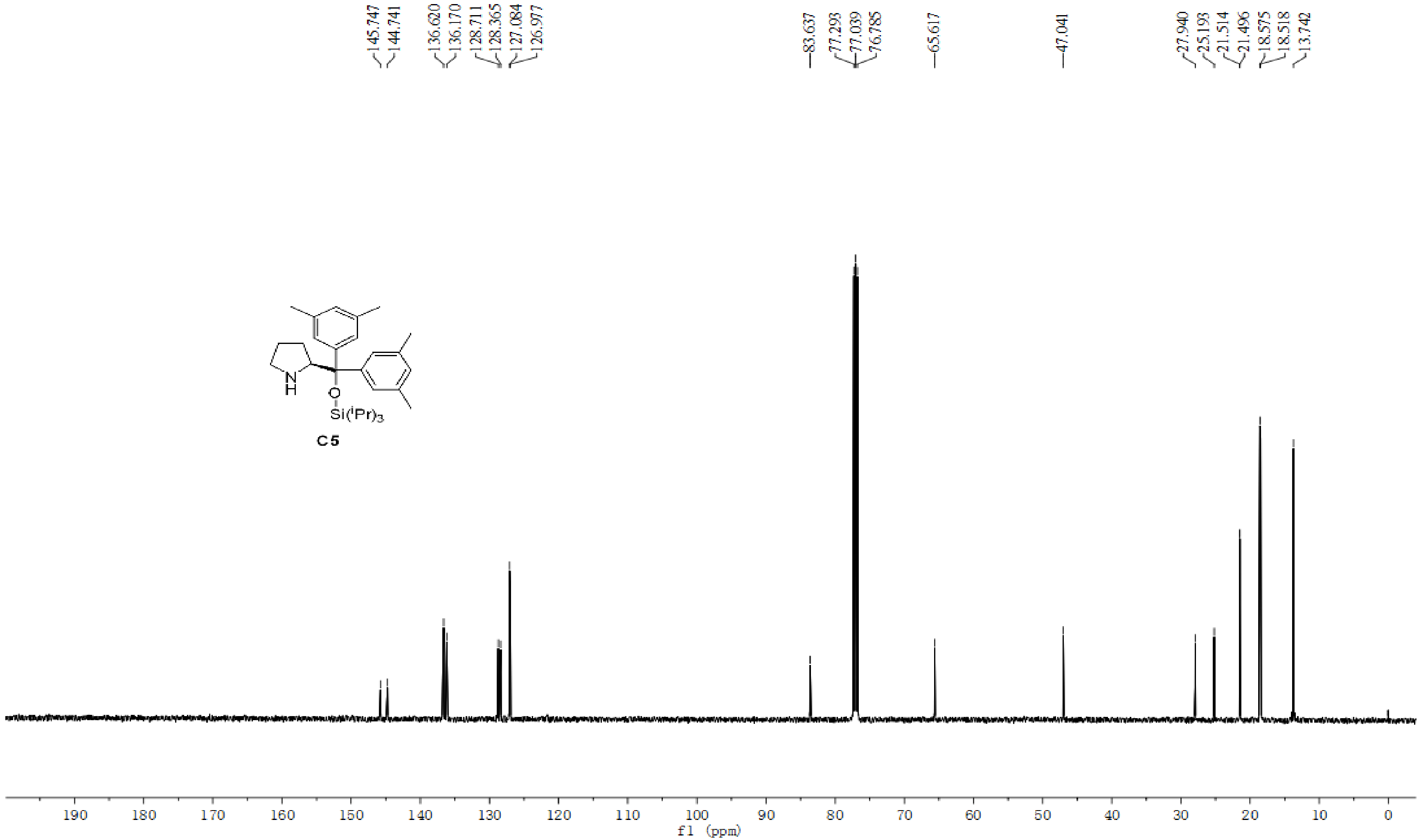

Supplementary Figure 7. ^{13}C NMR of **C5**

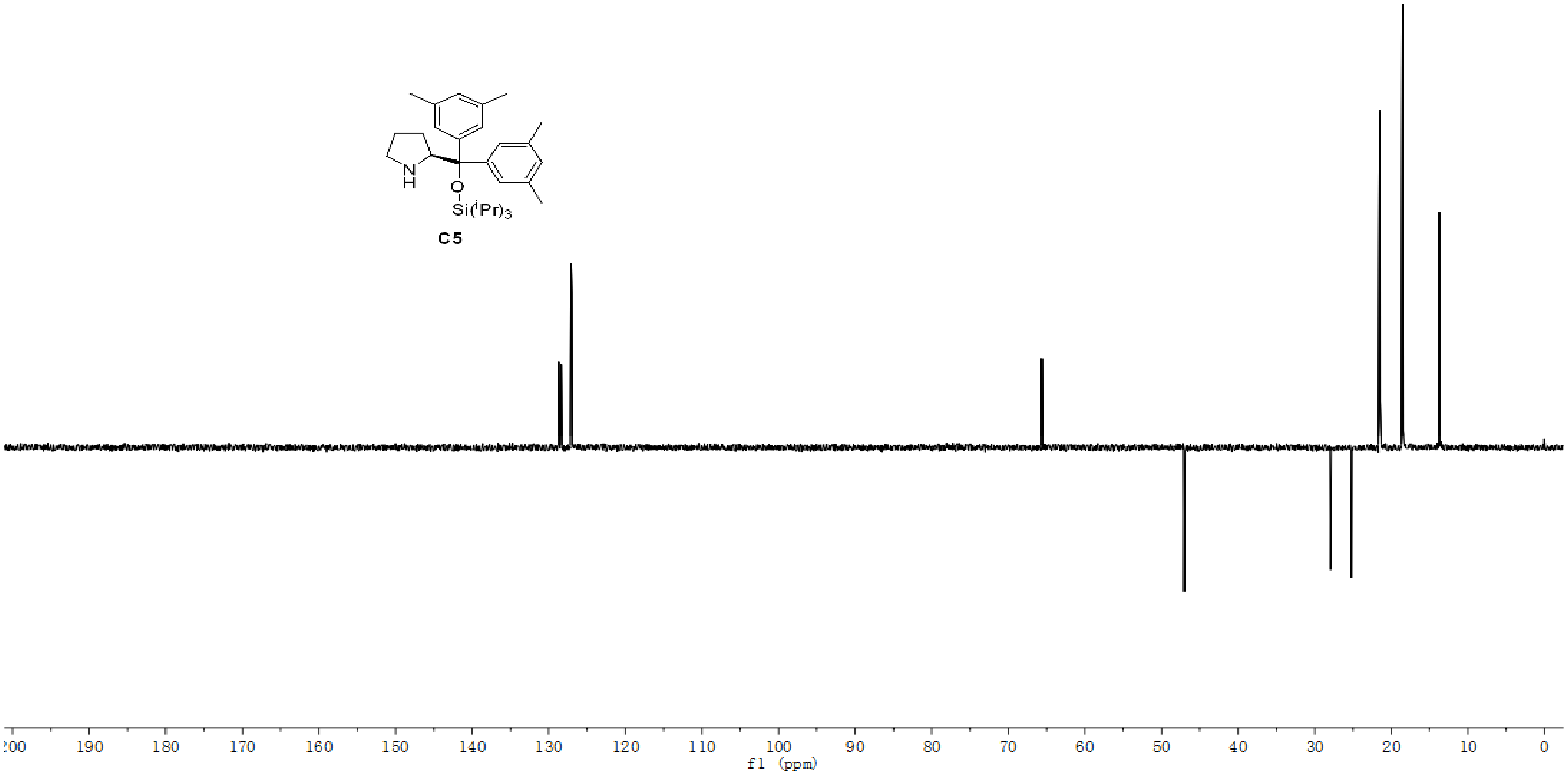

Supplementary Figure 8. DEPT-135 of **C5**

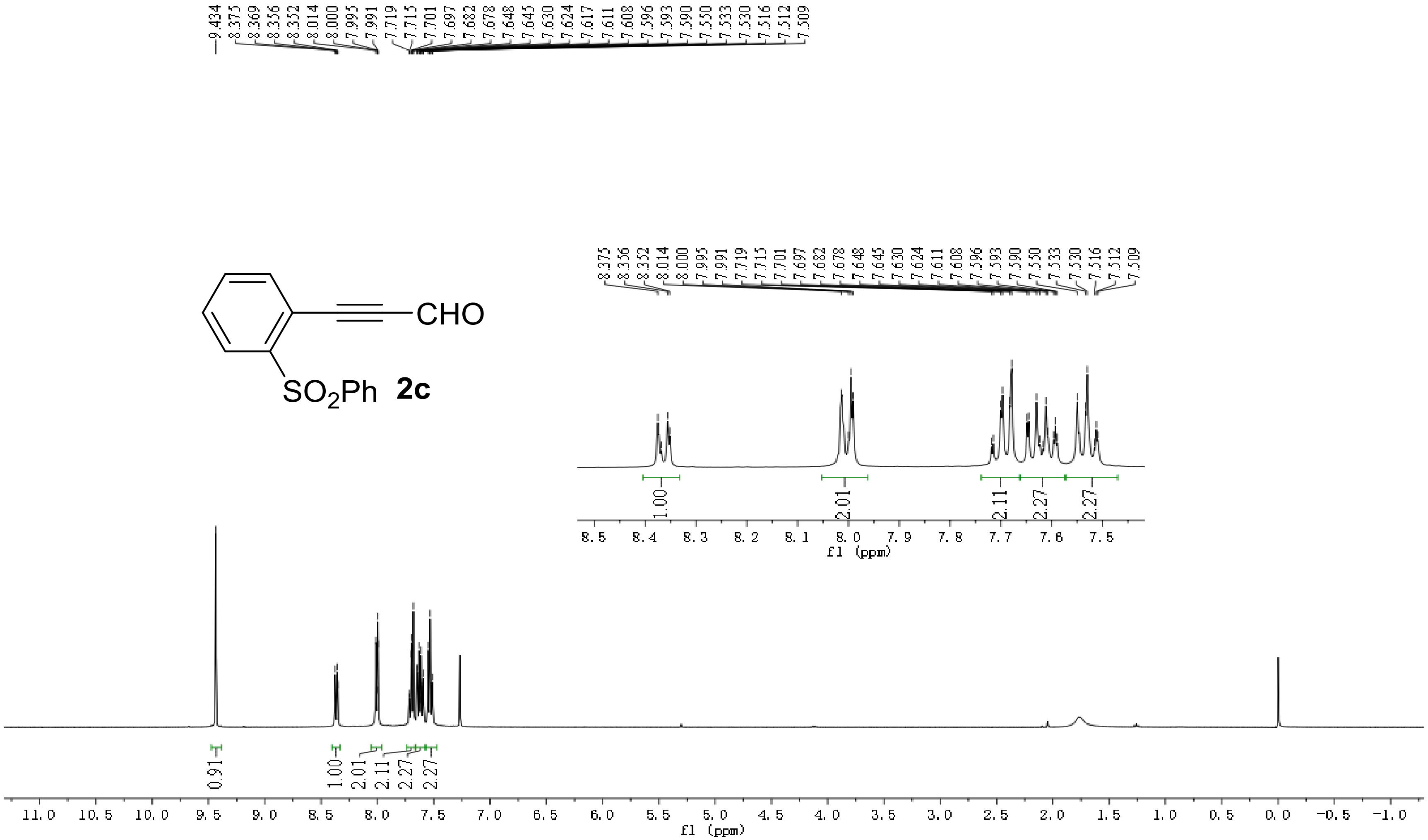

Supplementary Figure 9. ^{1}H NMR of **2c**

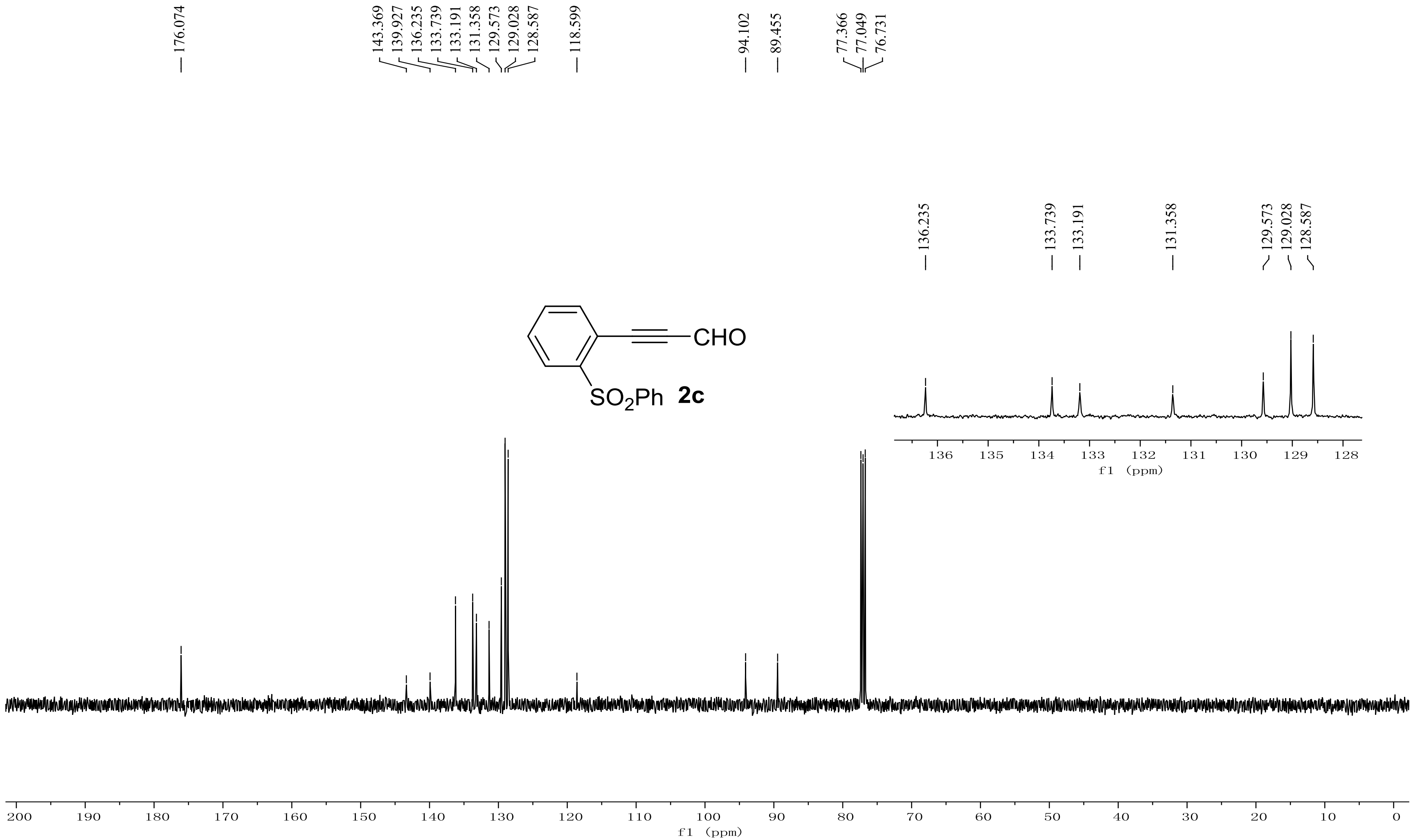

Supplementary Figure 10. ^{13}C NMR of **2c**

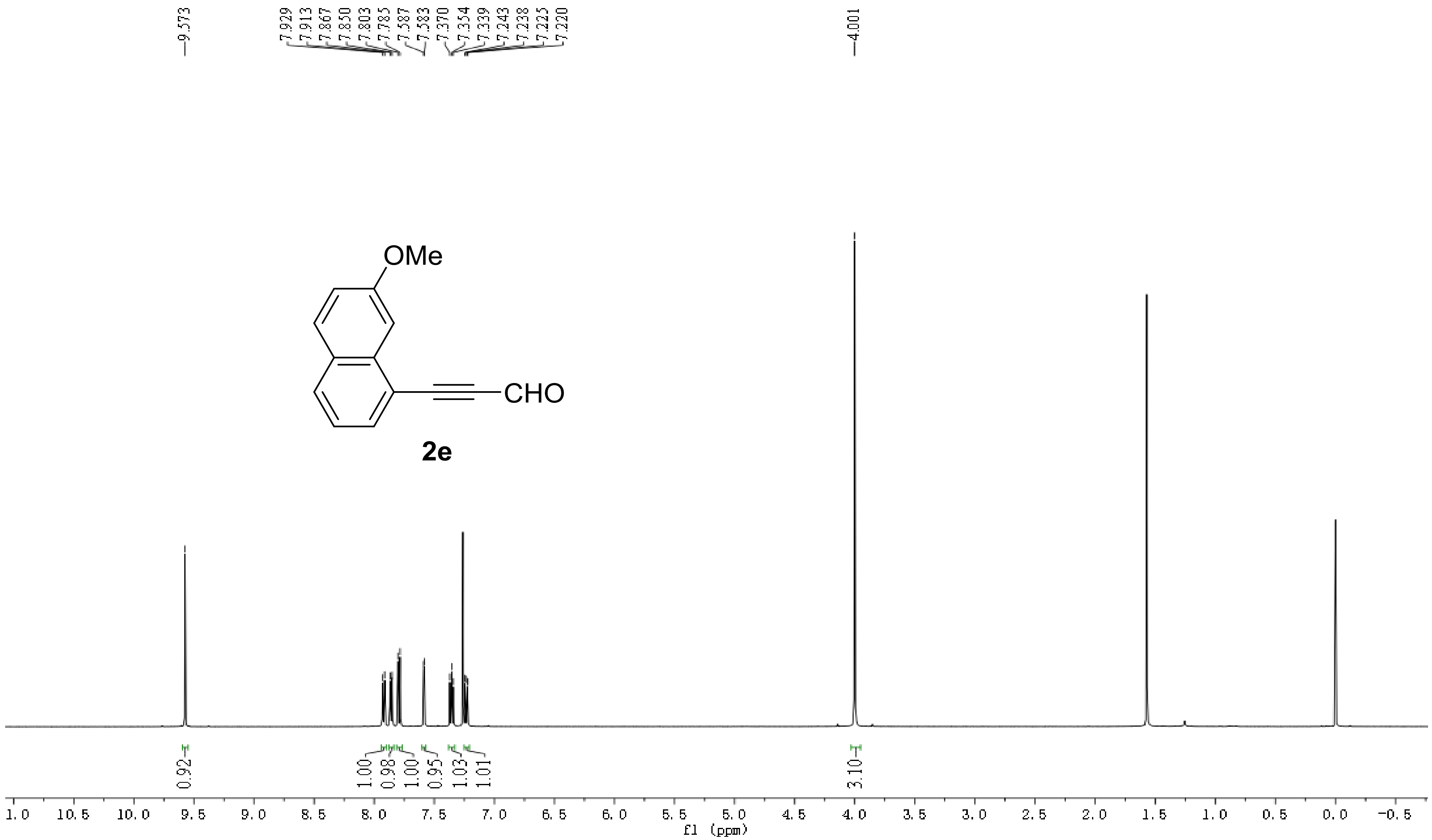

Supplementary Figure 11. ^{1}H NMR of **2e**

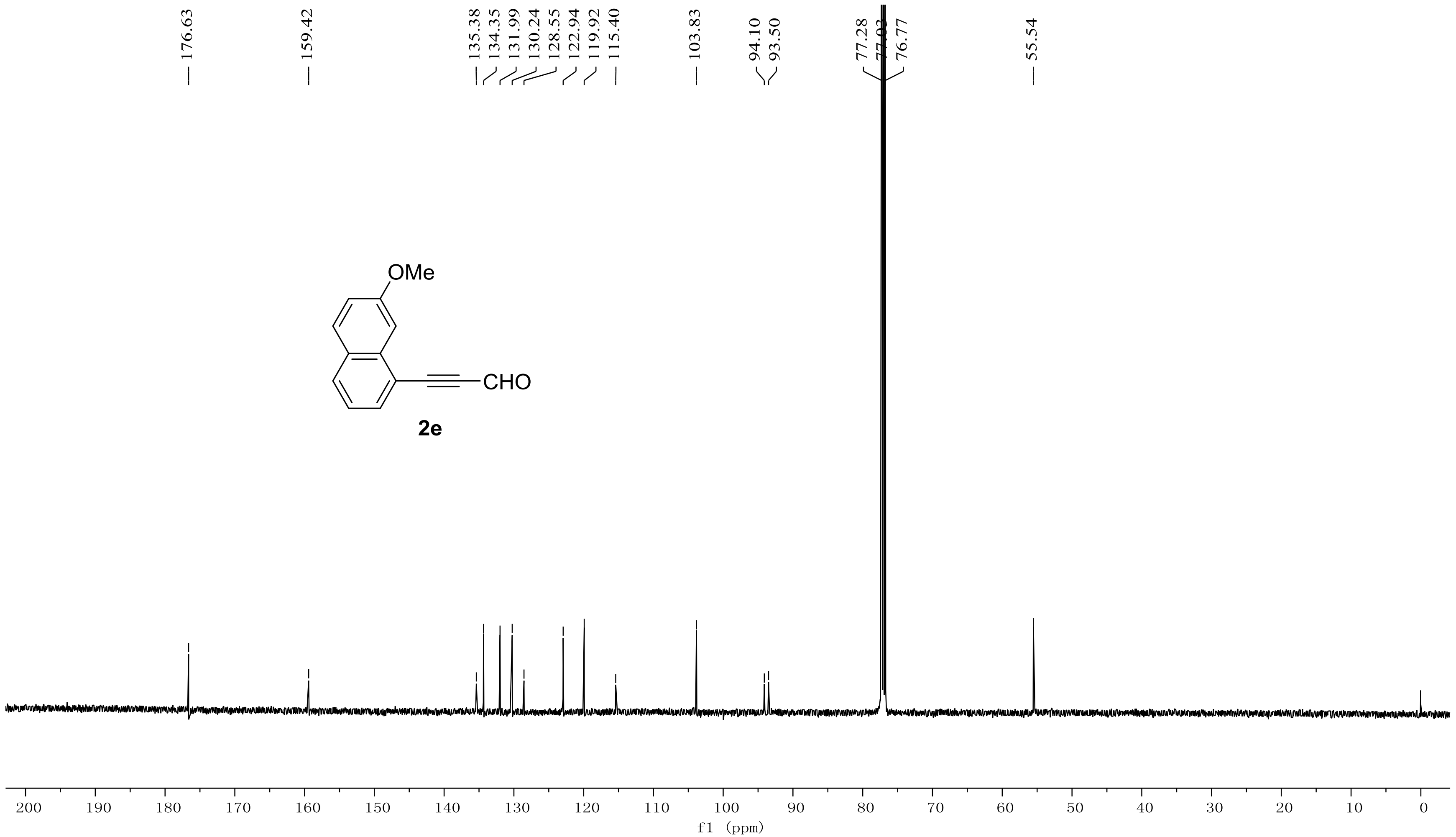

Supplementary Figure 12. ^{13}C NMR of **2e**

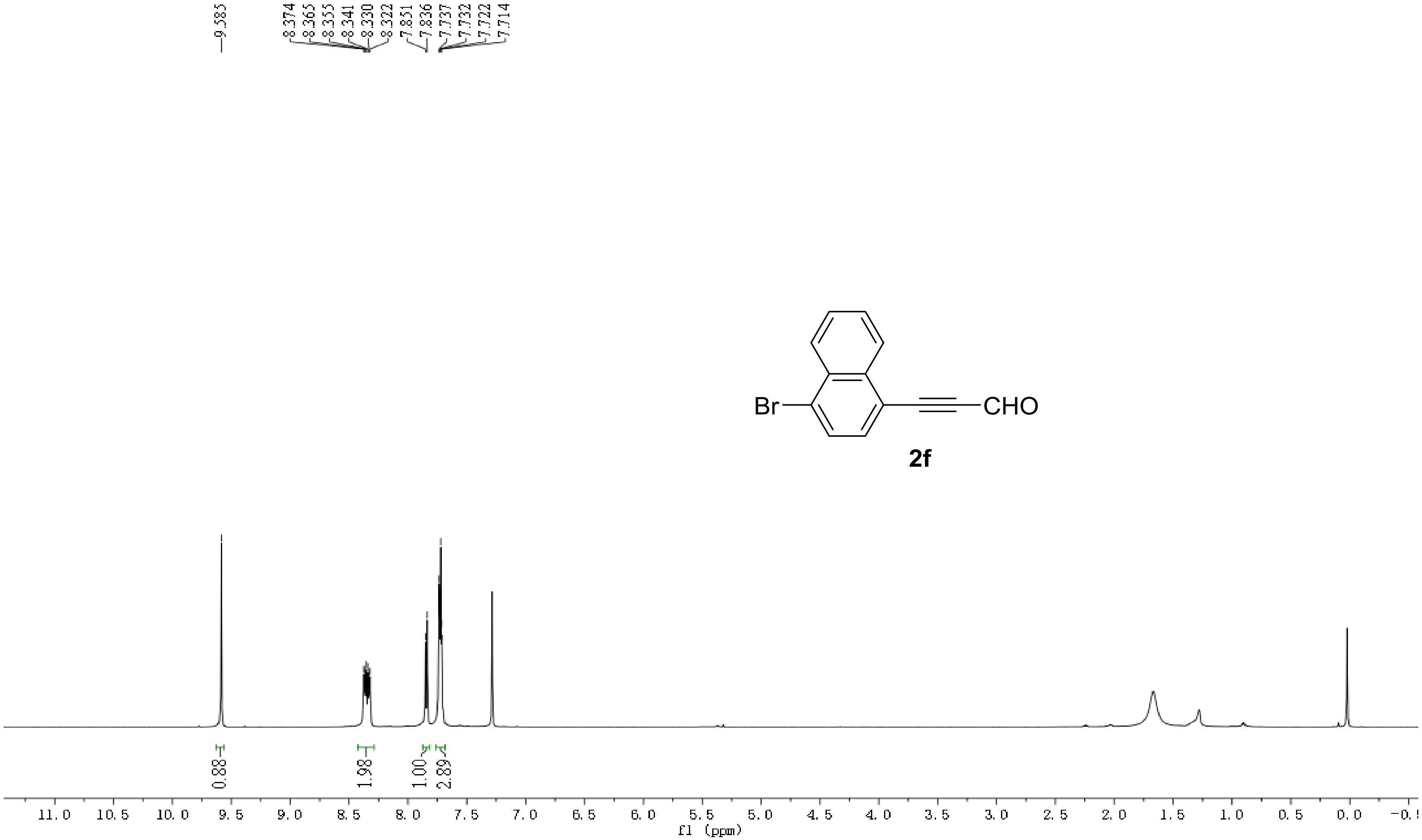

Supplementary Figure 13. ^{1}H NMR of **2f**

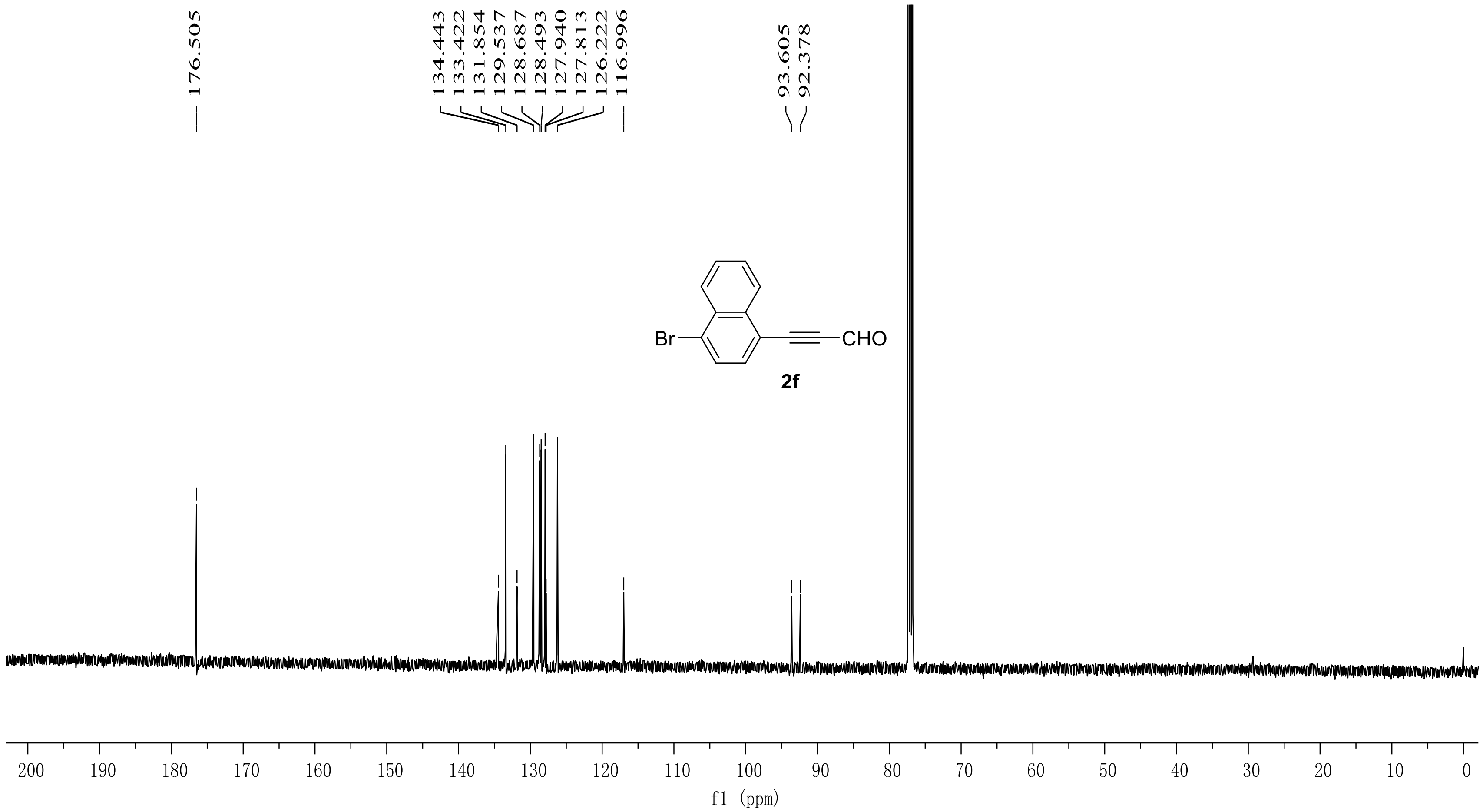

Supplementary Figure 14. ^{13}C NMR of **2f**

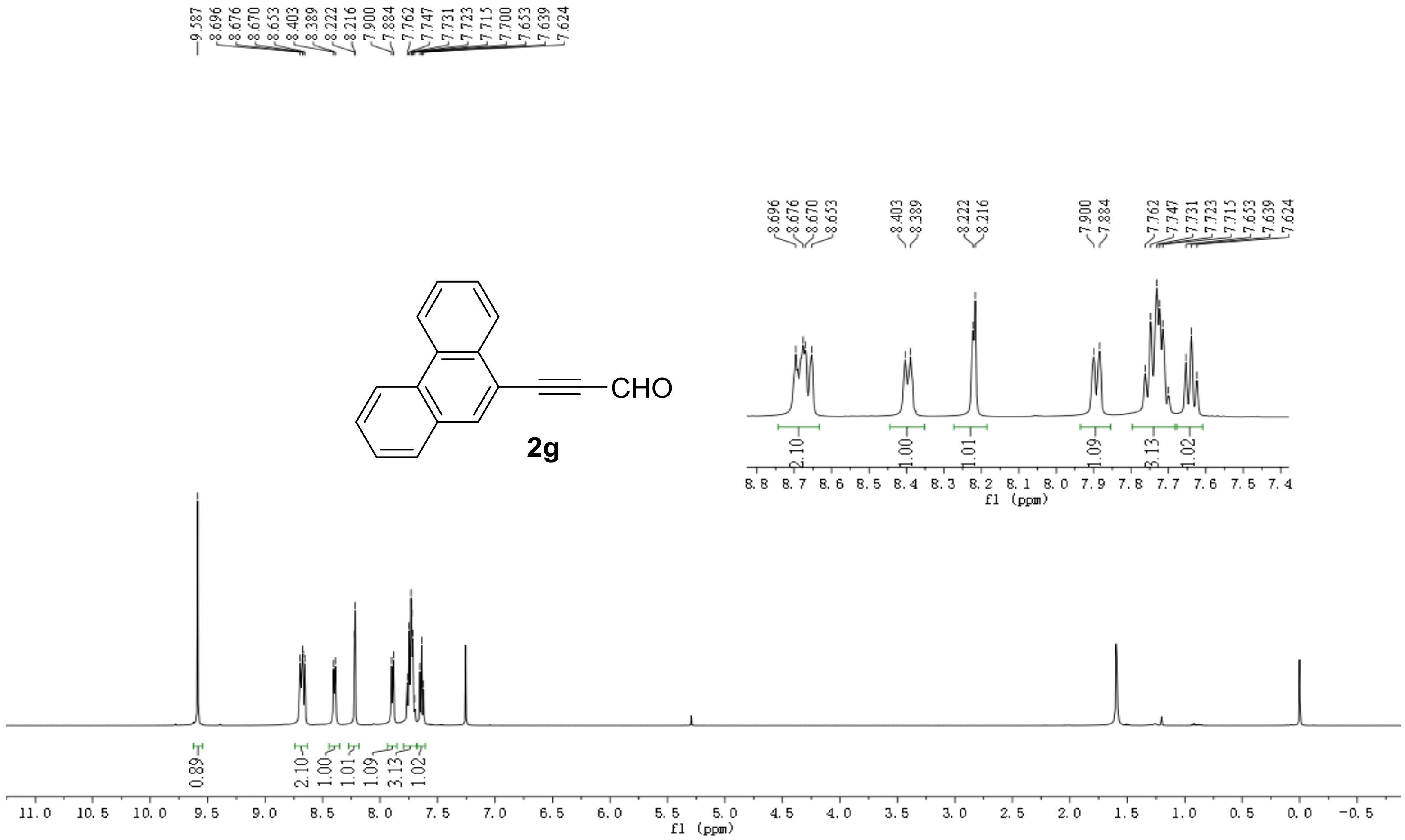

Supplementary Figure 15. ^{1}H NMR of **2g**

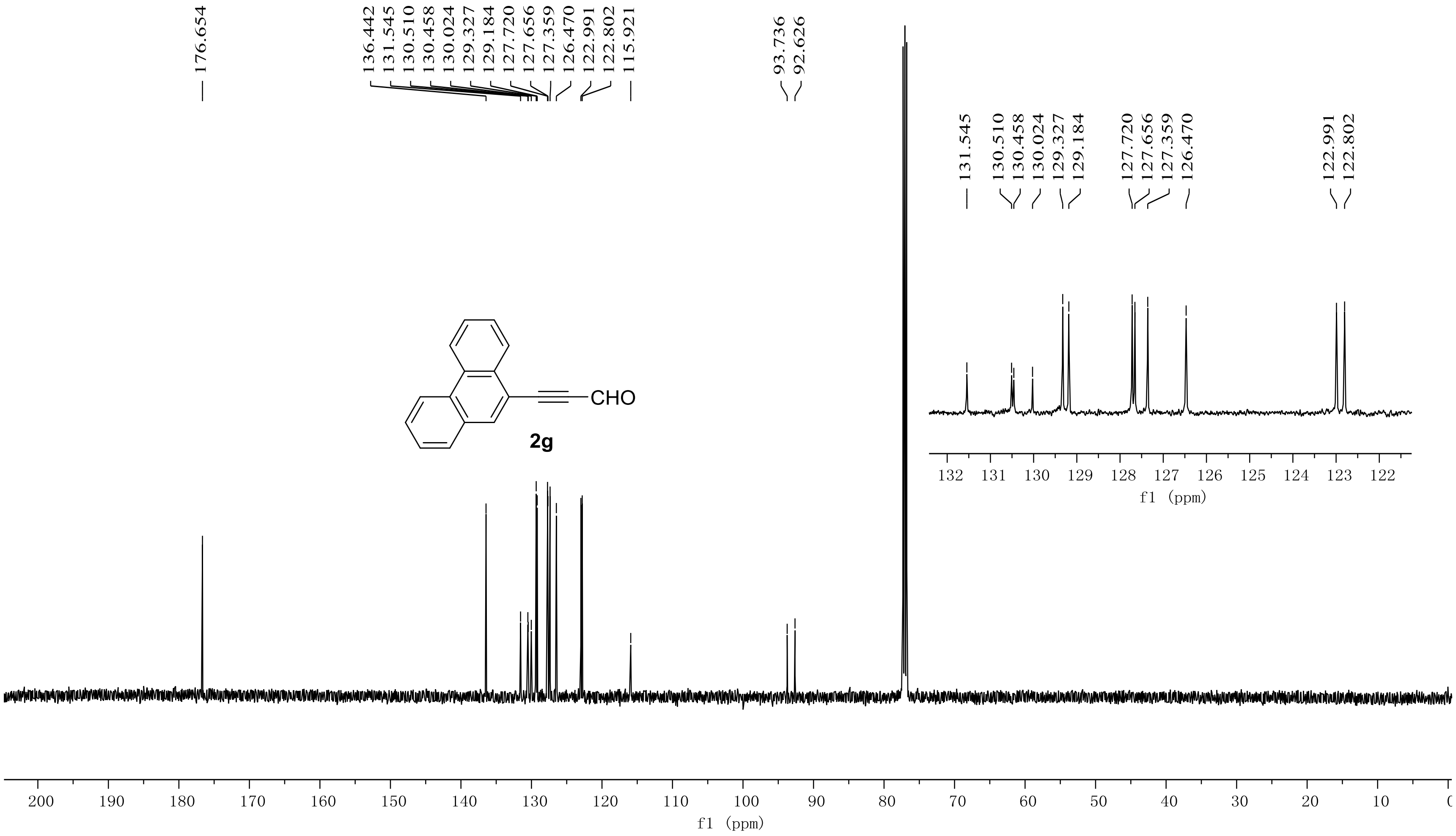

Supplementary Figure 16. ^{13}C NMR of **2g**

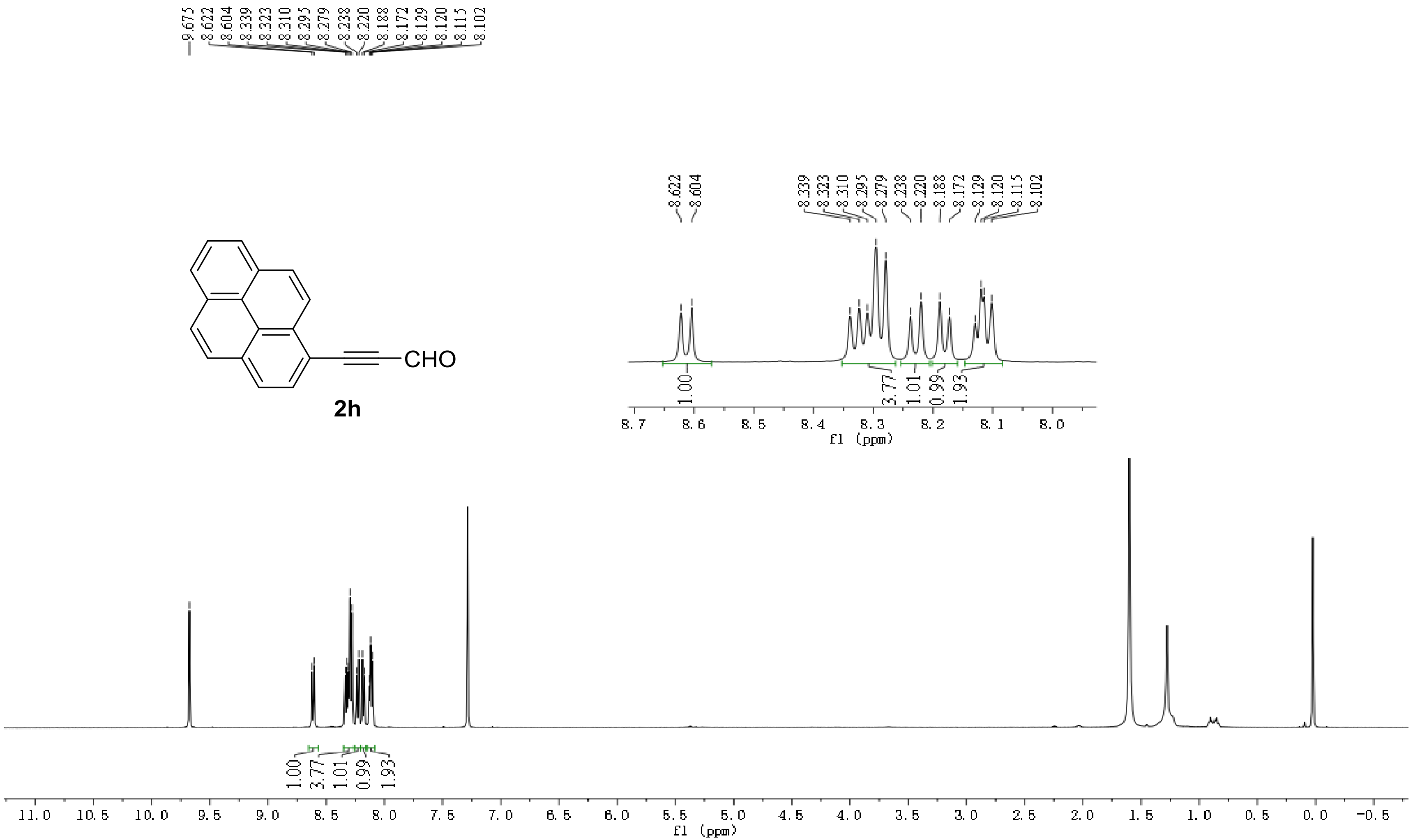

Supplementary Figure 17. ^{1}H NMR of **2h**

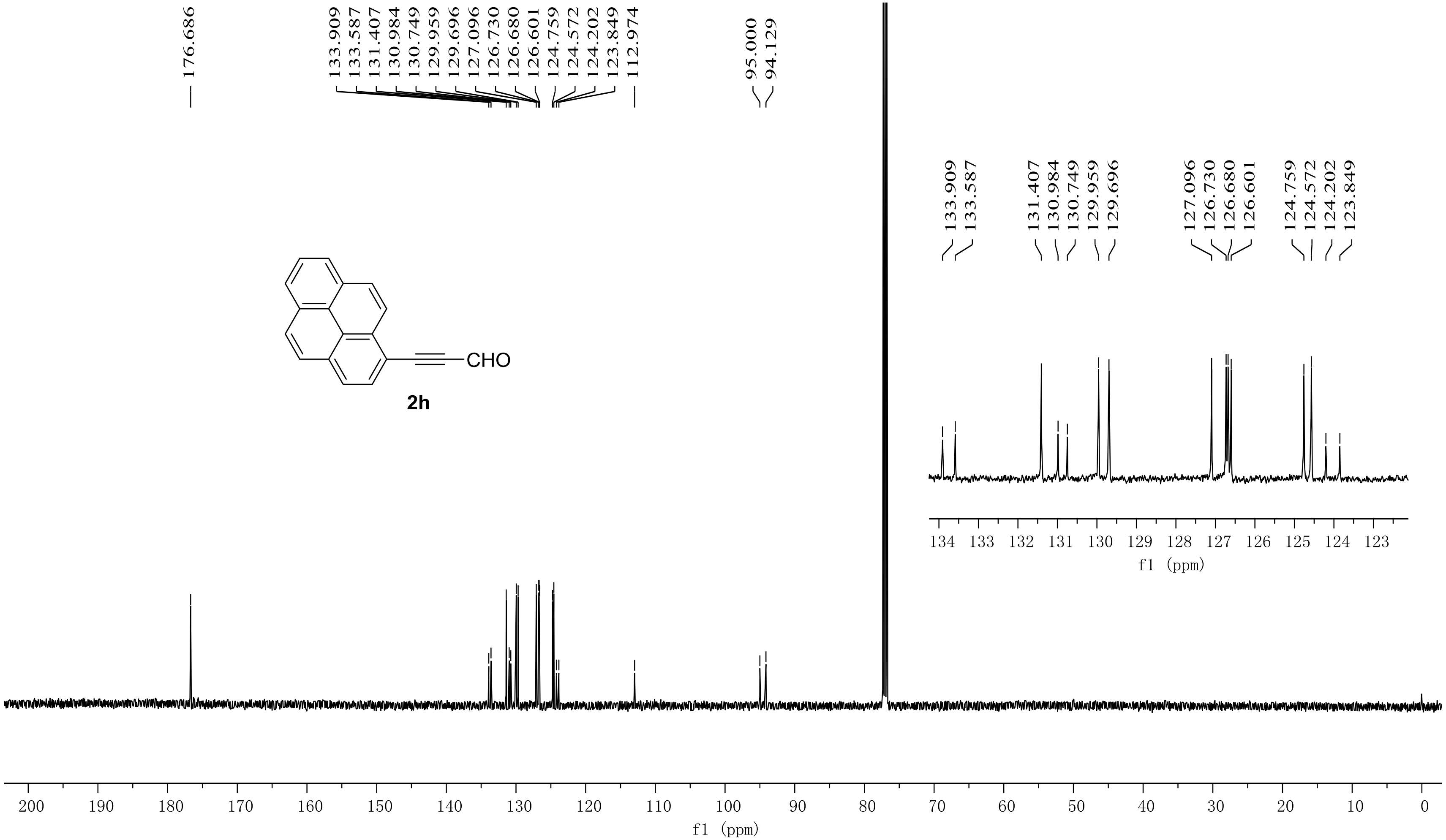

Supplementary Figure 18. ^{13}C NMR of **2h**

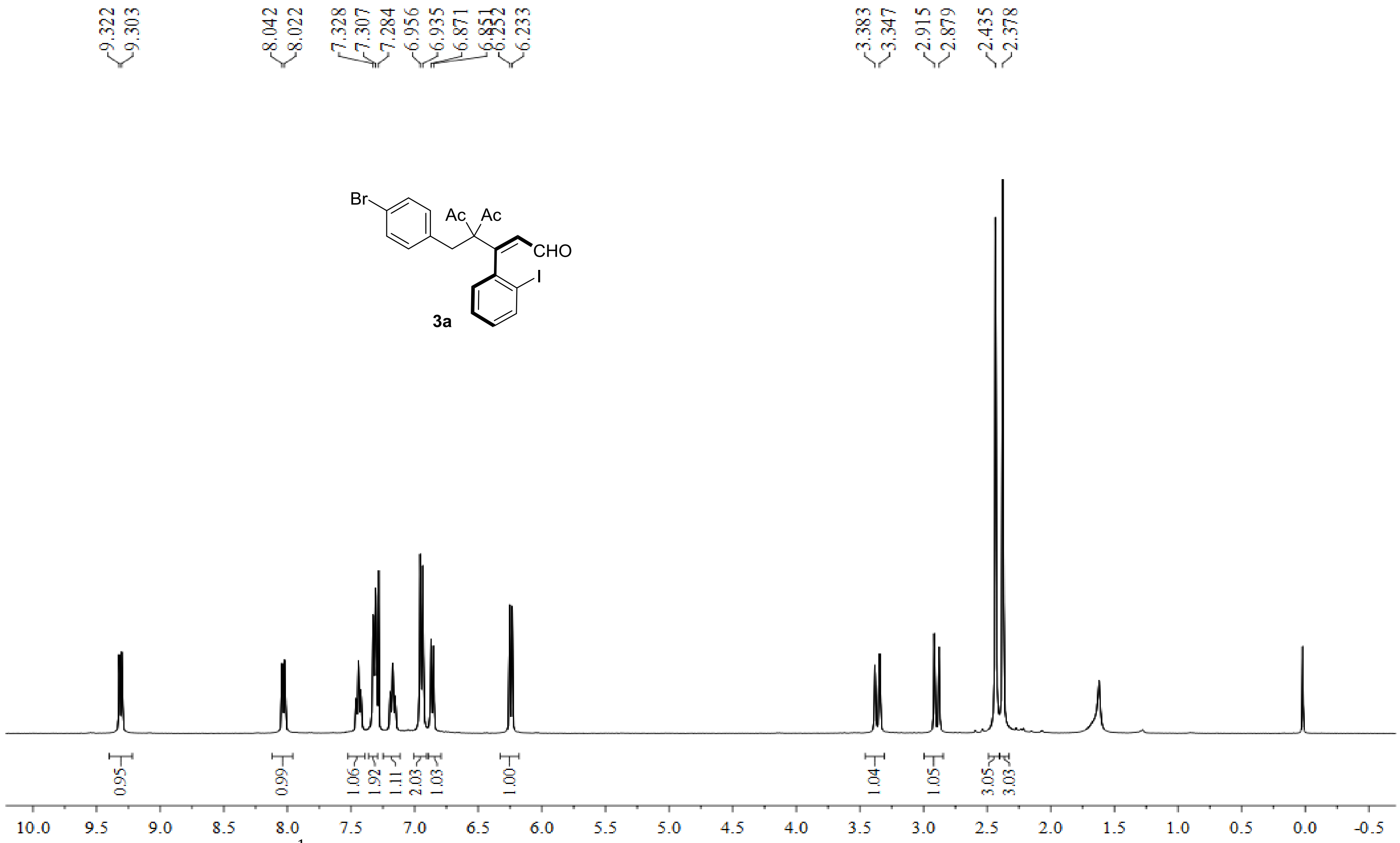

Supplementary Figure 19. ^{1}H NMR of **3a**

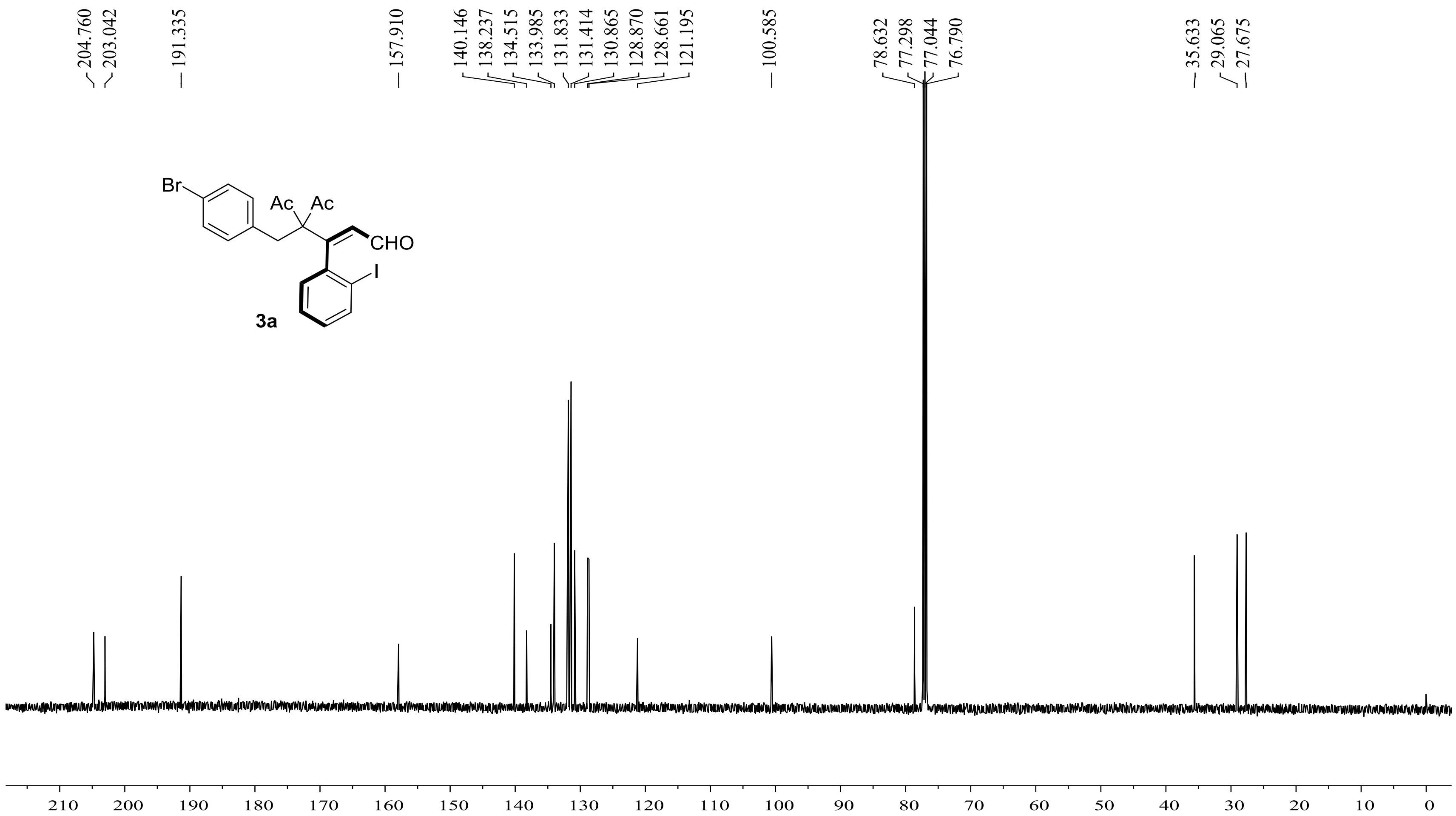

Supplementary Figure 20. ^{13}C NMR of **3a**

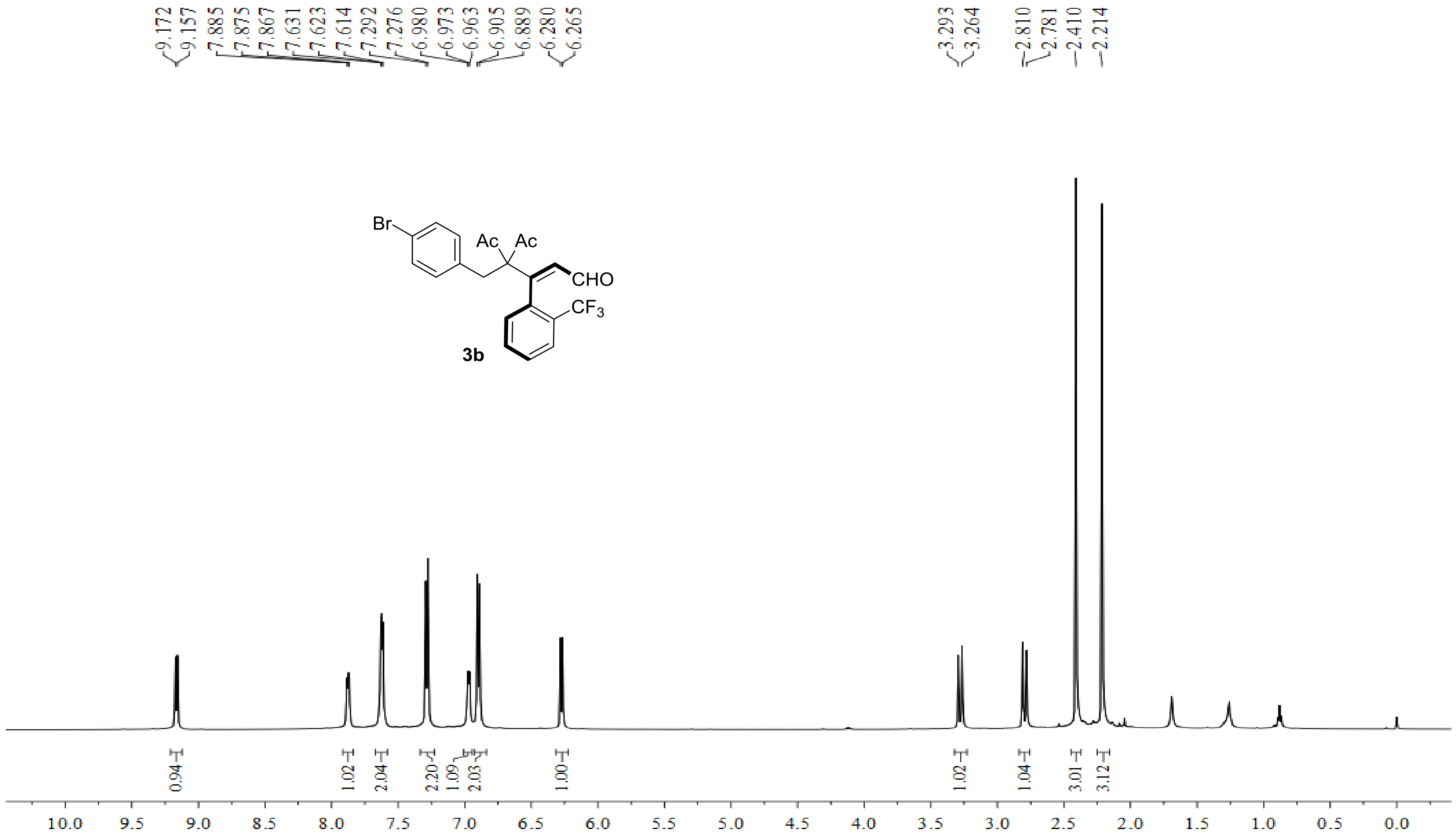

Supplementary Figure 21. ^{1}H NMR of **3b**

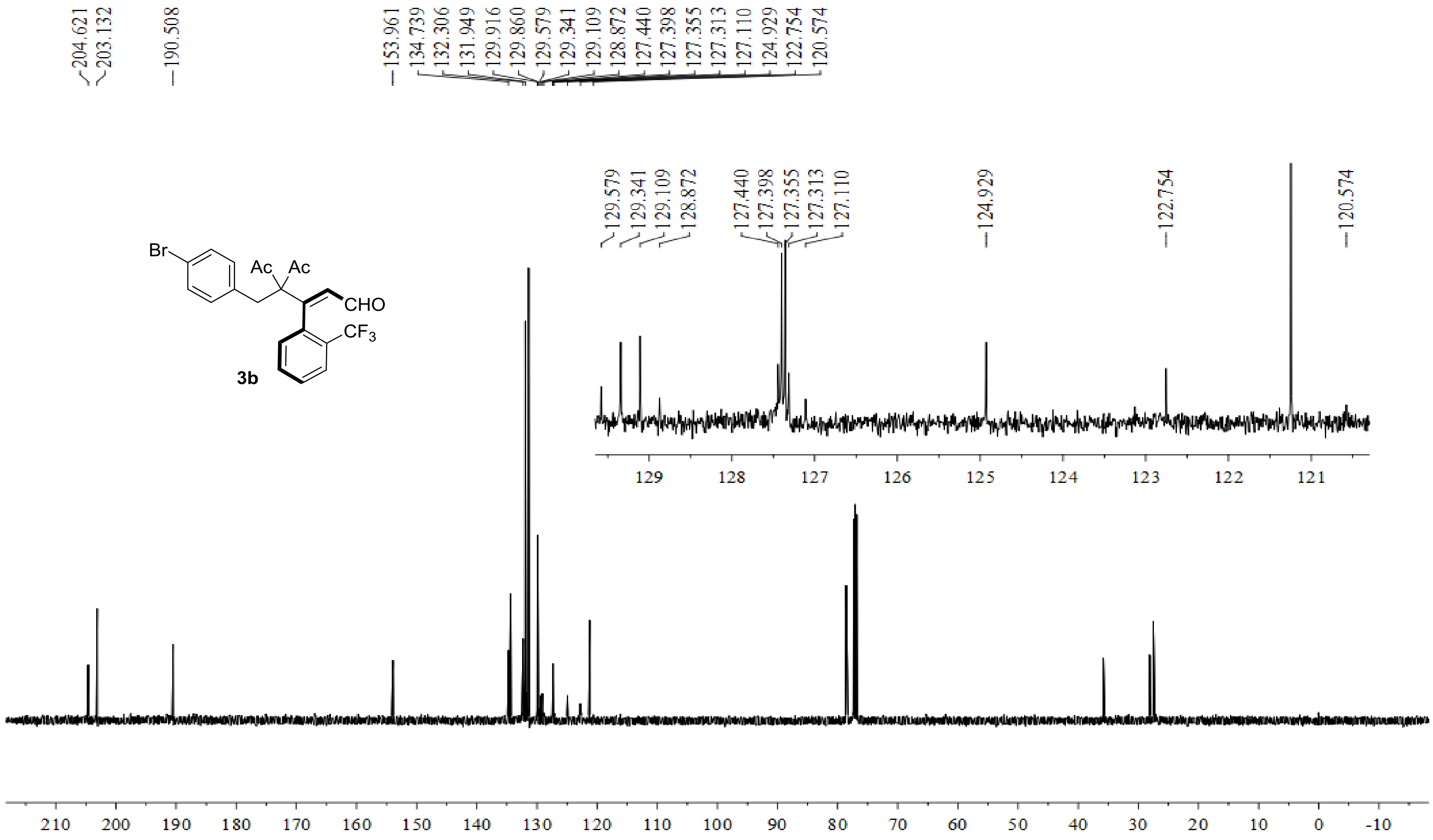

Supplementary Figure 22. ^{13}C NMR of **3b**

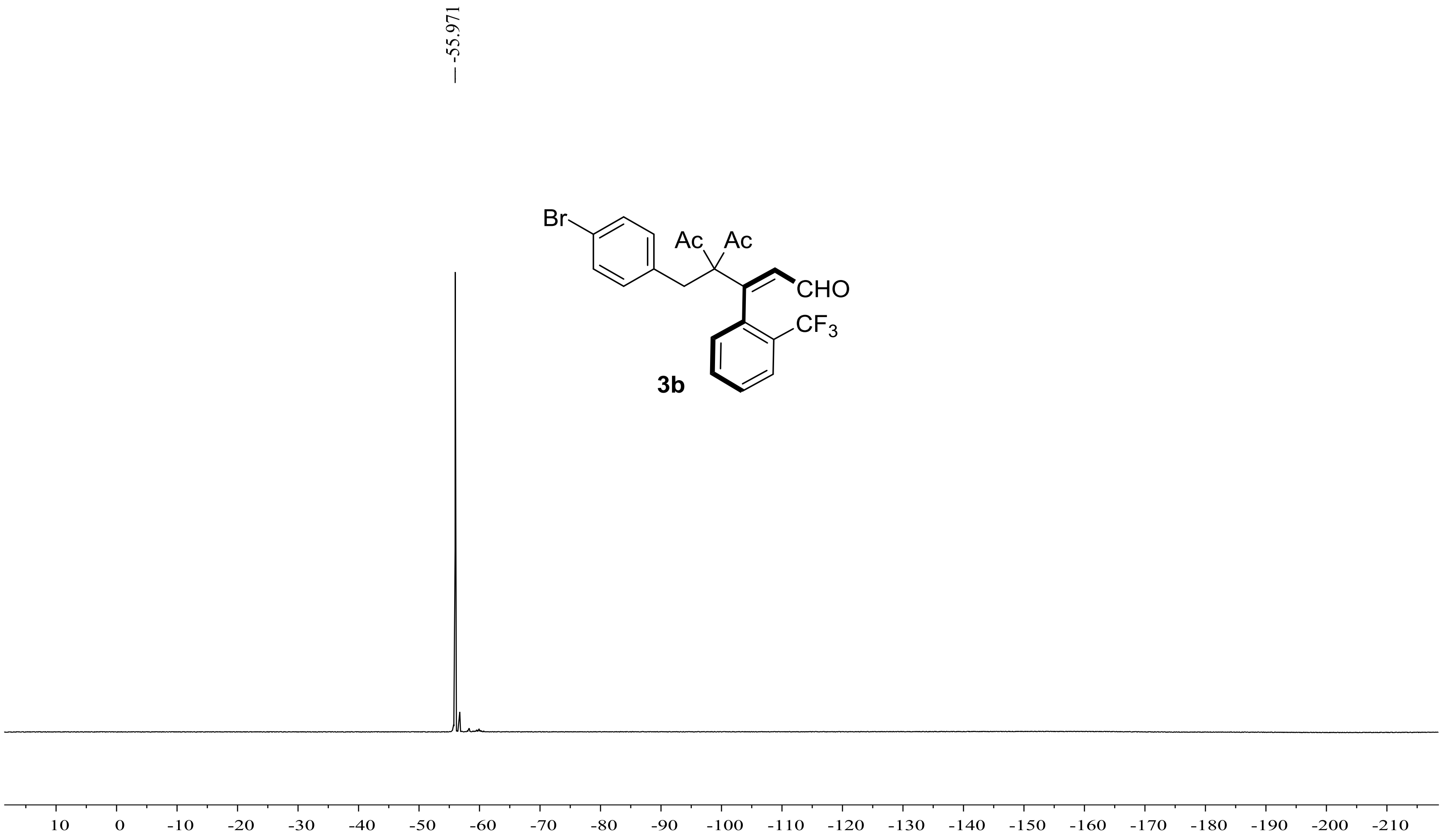

Supplementary Figure 23. ^{19}F NMR of **3b**

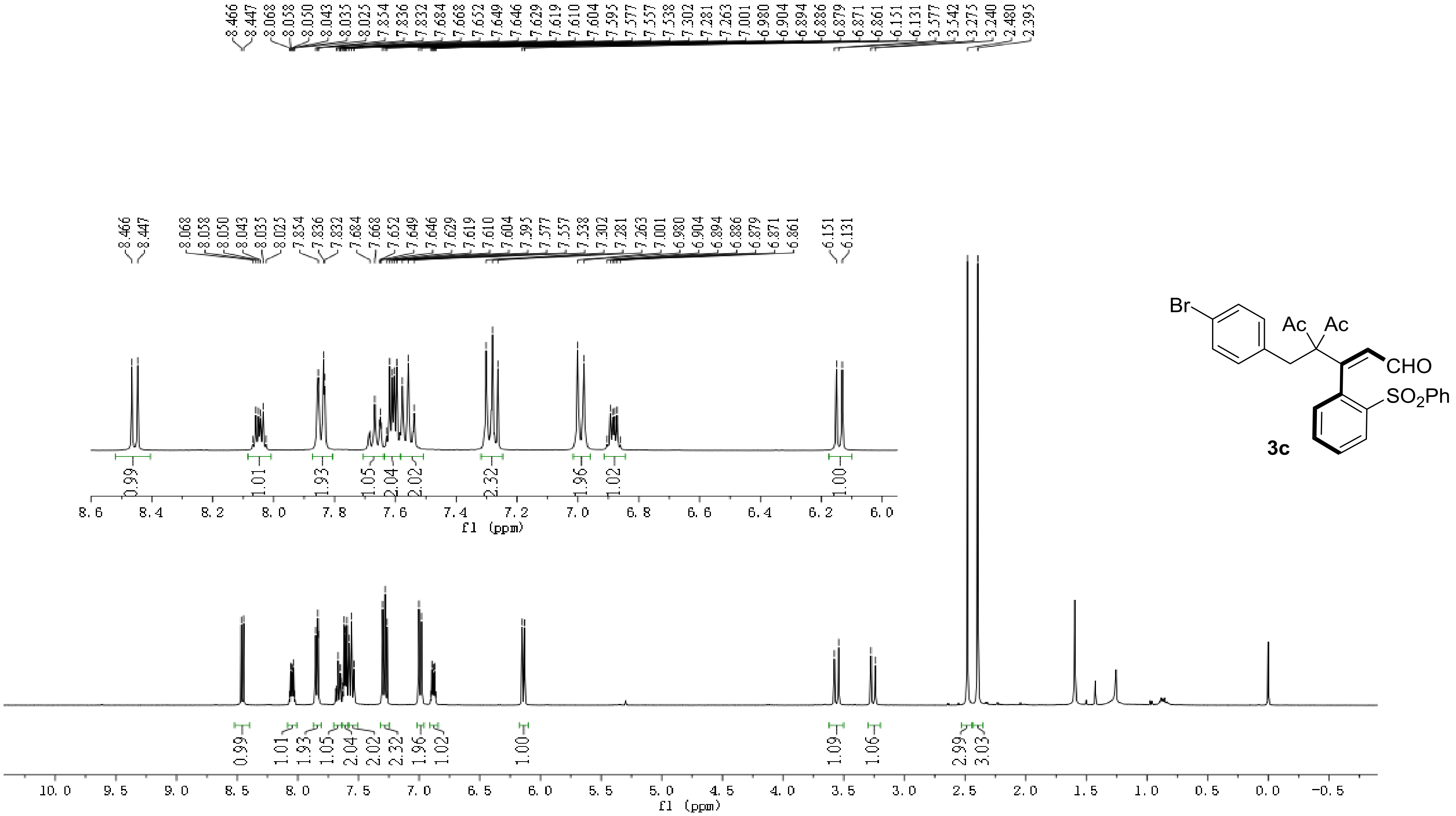

Supplementary Figure 24. ^{1}H NMR of **3c**

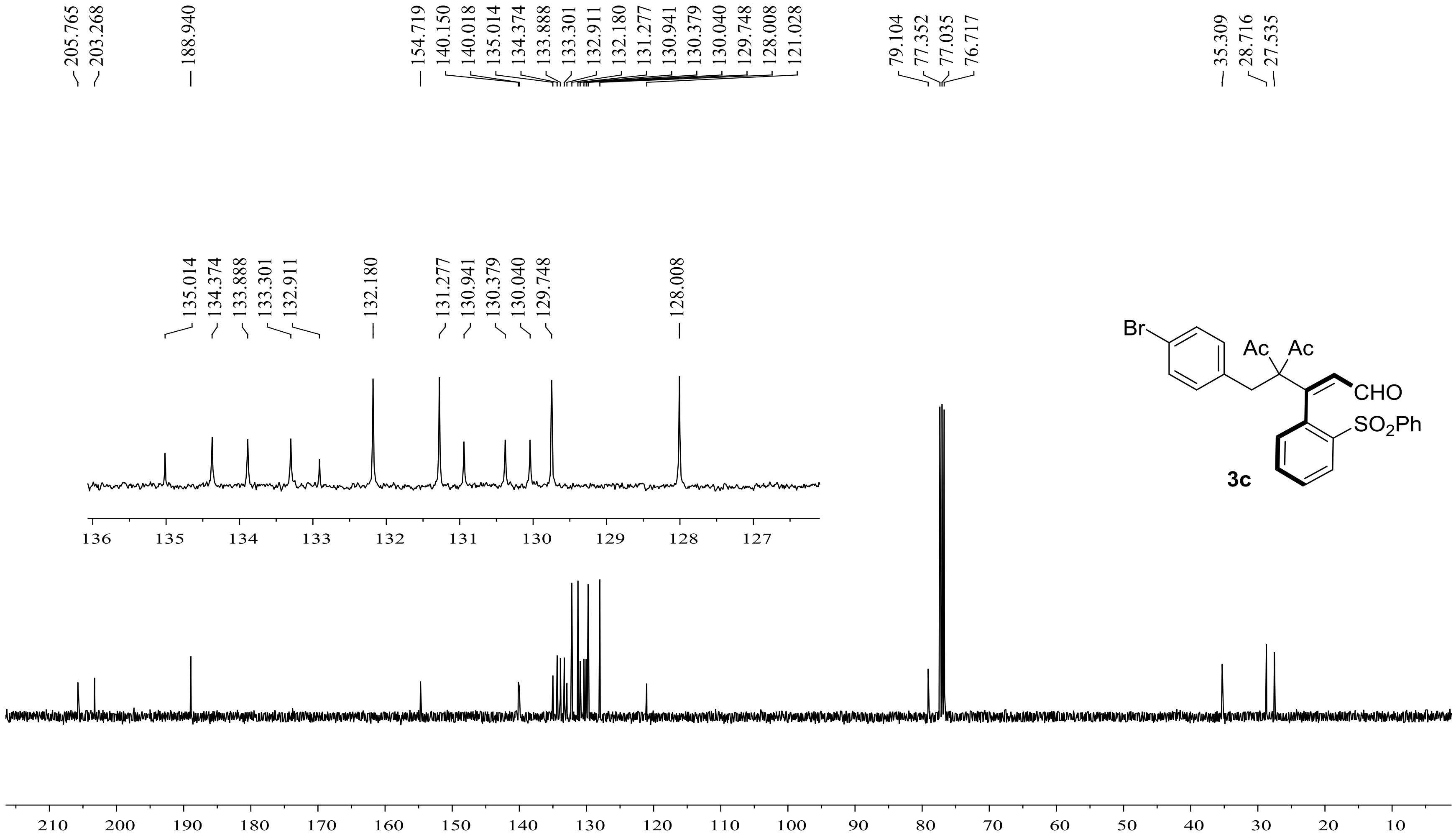

Supplementary Figure 25. ^{13}C NMR of **3c**

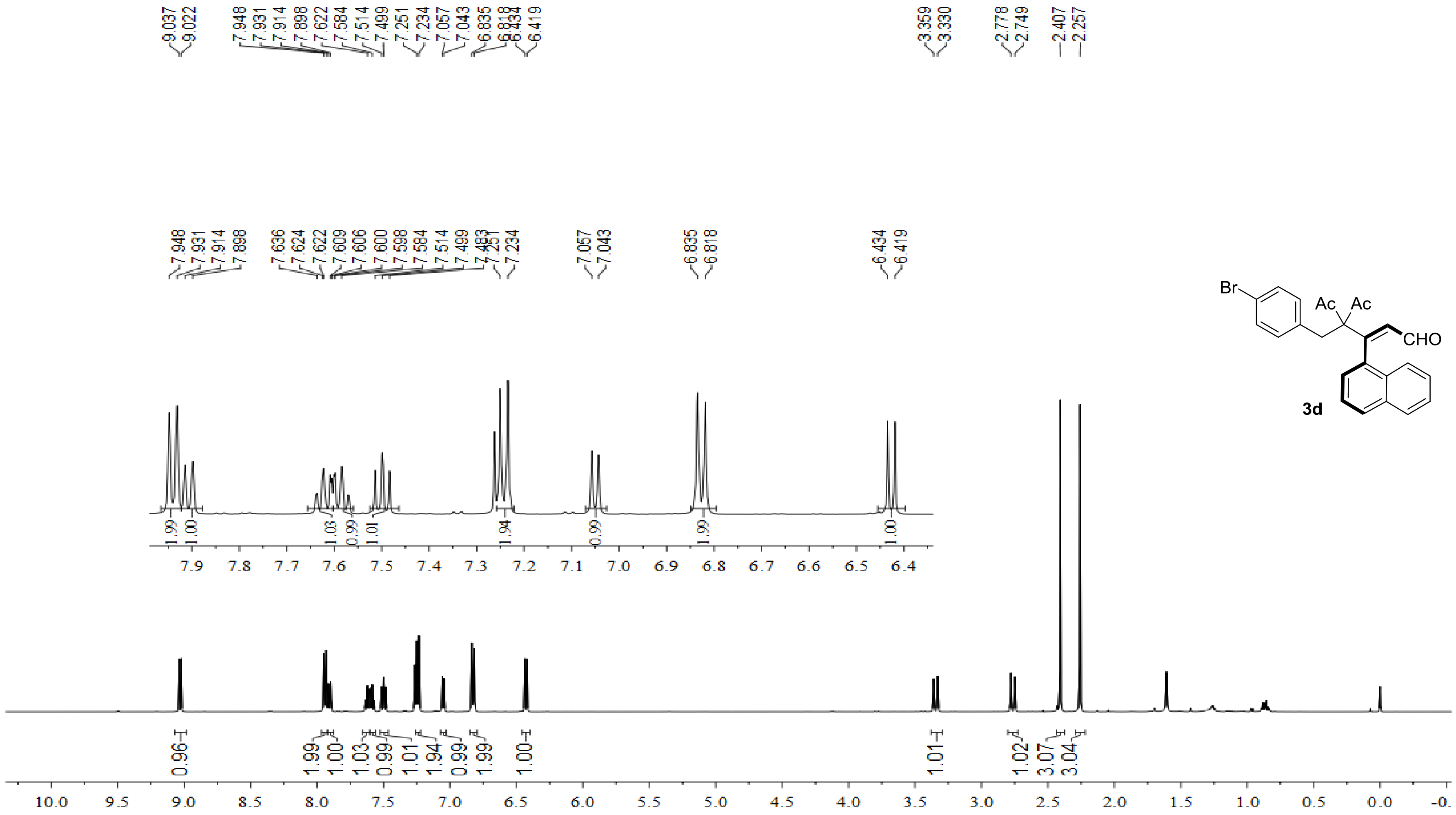

Supplementary Figure 26. ^{1}H NMR of **3d**

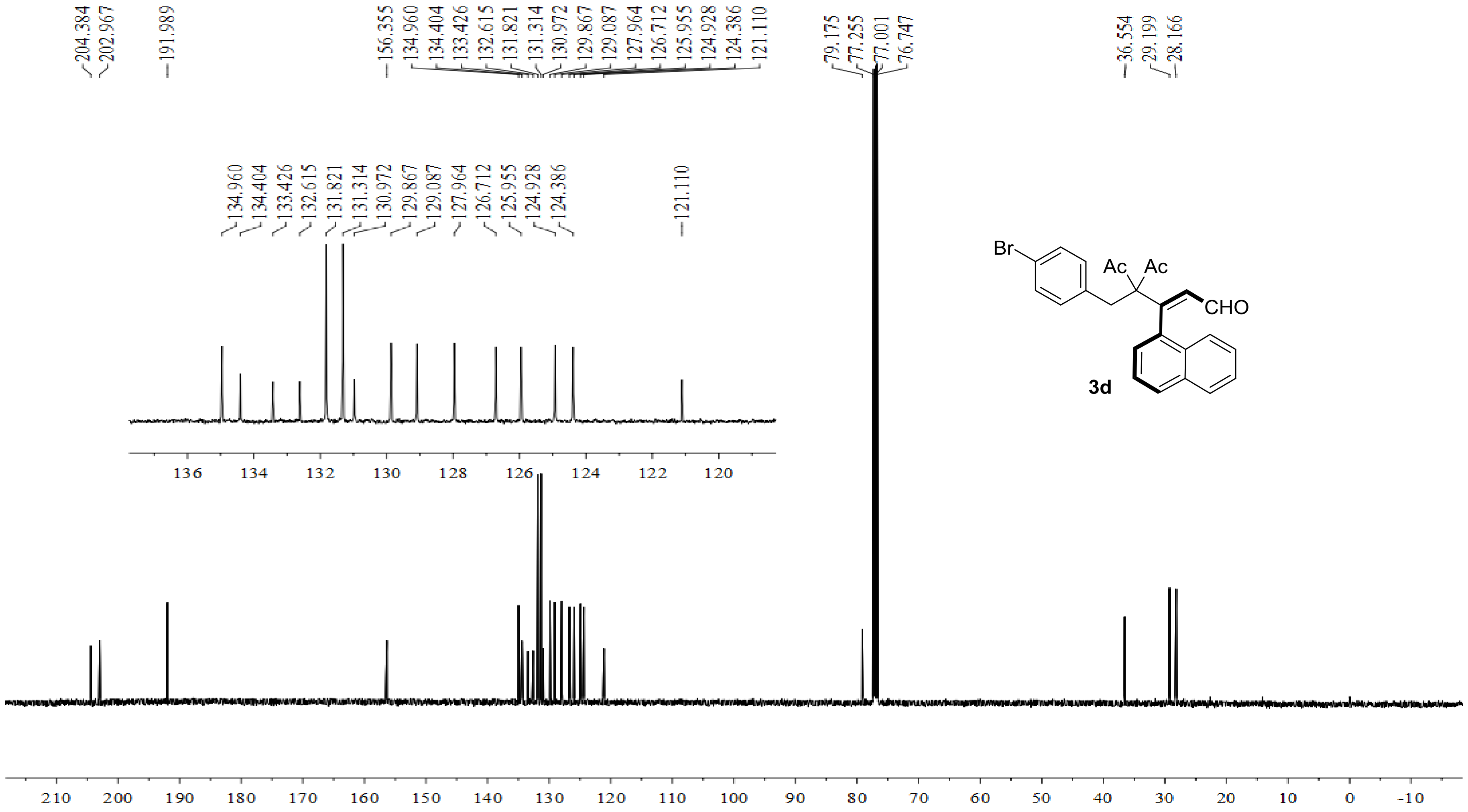

Supplementary Figure 27. ^{13}C NMR of **3d**

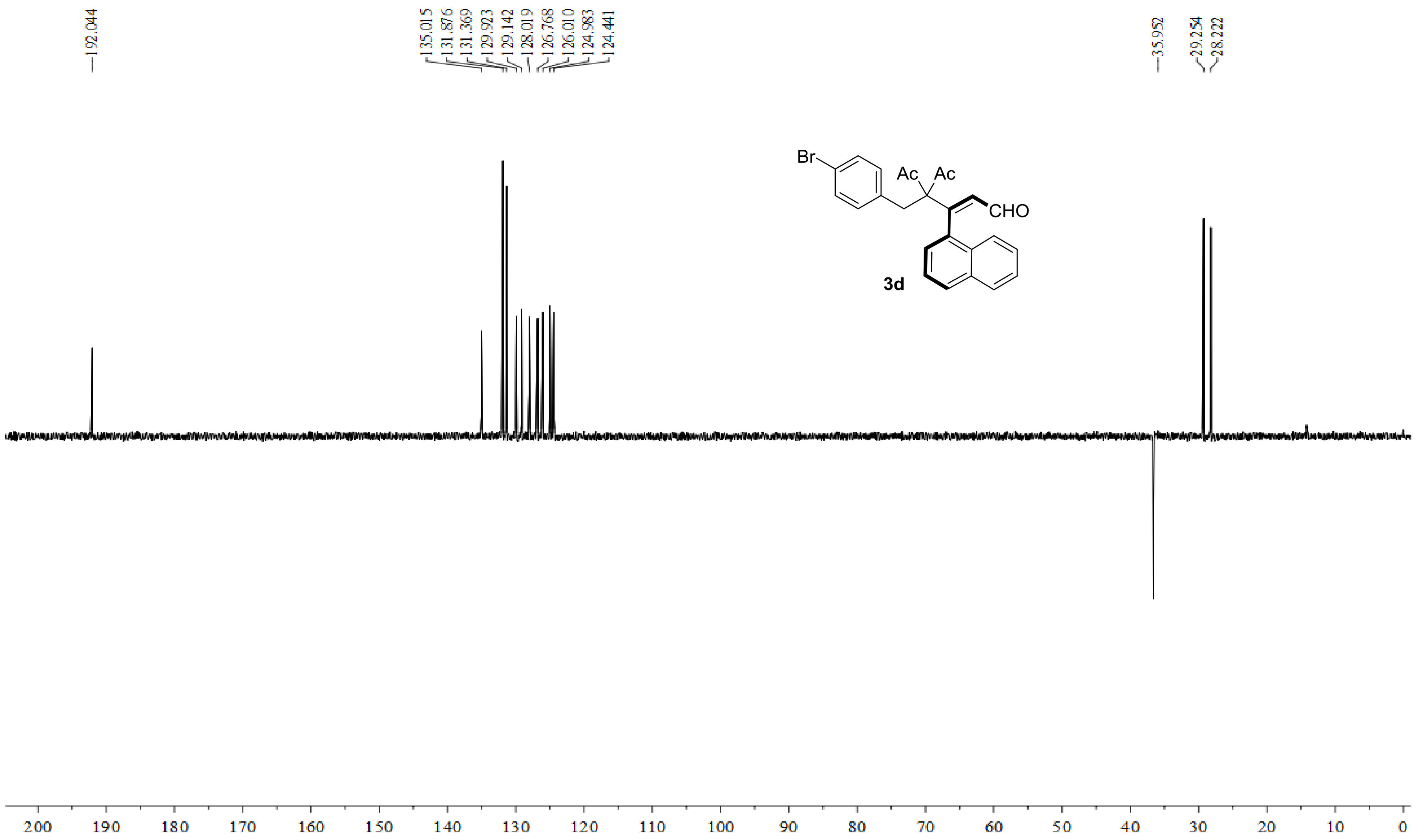

Supplementary Figure 28. DEPT-135 of **3d**

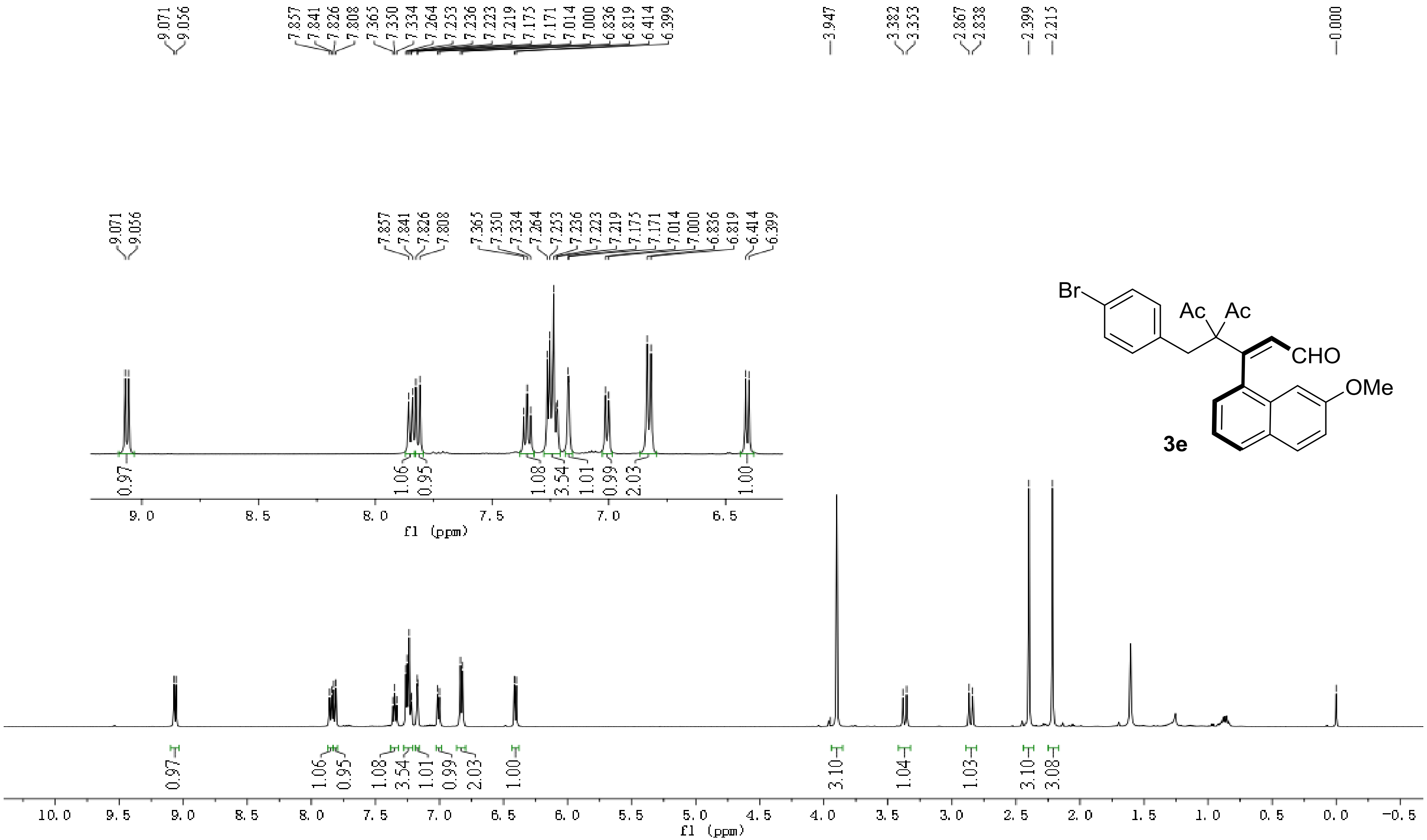

Supplementary Figure 29. ^{1}H NMR of **3e**

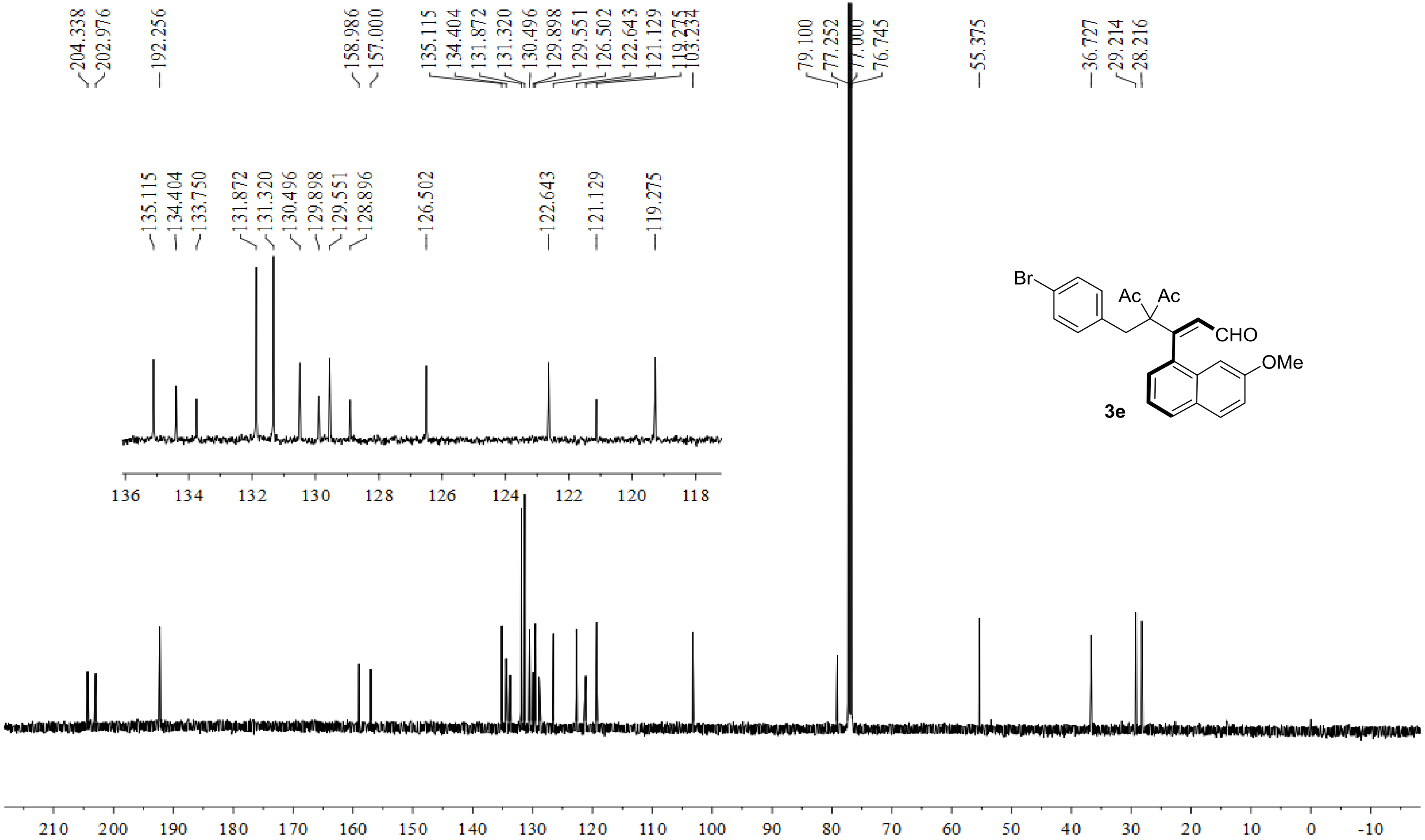

Supplementary Figure 30. ^{13}C NMR of **3e**

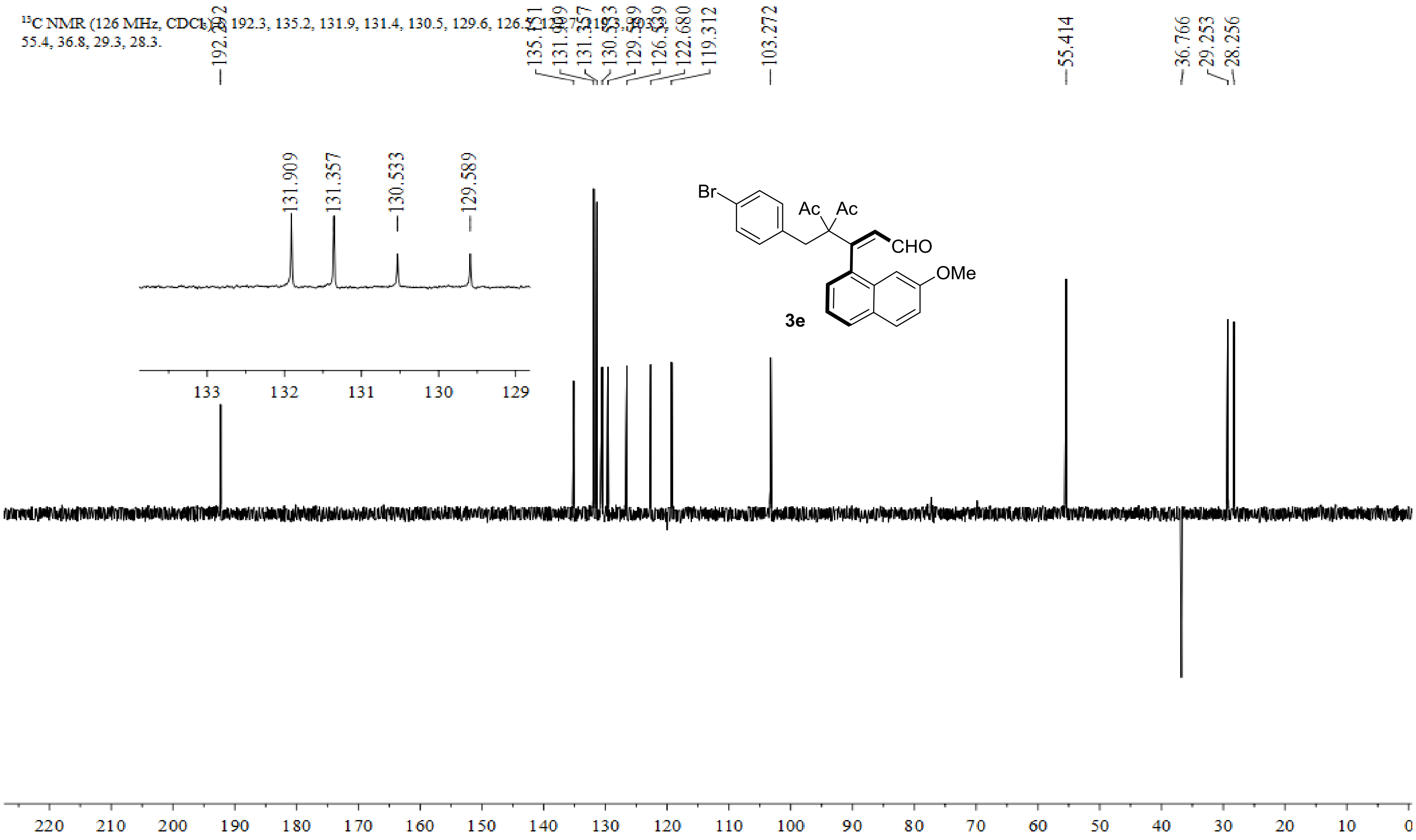

Supplementary Figure 31. DEPT-135 of **3e**

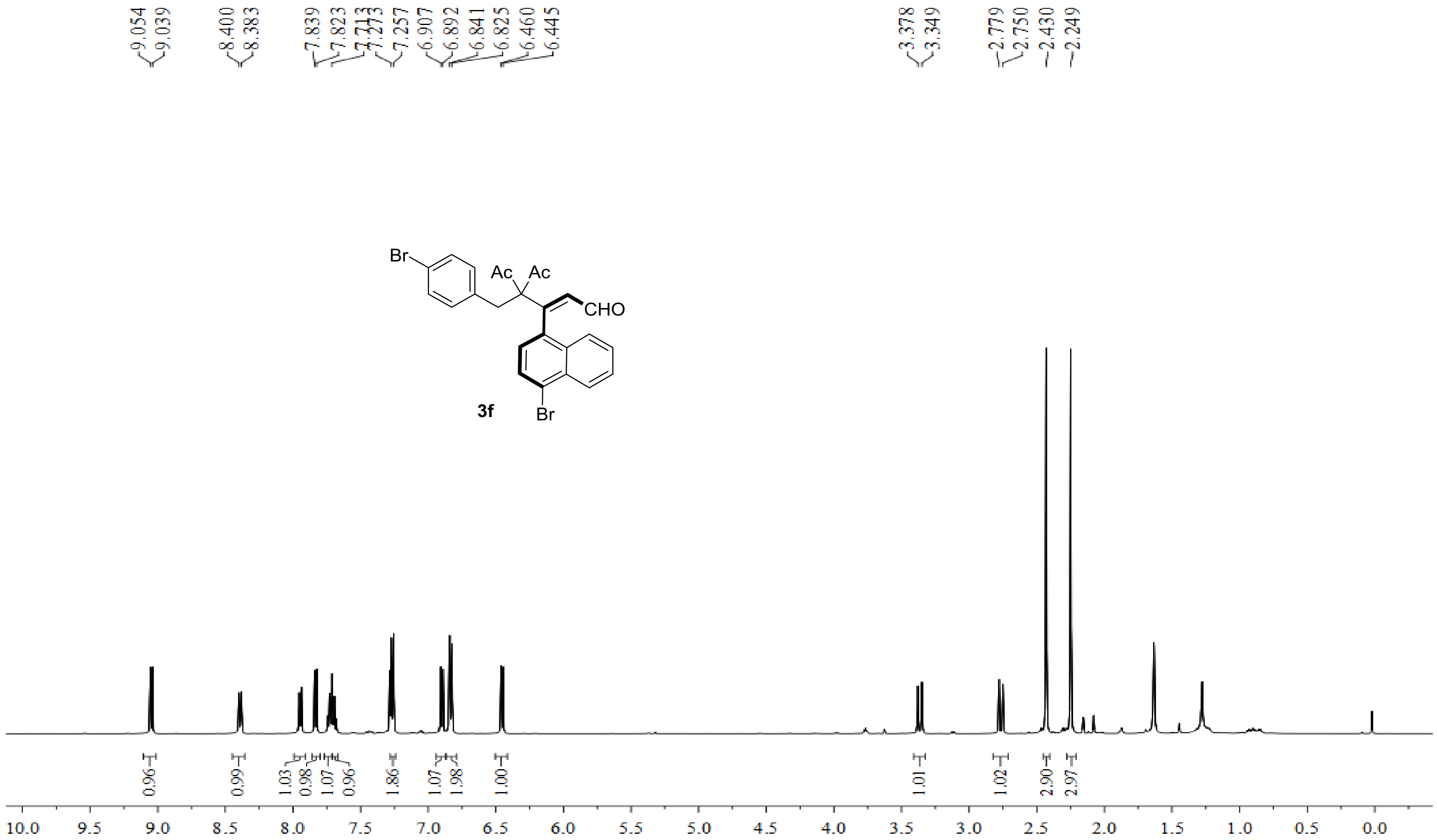

Supplementary Figure 32. ^{1}H NMR of **3f**

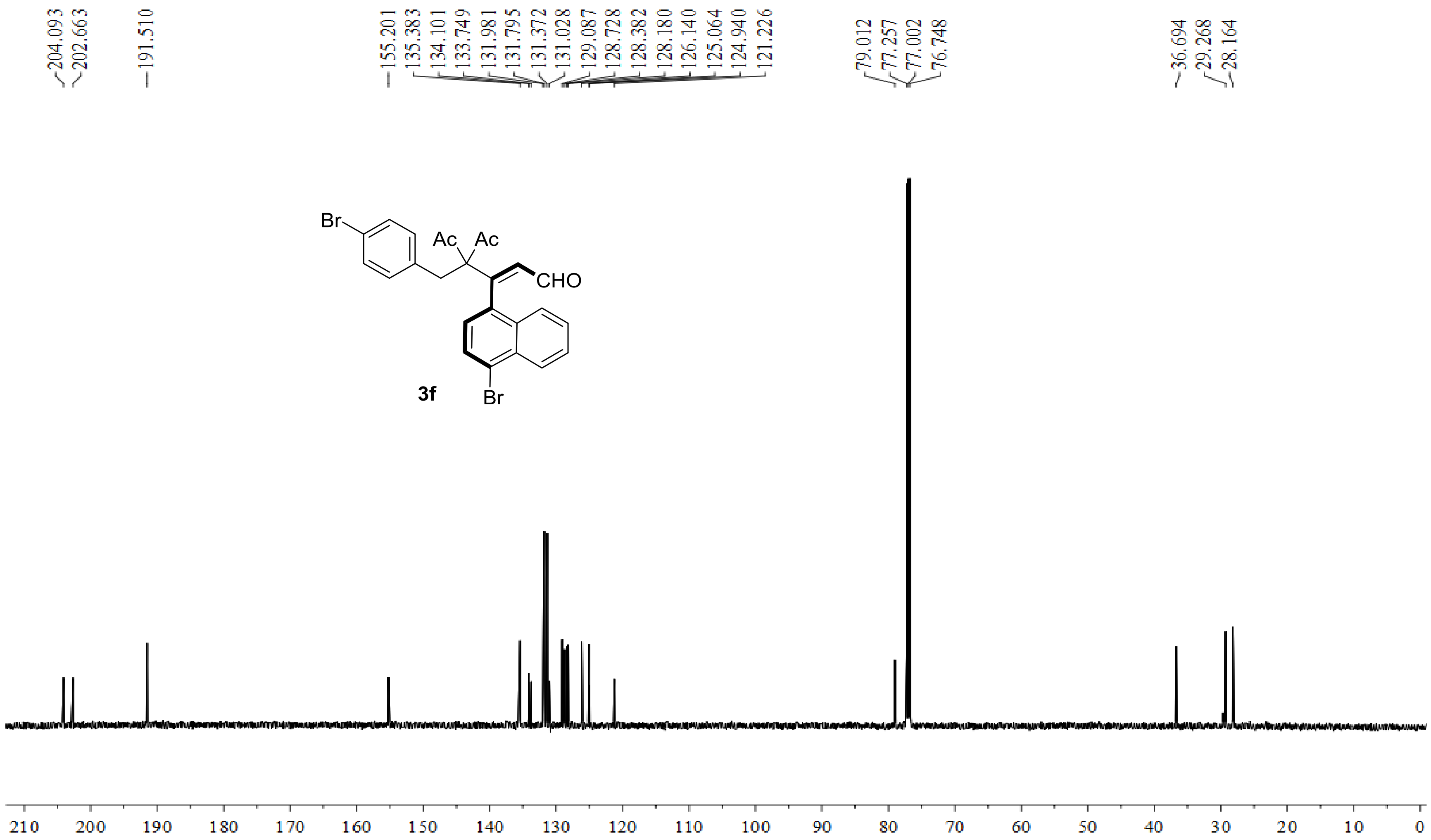

Supplementary Figure 33. ^{13}C NMR of **3f**

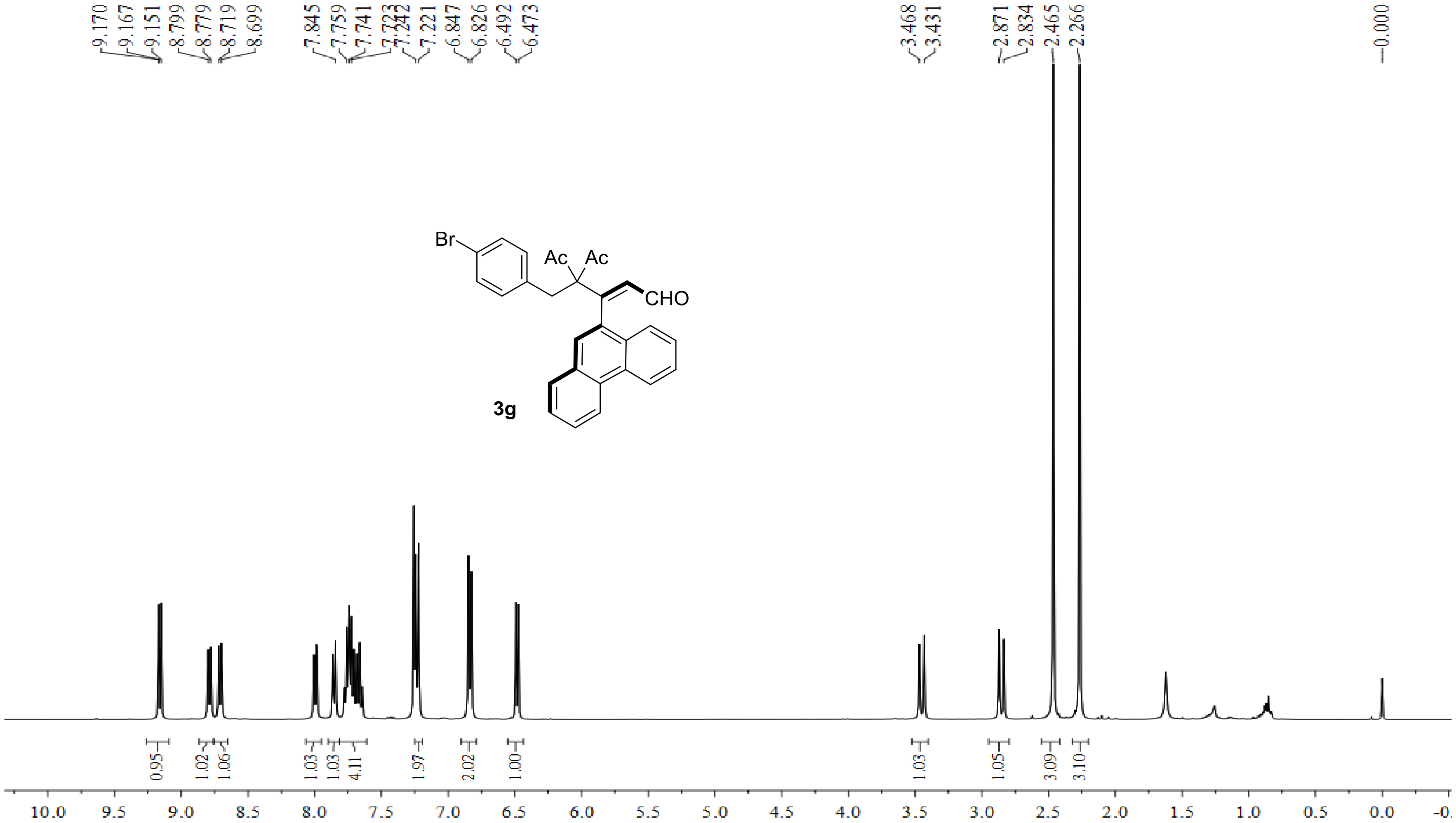

Supplementary Figure 34. ^{1}H NMR of **3g**

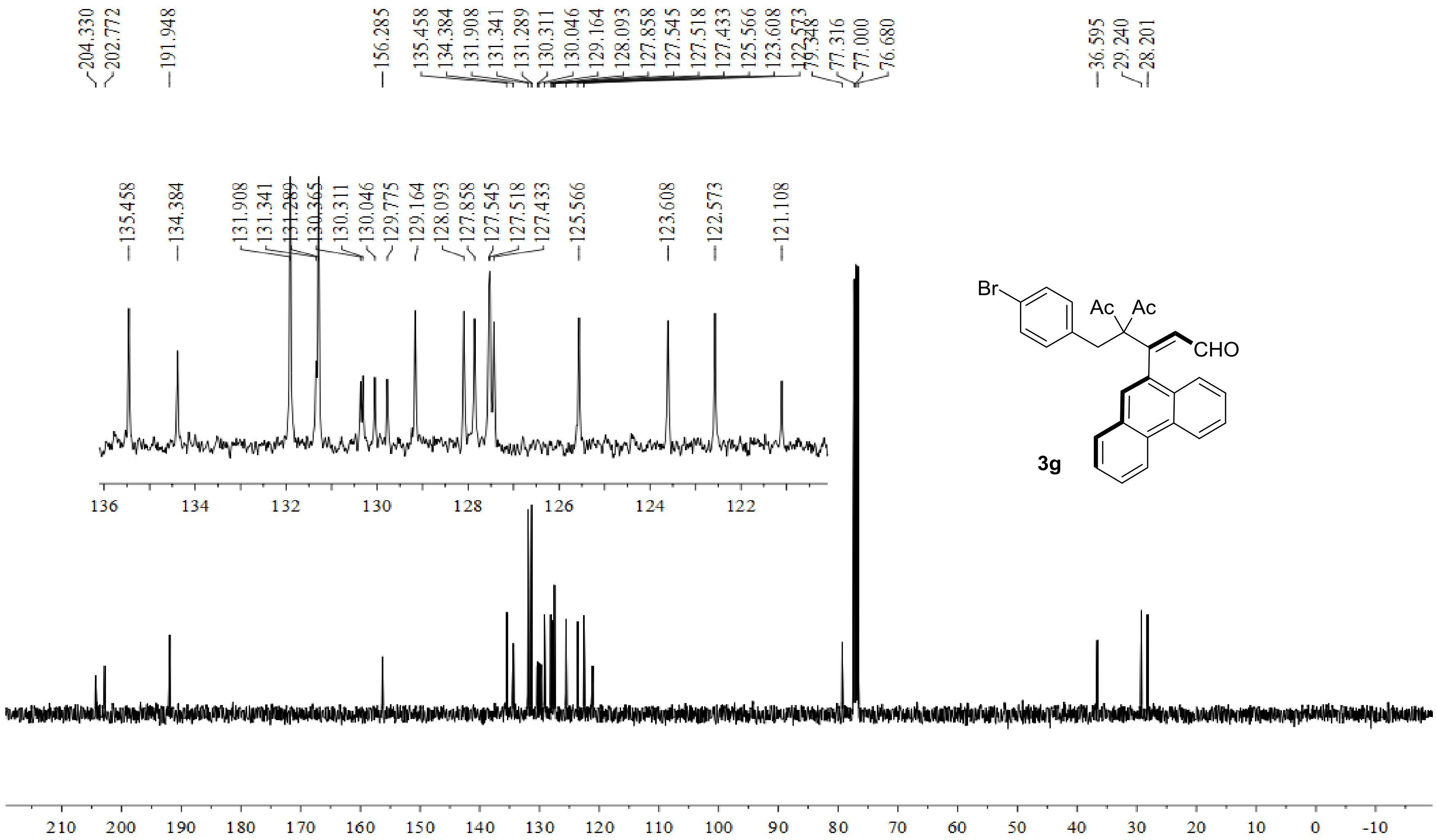

Supplementary Figure 35. ^{13}C NMR of **3g**

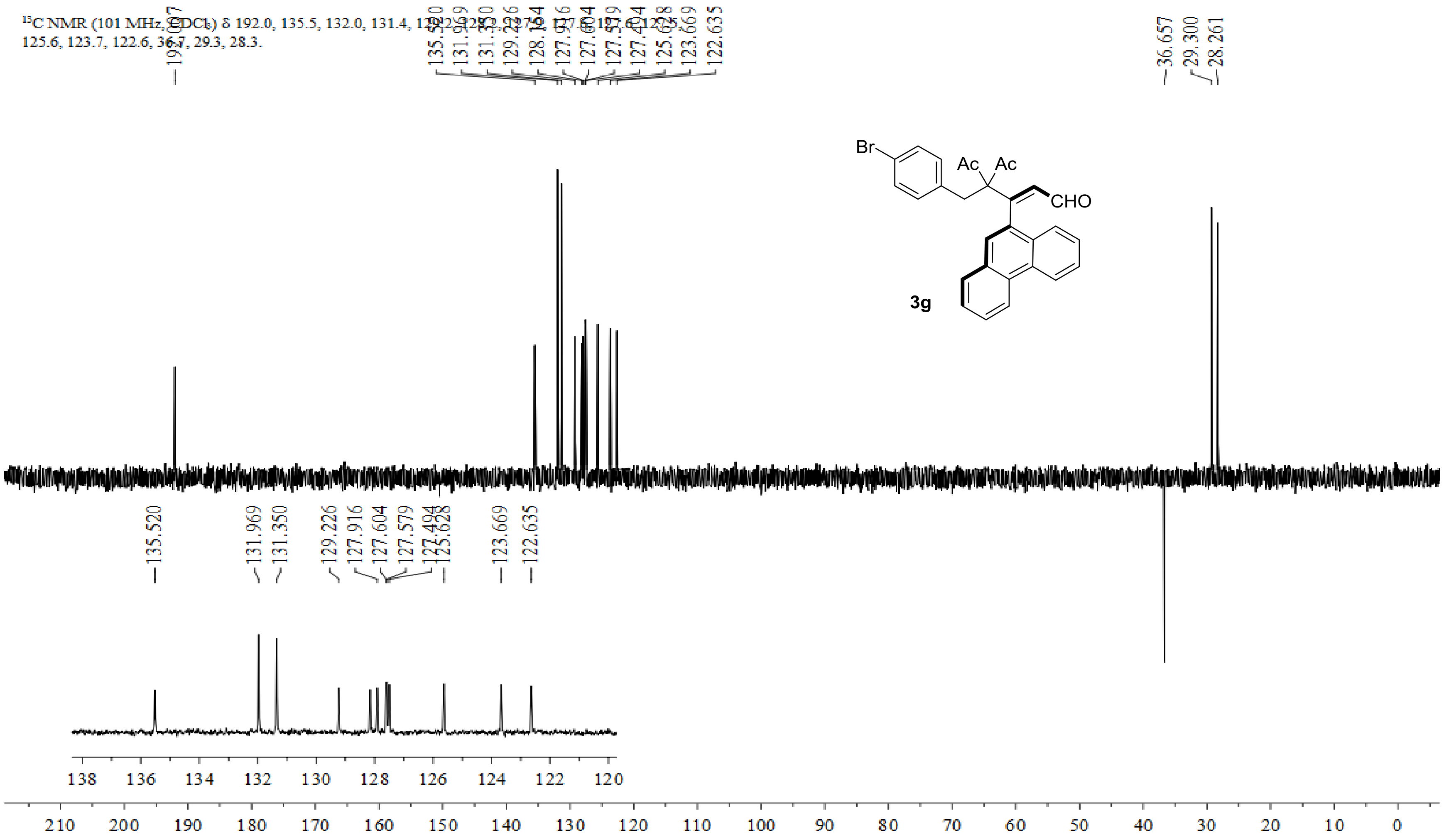

Supplementary Figure 36. DEPT-135 of **3g**

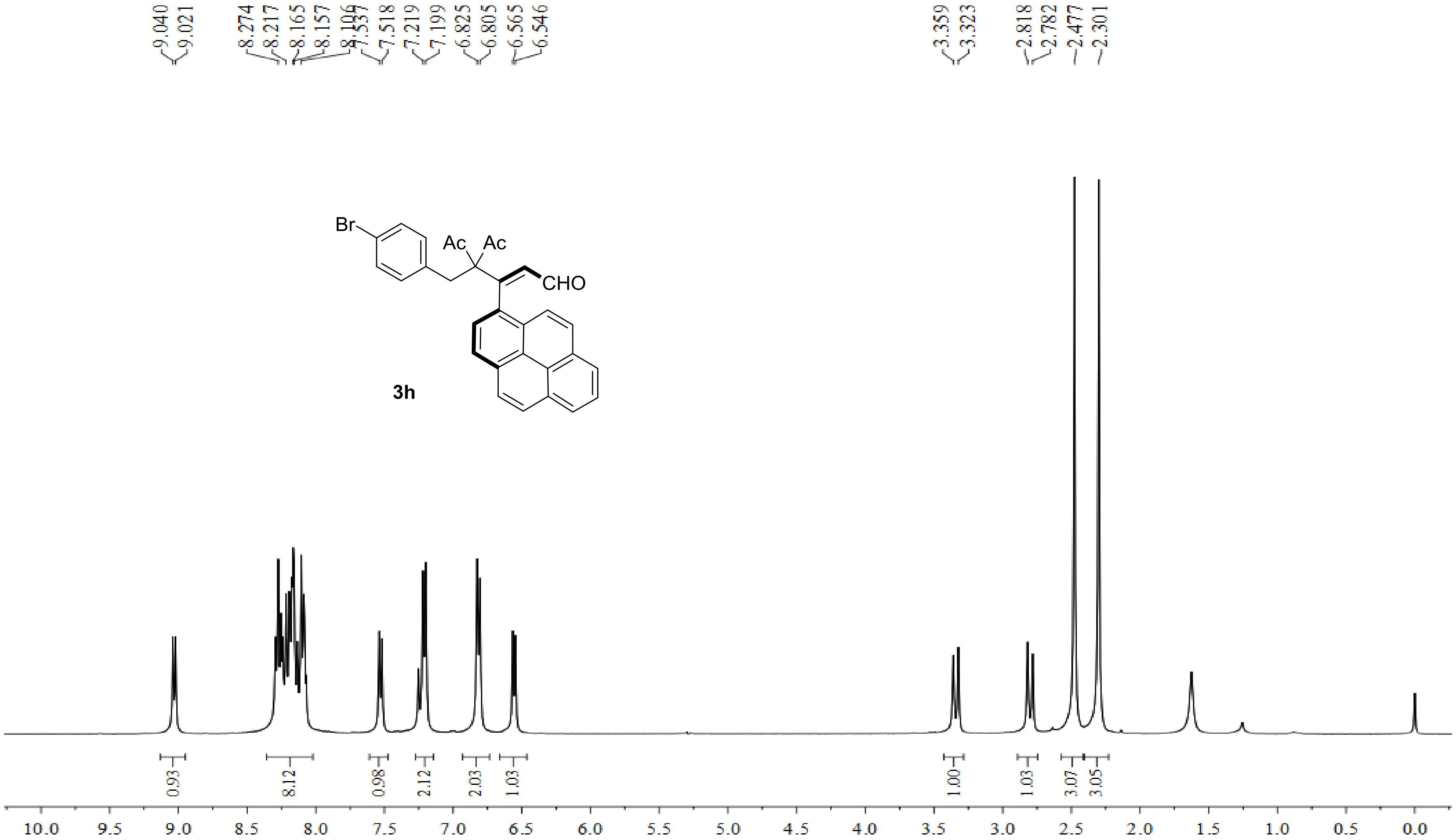

Supplementary Figure 37. ^{1}H NMR of **3h**

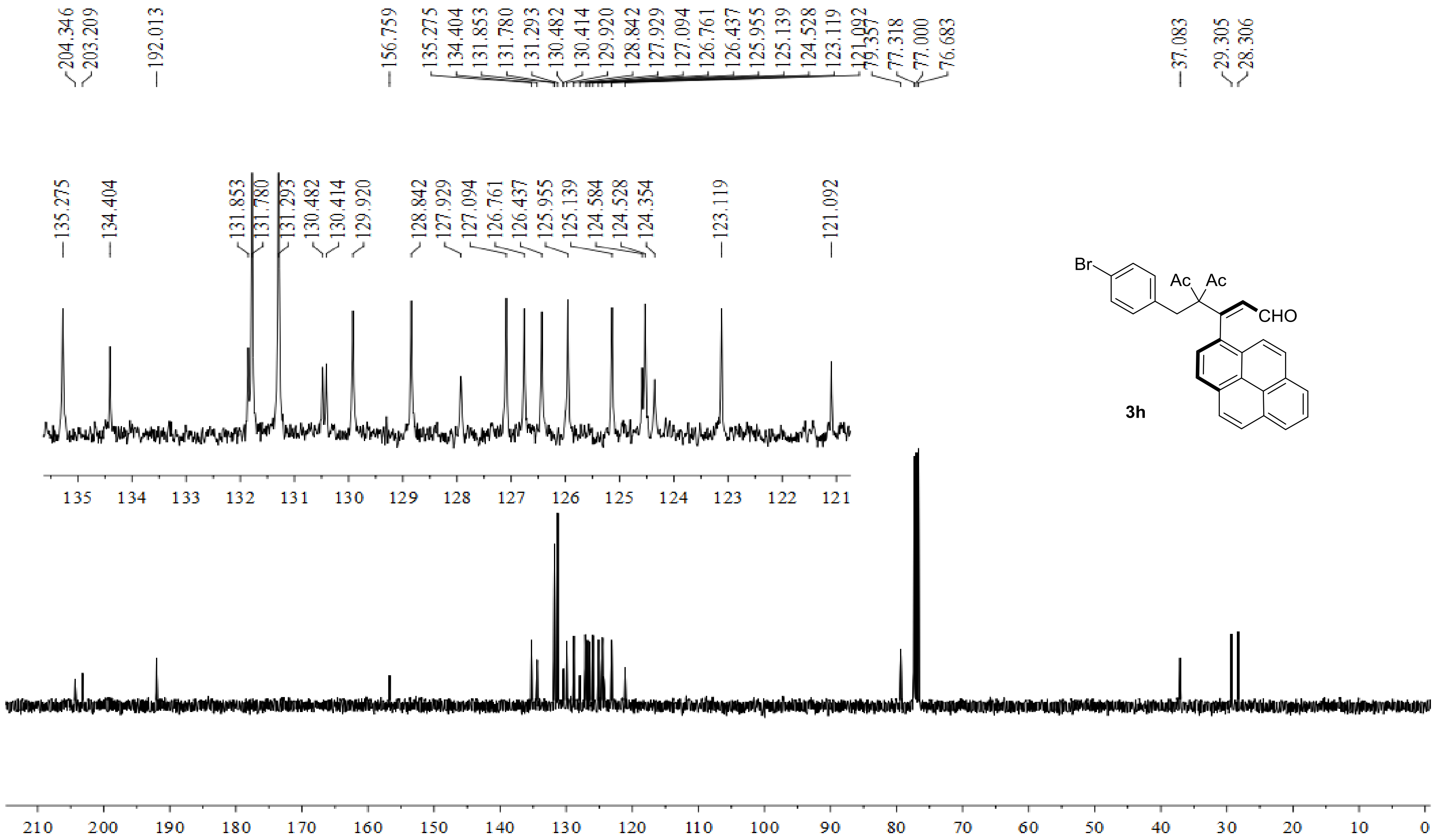

Supplementary Figure 38. ^{13}C NMR of **3h**

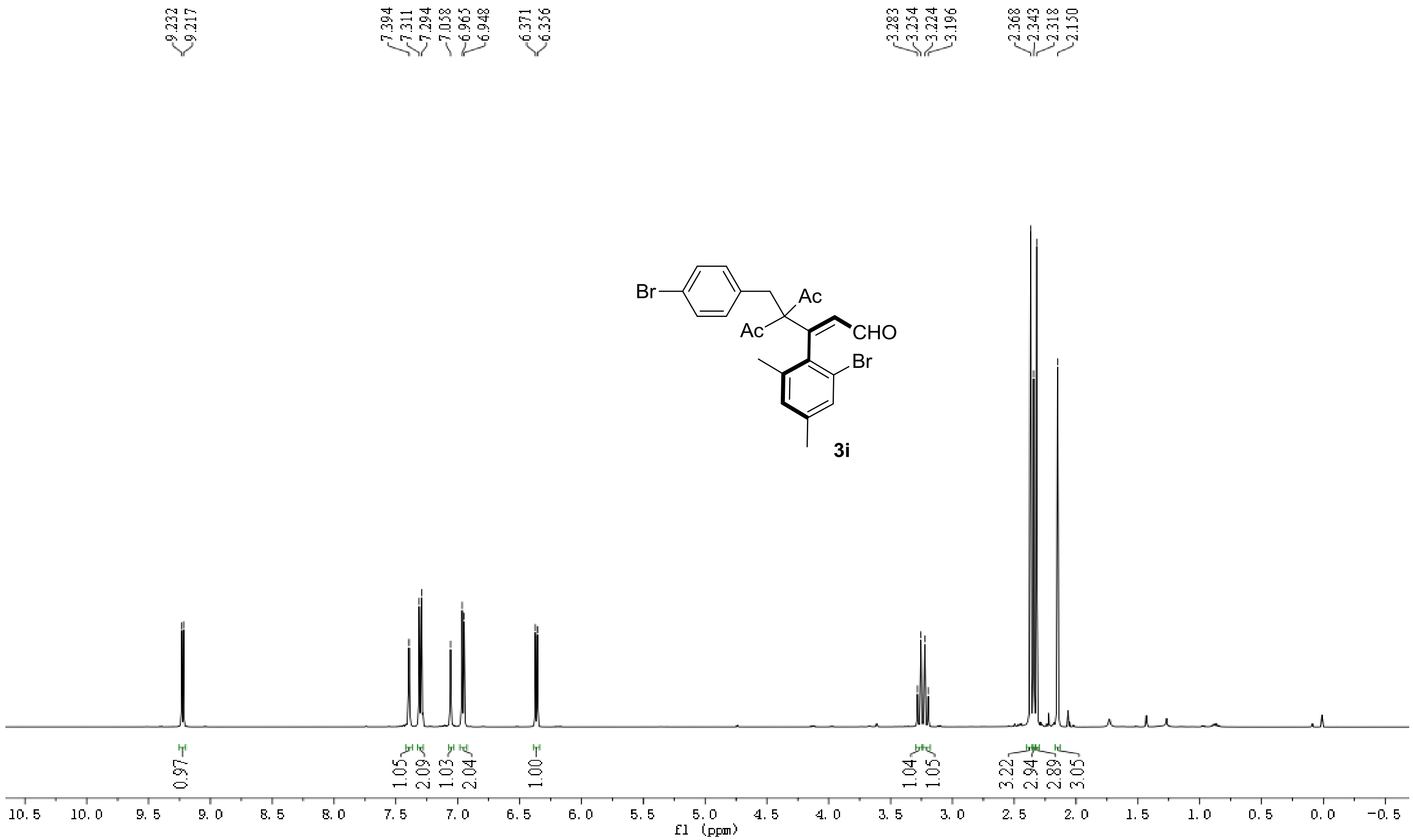

Supplementary Figure 39. ^{1}H NMR of **3i**

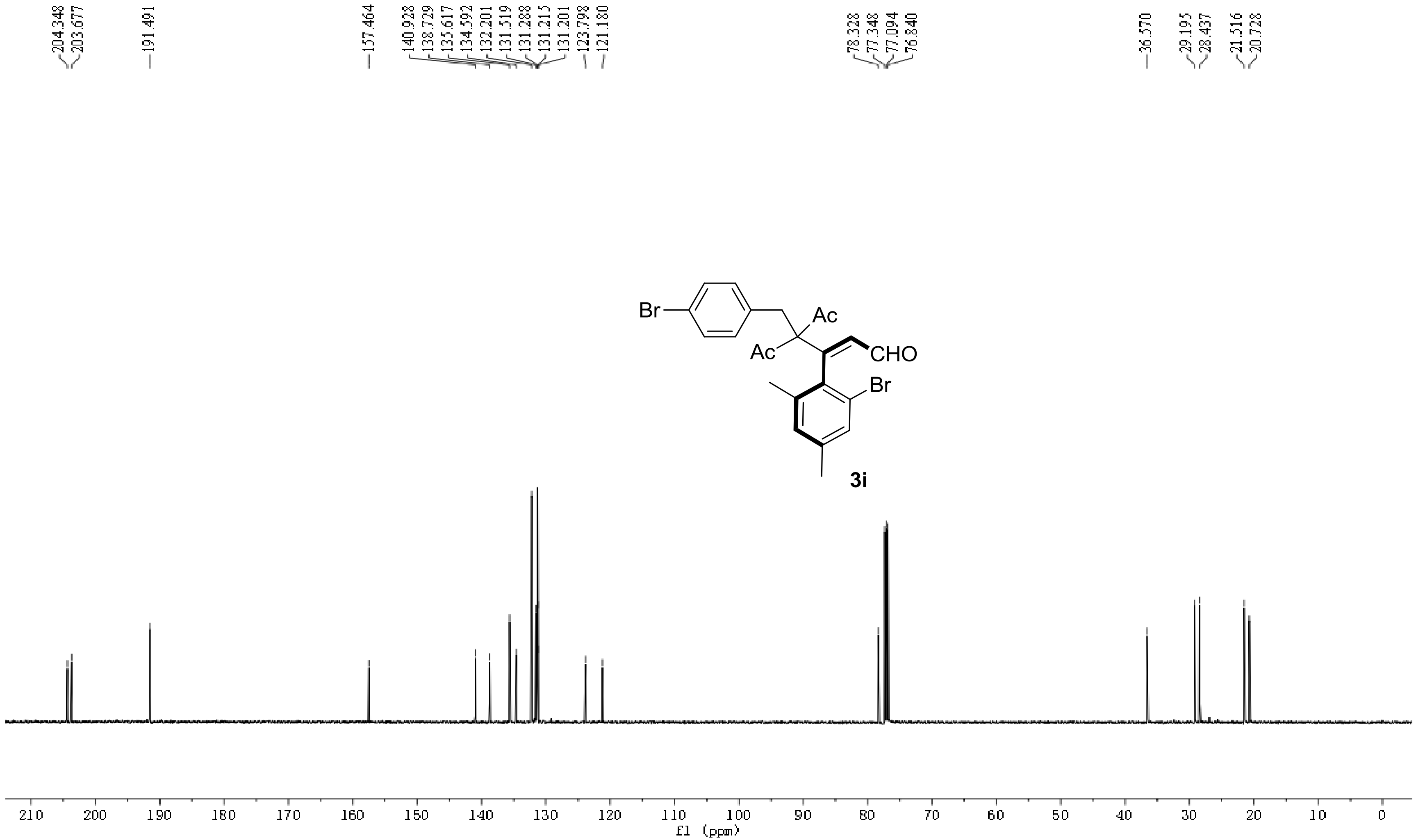

Supplementary Figure 40. ^{13}C NMR of **3i**

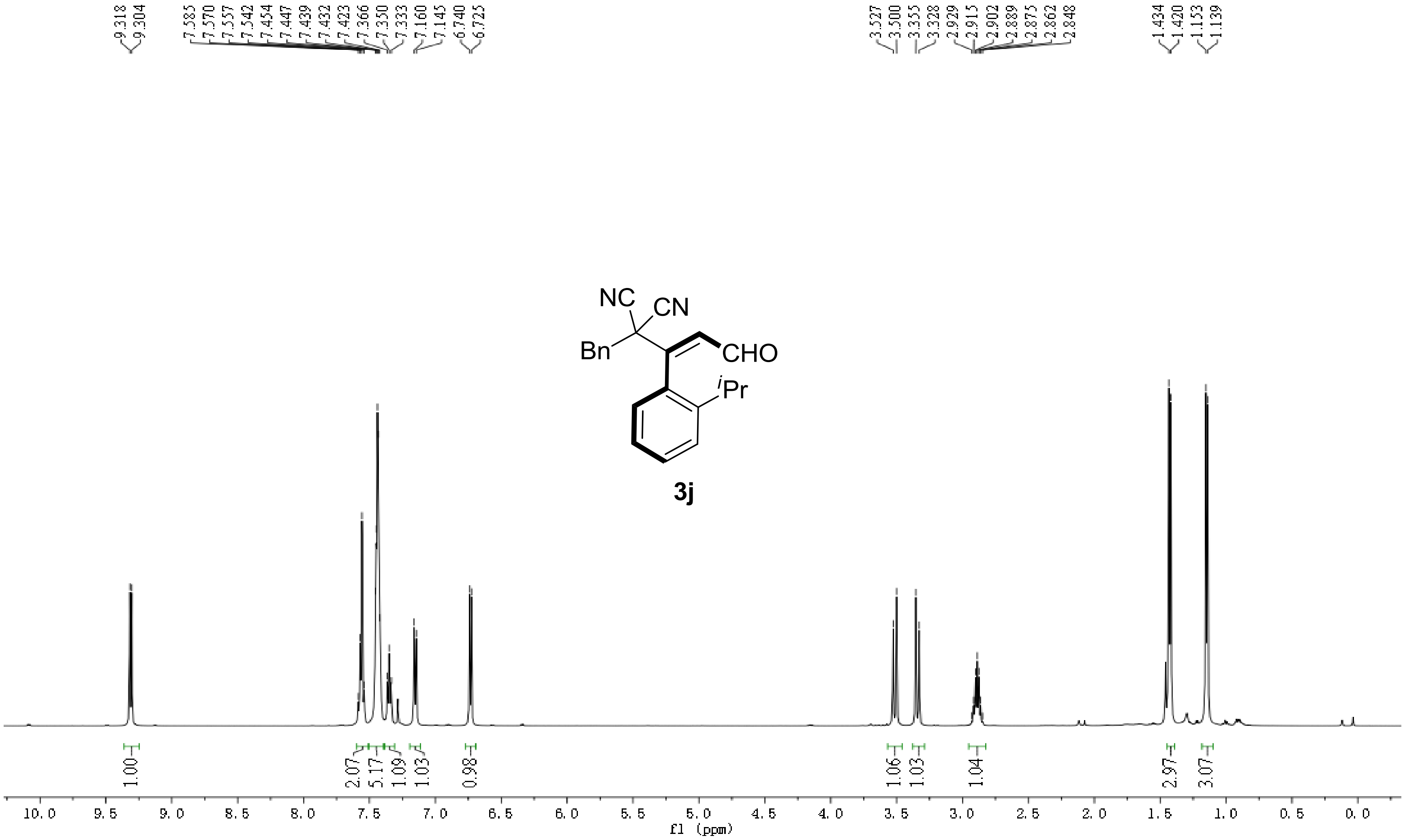

Supplementary Figure 41. ^{1}H NMR of **3j**

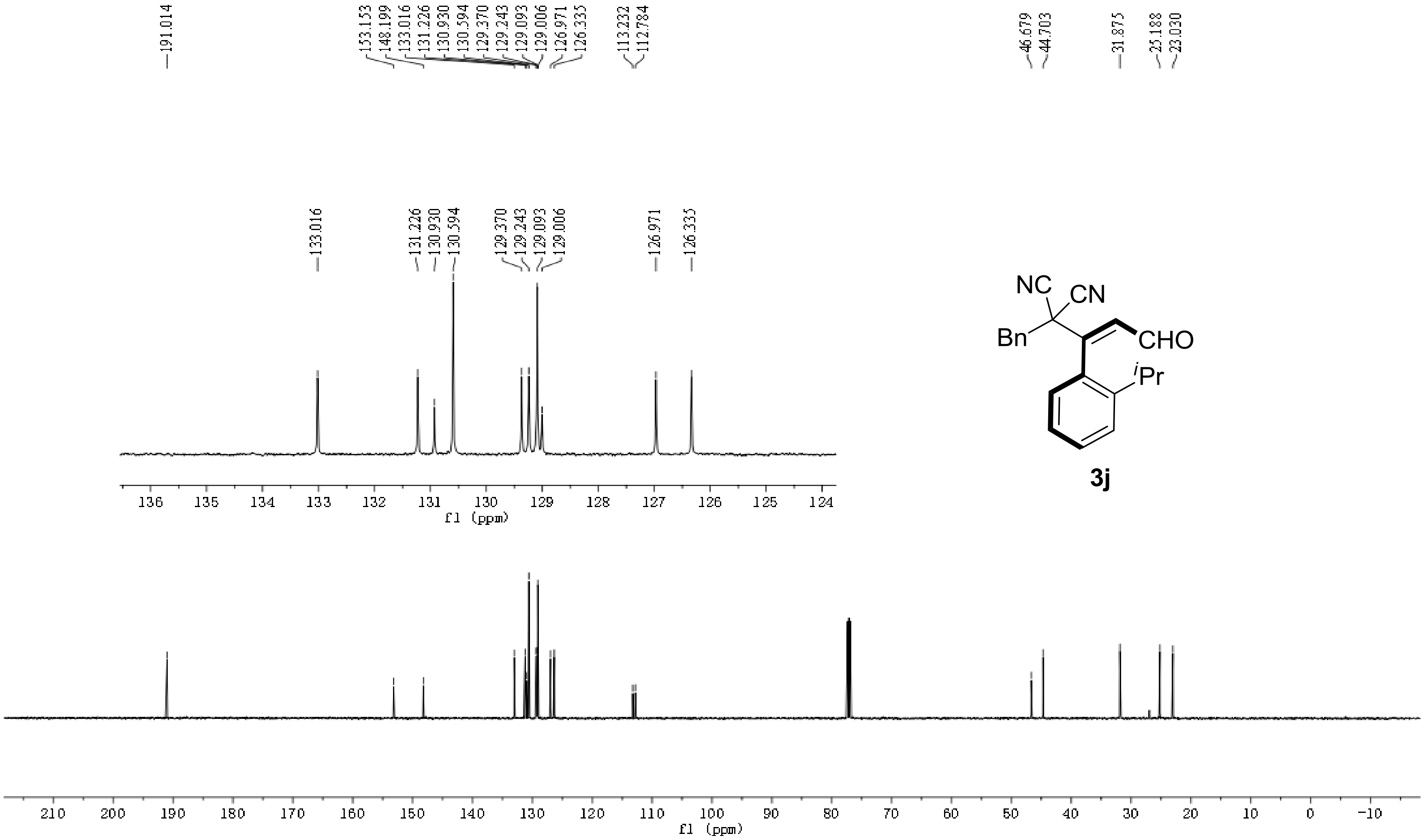

Supplementary Figure 42. ^{13}C NMR of **3j**

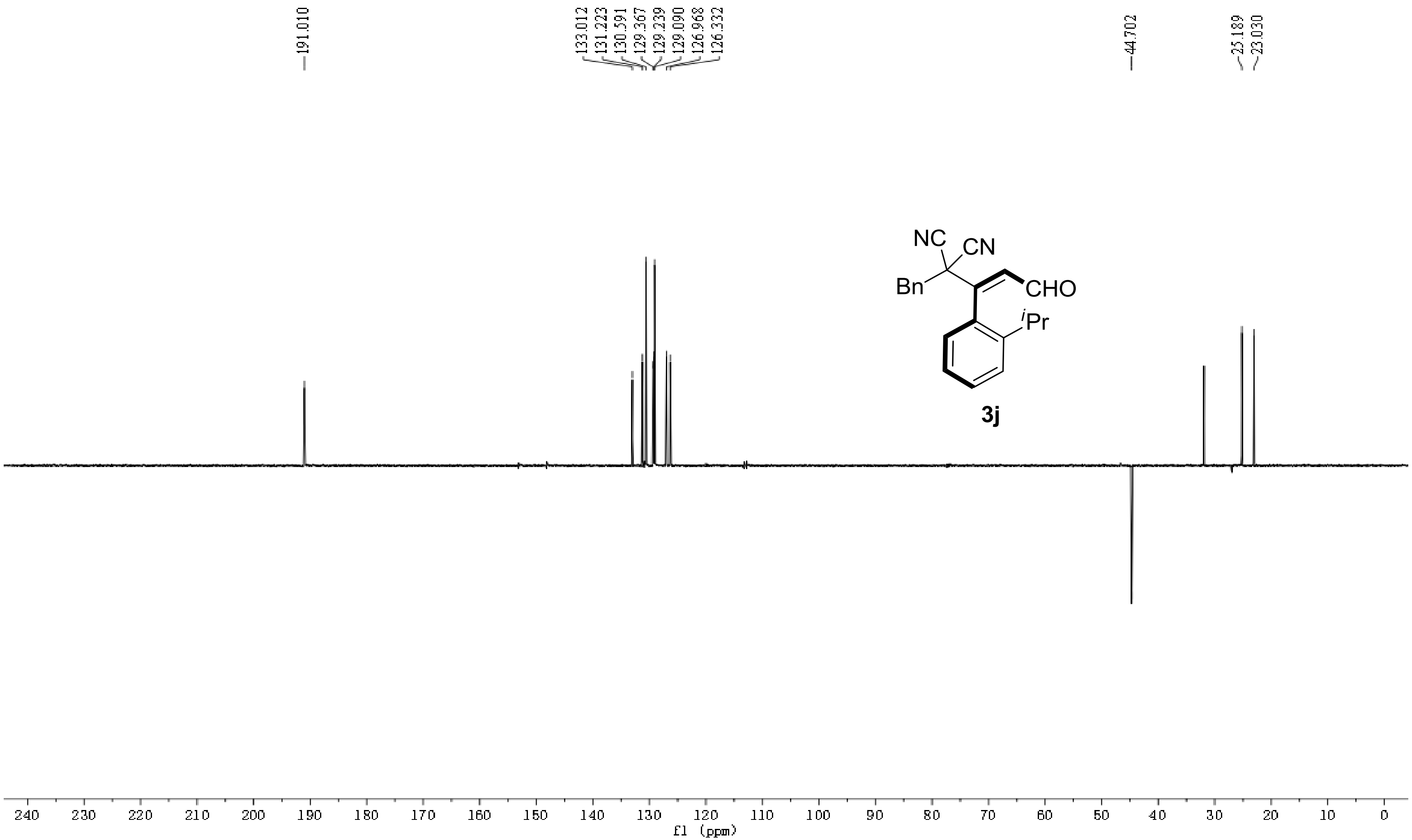

Supplementary Figure 43. DEPT-135 of **3j**

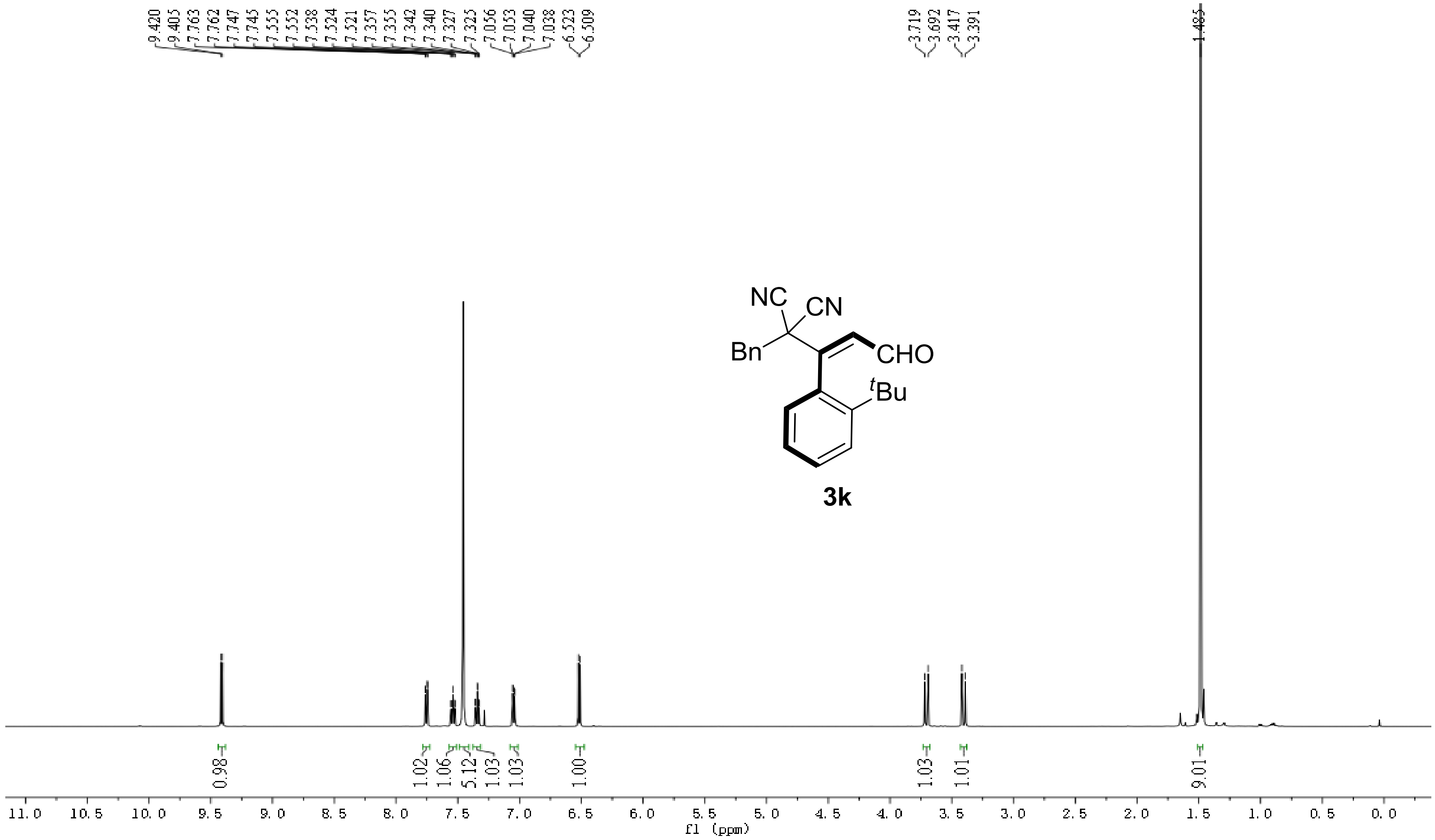

Supplementary Figure 44. ^{1}H NMR of **3k**

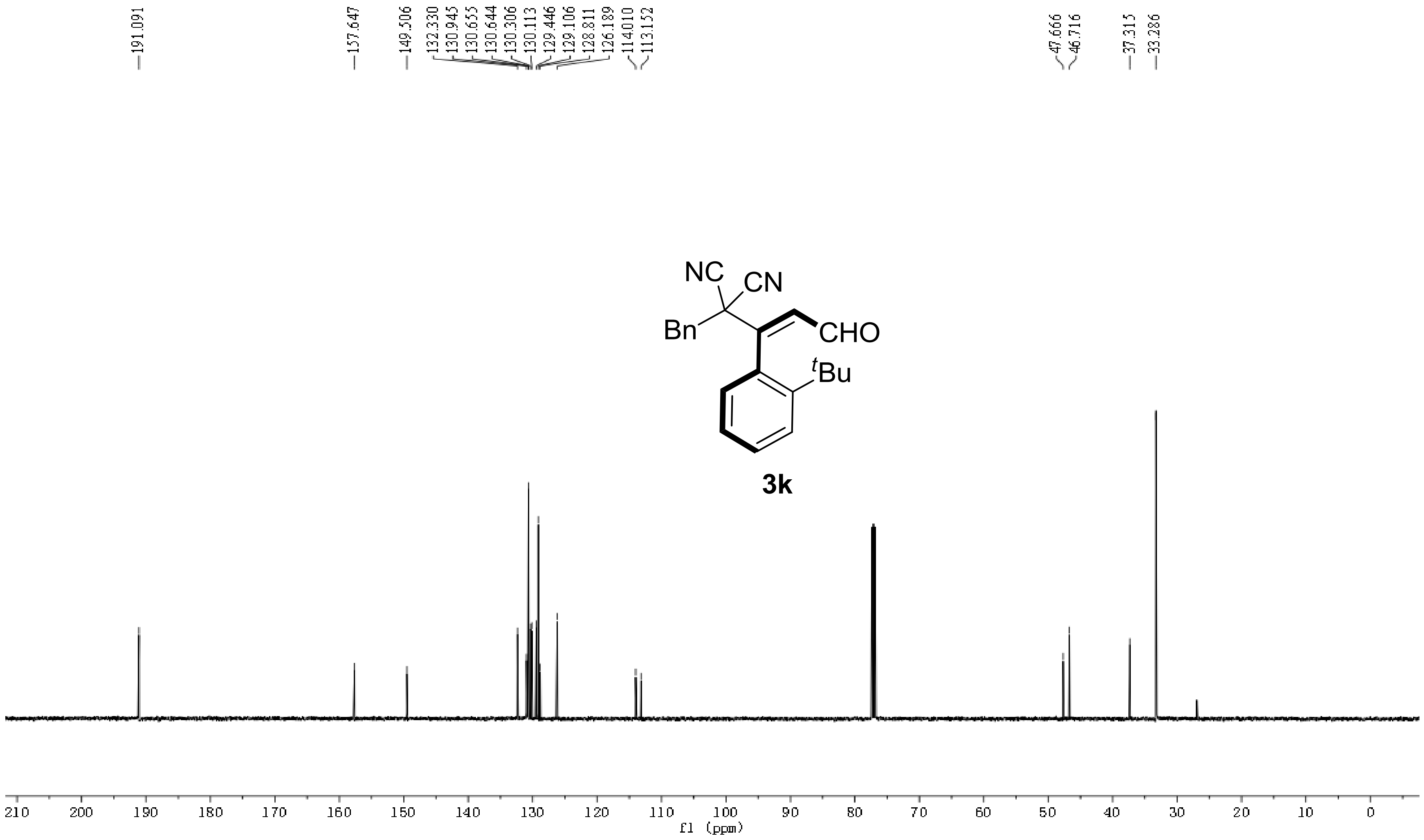

Supplementary Figure 45. ^{13}C NMR of **3k**

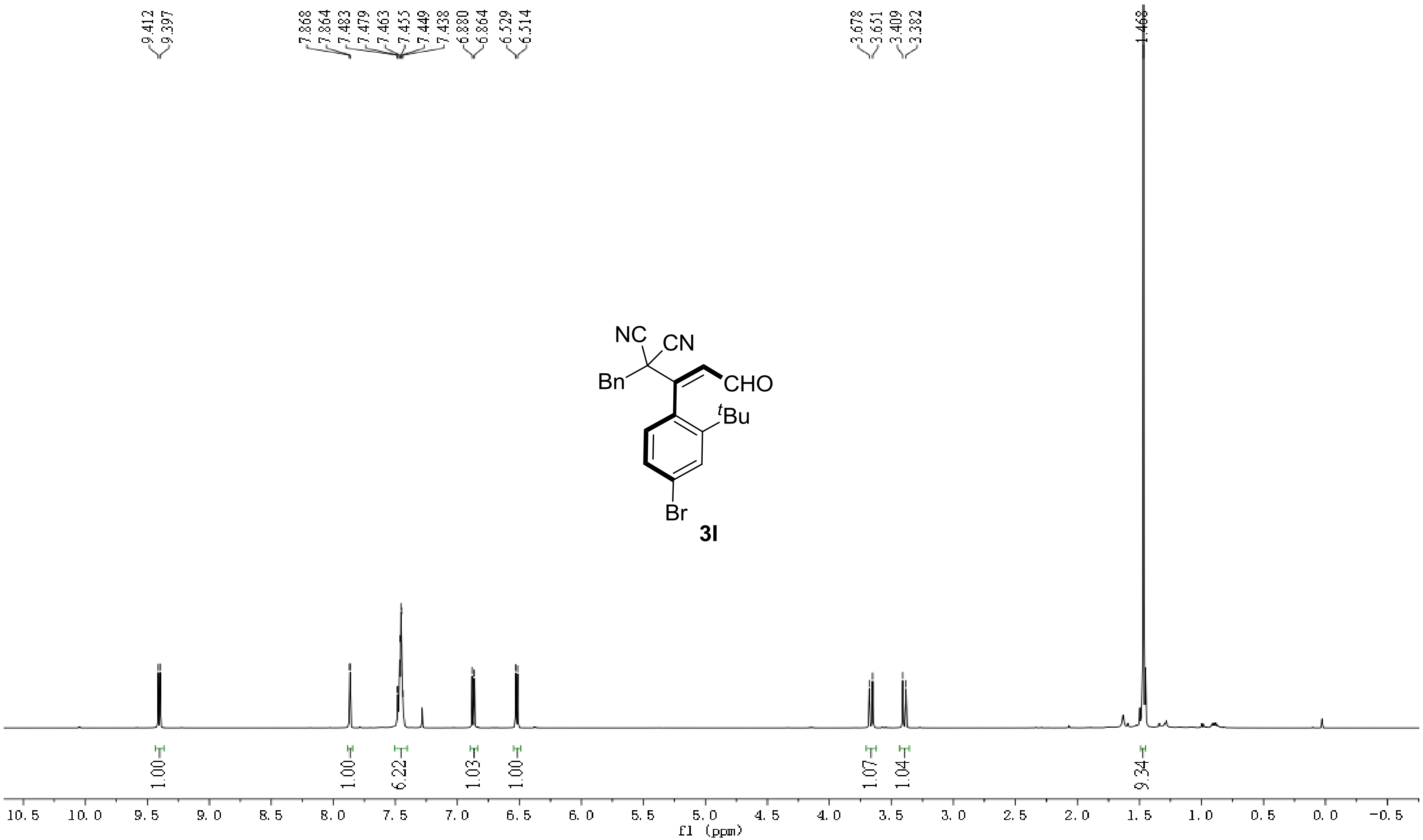

Supplementary Figure 46. ^{1}H NMR of **3l**

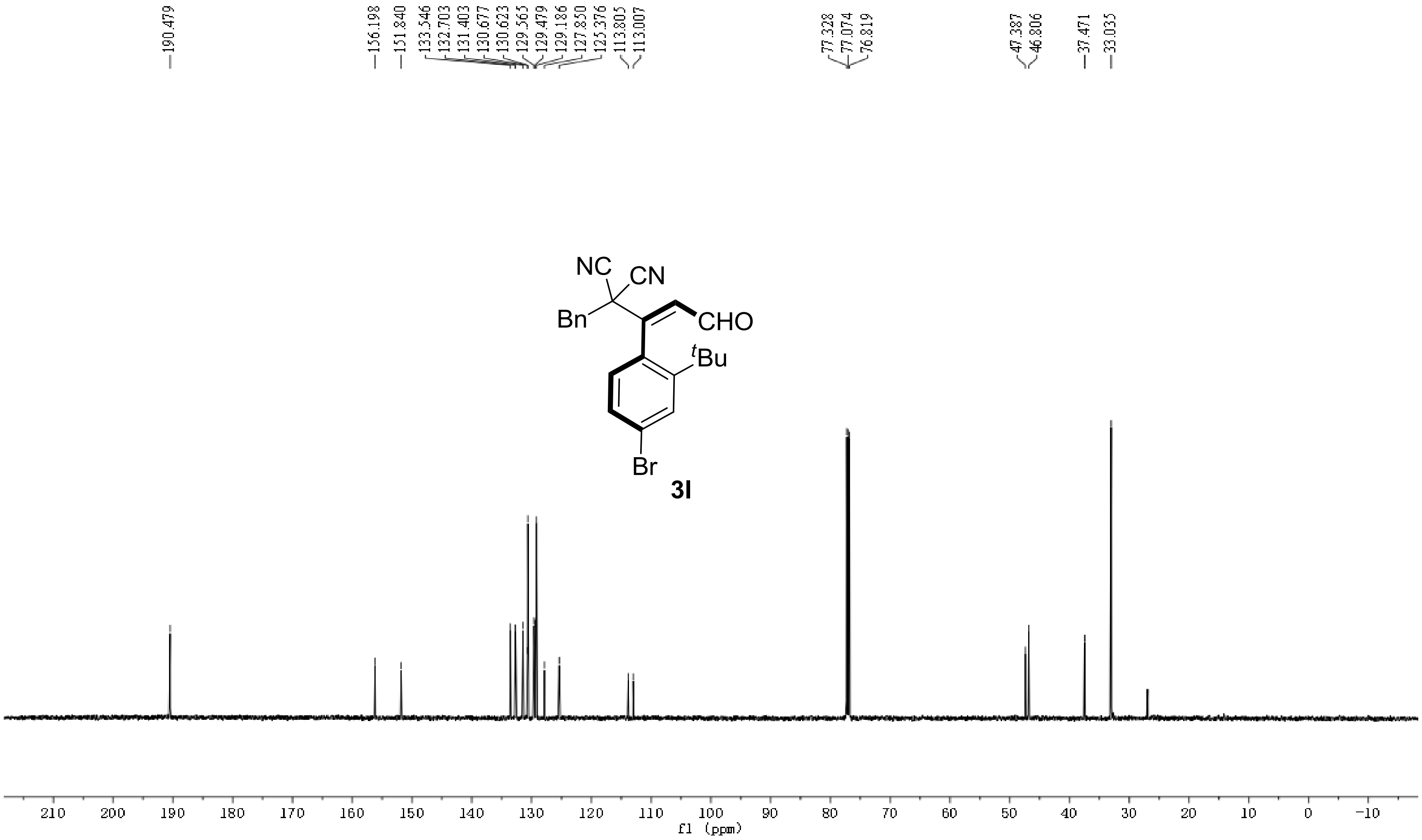

Supplementary Figure 47. ^{13}C NMR of **3l**

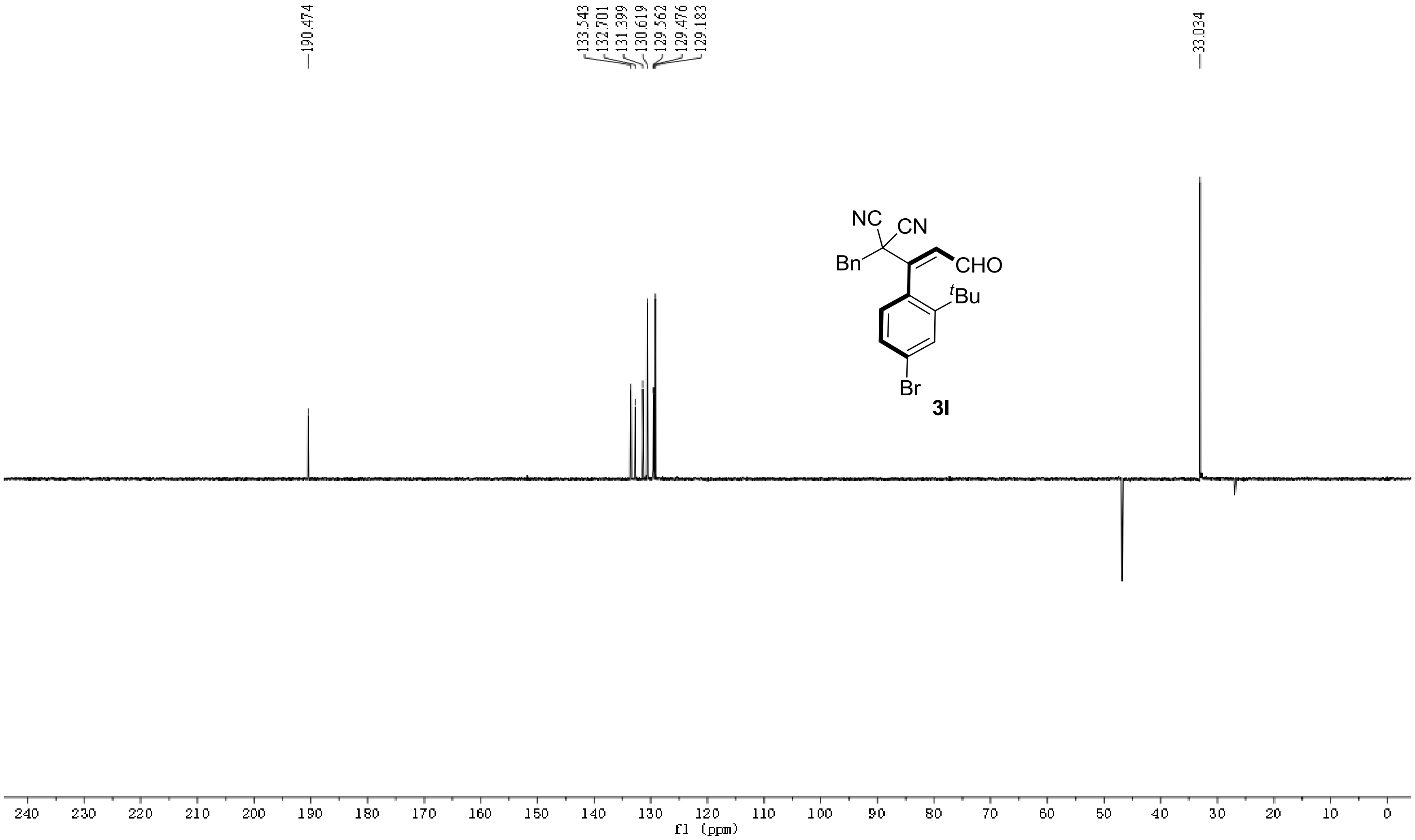

Supplementary Figure 48. DEPT-135 of **3l**

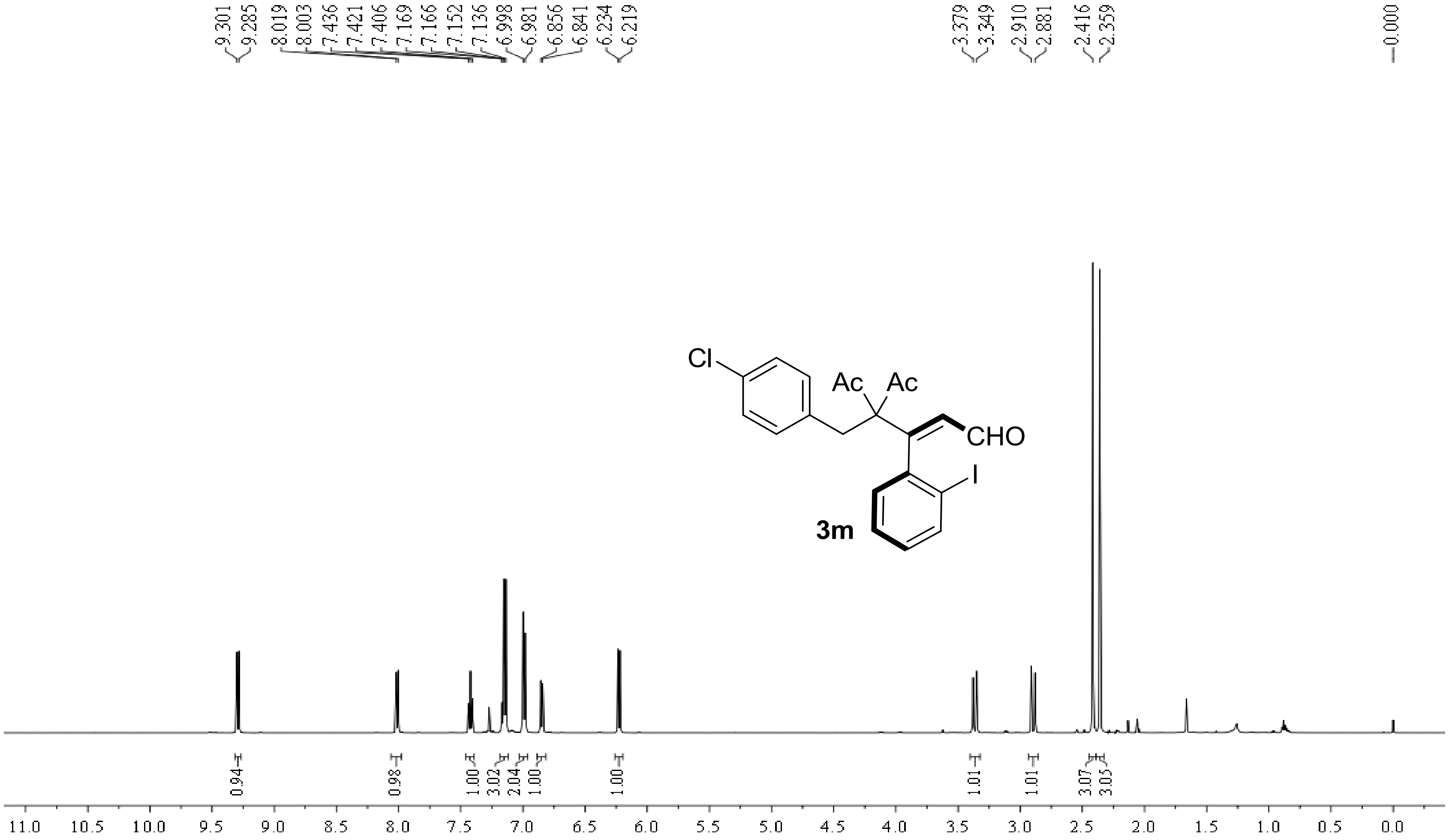

Supplementary Figure 49. ^{1}H NMR of **3m**

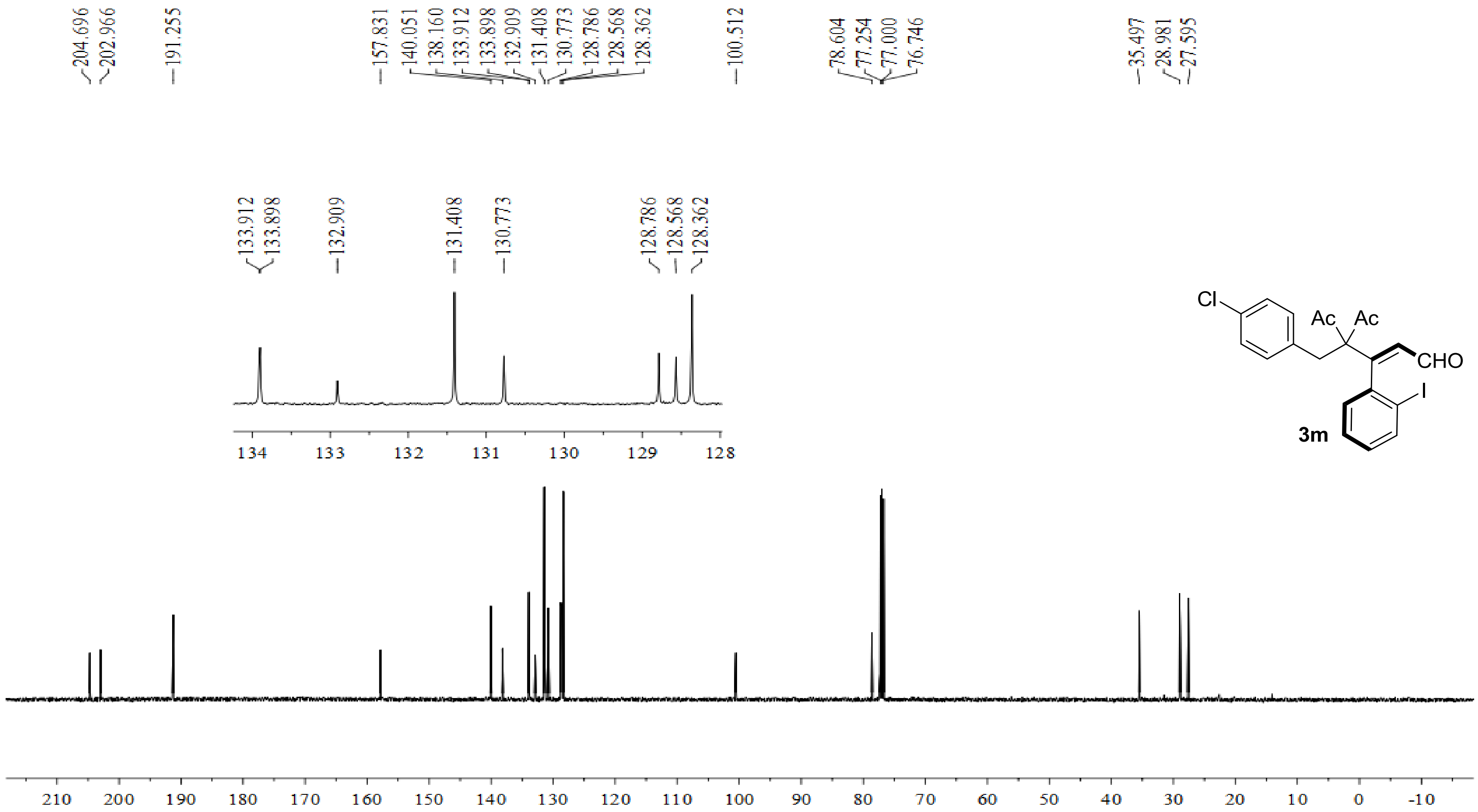

Supplementary Figure 50. ^{13}C NMR of **3m**

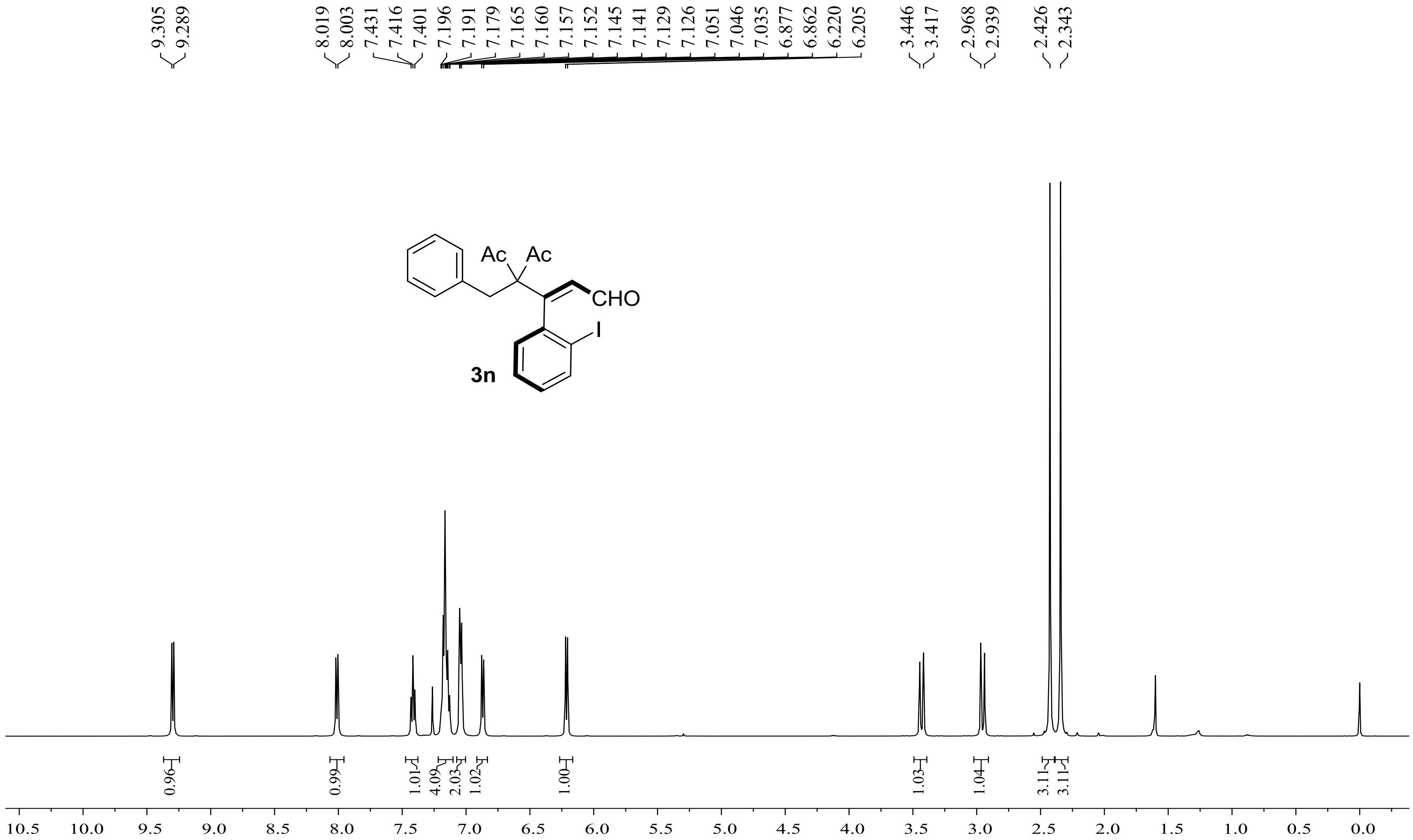

Supplementary Figure 51. ^{1}H NMR of **3n**

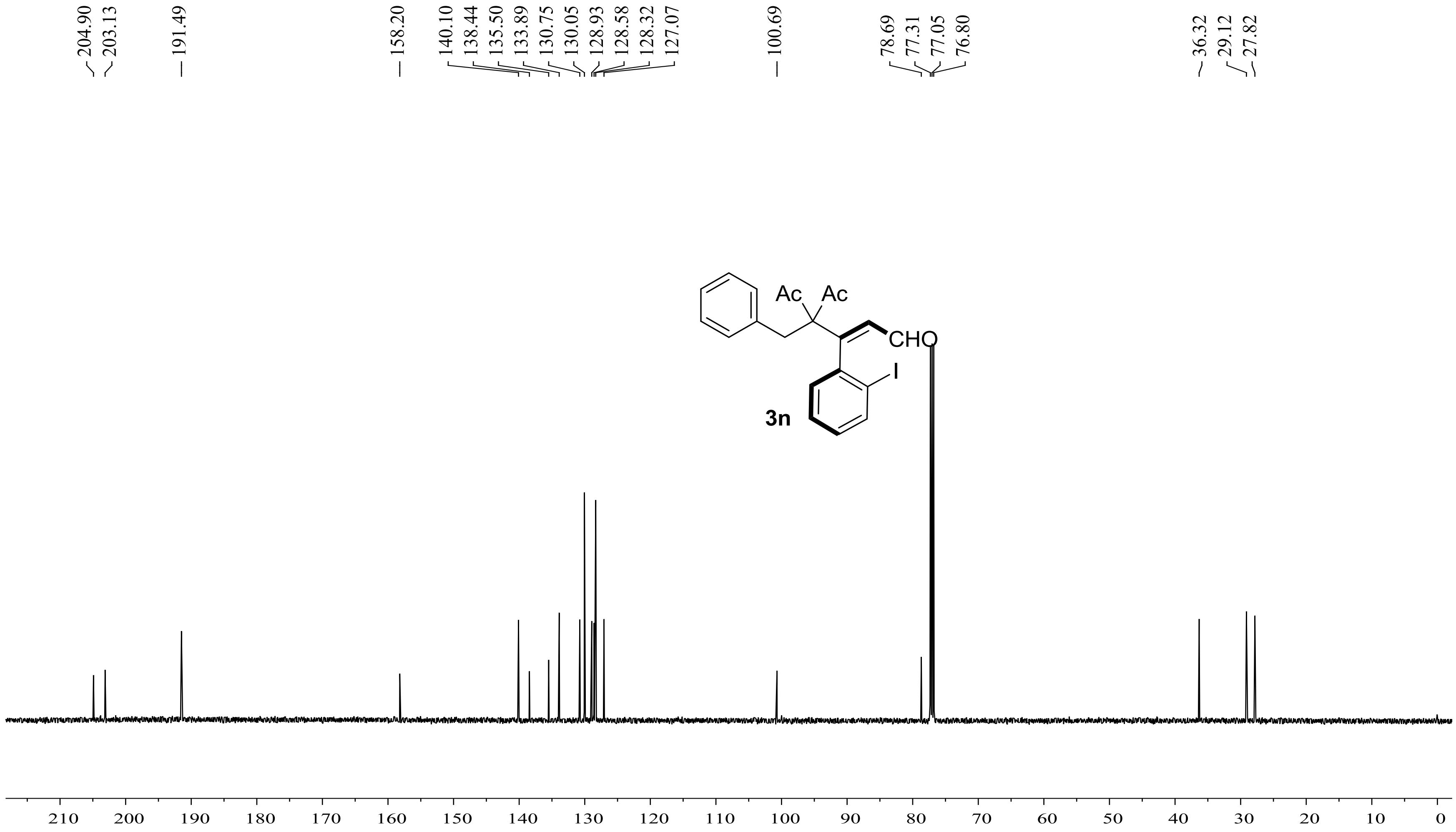

Supplementary Figure 52. ^{13}C NMR of **3n**

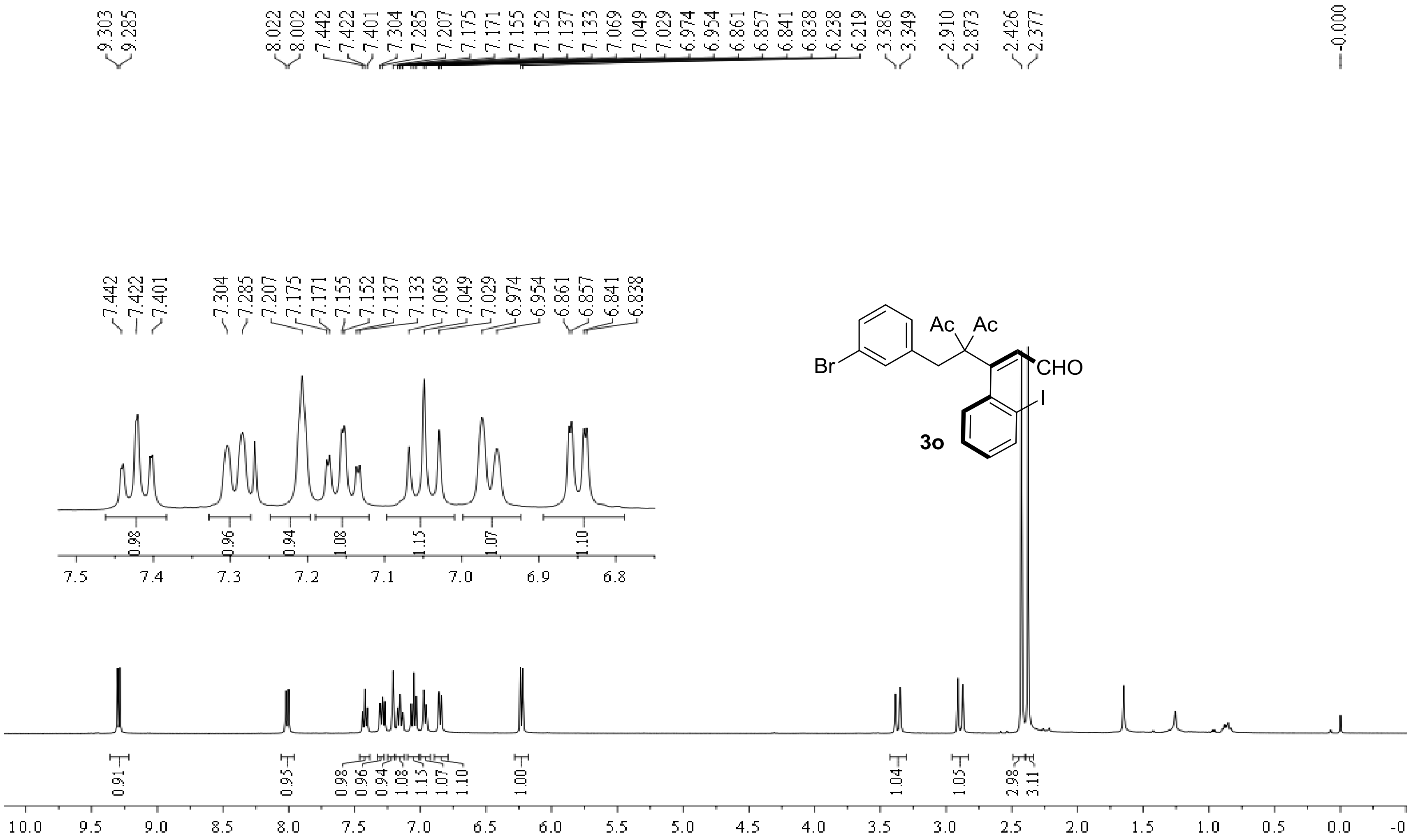

Supplementary Figure 53. ^{1}H NMR of **3o**

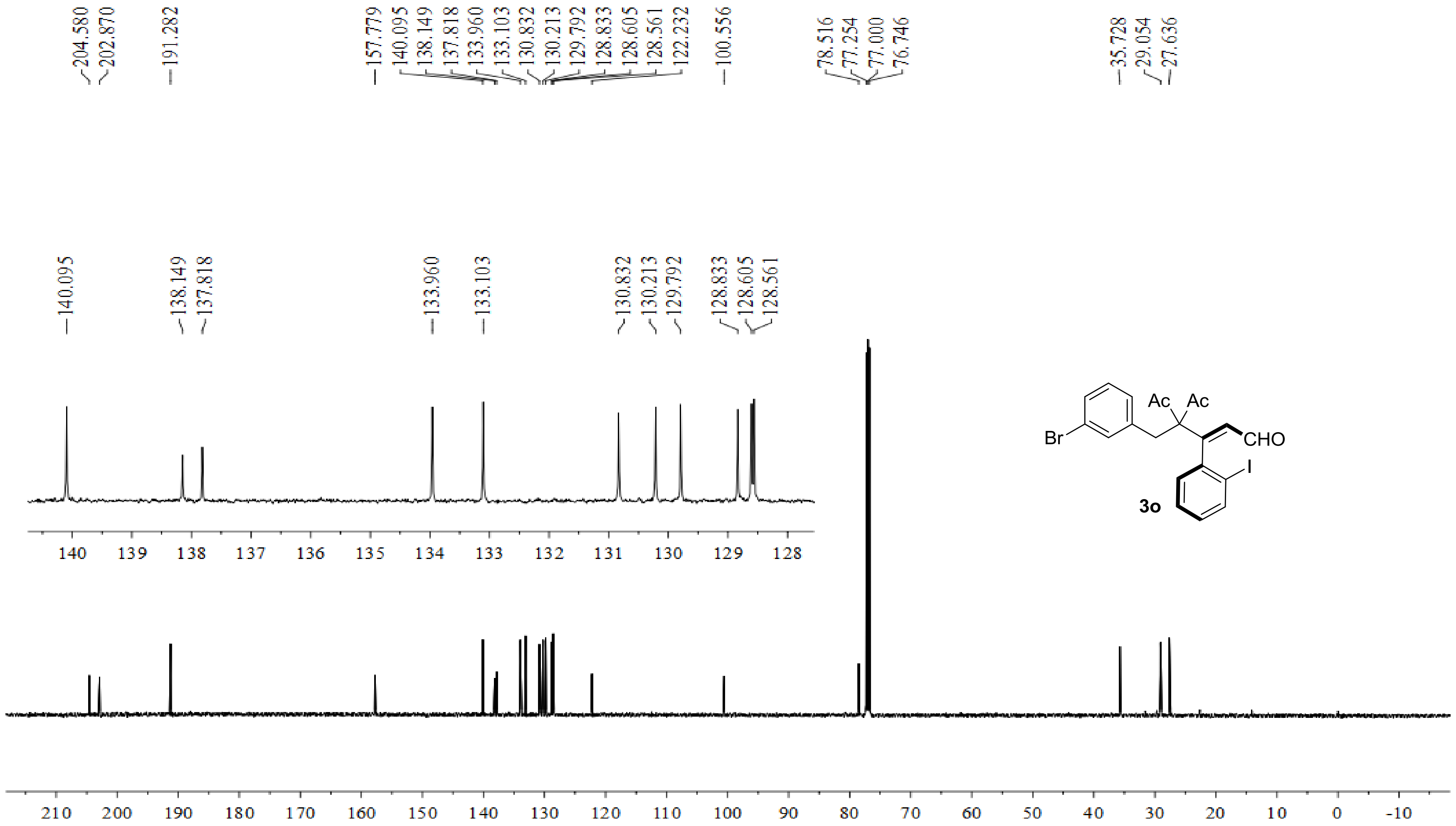

Supplementary Figure 54. ^{13}C NMR of **3o**

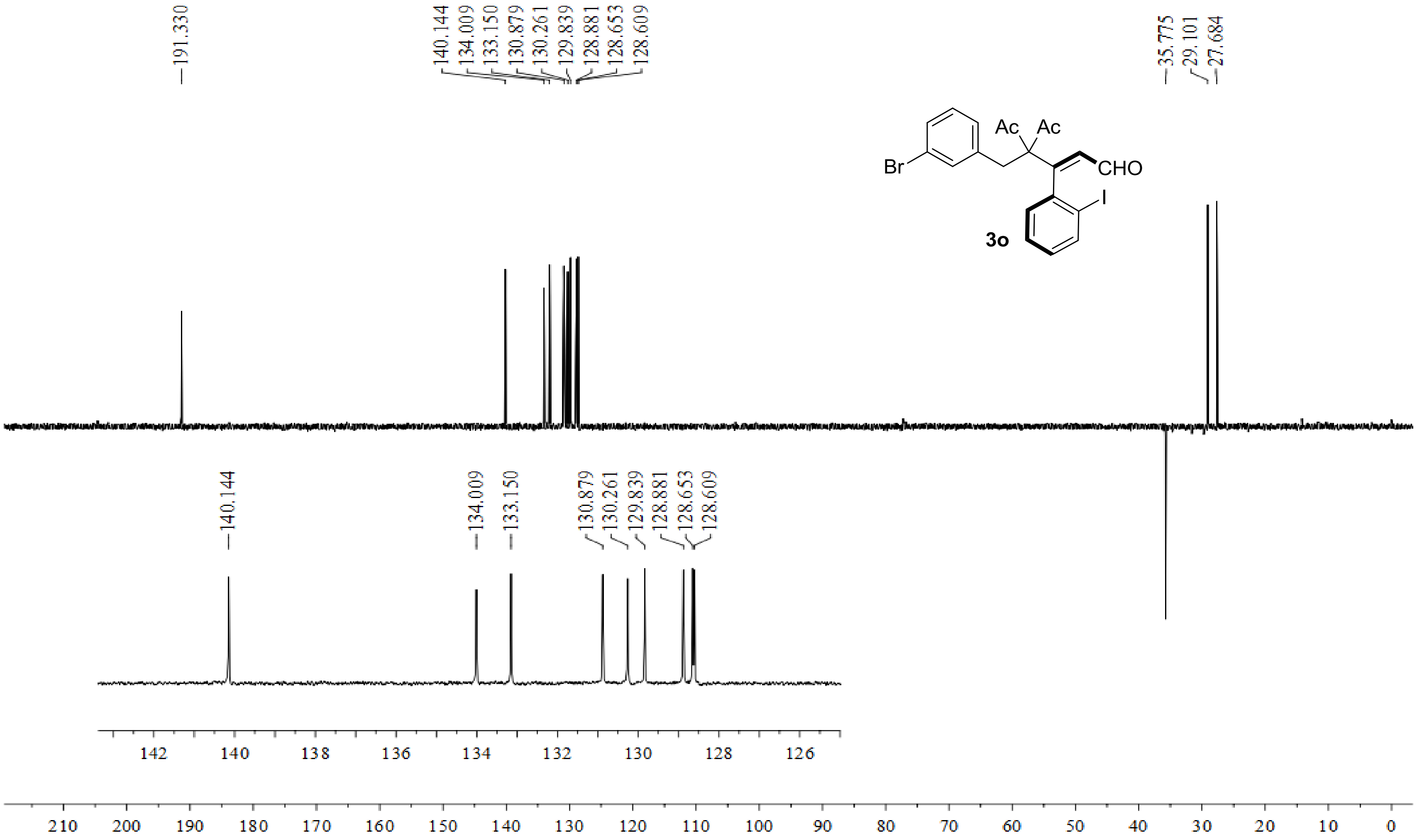

Supplementary Figure 55. DEPT-135 of **3o**

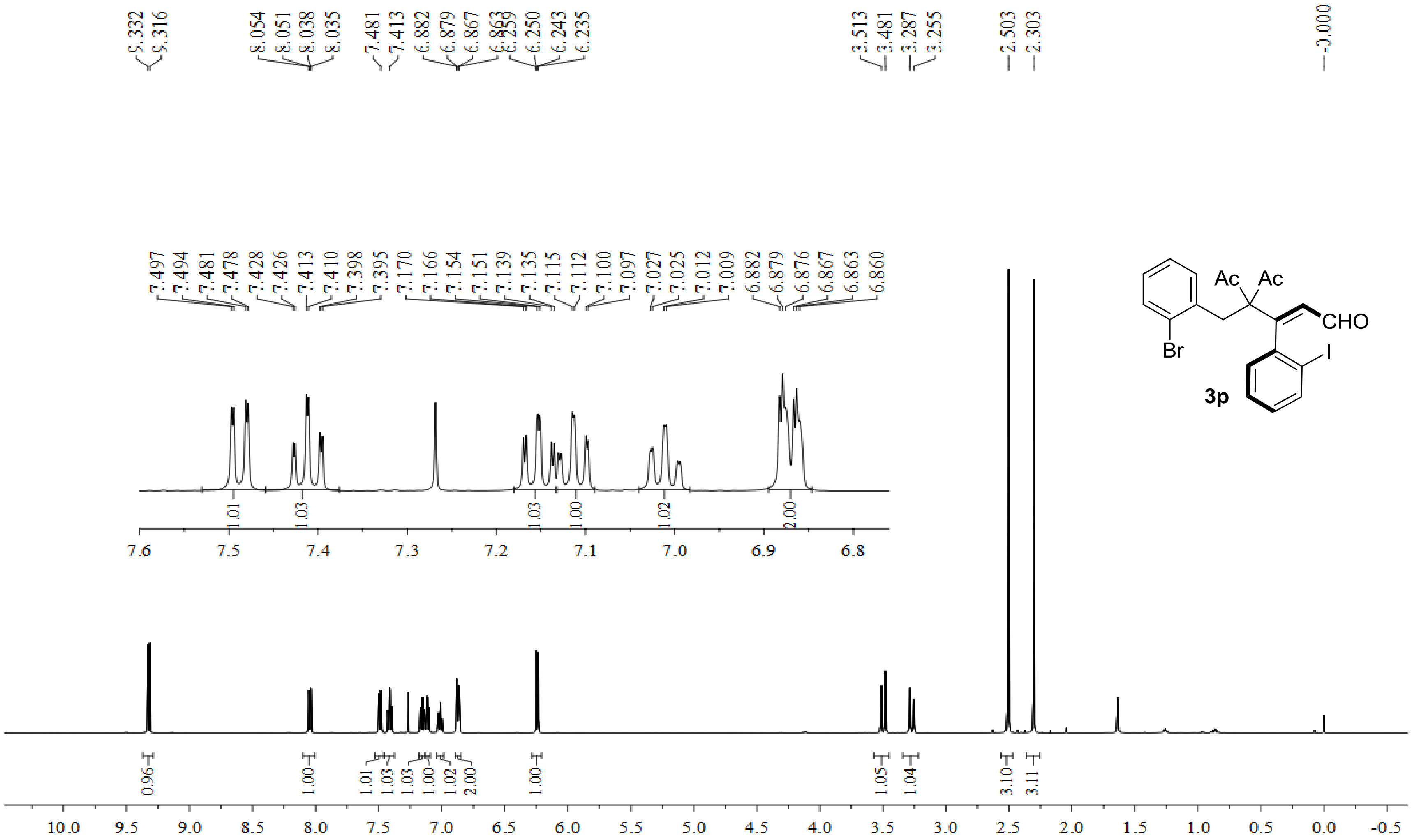

Supplementary Figure 56. ^{1}H NMR of **3p**

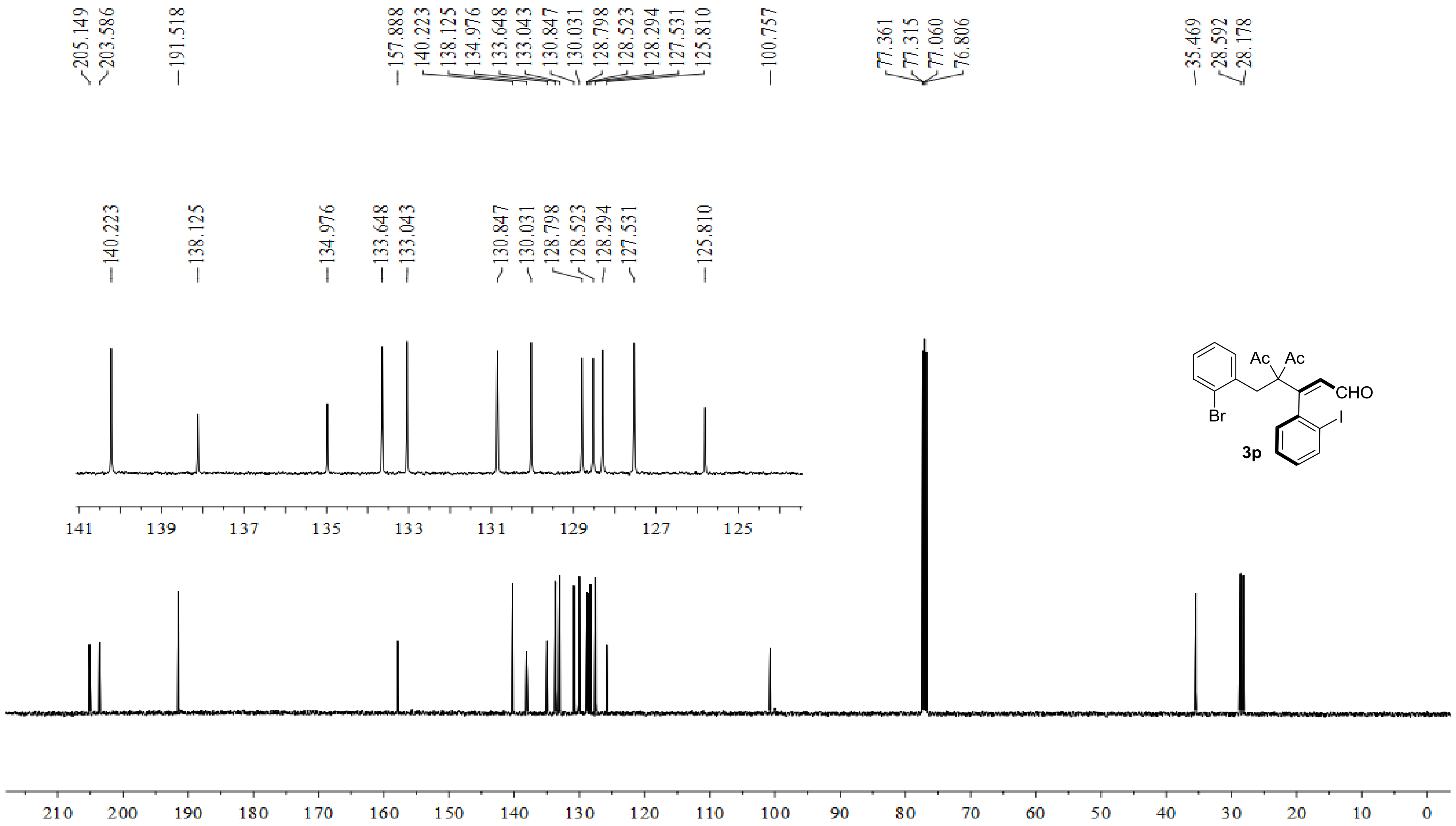

Supplementary Figure 57. ^{13}C NMR of **3p**

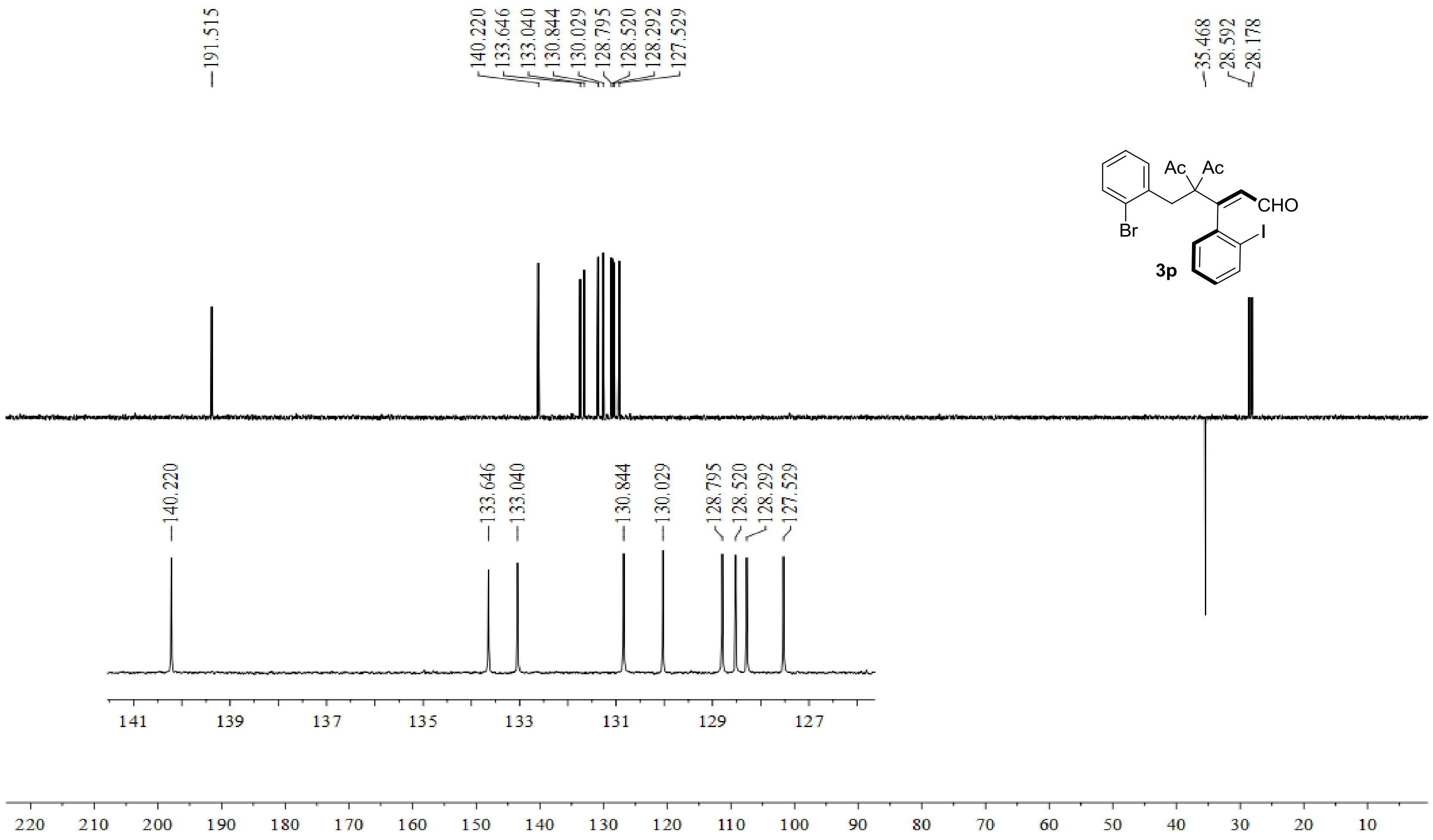

Supplementary Figure 58. DEPT-135 of **3p**

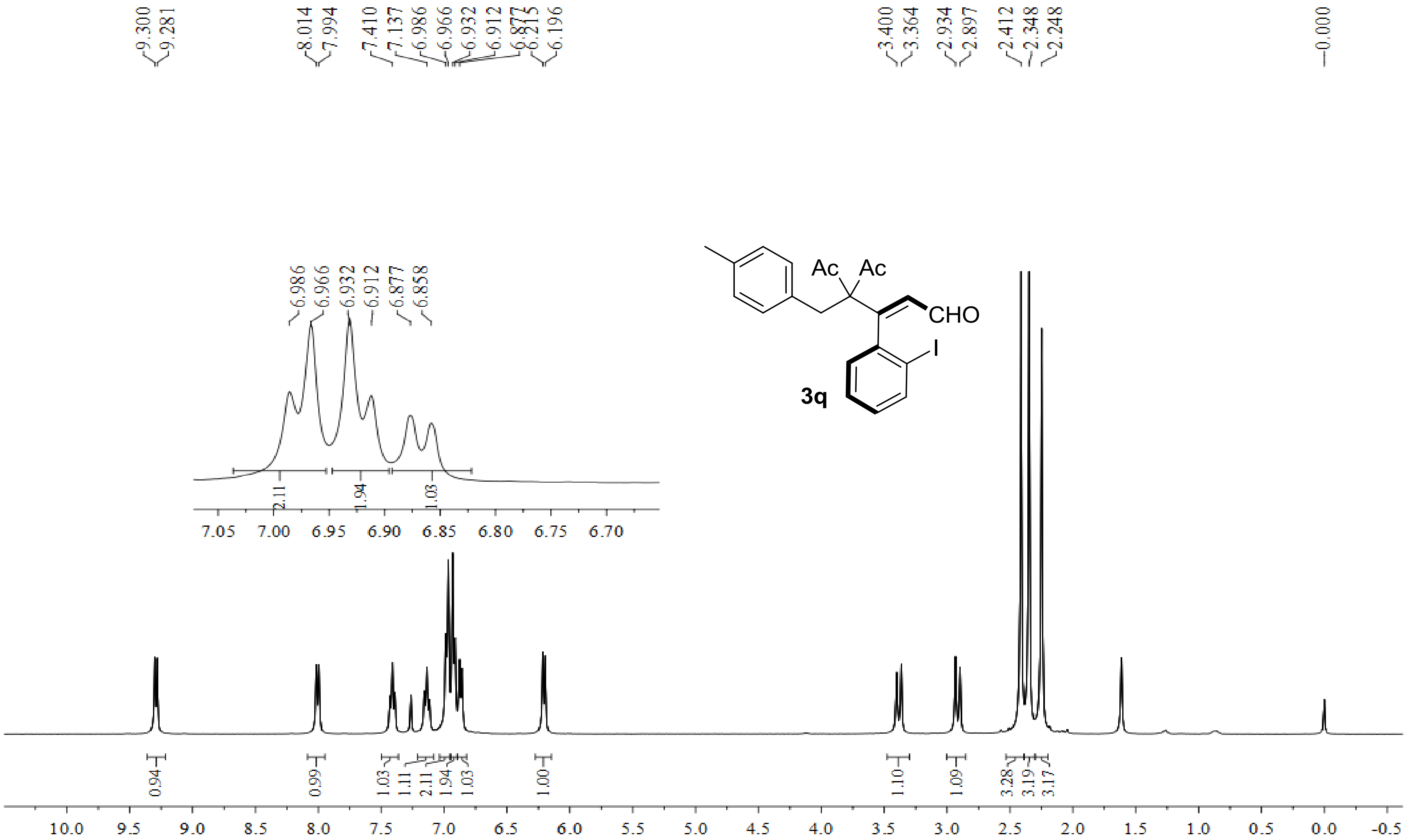

Supplementary Figure 59. ^{1}H NMR of **3q**

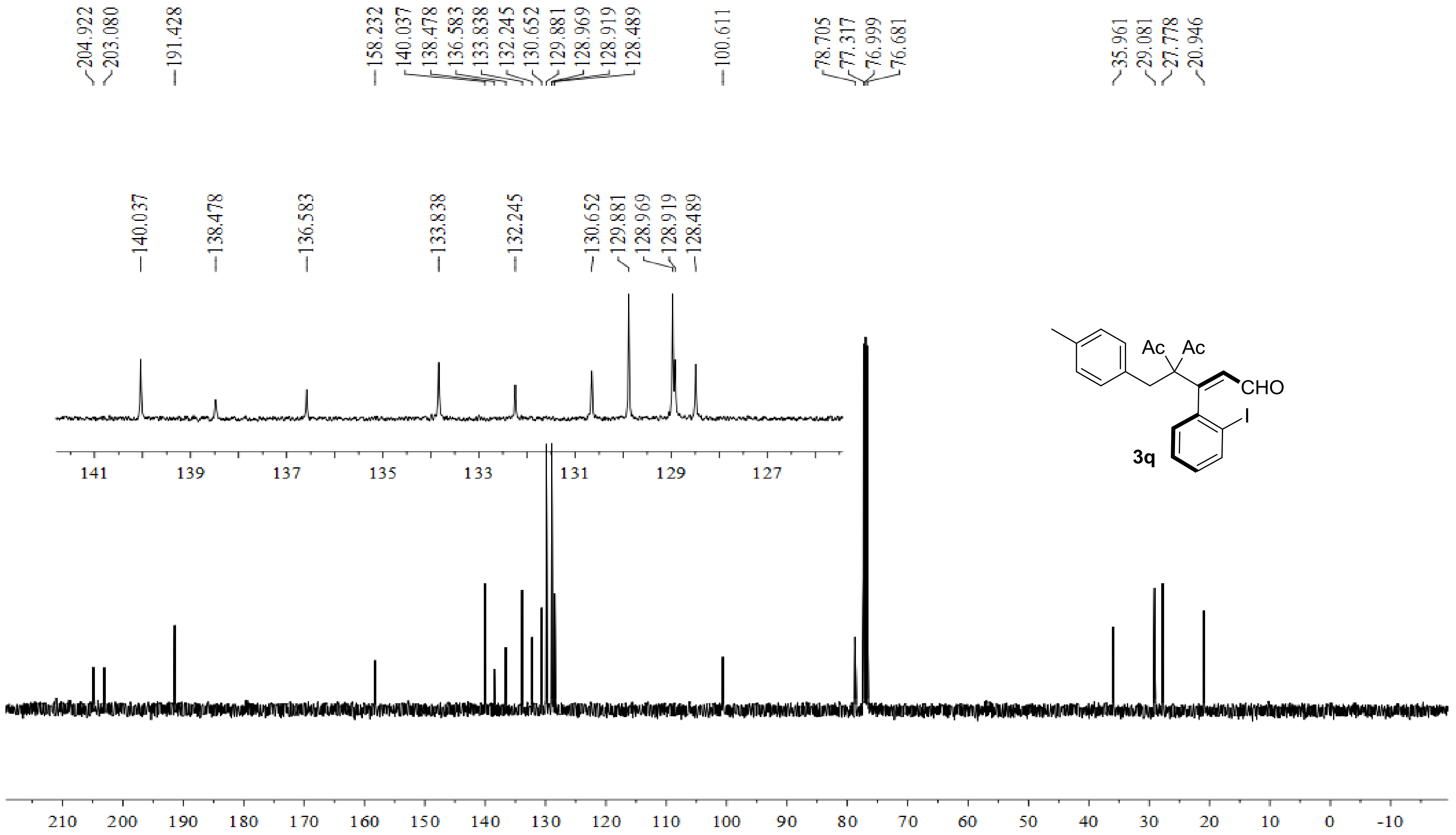

Supplementary Figure 60. ^{13}C NMR of **3q**

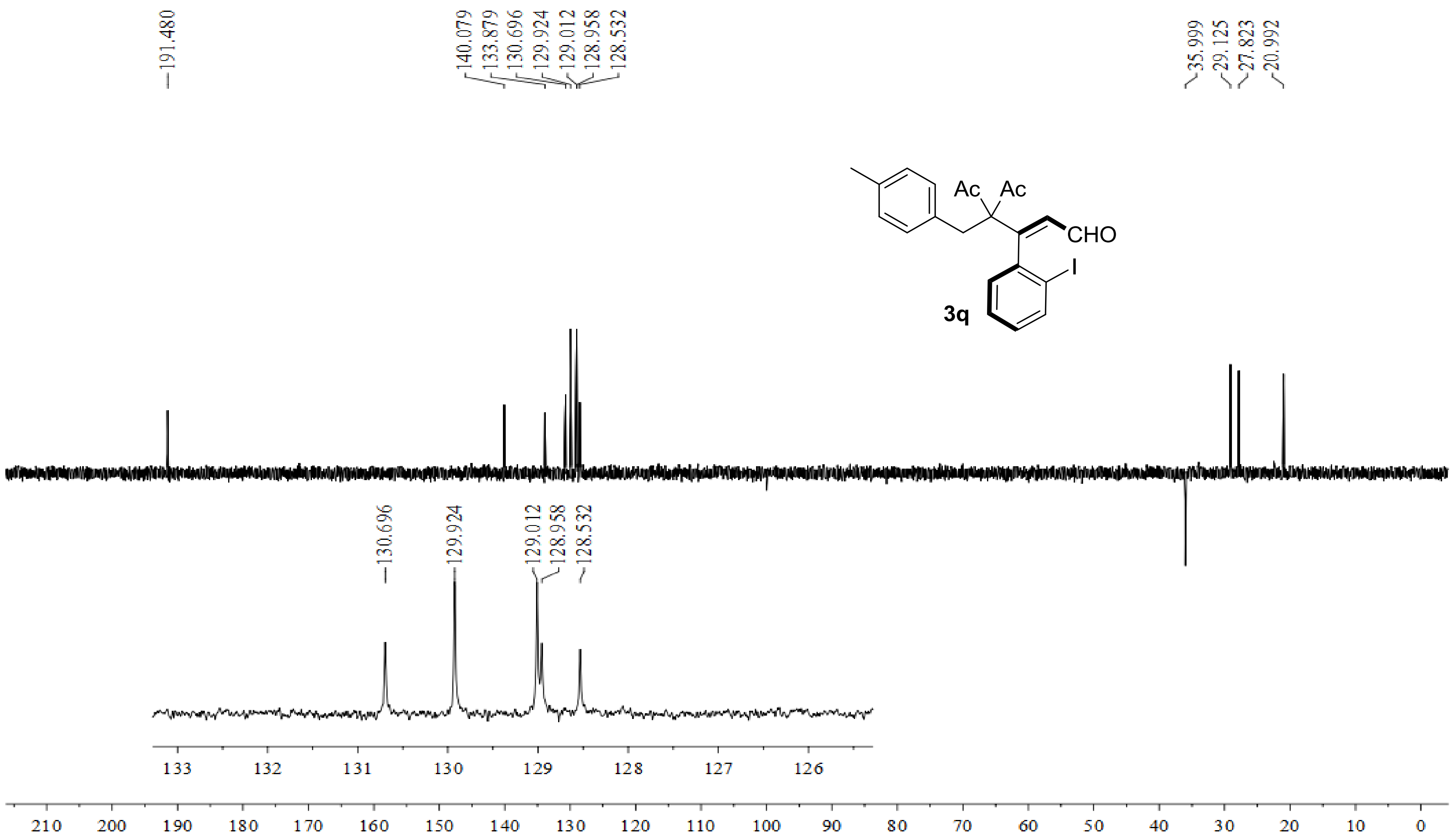

Supplementary Figure 61. DEPT-135 of **3q**

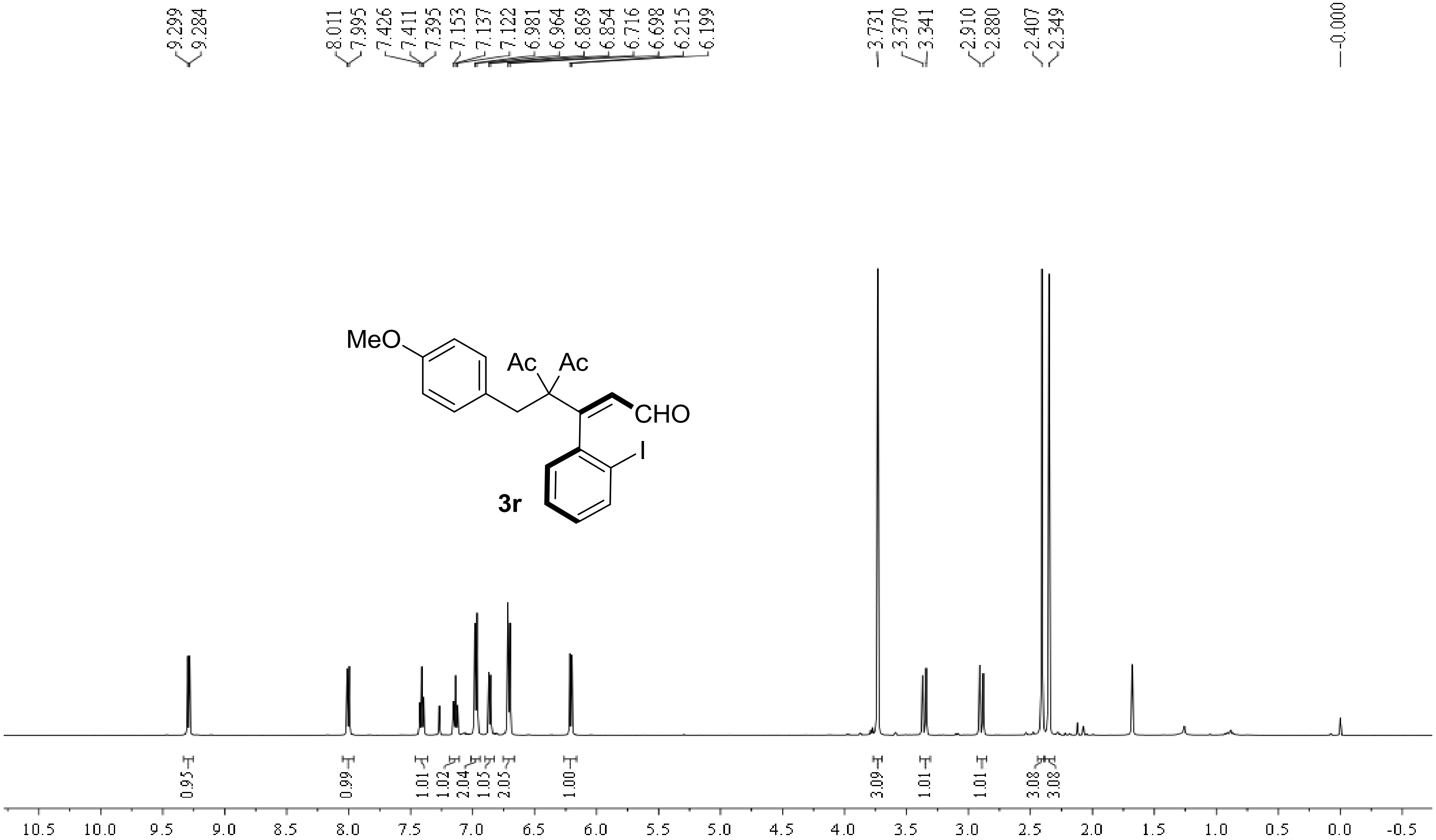

Supplementary Figure 62. ^{1}H NMR of **3r**

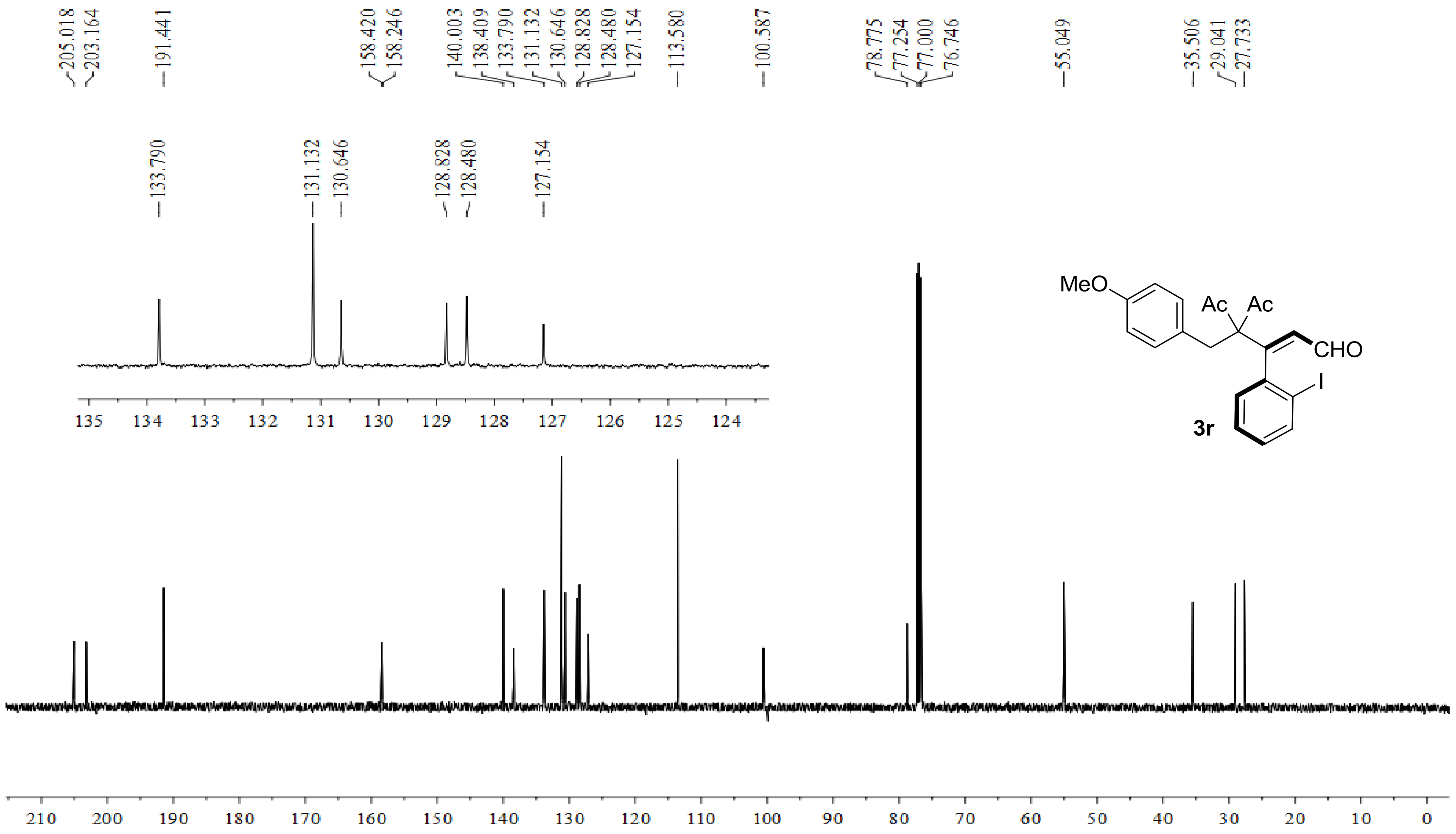

Supplementary Figure 63. ^{13}C NMR of **3r**

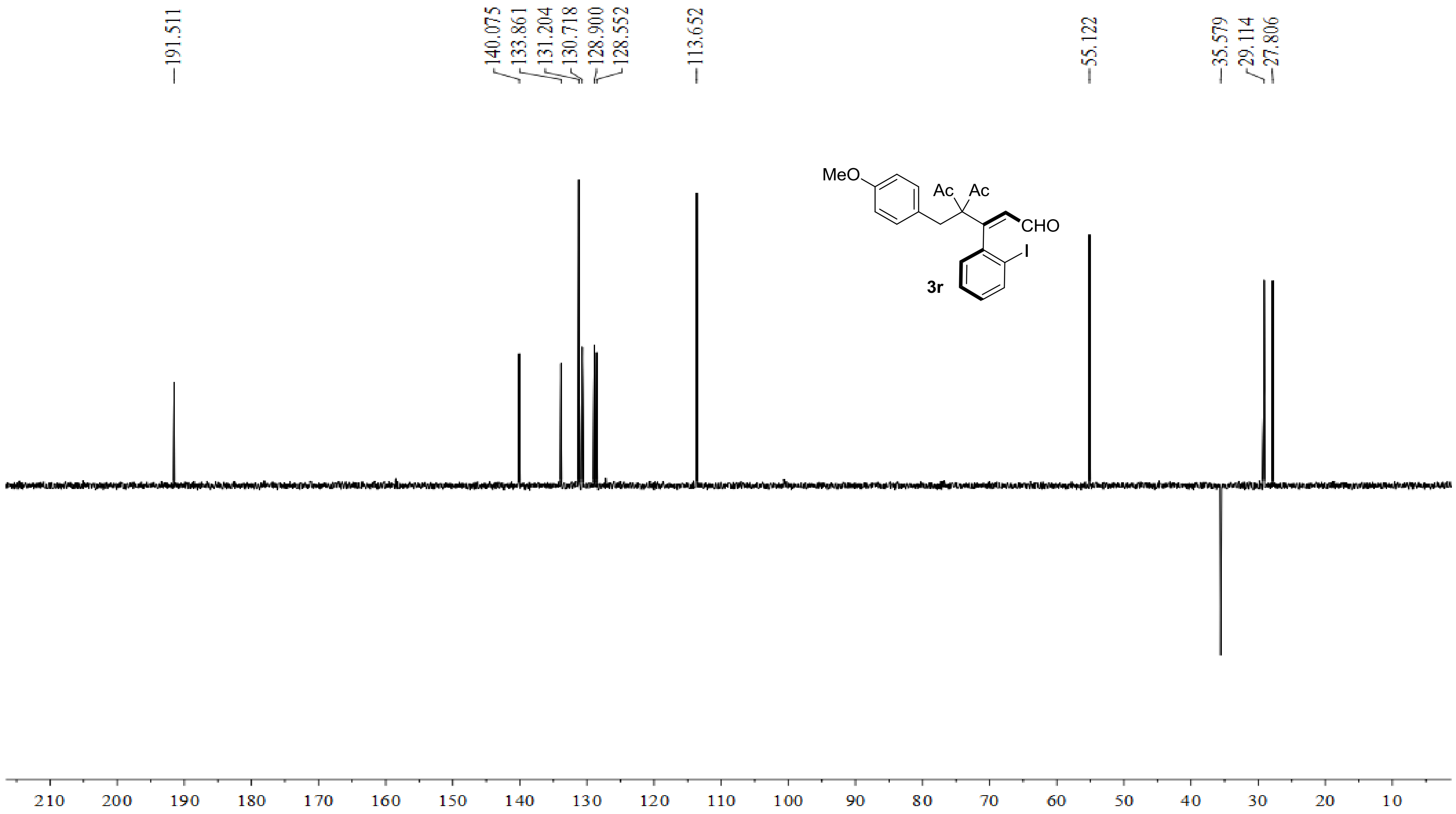

Supplementary Figure 64. DEPT-135 of **3r**

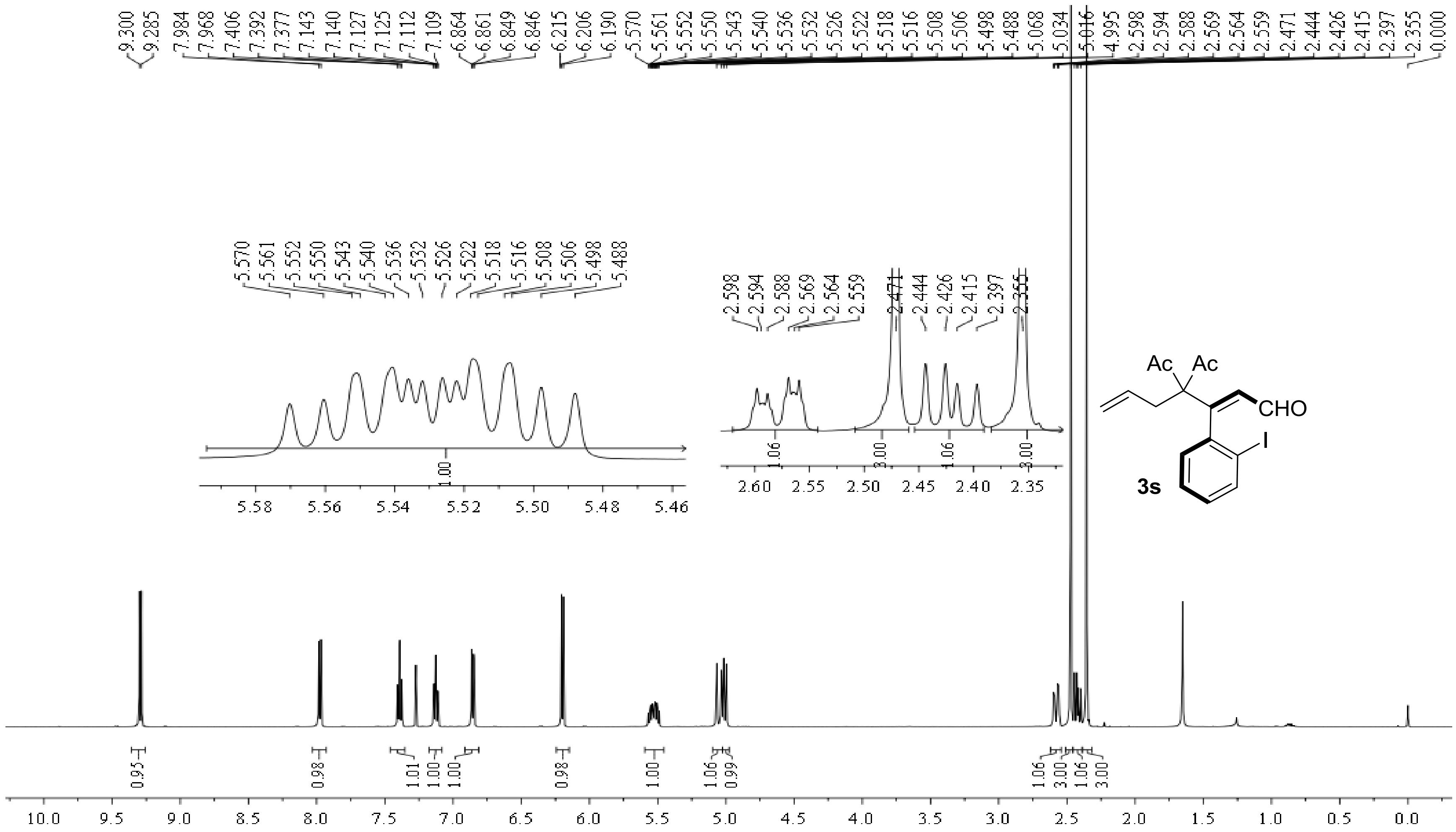

Supplementary Figure 65. ^{1}H NMR of **3s**

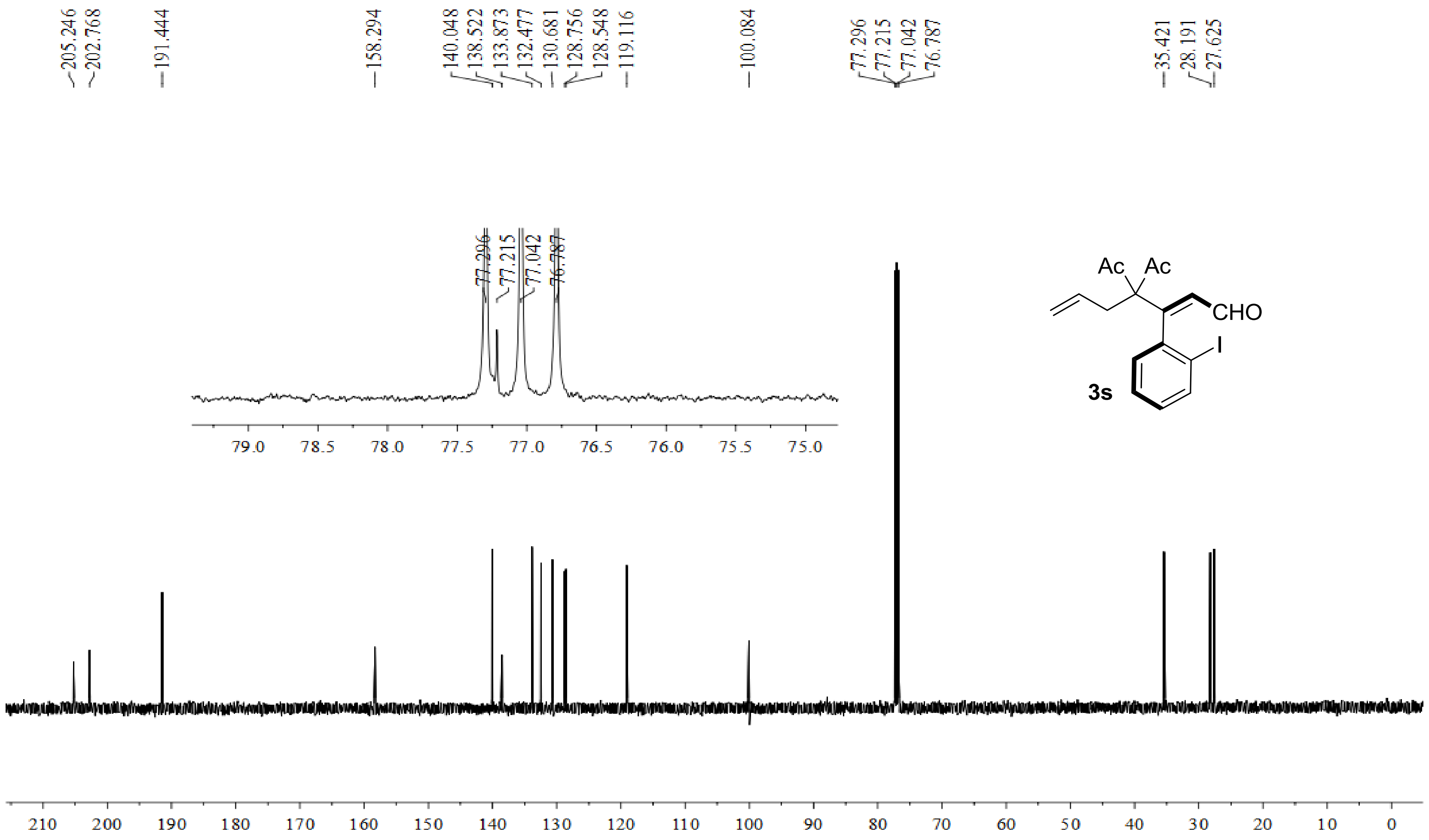

Supplementary Figure 66. ^{13}C NMR of **3s**

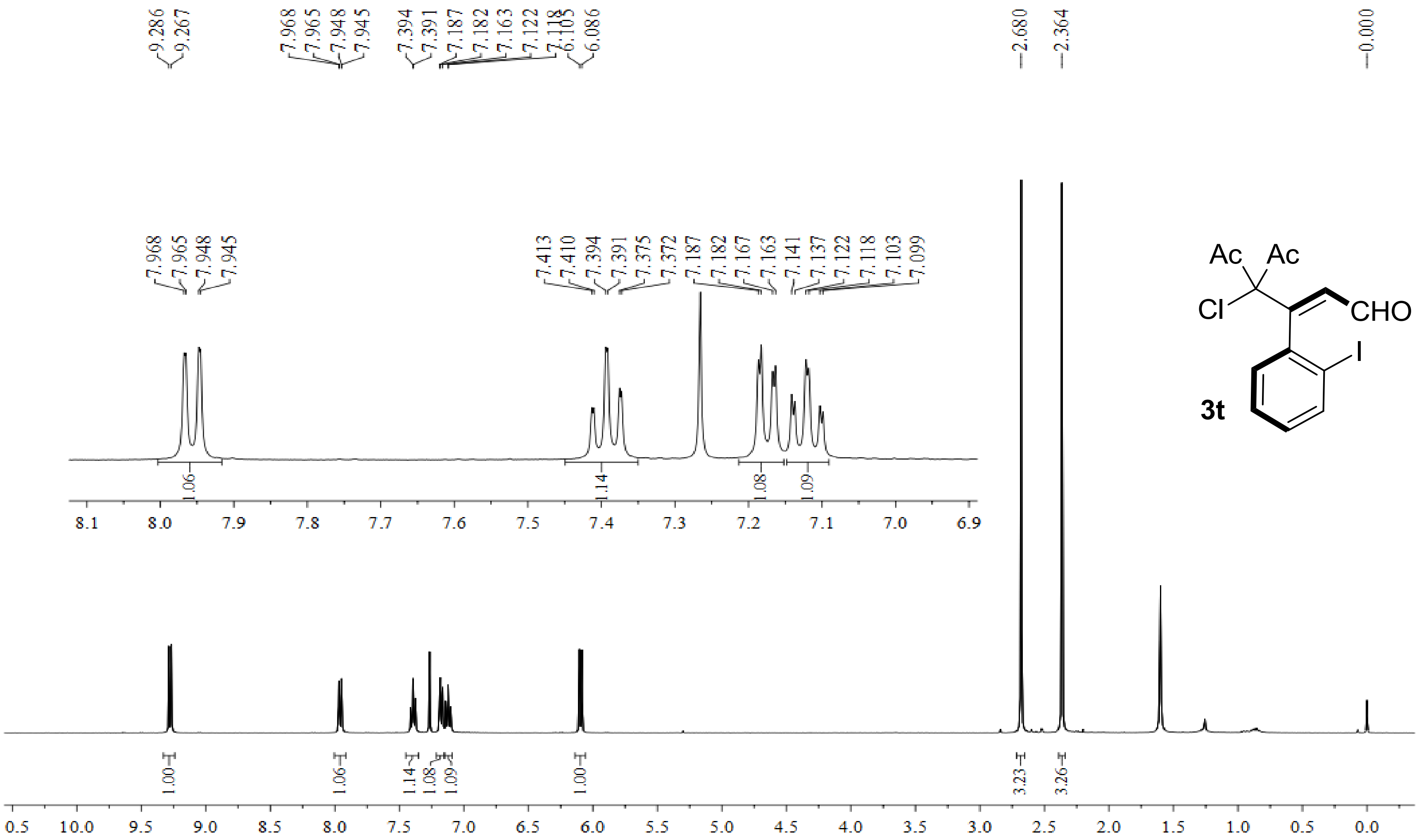

Supplementary Figure 67. ^{1}H NMR of **3t**

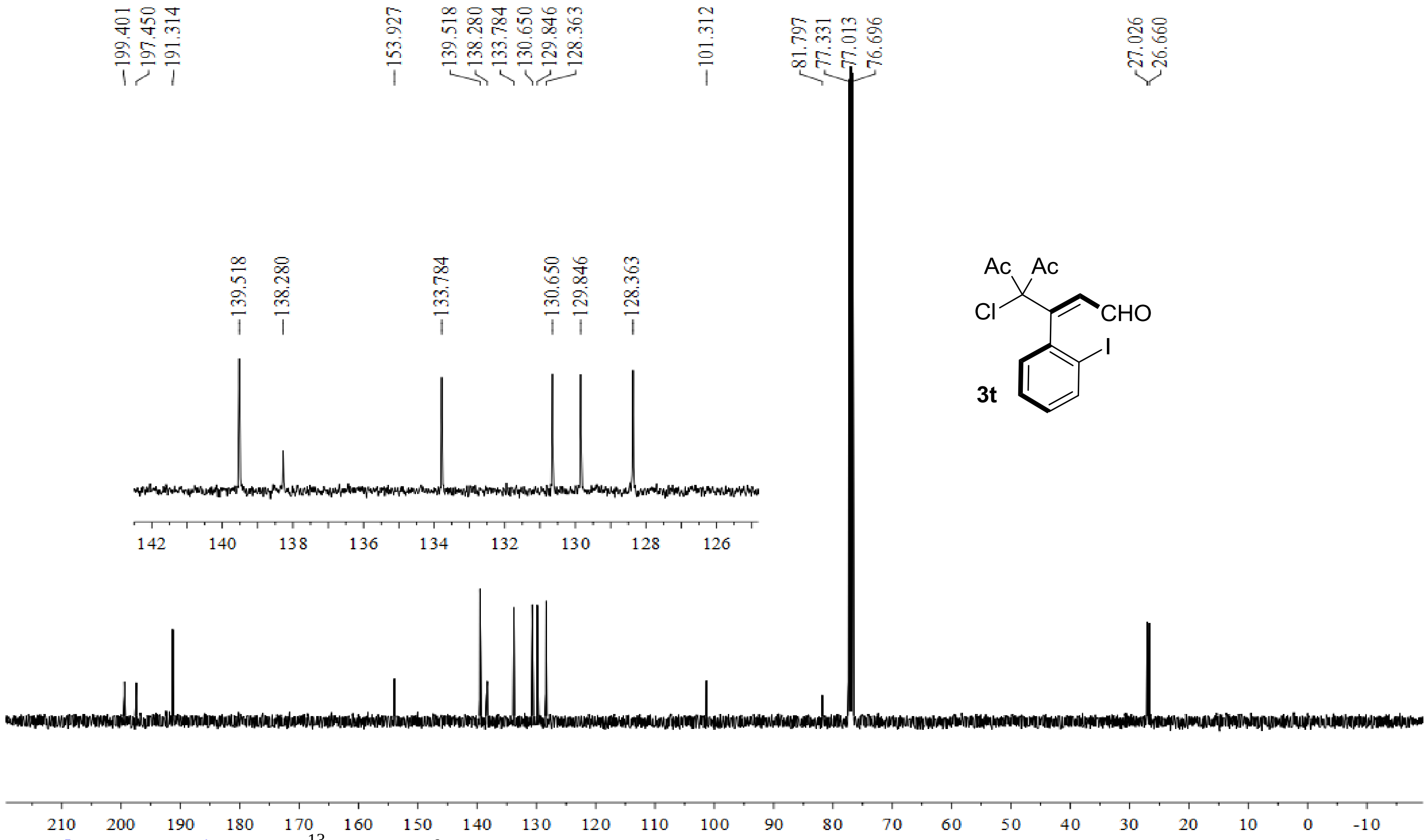

Supplementary Figure 68. ^{13}C NMR of **3t**

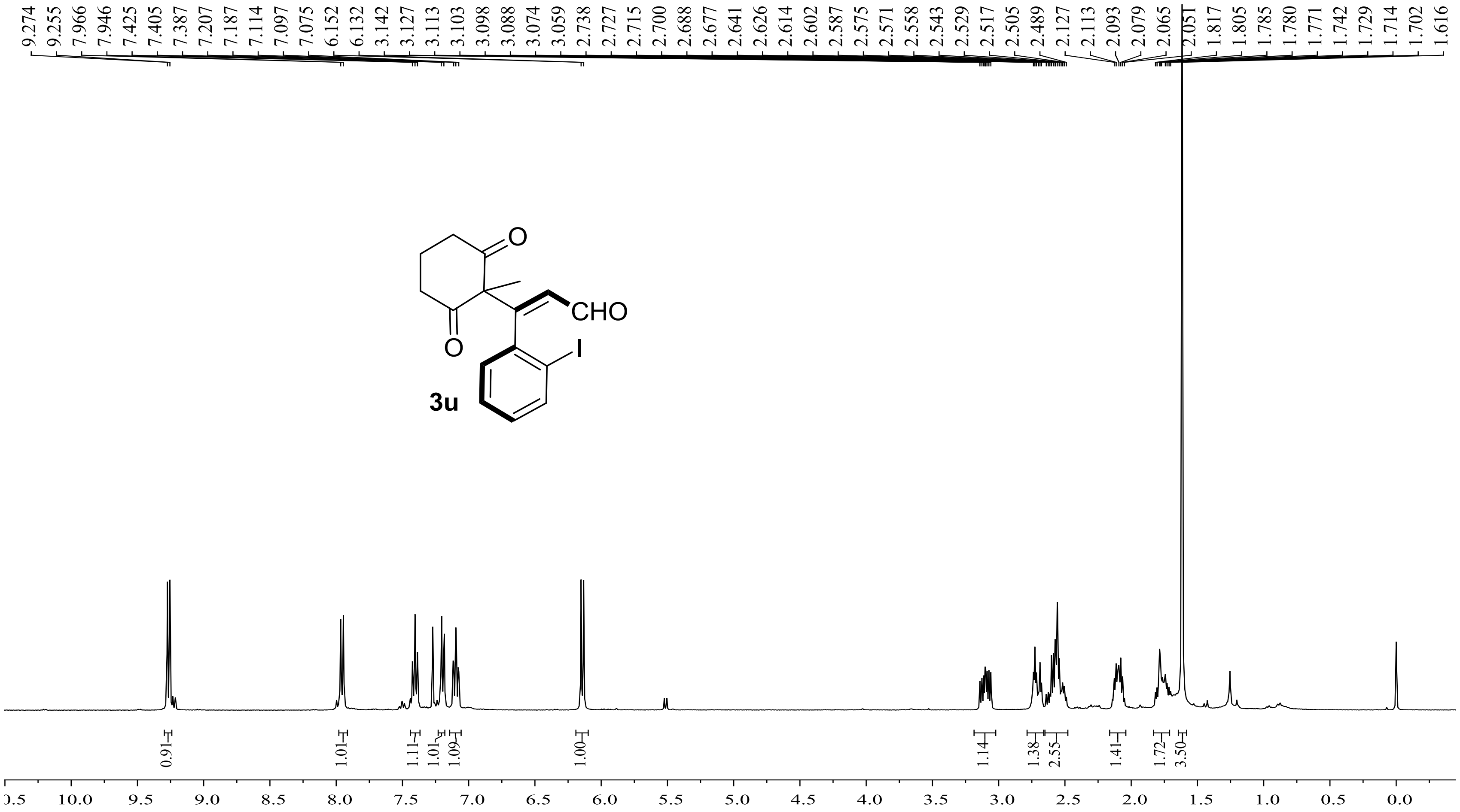

Supplementary Figure 69. ^{1}H NMR of **3u**

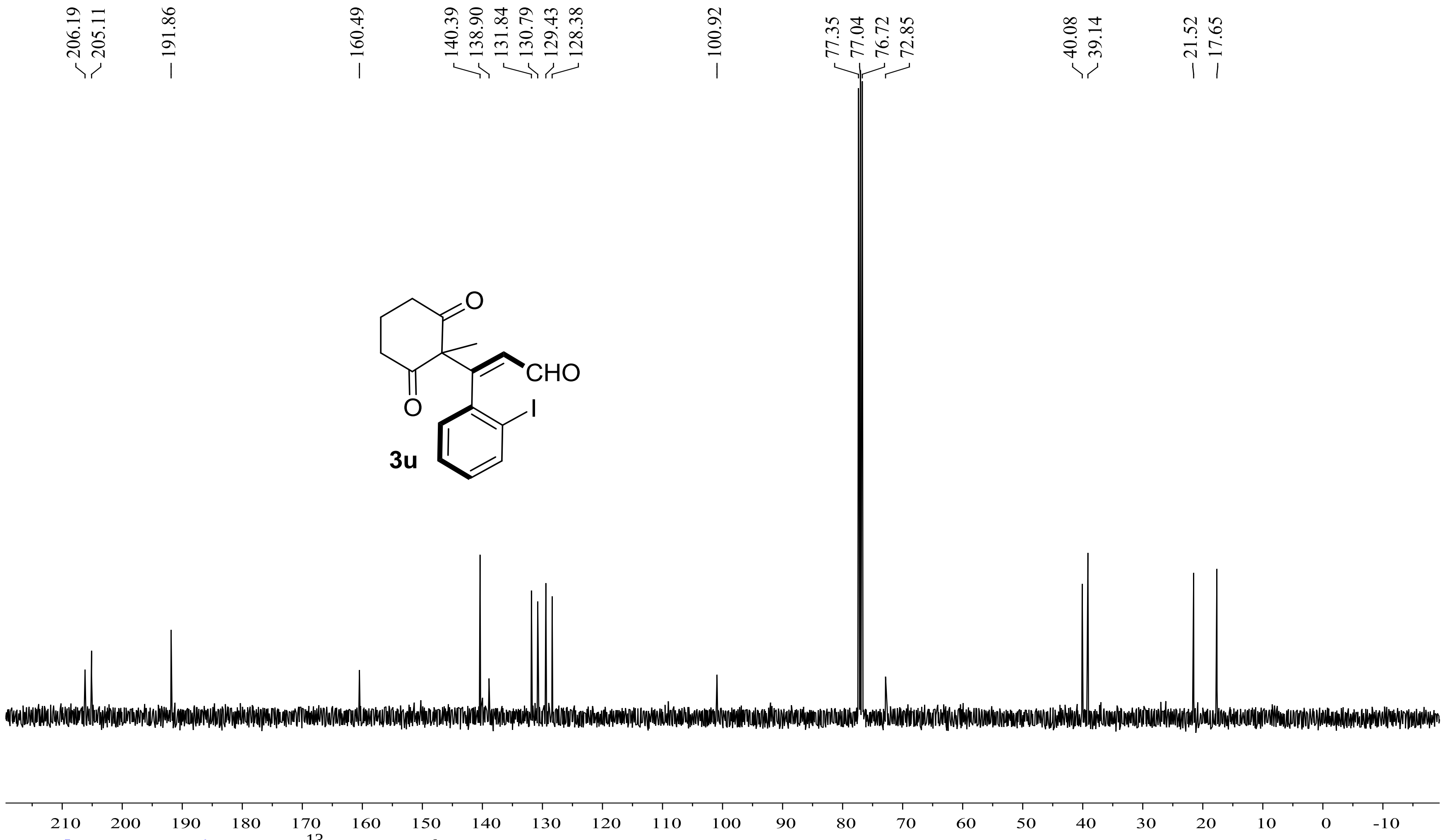

Supplementary Figure 70. ^{13}C NMR of **3u**

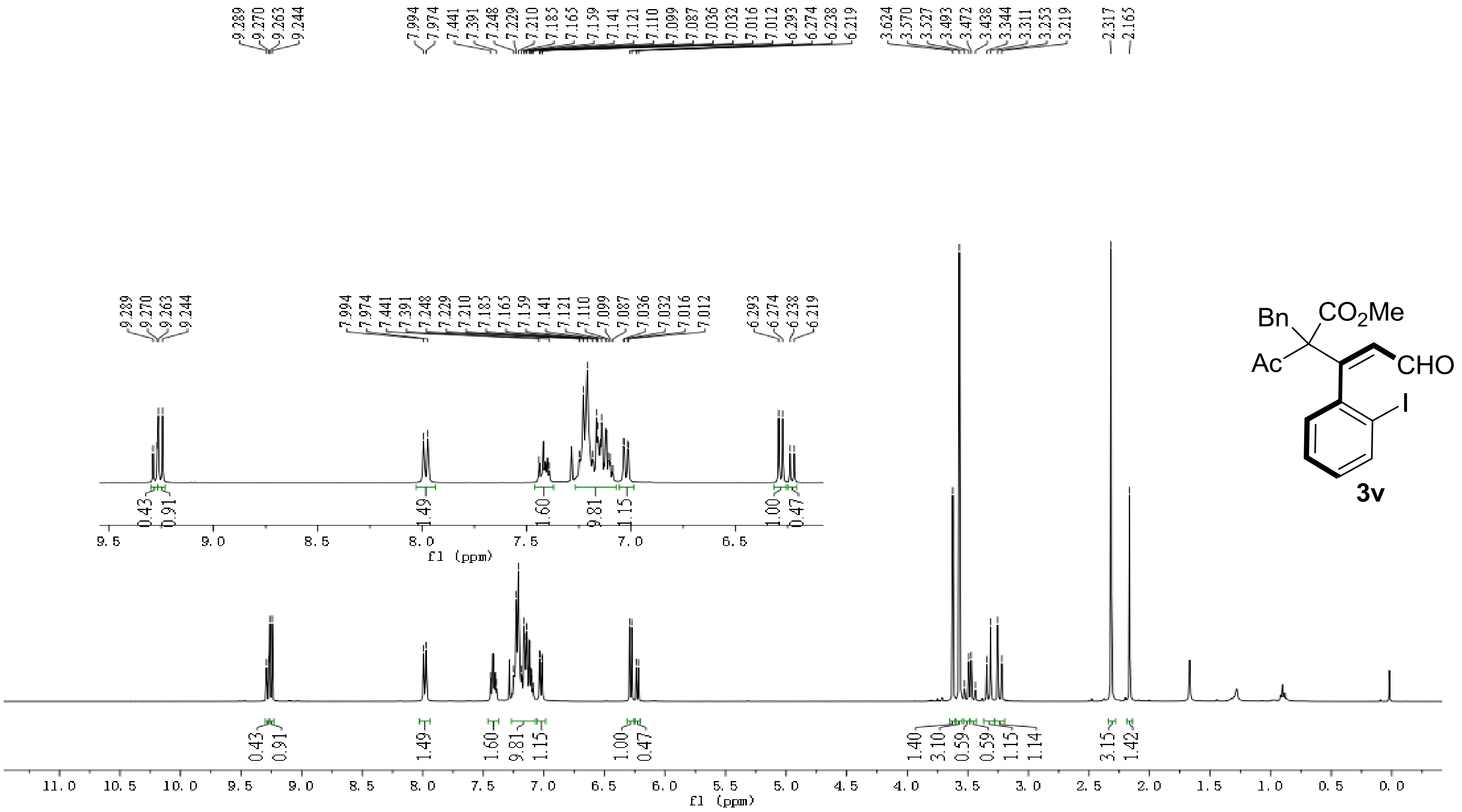

Supplementary Figure 71. ^{1}H NMR of **3v**

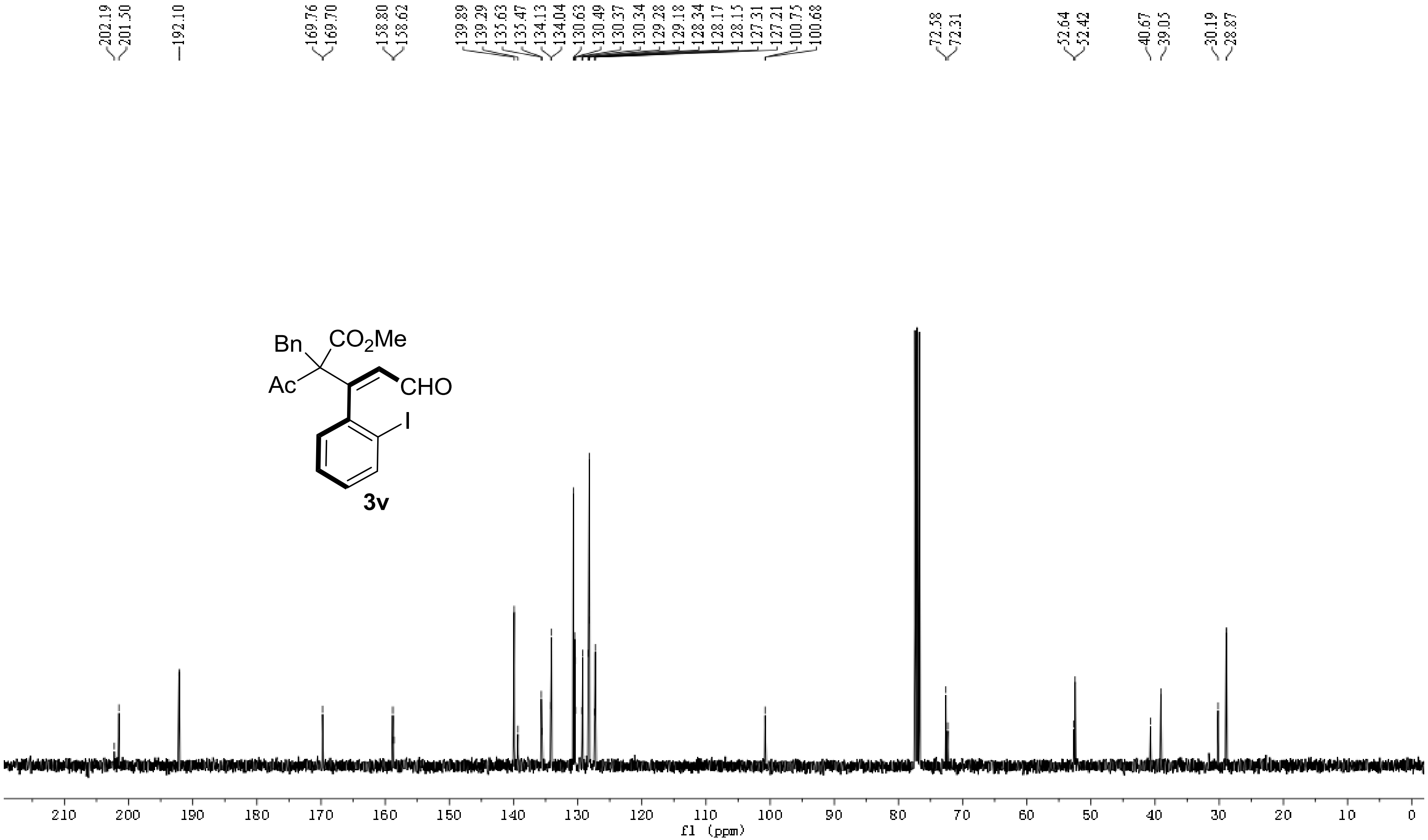

Supplementary Figure 72. ^{13}C NMR of **3v**

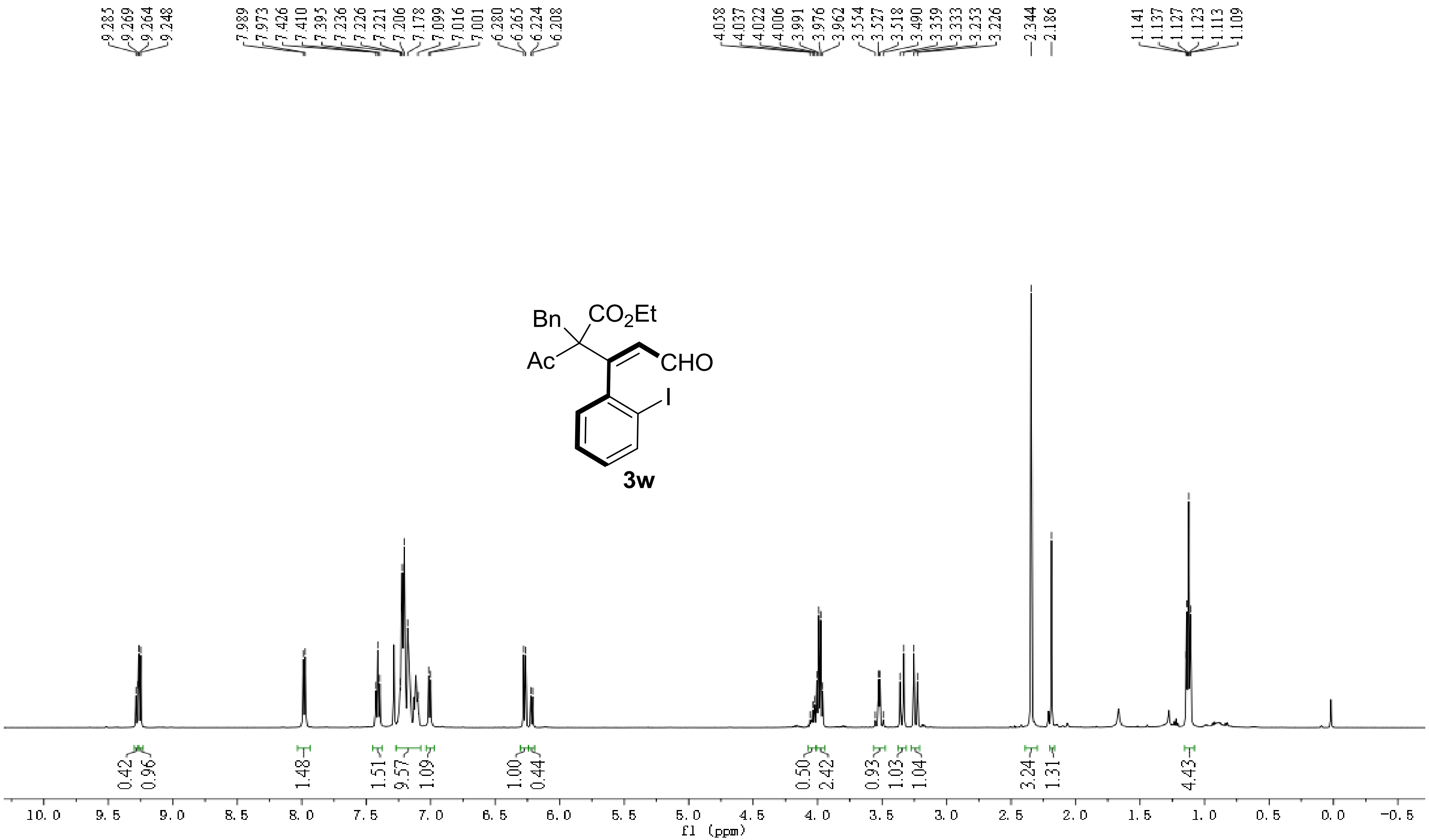

Supplementary Figure 73. ^{1}H NMR of **3w**

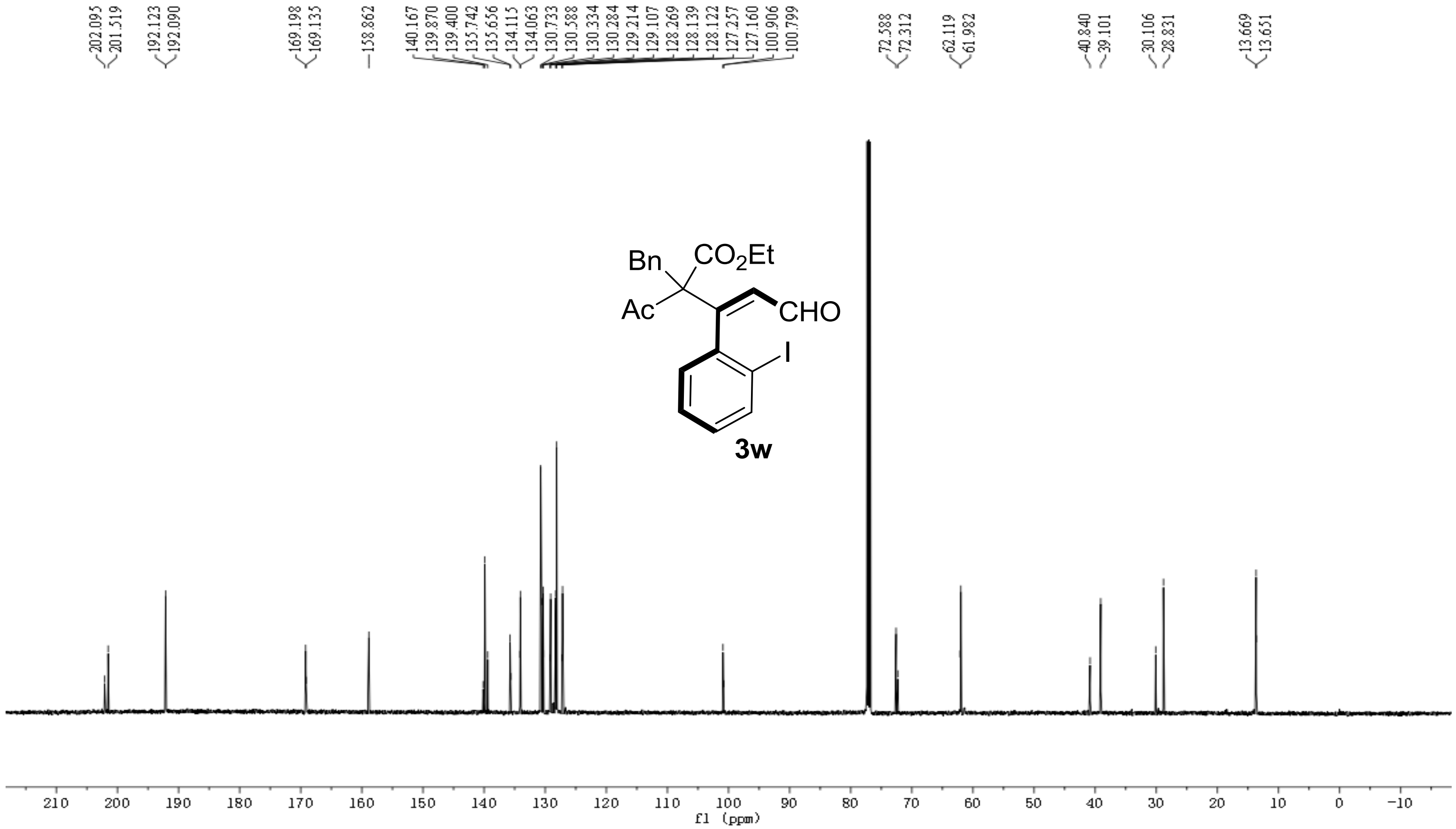

Supplementary Figure 74. ^{13}C NMR of **3w**

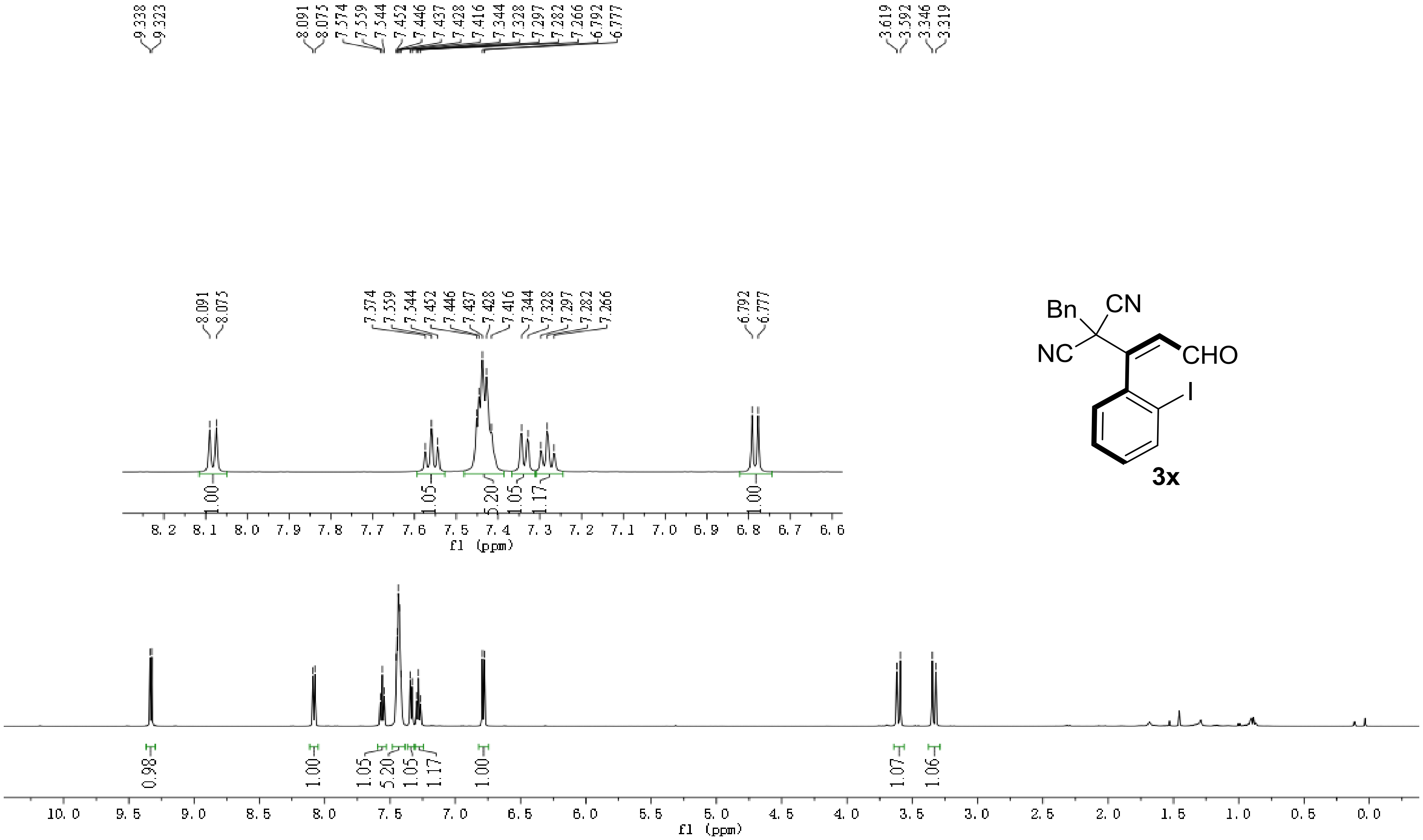

Supplementary Figure 75. ^{1}H NMR of **3x**

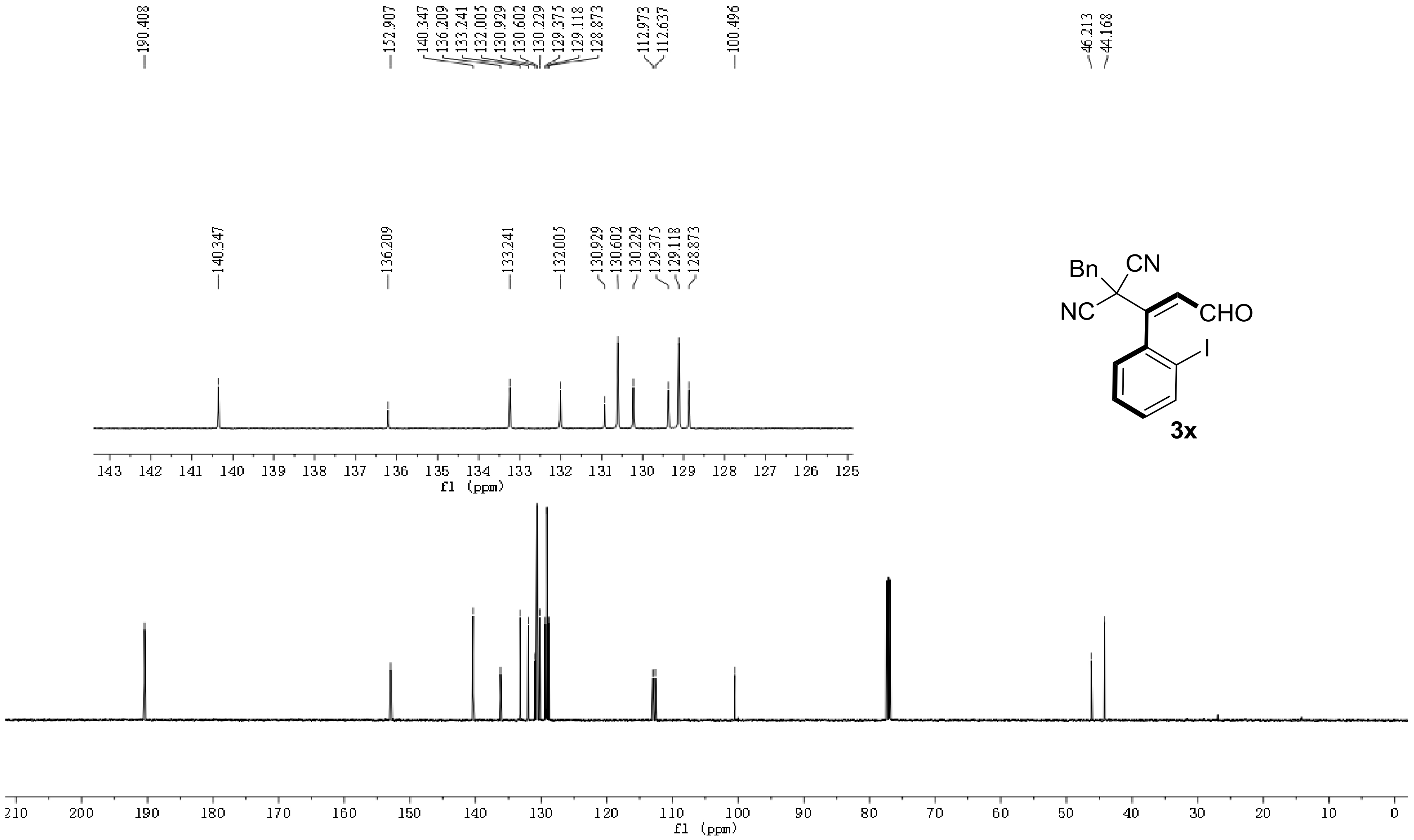

Supplementary Figure 76. ^{13}C NMR of **3x**

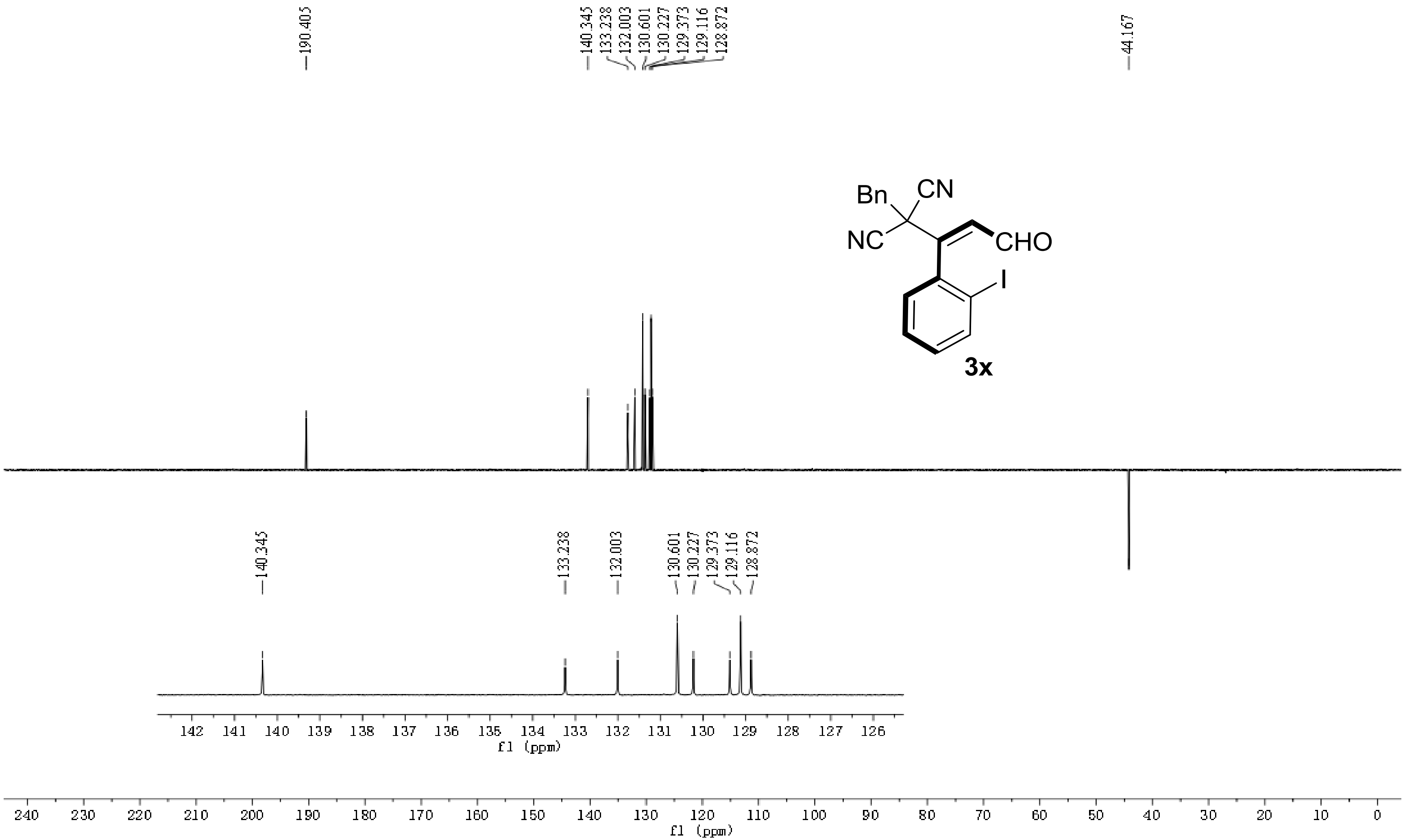

Supplementary Figure 77. DEPT-135 of **3x**

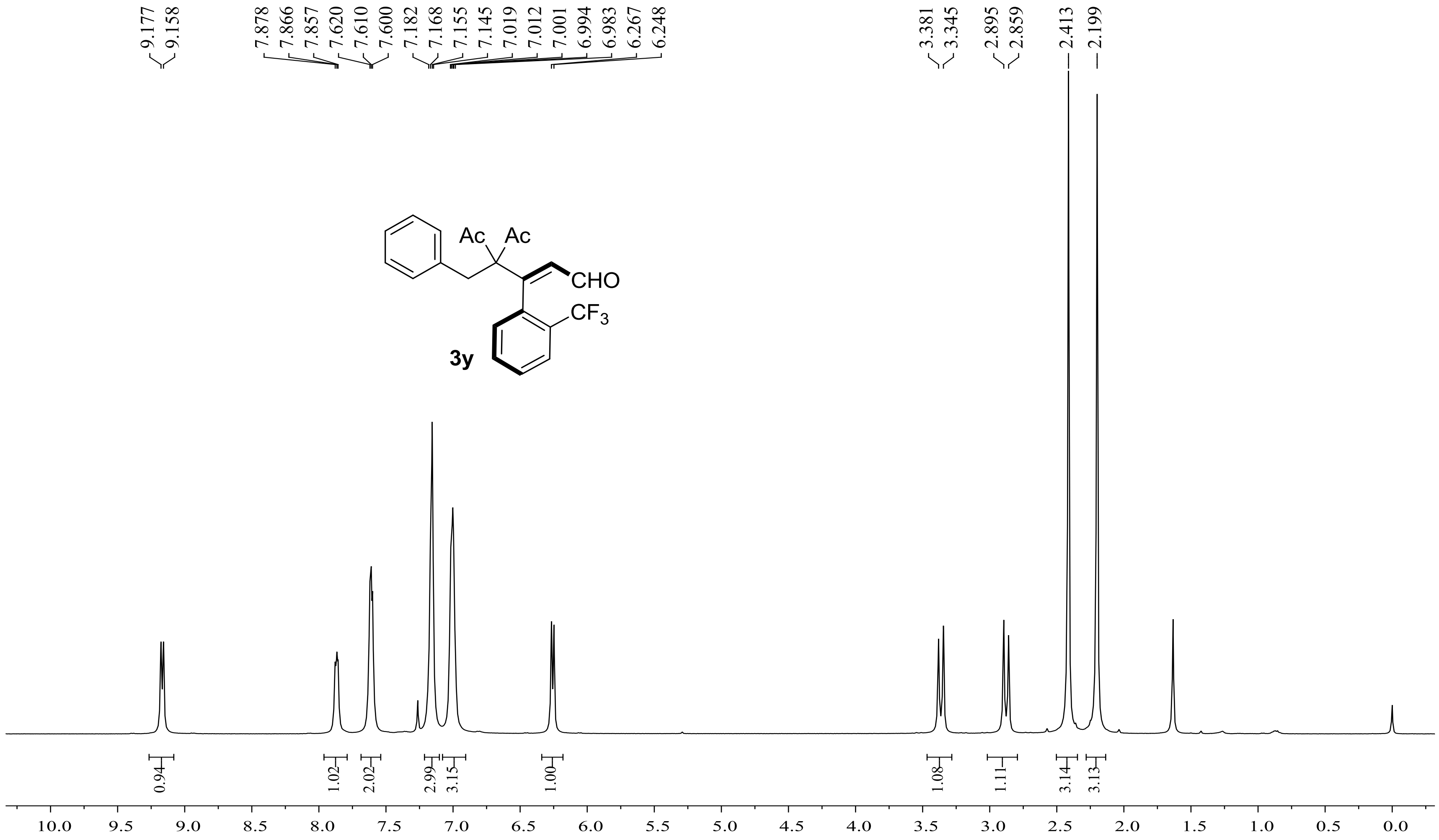

Supplementary Figure 78. ^{1}H NMR of **3y**

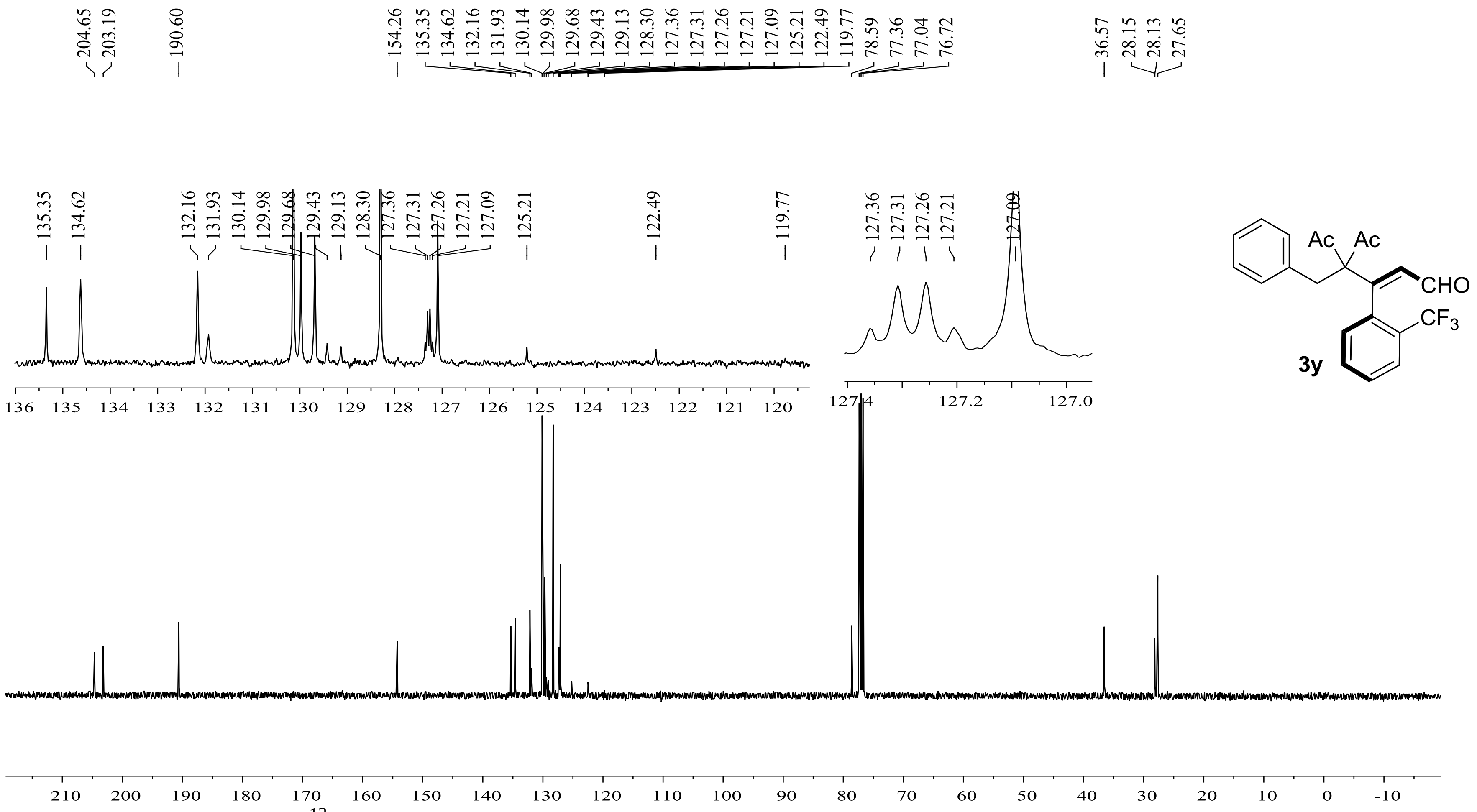

Supplementary Figure 79. ^{13}C NMR of **3y**

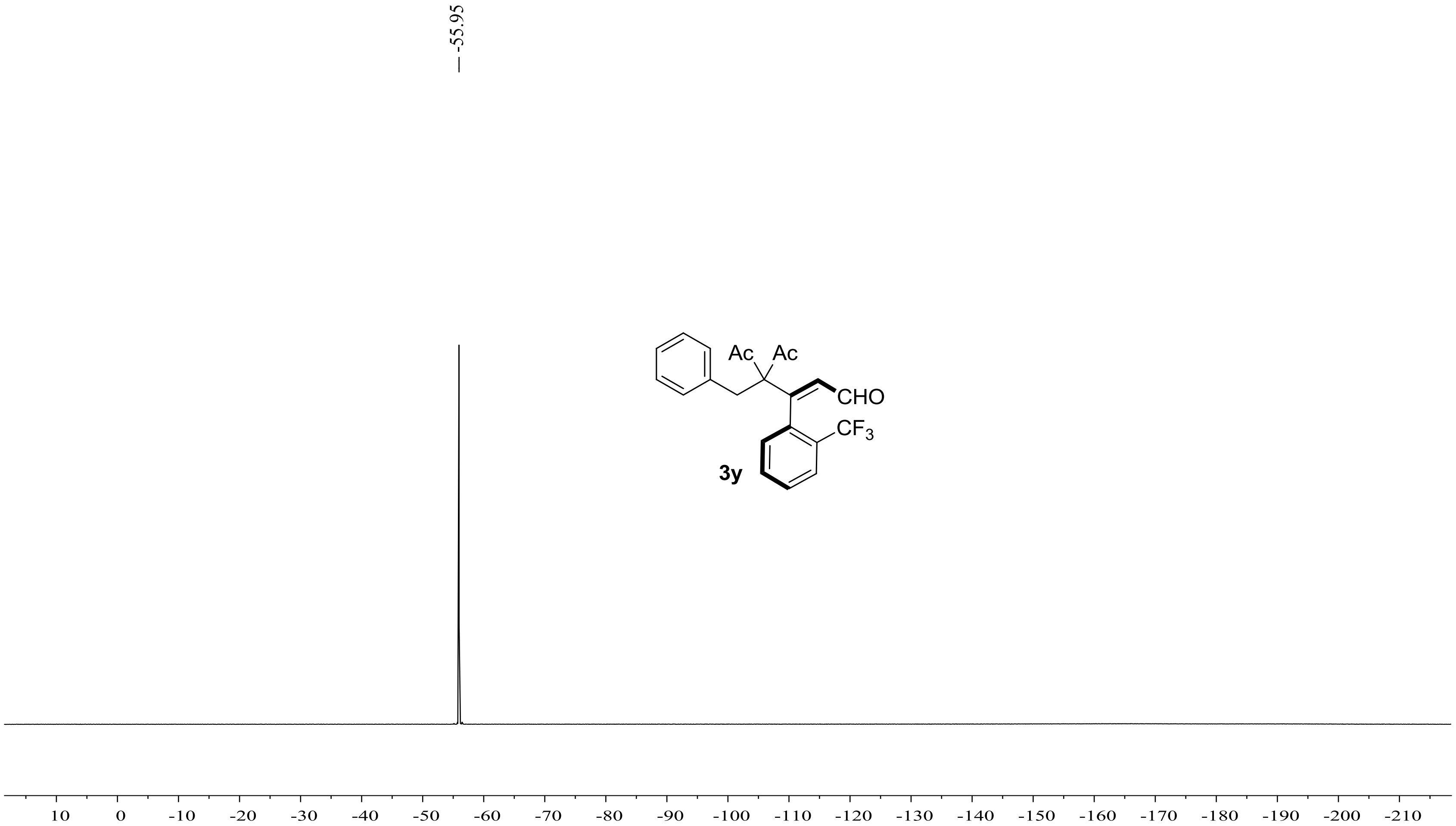

Supplementary Figure 80. ^{19}F NMR of **3y**

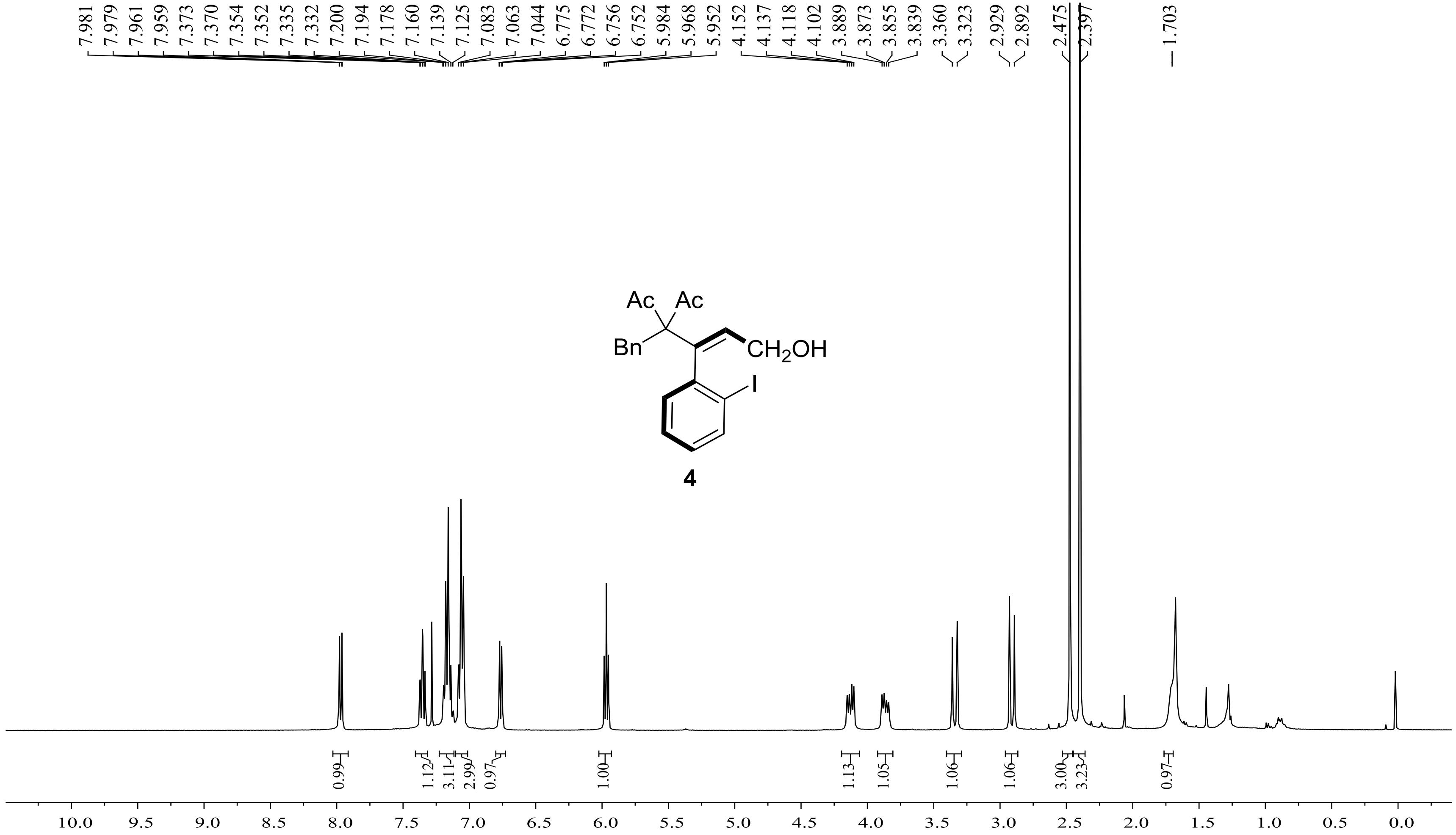

Supplementary Figure 81. ^{1}H NMR of **4**

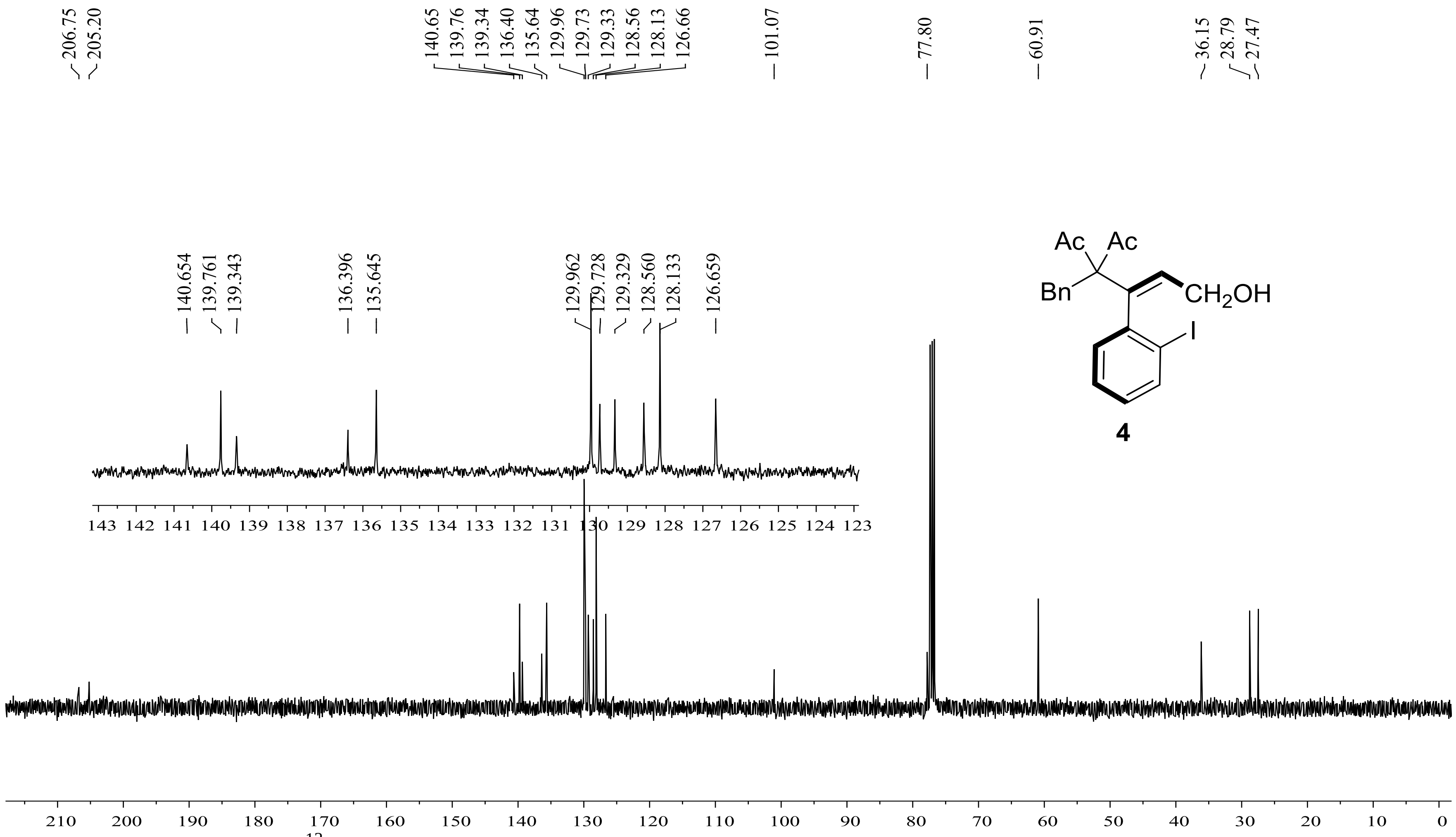

Supplementary Figure 82. ^{13}C NMR of **4**

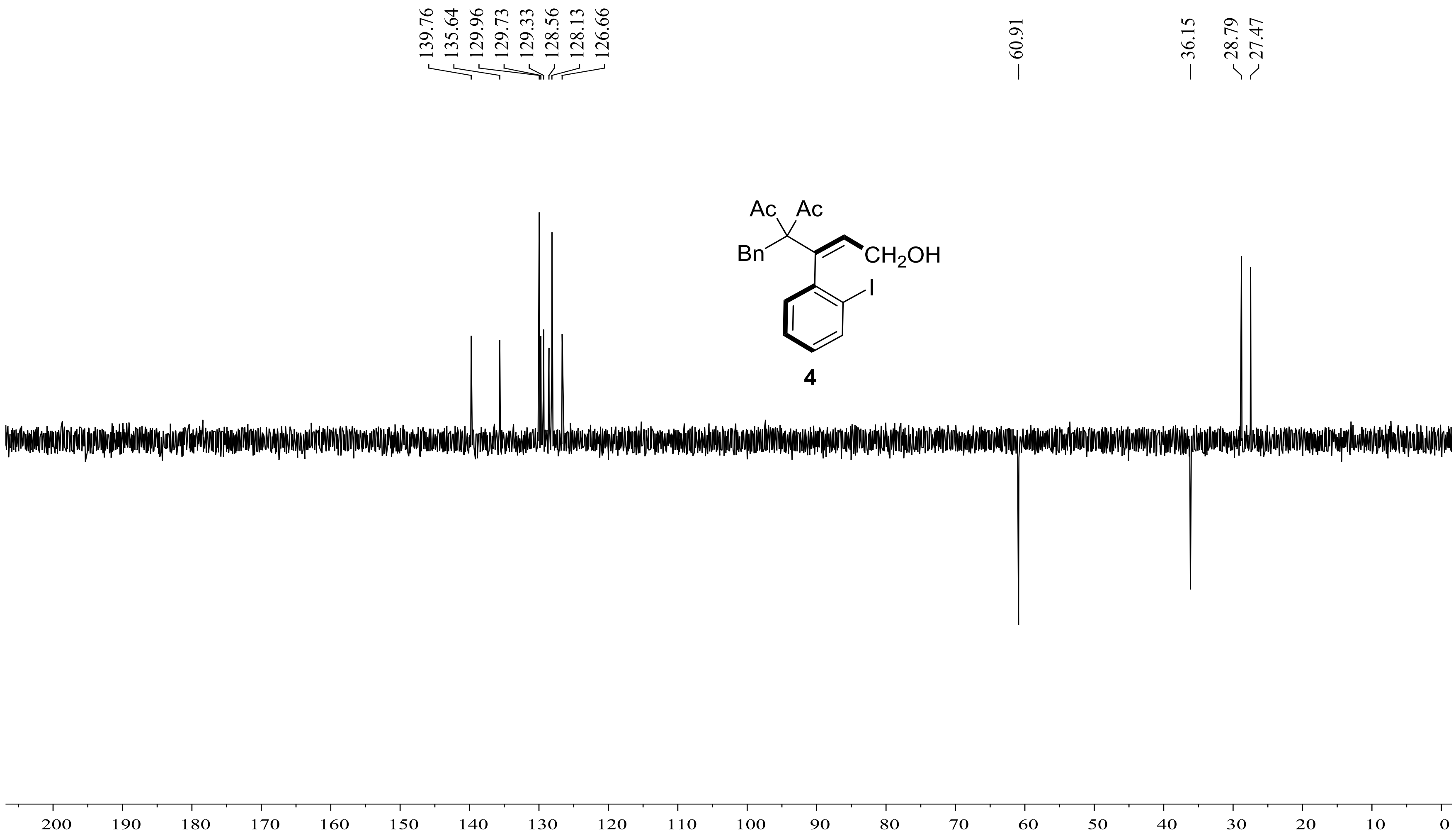

Supplementary Figure 83. DEPT-135 of **4**

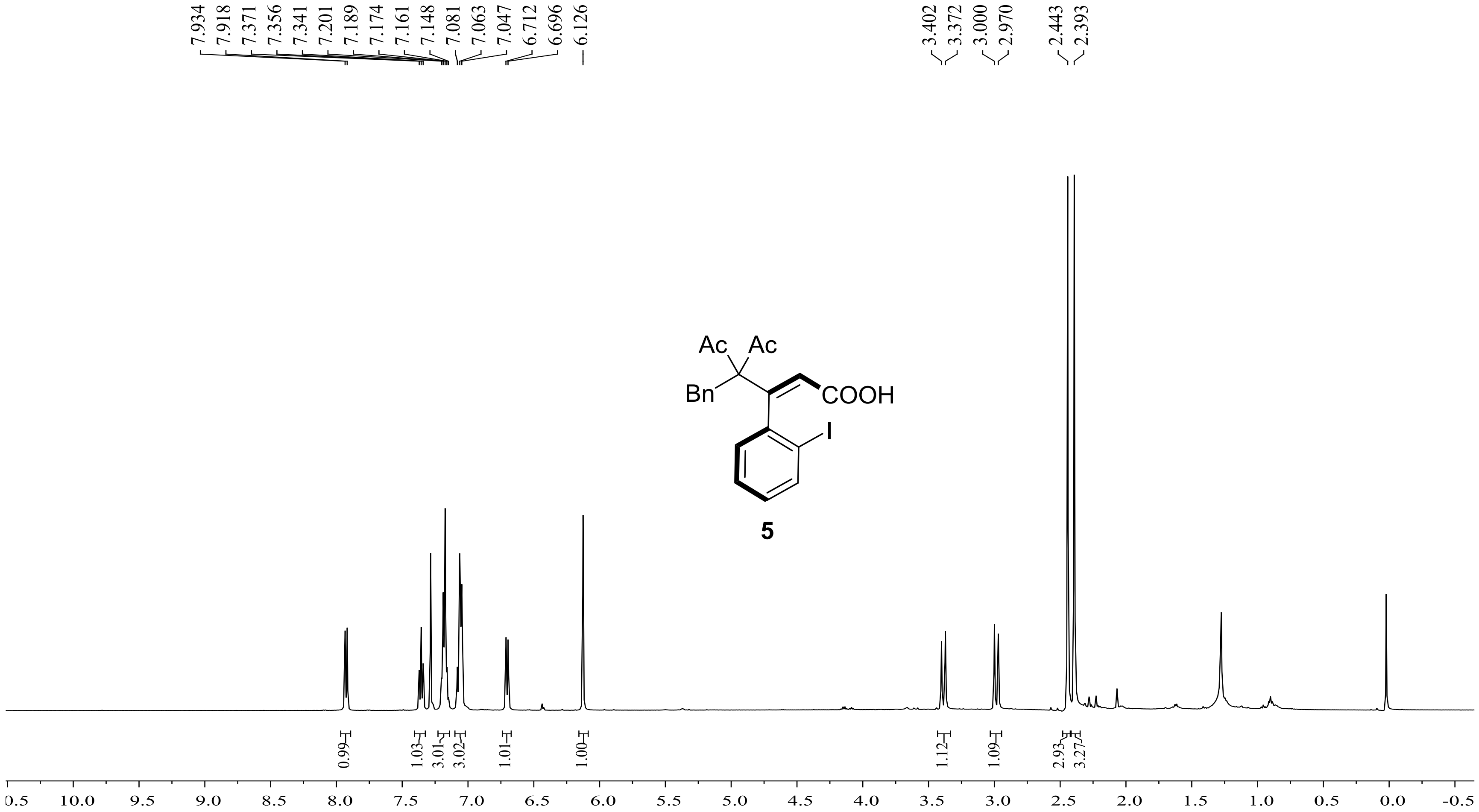

Supplementary Figure 84. ^{1}H NMR of **5**

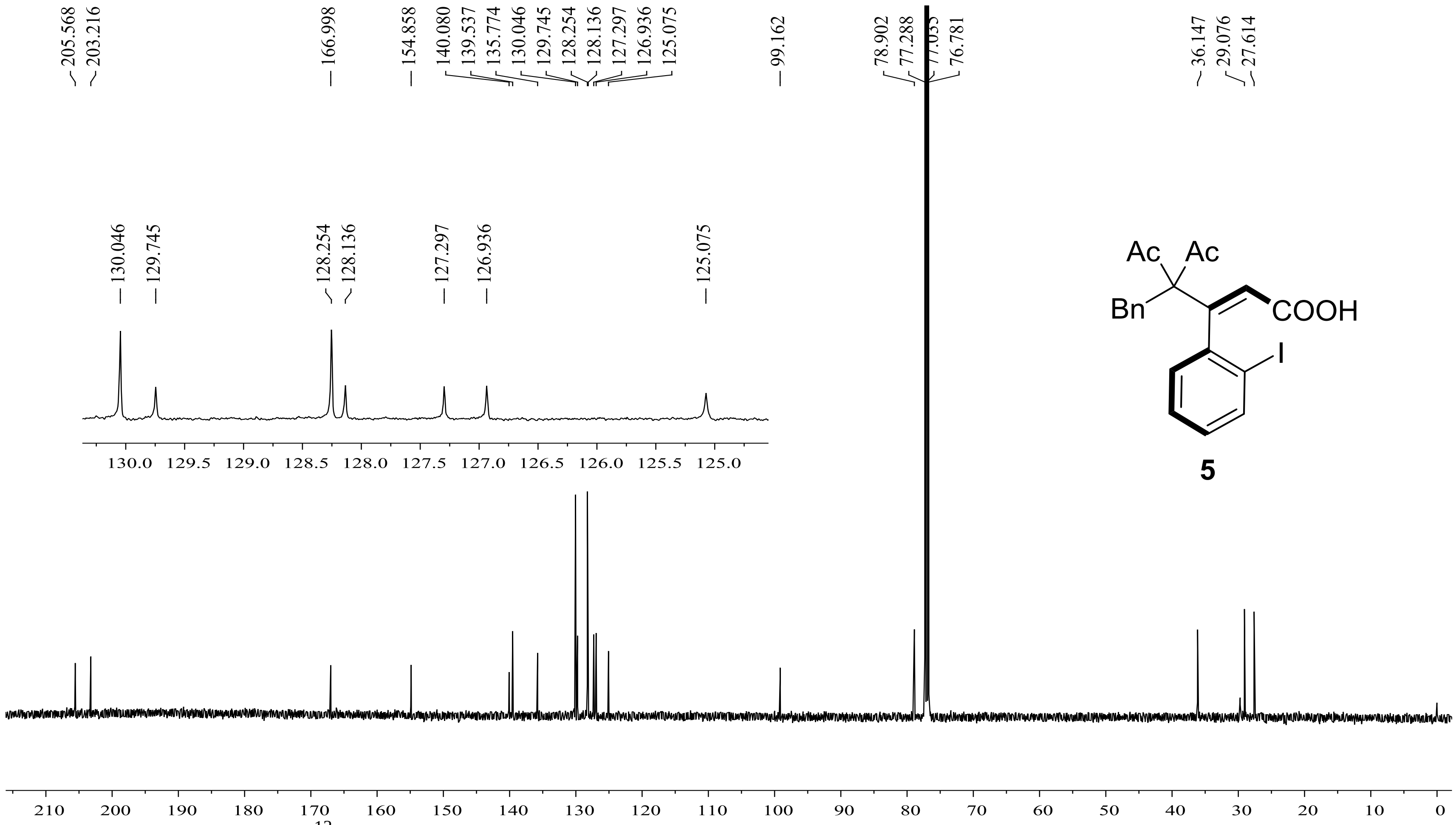

5

Supplementary Figure 85. ^{13}C NMR of **5**

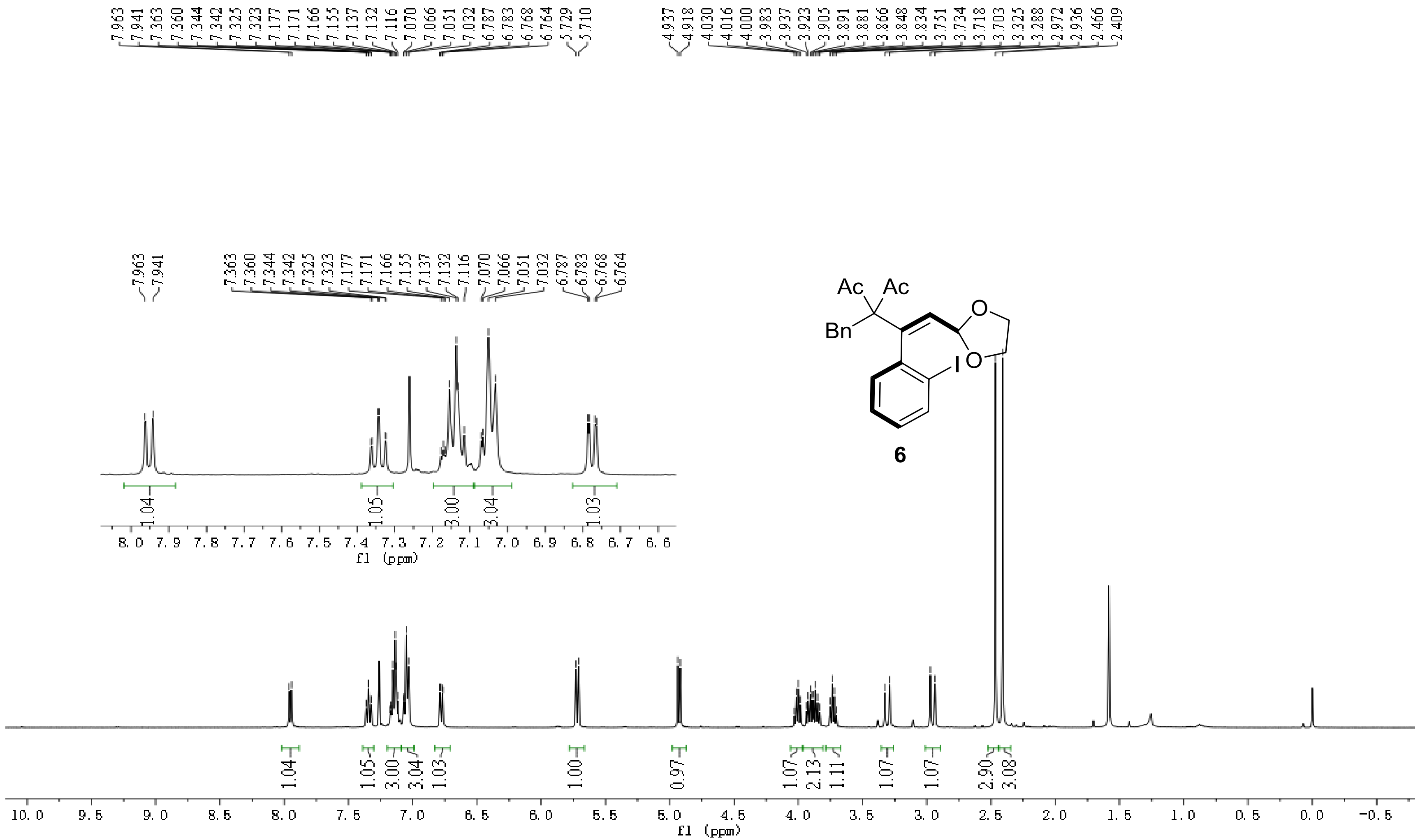

Supplementary Figure 86. ^{1}H NMR of **6**

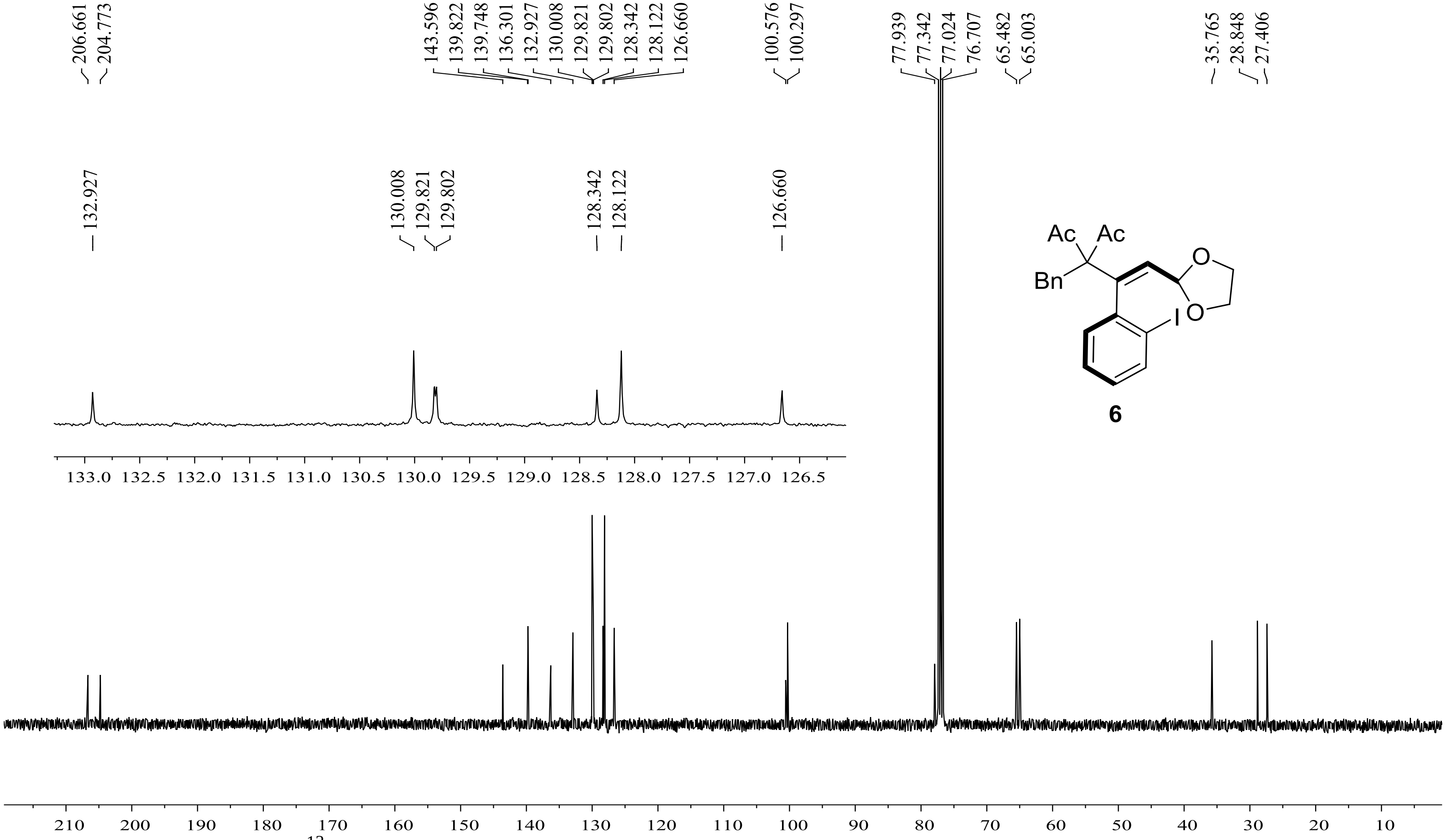

Supplementary Figure 87. ^{13}C NMR of **6**

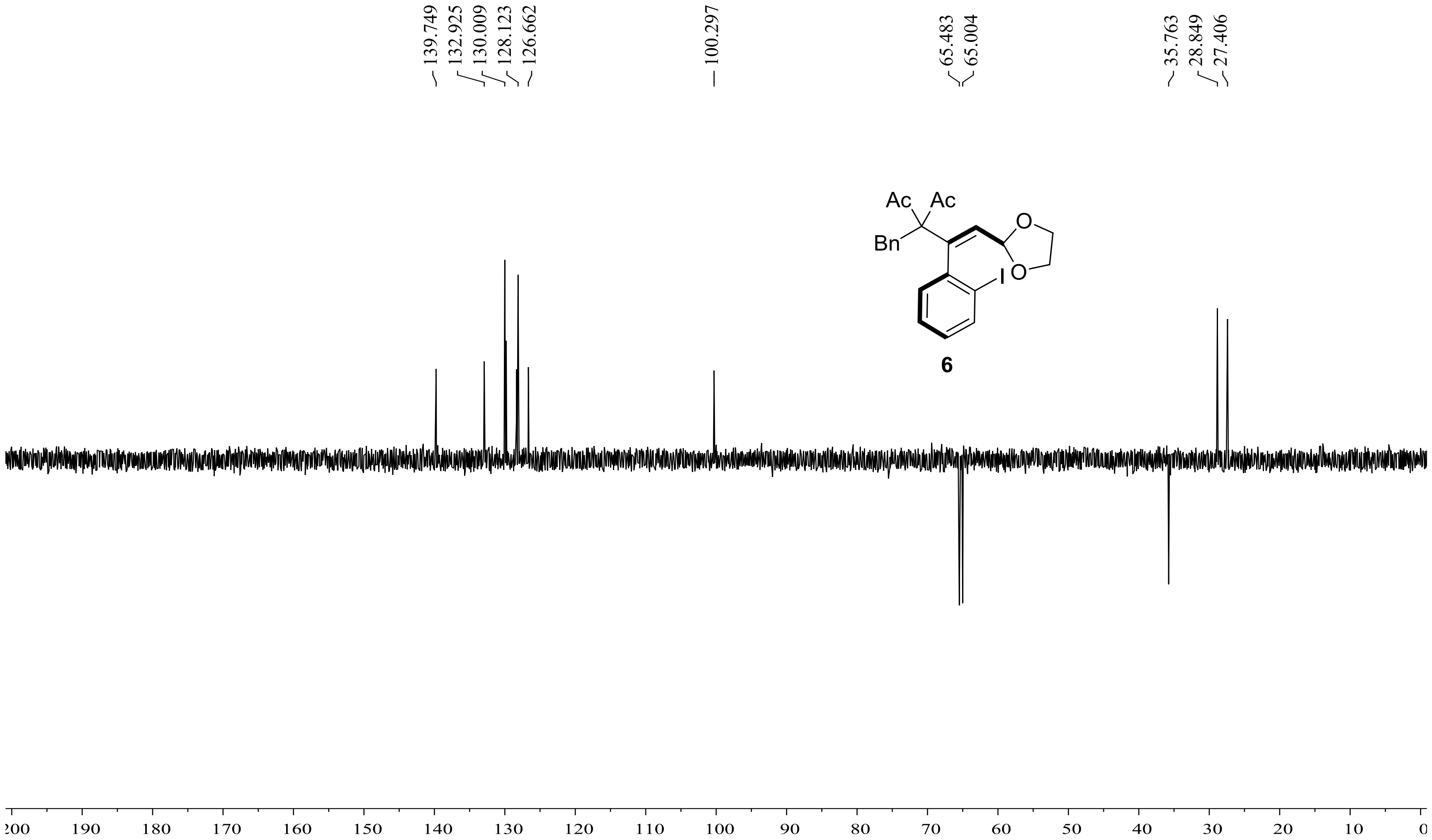

6

Supplementary Figure 88. DEPT-135 of **6**

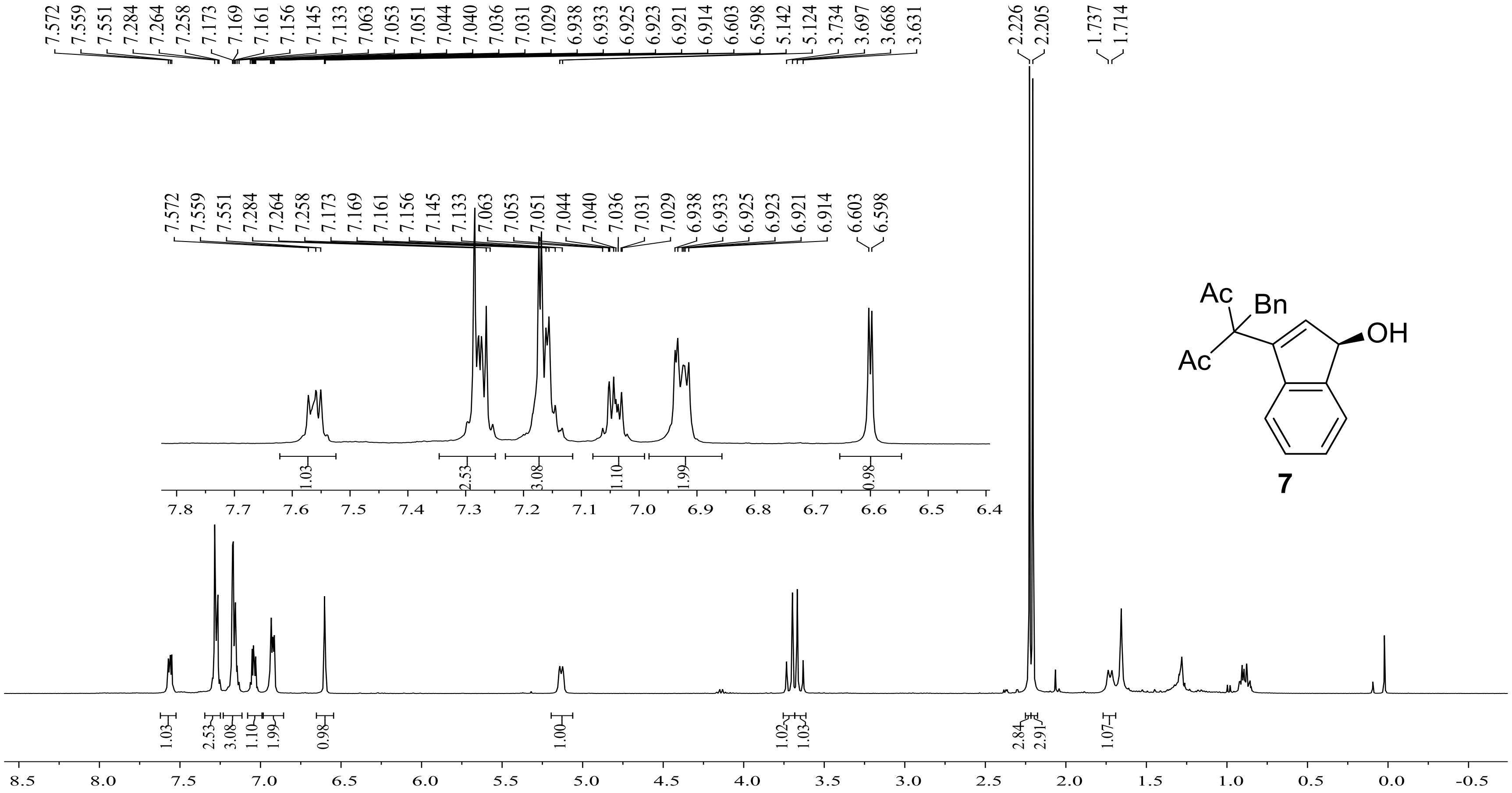

Supplementary Figure 89. ^{1}H NMR of **7**

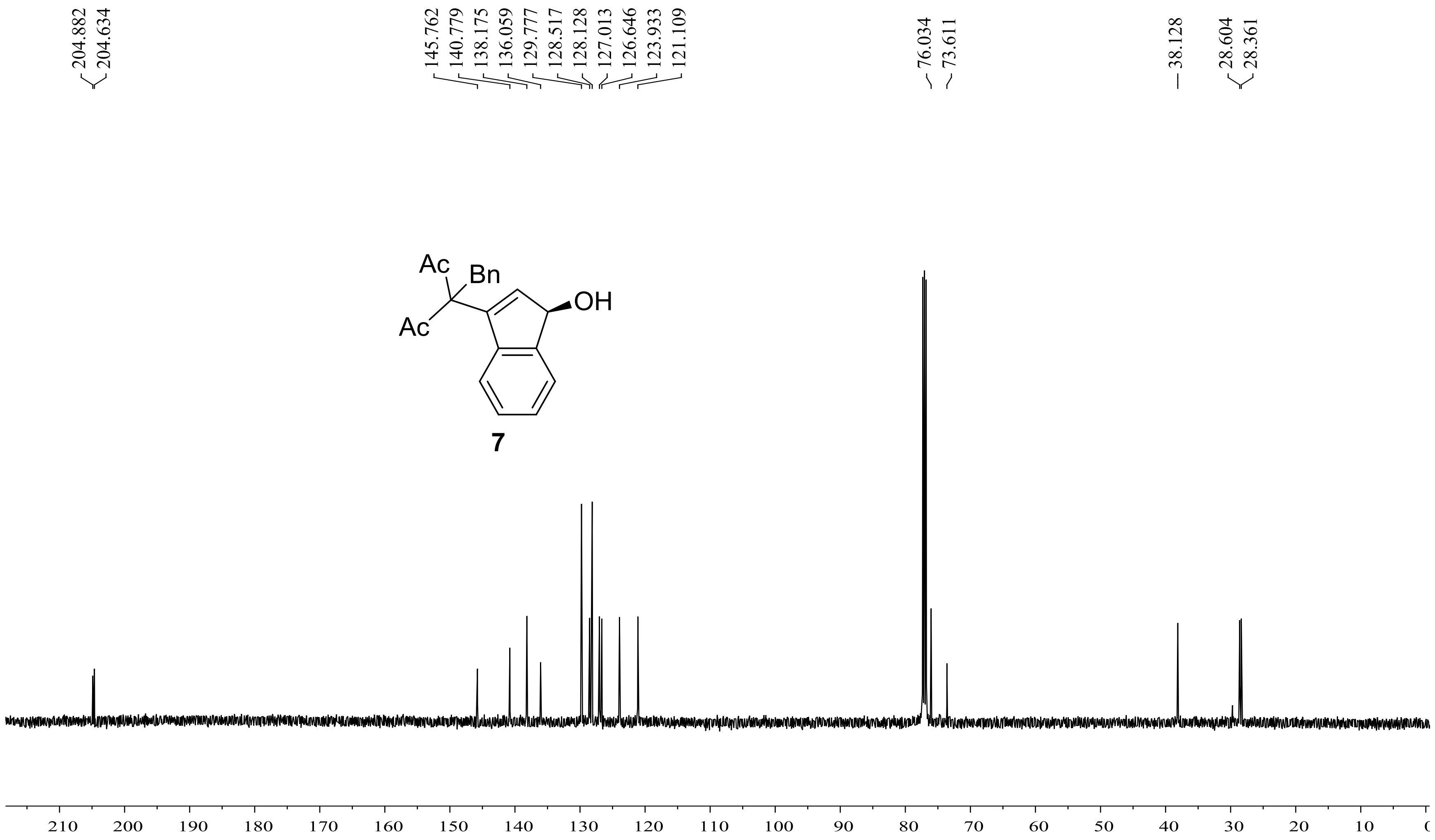

7

Supplementary Figure 90. ^{13}C NMR of **7**

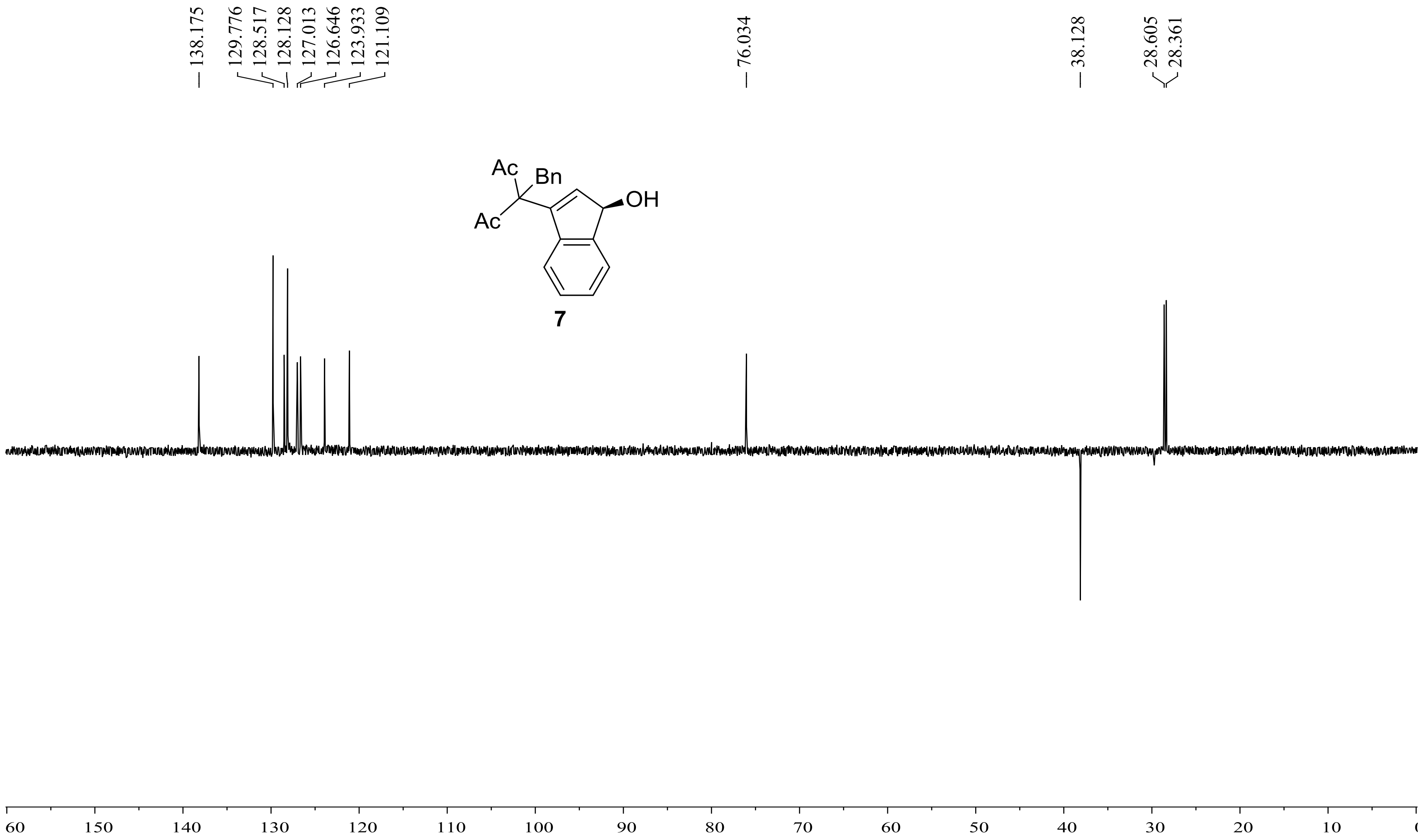

Supplementary Figure 91. DEPT-135 of **7**

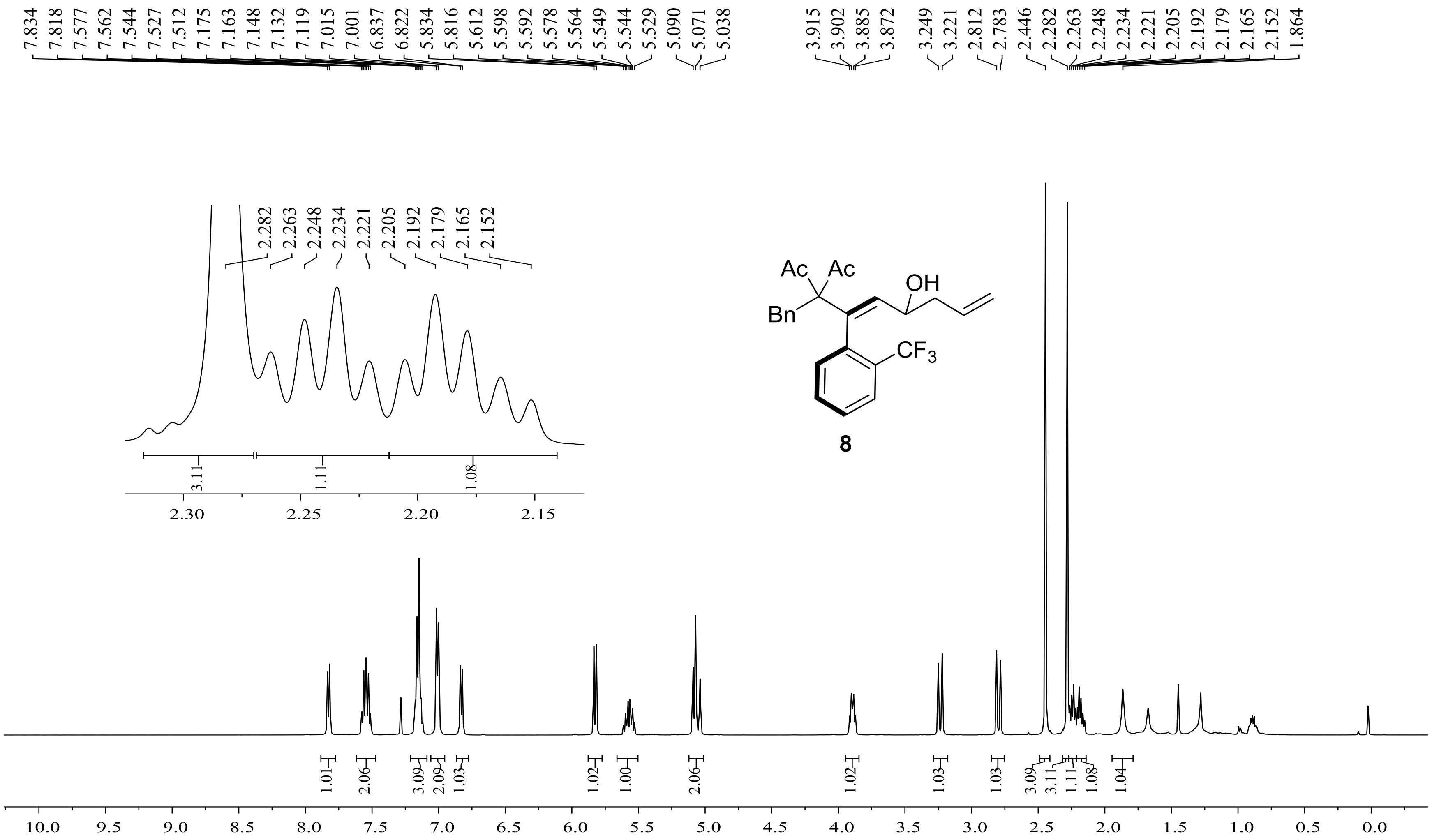

Supplementary Figure 92. ^{1}H NMR of **8**

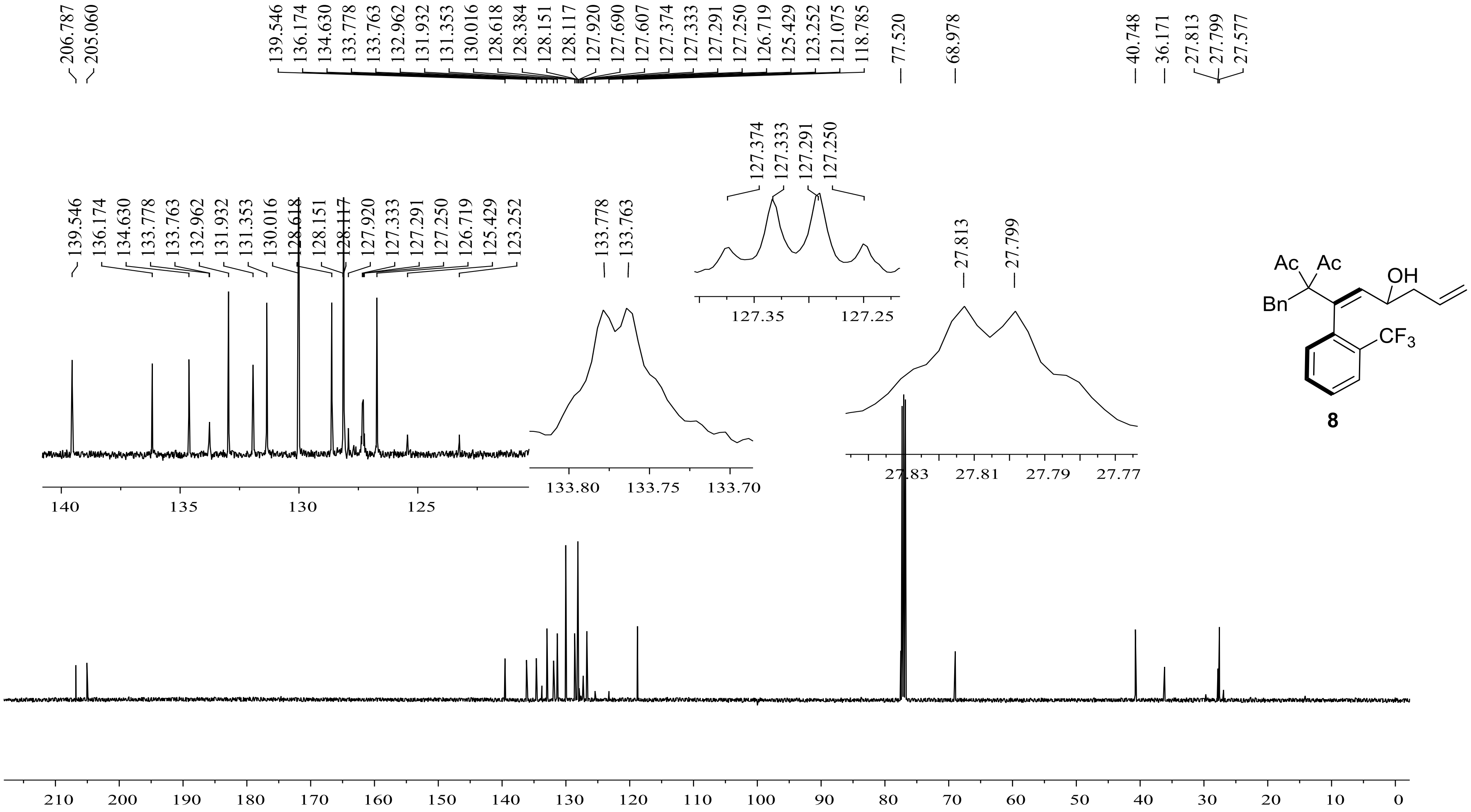

8

Supplementary Figure 93. ^{13}C NMR of **8**

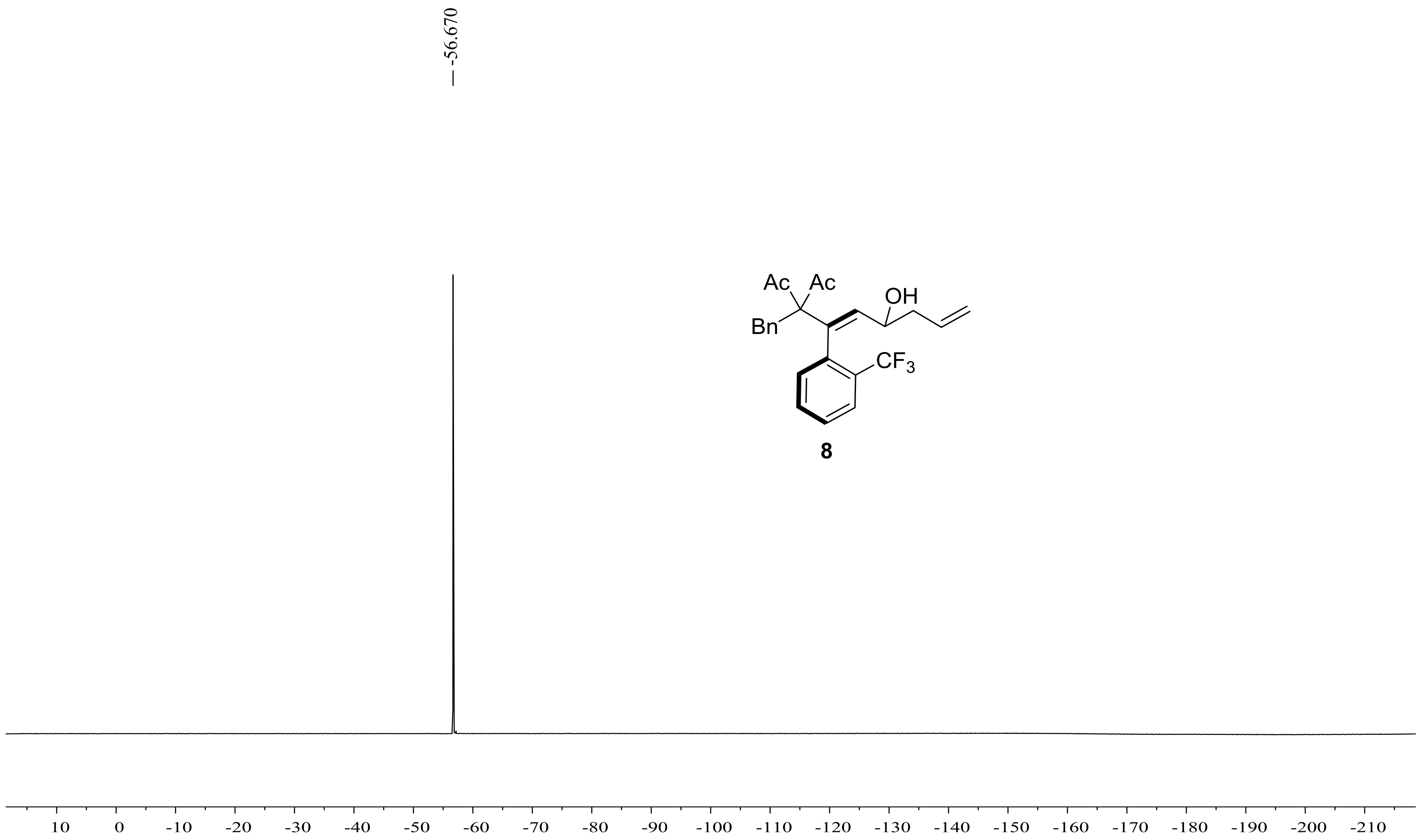

Supplementary Figure 94. ^{19}F NMR of **8**

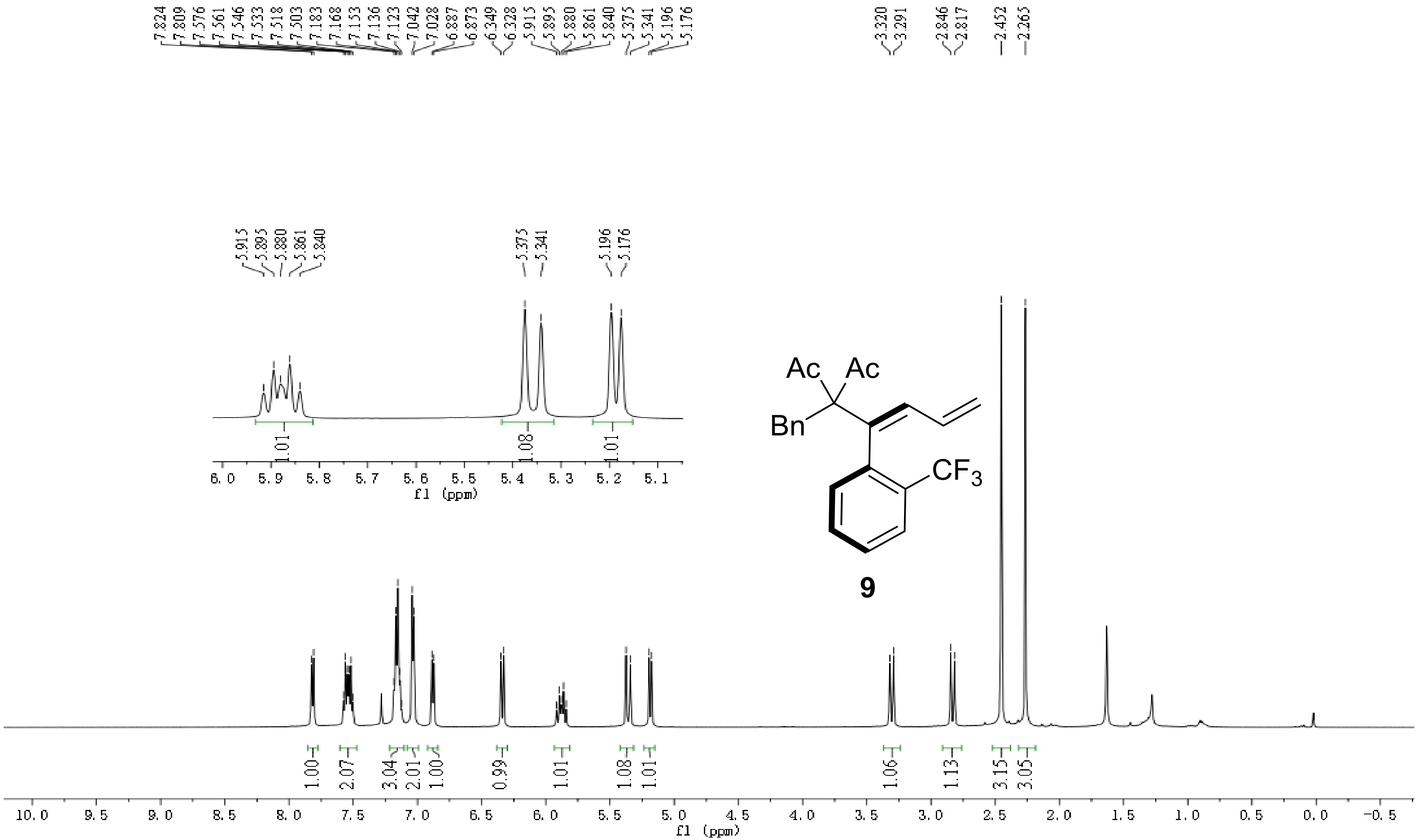

Supplementary Figure 95. ^{1}H NMR of **9**

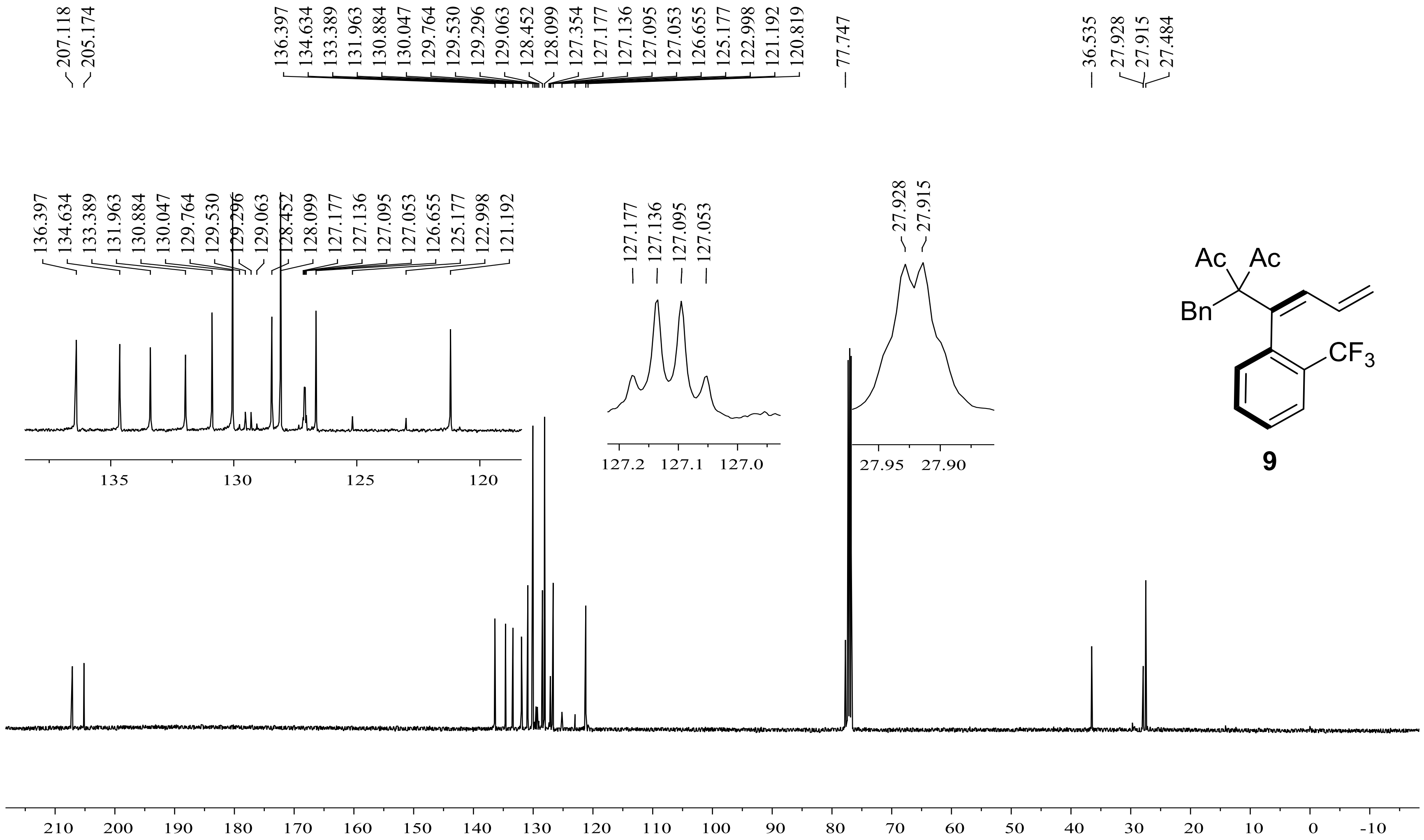

Supplementary Figure 96. ^{13}C NMR of **9**

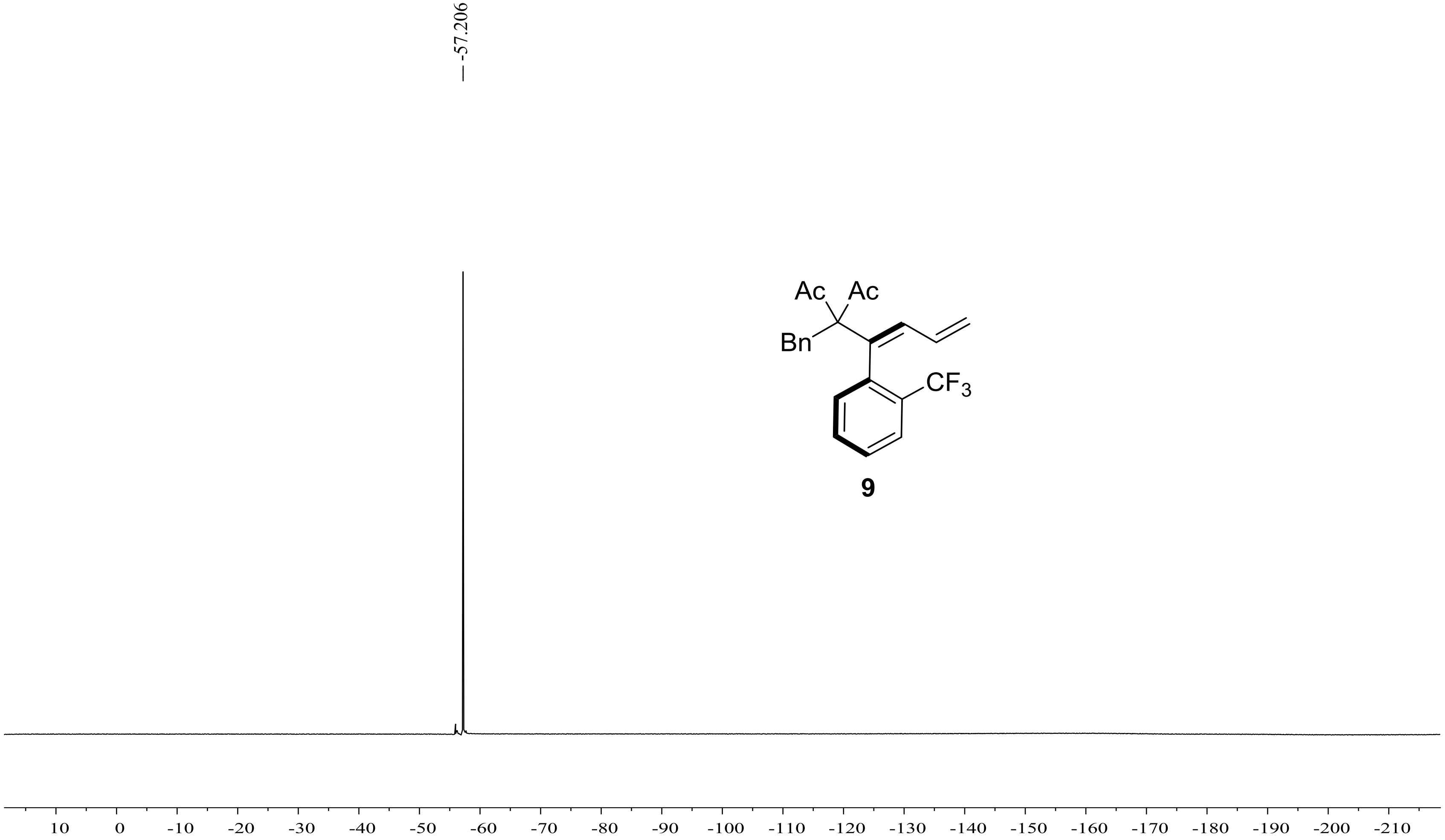

Supplementary Figure 97. ^{19}F NMR of **9**

Supplementary Tables (Optimization of reaction conditions)

Supplementary Table 1.

Organocatalysts screening[a]

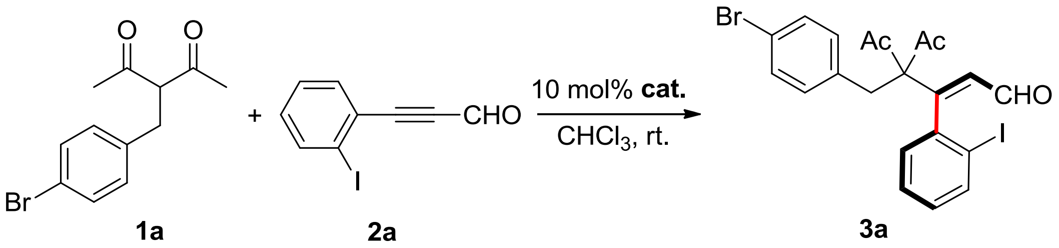

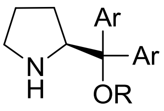

cat.
(**C1-C7**)

C1: R = TMS, Ar = Ph
C2: R = TMS, Ar = 3,5-$(CF_3)_2$-C_6H_3
C3: R = TMS, Ar = 3,5-$(CH_3)_2$-C_6H_3
C4: R = TBS, Ar = 3,5-$(CH_3)_2$-C_6H_3
C5: R = TIPS, Ar = 3,5-$(CH_3)_2$-C_6H_3
C6: R = TBS, Ar = Ph
C7: R = TIPS, Ar = Ph

entry	**catalyst**	T (ºC)	solvent	yield (%)[b]	ee (%) [c]
1	**C1**	rt	$CHCl_3$	74	65
2	**C2**	rt	$CHCl_3$	trace	ND[d]
3	**C3**	rt	$CHCl_3$	93	65
4	**C4**	rt	$CHCl_3$	97	83
5	**C5**	rt	$CHCl_3$	54	90
6	**C6**	rt	$CHCl_3$	90	85
7	**C7**	rt	$CHCl_3$	54	86

[a] All reactions were performed by using **1a** (0.05 mmol), **2a** (0.055 mmol, 1.1 equiv), catalyst (10 mol%) and solvent (0.5 mL) at room temperature (rt.) for 24 hours. [b] Isolated yield. [c] Determined by chiral stationary HPLC. [d] Not determined.

Supplementary Table 2.

Solvent effect[a]

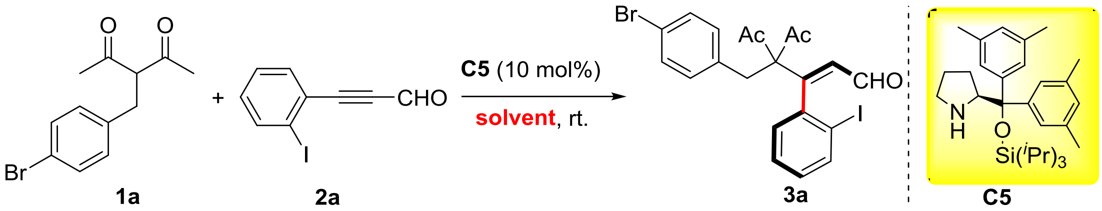

entry	cat.	T(ºC)	solvent	isolated yield (%)[b]	ee [c]
1	**C5**	rt	$CHCl_3$	54	90
2	**C5**	rt	THF	87	90
3	**C5**	rt	Tol	78	88
4	**C5**	rt	CH_3CN	51	84
5	**C5**	rt	Et_2O	70	90
6	**C5**	rt	EA	85	90
7	**C5**	rt	DCM	95	90

[a] All reactions were performed by using **1a** (0.05 mmol), **2a** (0.055 mmol, 1.1 equiv), catalyst (10 mol%) and solvent (0.5 mL) at room temperature (rt.) for 24 hours. [b] Isolated yield. [c] Determined by chiral stationary HPLC.

Supplementary Table 3.

Catalyst loading and reactant molar ratio effect [a]

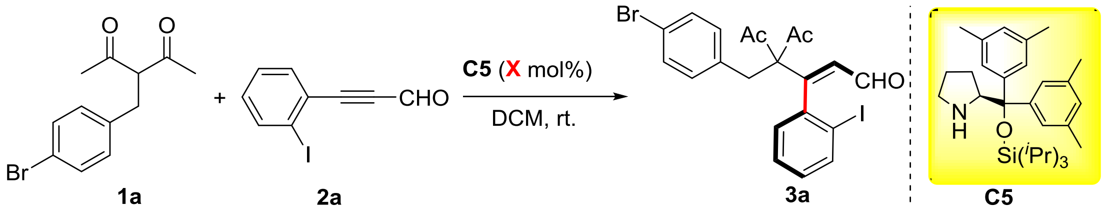

entry	catalyst loading (X mol%)	ratio（1a： 2a）	isolated yield (%)[b]	ee (%) [c]
1	10%	1.5 : 1	93	89
2	10%	1 : 1.5	92	90
3	10%	1: 1.1	95	90
4	20%	1: 1.1	88	89
5	5%	1: 1.1	94	90

[a] All reactions were performed at room temperature (rt.) for 24 hours and solvent (0.5 mL) [b] Isolated yield. [c] Determined by chiral stationary HPLC.

Supplementary Table 4.

Temperature and additive effect[a]

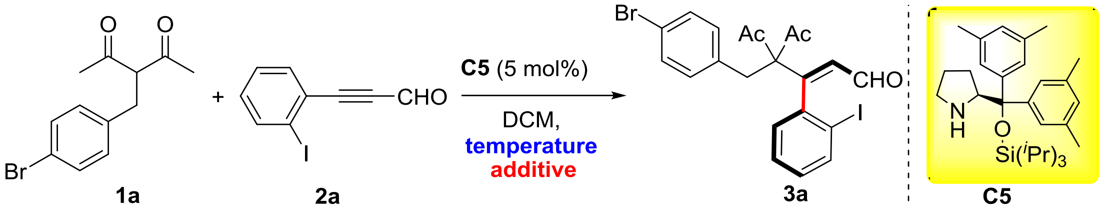

entry	catalyst	T (ºC)	additive	yield (%)[b]	ee (%) [c]
1	**C5** (5 mol%)	rt	-	94	90
2	**C5** (5 mol%)	0	-	55	94
3	**C5** (5 mol%)	-20	-	trace	ND[d]
4	C5 (5 mol%)	0	LiOAc	96	94
**5**	**C5** (5 mol%)	0	CsOAc	93	94
**6**	**C5** (5 mol%)	0	^{t}BuCO$_2$Cs	90	94

[a] All reactions were performed by using **1a** (0.05 mmol), **2a** (0.055 mmol, 1.1 equiv), catalyst (5 mol%) and solvent (0.5 mL) for 24 hours. [b] Isolated yield. [c] Determined by chiral stationary HPLC. [d] Not determined.

Supplementary Table 5.

Crystal data and structure refinement for 3n.

Identification code	**3n**
Empirical formula	$C_{21}H_{19}IO_3$
Formula weight	446.26
Temperature/K	100
Crystal system	orthorhombic
Space group	$P2_12_12_1$
a/Å	11.5300(5)
b/Å	12.6720(5)
c/Å	12.6749(5)
α/°	90
β/°	90
γ/°	90
Volume/Å^3	1851.91(13)
Z	4
ρ_{calc}g/cm^3	1.601
μ/mm^{-1}	13.718
F(000)	888.0
Crystal size/mm^3	0.25 × 0.23 × 0.2
Radiation	CuKα (λ = 1.54178)
2Θ range for data collection/°	9.87 to 134.76
Index ranges	$-13 \leq h \leq 9$, $-15 \leq k \leq 13$, $-15 \leq l \leq 14$
Reflections collected	19803
Independent reflections	3278 [R_{int} = 0.0545, R_{sigma} = 0.0301]
Data/restraints/parameters	3278/0/228
Goodness-of-fit on F^2	1.095
Final R indexes [I>=2σ (I)]	R_1 = 0.0230, wR_2 = 0.0603
Final R indexes [all data]	R_1 = 0.0231, wR_2 = 0.0603
Largest diff. peak/hole / e Å^{-3}	0.44/-0.79
Flack parameter	-0.007(3)

Supplementary Note 1

Enantiomerization barrier determination for 3a

The enantiomerisation barrier, corresponding to barrier to rotation for **3a** atropisomers, was obtained by kinetic of racemization of an enantiomer. The slope of the first-order kinetic line gives the racemization constant (k racemization =2 * k enantiomersation). Eyring equation gives the enantiomerisation barrier from enantiomerisation constant (k enantiomerisation), R = 8.31451 J.K^{-1}.mol^{-1}, h = 6.62608 x 10^{-34}J.s and k_B = 1.38066 10^{-23} J.K^{-1}.

A. solvent: Acetone

Temperature = 50 ˚C

Time (min)	% first eluted enantiomer (%t)	ln((%t-50)/ (%t_0 -50))
0	97.0	0
30	96.56	-0.00940579828687615
60	96.3	-0.015005640617870162
90	96.2	-0.01716780362236533
120	96.1	-0.01933465170745584
420	94.6	-0.052413742684040164
810	92.76	-0.09454451555321405

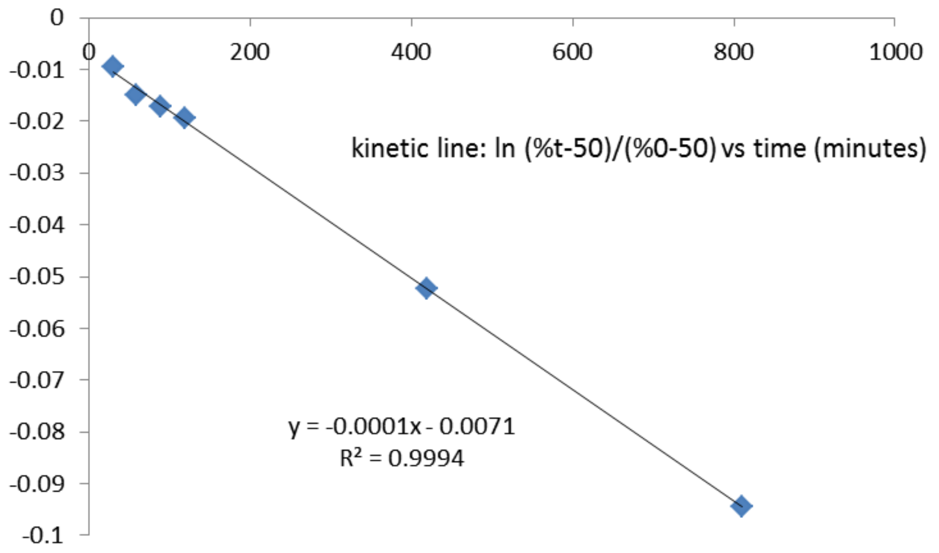

K racemization = 1.6666667 x 10^{-6} S^{-1}

K enantiomerization = 8.3333333 x 10^{-7} S^{-1}

ΔG$^{\neq}$ enantiomerization = 117.0 KJ.mol^{-1}

Half-life time $t_{1/2}$ = 415888 seconds

6931.47 mins

115.5 hours

4.8 days

B. solvent: chloroform

Temperature = 61 ˚C

Time (min)	% first eluted enantiomer (%t)	ln((%t-50)/ (%0-50))
0	96.88	0
30	96.79	-0.00192164039063229
60	95.86	-0.0219978678389987
90	95.66	-0.0263685041671076
120	95.13	-0.0380439314298473
420	90.85	-0.1376843239627452
810	85.58	-0.275807463031068

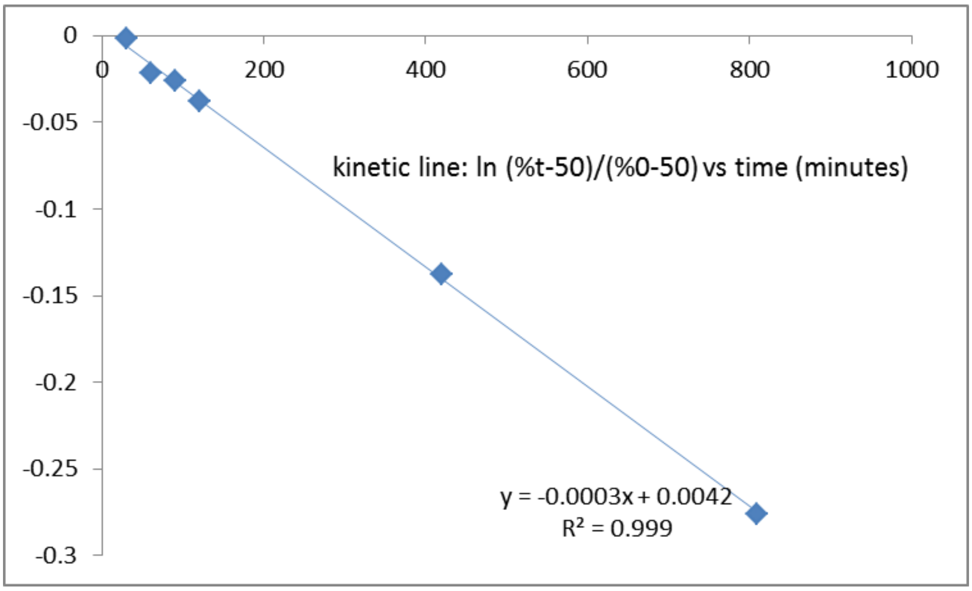

K racemization = 5.0 x 10^{-6} S^{-1}

K enantiomerization = 2.5 x 10^{-6} S^{-1}

ΔG enantiomerization = 117.9 $KJ.mol^{-1}$

Half-life time $t_{1/2}$ = 138629 seconds

2310.49 mins

38.5 hours

1.6 days

Supplementary Note 2

General Experimental Procedures

Preparation of catalyst C5

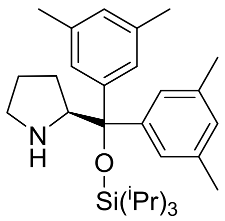

C5

(*S*)-2-(bis(3,5-dimethylphenyl)((triisopropylsilyl)oxy)methyl)pyrrolidine (**C5**)

Catalyst **C5** was prepared according to the literature[1]

To a stirred solution of (*S*)-bis(3,5-dimethylphenyl)(pyrrolidin-2-yl)methanol (500 mg, 1.6 mmol, 1.00 equiv) in dry CH_2Cl_2 (4 mL, 0.4 M) triethylamine (1.35 mL, 9.6 mmol, 6.00 equiv.) and *tert*-butyldimethylsilyltrifluoromethanesulfonate (1.1 mL, 4.8 mmol, 3.00 equiv.) were added at 0 °C. The resulting reaction mixture was then heated at 100 °C in a sealed tube for 8 h. The reaction mixture was quenched with H_2O (20 mL) and extracted with EtOAc (2 x 20 mL). The combined organic layers were washed with saturated $NaHCO_3$ (2 x 20 mL), dried over Na_2SO_4 and concentrated under reduced pressure. The residue was purified by flash column chromatography on silica gel and eluted with PE/EA (5/1) to give compound **C5** (420 mg, 56% yield) as pale solid, mp: 73.2-74.6 °C.

^{1}H NMR (500 MHz, $CDCl_3$) δ 7.09 (s, 2H), 7.03 (s, 2H), 6.88 (s, 1H), 6.87 (s, 1H), 4.09 (t, J = 7.0 Hz, 1H), 2.75 (dd, J = 16.5, 7.0 Hz, 1H), 2.48 (dd, J = 16.5, 7.0 Hz, 1H), 2.27 (s, 6H), 2.26 (s, 6H), 1.80 –1.73 (m, 2H), 1.66 – 1.60 (m, 1H), 1.54 – 1.45 (m, 1H), 1.15 – 1.02 (m, 1H), 0.94 (t, J = 7.5 Hz, 18H), 0.77 (Hept, J = 7.5 Hz, 3H).

^{13}C NMR (125 MHz, $CDCl_3$) δ = 145.6, 144.7, 136.6, 136.2, 128.7, 128.4, 127.1, 127.0, 83.6, 65.6, 47.0, 27.9, 25.2, 21.5, 21.5, 18.6, 18.5, 13.7.

HRMS (m/z) $[M]^+$ calcd for $C_{30}H_{48}NOSi$, 466.3500; found 466.3495.

1,3-Dicarbonyl Compounds **1**,[2] and phenylpropiol-aldehyde **2**[3] were prepared according to known procedures.

Preparation of 1,3-Dicarbonyl Compounds 1

1,3-Dicarbonyl Compounds **1j** and **1k** were purchased from TCI(Shanghai) Development Co., Ltd. and used without further purification. **1a-1n** were prepared similar to a literature procedure[2]: to a solution of acetylacetone (10.0 mmol, 1.0 equiv) in acetone (5 mL) at 0 ºC was added potassium carbonate (13 mmol, 1.3 equiv) in portions. The mixture was allowed to stir for 15 minutes. Then substituted benzyl bromide (13 mmol, 1.3 equiv) was added dropwise. The mixture was heated to reflux for 24 hours. The cooled mixture was filtered under reduced pressure and the filtrate was concentrated in vacuo. Flash column chromatography (silica gel, PE/EA 20/1) afforded **1** as a keto/enol

tautomer. The characterization data were consistent with those in ref. 2.

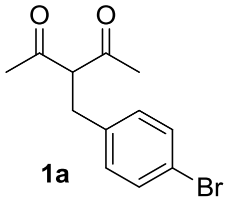

3-(4-bromobenzyl)pentane-2,4-dione

Pale yellow oil; 1.69 g, 63% yield**;**

^{1}H NMR (500 MHz, $CDCl_3$, 1:1.7 keto:enol tautomer, 1:11.4 keto:enol tautomer after recrystallization, enol tautomer annotated by an asterisk): δ 7.44 (d, J = 8.0 Hz, 2H*), 7.42 (d, J = 8.0 Hz, 2H), 7.06-7.04 (m, 2H and 2H*), 3.98 (t, J = 7.5 Hz, 1H), 3.63 (s, 2H*), 3.12 (d, J = 7.5 Hz, 2H), 2.16 (s, 6H), 2.08 (s, 6H*).

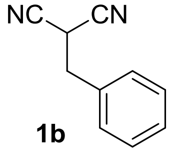

2-benzylmalononitrile

1b were prepared according to literature procedure[2]

Malononitrile (661 mg, 10 mmol) was dissolved in 95% EtOH (10 mL) and to this solution was added the appropriate benzyl aldehyde (10 mmol). The solution was stirred at r.t. until precipitation was complete or overnight. Additional EtOH (20 mL) was added and the mixture cooled to 0 °C in an ice bath. $NaBH_4$ (169 mg, 5 mmol) was introduced to the vigorously stirred mixture and the reduction was complete in about 10 min. To the reaction mixture was added H_2O (50 mL) and CH_2Cl_2 (25 mL), followed by aq 1.0 M HCl until all hydride was quenched. The layers were separated and the aqueous layer was extracted with CH_2Cl_2 (2 × 25 mL). The combined organic layers were dried (Na_2SO_4), filtered, concentrated via rotoevaporation and then under high vacuum, and Pale yellow solid was obtained via recrystallization from EA/PE, 1.48 g, 95% yield.

^{1}H NMR (400 MHz, $CDCl_3$): δ 7.51 – 7.40 (m, 3H), 7.35 (d, J = 5.8 Hz, 2H), 3.94 (t, J = 6.9 Hz, 1H), 3.30 (d, J = 6.9 Hz, 2H).

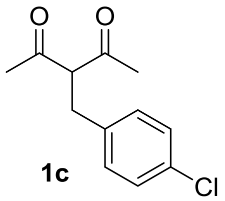

3-(4-chlorobenzyl)pentane-2,4-dione

White solid, 1.64 g, 73% yield.

^{1}H NMR (500 MHz, $CDCl_3$, 1:3.5 keto:enol tautomer, enol tautomer annotated by an asterisk): δ 7.29 (d, J = 8.5 Hz, 2H*), 7.27 (d, J = 8.5 Hz, 2H), 7.12-7.09 (m, 2H and 2H*), 4.00 (t, J = 7.5 Hz, 1H), 3.65 (s, 2H*), 3.14 (d, J = 7.5 Hz, 2H), 2.16 (s, 6H), 2.08 (s, 6H*).

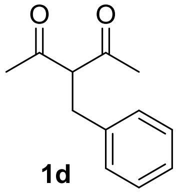

1d

3-benzylpentane-2,4-dione

Colorless oil, 1.56 g, 82% yield.

^{1}H NMR (500 MHz, $CDCl_3$, 1:2.9 keto:enol tautomer, enol tautomer annotated by an asterisk): δ 7.34-7.28 (m, 2H and 2H*), 7.25-7.22 (m, 1H and 1H*), 7.18-7.16 (m, 2H and 2H*), 4.03 (t, J = 7.5 Hz, 1H), 3.69 (s, 2H*), 3.17 (d, J = 7.5 Hz, 2H*), 2.15 (s, 6H), 2.10 (s, 6H*).

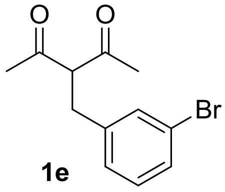

1e

3-(3-bromobenzyl)pentane-2,4-dione

White solid, 2.18 g, 85% yield.

^{1}H NMR (500 MHz, $CDCl_3$, 1:2.8 keto:enol tautomer, enol tautomer annotated by an asterisk): δ7.38-7.37 (m, 1H and 1H*), 7.35 (s, 1 H), 7.31 (s, 1 H*), 7.22-7.16 (m, 1H and 1H*), 7.12-7.09 (m, 1H and 1H*), 4.00 (t, J = 7.5 Hz, 1H), 3.67 (s, 2H*), 3.14 (d, J = 7.5 Hz, 2H), 2.17 (s, 6H), 2.09 (s, 6H*).

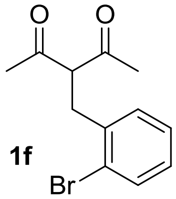

1f

3-(2-bromobenzyl)pentane-2,4-dione

White solid, 2.28 g, 89% yield.

^{1}H NMR (500 MHz, $CDCl_3$, 1:6.5 keto:enol tautomer, < 1:35 keto:enol tautomer after recrystallization, enol tautomer annotated by an asterisk): δ 7.61 (d, J = 7.9 Hz, H*), 7.29 (t, J = 8.5 Hz, H*), 7.13 (t, J = 7.6 Hz, H*), 7.07 (d, J = 7.7 Hz, H*), 3.70 (s, 2H*), 2.05 (s, 6H*).

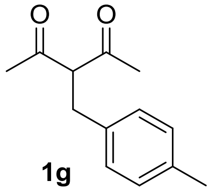

1g

3-(4-methylbenzyl)pentane-2,4-dione

White solid, 1.59 g, 78% yield.

^{1}H NMR (500 MHz, $CDCl_3$, 1:2.9 keto:enol tautomer, enol tautomer annotated by an asterisk): δ 7.14 (d, J = 8.0 Hz, 2H*), 7.11 (d, J = 8.0 Hz, 2H), 7.07-7.05 (m, 2H and 2H*), 4.01 (t, J = 7.5 Hz, 1H), 3.64

(s, 2H*), 3.14 (d, J = 7.5 Hz, 2H), 2.35 (s, 3H), 2.33 (s, 3H*), 2.15 (s, 6H), 2.10 (s, 6H*).

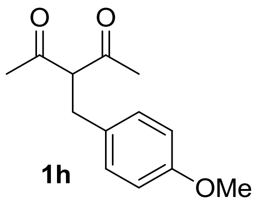

1h

3-(4-methoxybenzyl)pentane-2,4-dione

White solid; 1.52 g, 69% yield.

^{1}H NMR (400 MHz, $CDCl_3$, 1:3.2 keto:enol tautomer, enol tautomer annotated by an asterisk): δ 7.10-7.07 (m, 2H and 2H*), 6.87 (d, J = 8.8 Hz, 2H*), 6.84 (d, J = 8.8 Hz, 2H), 3.99 (t, J = 7.2 Hz, 1H), 3.81 (s, 3H*), 3.80 (s, 3H), 3.62 (s, 2H*), 3.12 (d, J = 7.6 Hz, 2H), 2.14 (s, 6H), 2.10 (s, 6H*).

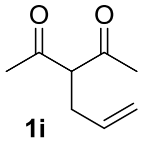

1i

3-allylpentane-2,4-dione

Pale yellow oil, 1.13 g, 82% yield.

^{1}H NMR (400 MHz, $CDCl_3$, 1:3.1 keto:enol tautomer, enol tautomer annotated by an asterisk): δ 5.86 (ddt, J = 17.2, 10.0, 5.2 Hz, 1H*), 5.72 (ddt, J = 17.2, 10.0, 6.8 Hz, 1H), 5.16-4.99 (m, 2H and 2H*), 3.74 (t, J = 7.2 Hz, 1H), 2.95 (dt, J = 7.2, 1.2 Hz, 2H*), 2.61 (tt, J = 7.2, 1.6 Hz, 2H), 2.20 (s, 6H), 2.12 (s, 6H*);

Preparation of 2a

2a were prepared according to a literature procedure[3]: to a stirred mixture of 1,2-Diiodobenzene (2.010 g, 6.1 mmol) in Et_2O (20 mL), $Pd(PPh_3)_4$ (280 mg, 0.24 mmol) and CuI (105 mg, 0.56 mmol) were added, followed by ^{n}BuNH_2 (3.0 mL, 30.4 mmol). Once a clear, homogeneous solution had been formed, prop-2-yn-1-ol (0.35 mL, 6.0 mmol) was added via syringe and stirring was continued for 6 h. One aliquot of a saturated solution of NH_4Cl was poured into the reaction flask, the phases were separated, and the aqueous layer was extracted with EtOAc (3 x 10 mL). The combined organic layers were washed with brine, dried over $MgSO_4$, and concentrated in vacuo. The crude product was purified by flash chromatography (silica gel, PE/EA 5/1) affording 3-(2-iodophenyl)prop-2-yn-1-ol as a yellowish oil. (853.4 mg, 56%). The characterization data were consistent with those in ref. 3.

To a solution of 3-(2-iodophenyl)prop-2-yn-1-ol (768.2 mg, 3.0 mmol) in CH_2Cl_2 (10 mL) was added Dess–Martin periodinane (1.53 g, 3.6 mmol) at 0 °C. Then the mixture was allowed to warm to room temperature. After stirring at this temperature for 3 h, the mixture was poured into an ice-cooled solution of saturated aqueous $NaHCO_3$ (5 mL). The mixture was extracted with EtOAc (50 mL). The combined organic layers were washed with water (2 x 5 mL) and brine (2 x 5 mL), and dried over anhydrous Na_2SO_4. Filtration and evaporation in vacuo followed by column chromatography (silica gel, PE/EA 100/1) provided **2a** (602.1 mg, 79%) as a pale yellow oil.

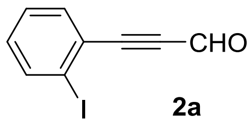

2a

3-(2-iodophenyl)propiolaldehyde

Light yellow liquid, 602.1 mg, 79% yield

^{1}H NMR (400 MHz, $CDCl_3$): δ 9.52 (s, 1H), 7.94 (d, J = 8.0 Hz, 1H), 7.62 (d, J = 7.7 Hz, 1H), 7.41 (t, J = 7.6 Hz, 1H), 7.19 (t, J = 8.5 Hz, 1H).

Preparation of 2b-2h

Bis[triphenylphosphine]palladium dichloride (1.0 mmol, 0.05 equiv) and copper iodide (1.2 mmol, 0.06 equiv) were added to a to a solution of aryl iodide (20 mmol, 1.0 equiv) in trimethylamine (50 mL) at room temperature. The reaction was allowed to stir 10 minutes, and then propargyl alcohol (22 mmol, 1.1 equiv) was added to the mixture. After reaction complete (monitored by TLC) TEA was evaporated and the residue was washed with sat NH_4Cl (50 mL), extracted with CH_2Cl_2 (2 x 150 mL) and dry over Na_2SO_4. After concentration the crude product was purified by column chromatography (silica gel, PE/EA 5/1) affording desired alcohol.

To a solution of substituted prop-2-yn-1-ol (5.0 mmol, 1.0 equiv) in CH_2Cl_2 (10 mL) was added Dess–Martin periodinane (6.0 mmol, 1.2 equiv) at 0 °C. After stirring at this temperature for 3 h, the mixture was poured into an ice-cooled solution of saturated aqueous $NaHCO_3$ (5 mL). The mixture was extracted with EtOAc (50 mL). The combined organic layers were washed with water (2 x 5 mL) and brine (2 x 5 mL), and dried over anhydrous Na_2SO_4. Filtration and evaporation in vacuo followed by column chromatography (silica gel, PE/EA 100/1-5/1) provided **2b**-**2l** desired product.

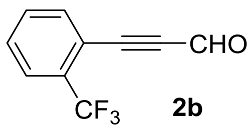

2b

3-(2-(trifluoromethyl)phenyl)propiolaldehyde

Light yellow liquid, 821.7 mg, 83 % yield.

^{1}H NMR (500 MHz, $CDCl_3$): δ 9.49 (s, 1H), 7.80 – 7.75 (m, 2H), 7.63 (d, J = 3.3 Hz, 1H), 7.62 (d, J = 3.6 Hz, 1H).

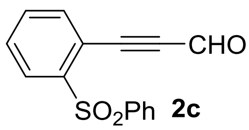

2c

3-(2-(phenylsulfonyl)phenyl)propiolaldehyde

White solid, m.p.: 110.4-111.2 °C, 1.20 g, 89 % yield.

^{1}H NMR (400 MHz, $CDCl_3$): δ 9.45 (s, 1H), 8.38 (d, J = 7.6 Hz, 1H), 8.03 – 8.01 (m, 2H), 7.74 – 7.70 (m, 2H), 7.67 – 7.61 (m, 2H), 7.57– 7.53 (m, 2H).

^{13}C NMR (100 MHz, $CDCl_3$): δ = 176.1, 143.4, 139.9, 136.2, 133.7, 133.2, 131.4, 129.6, 129.0, 128.6, 118.6, 94.1, 89.5.

HRMS (m/z): $[M]^+$ calcd for $C_{15}H_{11}O_3S$, 271.0423; found 271.0419.

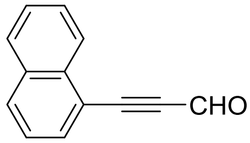

2d

3-(naphthalen-1-yl)propiolaldehyde

^{1}H NMR (500 MHz, $CDCl_3$): δ 9.59 (s, 1H), 8.35 (d, J = 8.0 Hz, 1H), 8.02 (d, J = 8.0 Hz, 1H), 7.93 – 7.90 (m, 2H), 7.67 (t, J = 7.5 Hz, 1H), 7.60 (t, J = 7.5 Hz, 1H), 7.52 (t, J = 7.5 Hz, 1H).

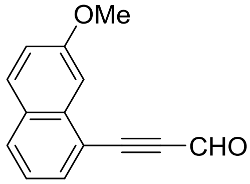

2e

3-(7-methoxynaphthalen-1-yl)propiolaldehyde

Pale yellow solid, m.p.: 80.2-81.1 °C, 0.96 g, 91 % yield.

^{1}H NMR (500 MHz, $CDCl_3$): δ 9.60 (s, 1H), 7.94 (d, J = 8.0 Hz, 1H), 7.88 (d, J = 8.0 Hz, 1H), 7.82 (d, J = 9.0 Hz, 1H), 7.61 (s, 1H), 7.41 – 7.34 (m, 1H), 7.25 (dd, J = 9.0, 2.5 Hz, 1H), 4.02 (s, 3H).

^{13}C NMR (125 MHz, $CDCl_3$): δ = 176.6, 159.4, 135.4, 134.4, 132.0, 130.2, 128.6, 122.9, 119.9, 115.4, 103.8, 94.1, 93.5, 55.5.

HRMS (m/z): $[M]^+$ calcd for $C_{14}H_{11}O_2$, 211.0754; found 211.0751.

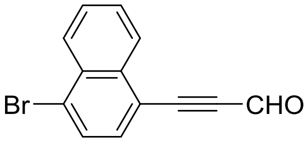

2f

3-(4-bromonaphthalen-1-yl)propiolaldehyde

Pale yellow solid, m.p.: 107.6-108.2 °C, 1.12 g, 87 % yield

^{1}H NMR (500 MHz, $CDCl_3$): δ 9.58 (s, 1H), 8.3 – 8.32 (m, 2H), 7.84 (d, J = 7.5 Hz, 1H), 7.74 – 7.71 (m, 3H).

^{13}C NMR (125 MHz, $CDCl_3$): δ = 176.5, 134.4, 133.4, 131.9, 129.5, 128.7, 128.5, 127.9, 127.8, 126.2, 117.0, 93.6, 92.4.

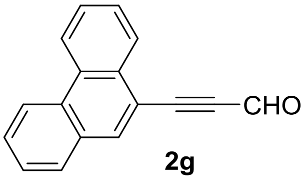

2g

3-(phenanthren-9-yl)propiolaldehyde

Pale yellow solid, m.p.: 98.4-99.0 °C, 0.98 g, 85 % yield.

^{1}H NMR (500 MHz, $CDCl_3$): δ 9.62 (s, 1H), 8.73 – 8.68 (m, 2H), 8.43 (d, J = 7.0 Hz, 1H), 8.25 (d, J = 3.0 Hz, 1H), 7.92 (d, J = 8.0 Hz, 1H), 7.82 – 7.71 (m, 3H), 7.67 (t, J = 7.0 Hz, 1H).

^{13}C NMR (125 MHz, $CDCl_3$): δ = 176.7, 136.4, 131.5, 130.5, 130.5, 130.0, 129.3, 129.2, 127.7, 127.7, 127.4, 126.5, 123.0, 122.8, 115.9, 93.7, 92.6.

HRMS (m/z): $[M]^+$ calcd for $C_{17}H_{11}O$, 231.0804; found 231.0799.

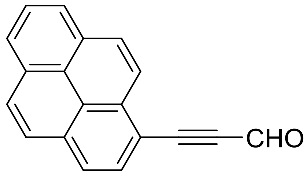

2h

3-(pyren-1-yl)propiolaldehyde

Yellow solid, m.p.: 121.3-124.3 ˚C, 1.06 g, 85 % yield

^{1}H NMR (500 MHz, $CDCl_3$): δ 9.67 (s, 1H), 8.61 (d, J = 9.0 Hz, 1H), 8.33 (d, J = 8.0 Hz, 1H), 8.31 – 8.28 (m, 3H), 8.23 (d, J = 9.0 Hz, 1H), 8.18 (d, J = 8.0 Hz, 1H), 8.13 – 8.10 (m, 2H).

^{13}C NMR (125 MHz, $CDCl_3$) δ = 176.7, 133.9, 133.6, 131.4, 131.0, 130.8, 123.0, 129.7, 127.1, 126.7, 126.7, 126.6, 124.8, 124.6, 124.2, 123.9, 113.0, 95.0, 94.1.

HRMS (ESI) calcd for $C_{19}H_{11}O$ (M + H^+) 255.0804, Found 255.0800.

Supplementary Note 3

General procedure for preparation of racemic compound 3

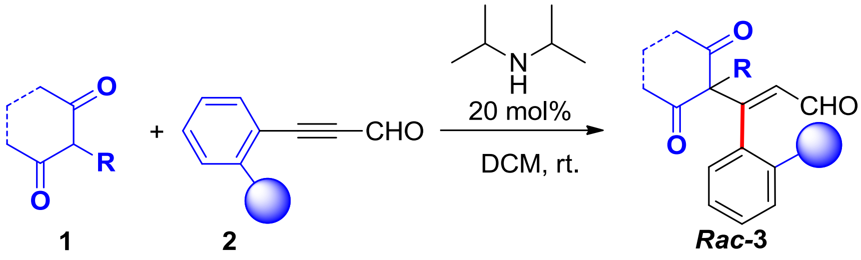

Diisopropylamine (20 mol%) was added to a solution of **1**(0.05 mmol) and **2** (0.06 mmol) in methylene chloride (0.5 mL). And the mixture was stirred till **1** was completely consumed (monitored by TLC). The mixture was concentrated under reduced pressure and purified by flash chromatography on silica gel and eluted with PE/EA (8/1 to 3/1) to afford the corresponding desired racemic product **3.**

General procedure for the asymmetric synthesis of axially chiral styrenes 3

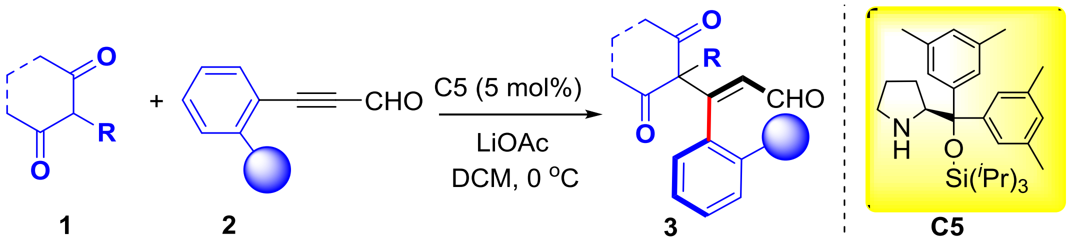

(*S*)-2-bis(3,5-dimethylphenyl)((triisopropylsilyloxy) methyl)pyrrolidine **C5** (5 mol%) and LiOAc were added to a solution of **2** (0.11 mmol, 1.1 equiv) in methylene chloride (1 mL). After the mixture was cooled to 0 °C, **1** (0.10 mmol, 1.0 equiv) was added and the system was maintained at 0 °C for 24 h till **1** was completely consumed (monitored by TLC). The mixture was concentrated under reduced pressure and purified by flash chromatography on silica gel and eluted with PE/EA (8/1 to 3/1) to afford the corresponding desired axially chiral styrenes product **3.**

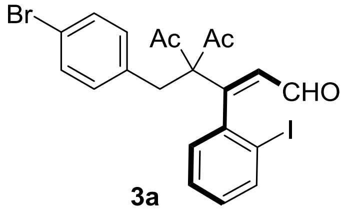

3a

(*S*$_a$)-(*Z*)-4-acetyl-4-(4-bromobenzyl)-3-(2-iodophenyl)-5-oxohex-2-enal

Pale yellow solid, m.p.: 102.7-103.6 °C, 50.4 mg, 96% yield, 94% ee.

^{1}H NMR (400 MHz, $CDCl_3$): δ 9.31 (d, J = 7.6 Hz, 1H), 8.03 (d, J = 8.0 Hz, 1H), 7.44 (t, J = 7.6 Hz, 1H), 7.32 (d, J = 8.4 Hz, 2H), 7.17 (t, J = 7.6 Hz, 1H), 6.95 (d, J = 8.4 Hz, 2H), 6.86 (d, J = 8.0 Hz, 1H), 6.24 (d, J = 7.6 Hz, 1H), 3.36 (d, J = 14.6 Hz, 1H), 2.90 (d, J = 14.6 Hz, 1H), 2.44 (s, 3H), 2.38 (s, 3H).

^{13}C NMR (125 MHz, $CDCl_3$): δ 204.8, 203.0, 191.3, 157.9, 140.1, 138.2, 134.5, 134.0, 131.8, 131.4, 130.9, 128.9, 128.7, 121.2, 100.6, 78.6, 35.6, 29.1, 27.7.

HRMS (m/z): $[M]^+$ calcd for $C_{21}H_{19}O_3BrI$, 524.9562; found 524.9566.

HPLC separation conditions: CHIRALPAK IA column (250 mm × 4.6 mm), hexane:*i*-PrOH = 80:20, 1.0 mL/min, T = 25 °C, λ = 254 nm, t_R (major) = 7.7 min, t_R (minor) = 9.8 min.

*Chiral HPLC spectrum of racemic **3a***

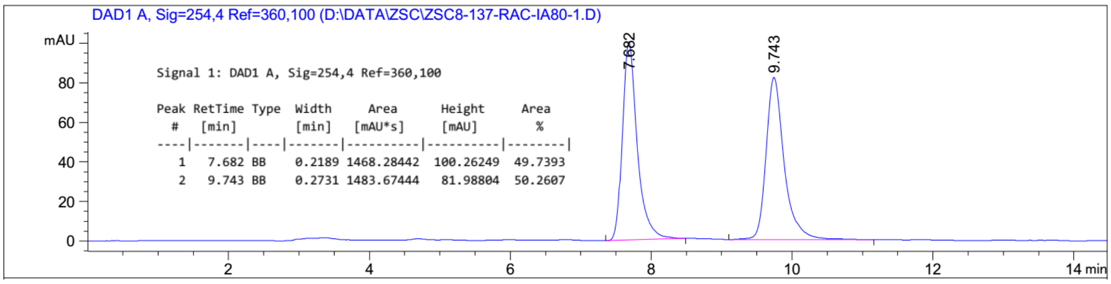

*Chiral HPLC spectrum of **3a***

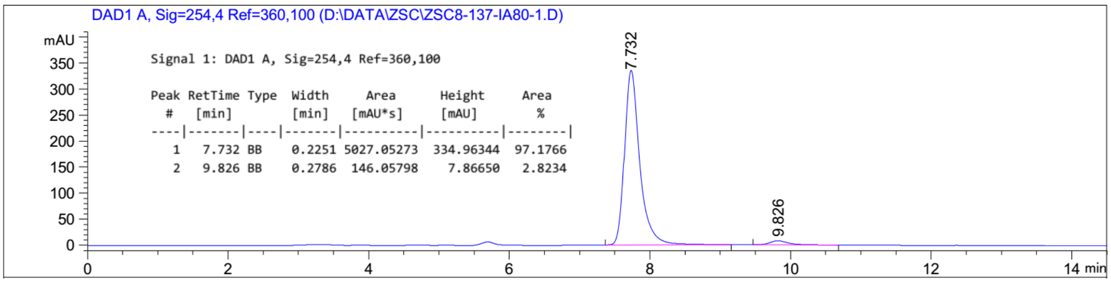

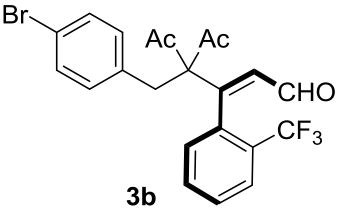

3b

(S_a)-(*E*)-4-acetyl-4-(4-bromobenzyl)-5-oxo-3-(2-(trifluoromethyl)phenyl)hex-2-enal

White solid, m.p.: 101.7-103.5 °C, 44.3 mg, 95% yield, 90% ee.

^{1}H NMR (500 MHz, $CDCl_3$): δ 9.16 (d, J = 7.5 Hz, 1H), 7.94 – 7.82 (m, 1H), 7.69 – 7.54 (m, 2H), 7.28 (d, J = 8.0 Hz, 2H), 7.02 – 6.94 (m, 1H), 6.90 (d, J = 8.0 Hz, 2H), 6.27 (d, J = 7.5 Hz, 1H), 3.28 (d, J = 14.5 Hz, 1H), 2.80 (d, J = 14.5 Hz, 1H), 2.41 (s, 3H), 2.21 (s, 3H).

^{13}C NMR (125 MHz, $CDCl_3$): δ 204.6, 203.1, 190.5, 153.9, 134.7, 134.4, 132.3, 131.9, 131.7 (q, J = 2.3 Hz), 131.4, 129.9, 129.8, 129.2 (q, J = 29.6 Hz), 127.4 (q, J = 5.2 Hz), 123.8 (q, J = 273.9 Hz), 121.2, 78.5, 35.8, 28.1, (q, J = 8.0 Hz), 27.5.

^{19}F NMR (376 MHz, $CDCl_3$): δ -56.0.

HRMS (m/z): $[M]^+$ calcd for $C_{22}H_{19}O_3BrF_3$, 467.0464; found 467.0468.

HPLC separation conditions: CHIRALPAK IA column (250 mm × 4.6 mm), hexane:*i*-PrOH = 80:20, 1.0 mL/min, T = 25 °C, λ = 254 nm, t_R (major) = 5.9 min, t_R (minor) = 6.8 min.

*Chiral HPLC spectrum of racemic **3b***

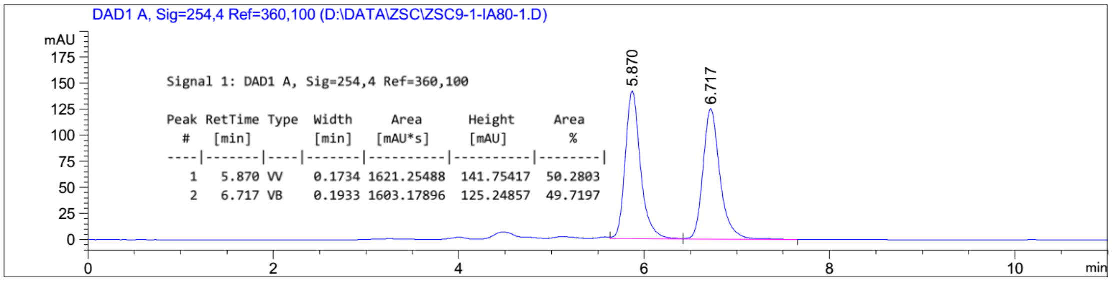

Chiral HPLC spectrum of ***3b***

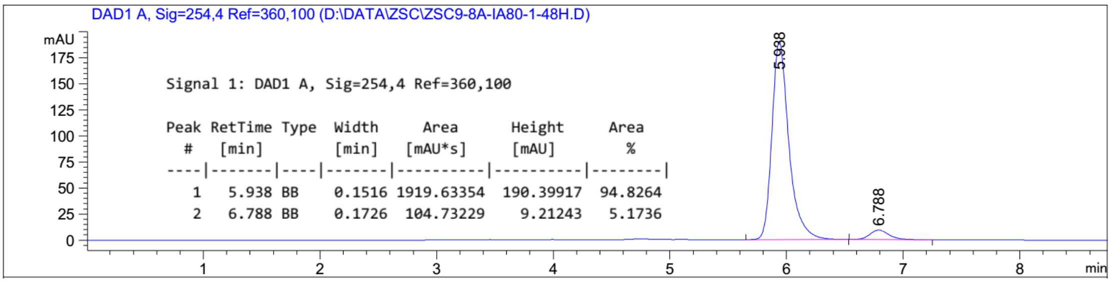

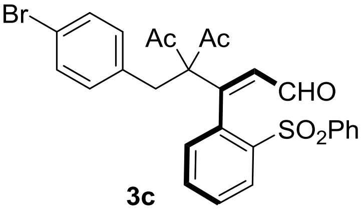

3c

(S_a)-(Z)-4-acetyl-4-(4-bromobenzyl)-5-oxo-3-(2-(phenylsulfonyl)phenyl)hex-2-enal

White solid, m.p.: 167.4-168.6 °C, 21.5 mg, 40% yield, 82% ee.

^{1}H NMR (400 MHz, $CDCl_3$): δ 8.48 (d, J = 7.6 Hz, 1H), 8.13 – 8.03 (m, 1H), 7.86 (dt, J = 7.2, 1.2 Hz, 2H), 7.69 (tt, J = 6.8, 1.2 Hz, 1H), 7.66 – 7.61 (m, 2H), 7.58 (t, J = 7.6 Hz, 2H), 7.31 (d, J = 8.4 Hz, 2H), 7.01 (d, J = 8.4 Hz, 2H), 6.95 – 6.87 (m, 1H), 6.16 (d, J = 7.6 Hz, 1H), 3.58 (d, J = 14.2 Hz, 1H), 3.28 (d, J = 14.1 Hz, 1H), 2.50 (s, 3H), 2.42 (s, 3H).

^{13}C NMR (100 MHz, $CDCl_3$): δ 205.8, 203.3, 188.9, 154.7, 140.1, 140.0, 135.0, 134.4, 133.9, 133.3, 132.9, 132.2, 131.3, 130.9, 130.4, 130.0, 129.7, 128.0, 121.0, 79.1, 35.3, 28.7, 27.5.

HRMS (m/z): $[M]^+$ calcd for $C_{27}H_{23}O_5{}^{81}BrNaS$, 563.0321; found 563.0311.

HPLC separation conditions: CHIRALPAK IA column (250 mm × 4.6 mm), hexane:*i*-PrOH = 80:20, 1.0 mL/min, T = 25 °C, λ = 254 nm, t_R (major) = 14.6 min, t_R (minor) =19.7 min.

Chiral HPLC spectrum of racemic ***3c***

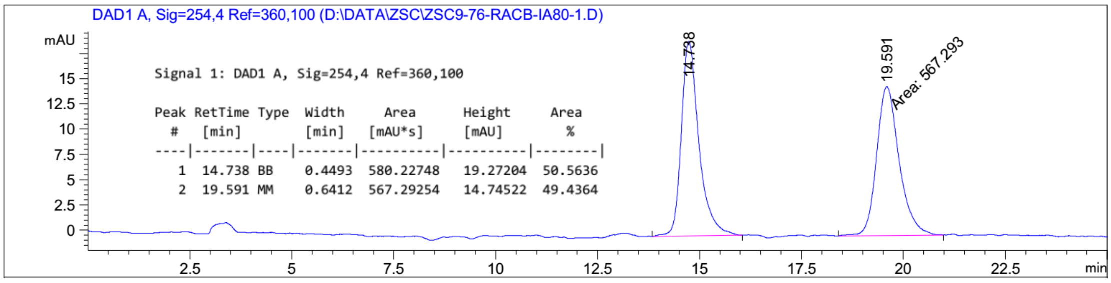

Chiral HPLC spectrum of ***3c***

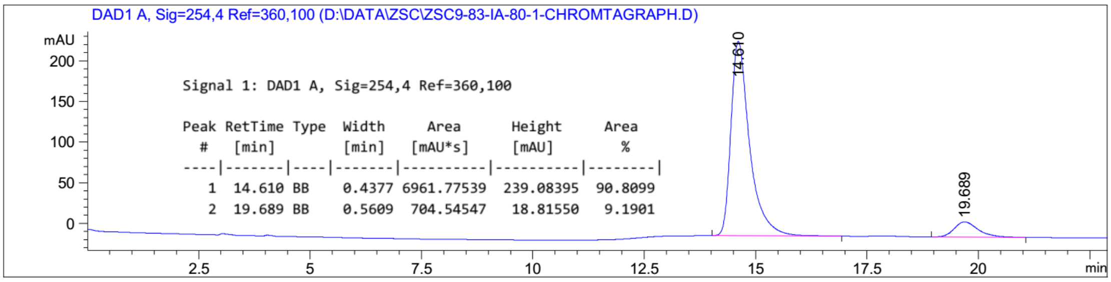

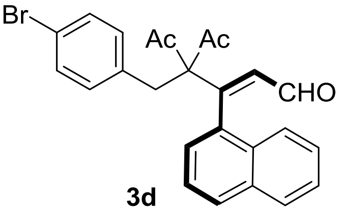

3d

(S_a)-(E)-4-acetyl-4-(4-bromobenzyl)-3-(naphthalen-1-yl)-5-oxohex-2-enal

White solid, m.p.: 131.8-132.7 °C, 44.5 mg, 99% yield, 92% ee.

^{1}H NMR (500 MHz, $CDCl_3$): δ 9.03 (d, J = 7.5 Hz, 1H), 7.94 (d, J = 8.5 Hz, 2H), 7.91 (d, J = 8.0 Hz, 1H), 7.62 (dd, J = 7.0, 1.0 Hz, 1H), 7.59 (dd, J = 7.0, 1.0 Hz, 1H), 7.50 (d, J = 8.0 Hz, 1H), 7.24 (d, J = 8.5 Hz, 2H), 7.05 (d, J = 7.0 Hz, 1H), 6.83 (d, J = 8.5 Hz, 2H), 6.43 (d, J = 7.5 Hz, 1H), 3.34 (d, J = 14.5 Hz, 1H), 2.76 (d, J = 14.5 Hz, 1H), 2.41 (s, 3H), 2.26 (s, 3H).

^{13}C NMR (125 MHz, $CDCl_3$): δ 204.4, 203.0, 192.0, 156.4, 135.0, 134.4, 133.4, 132.6, 131.8, 131.3, 131.0, 129.9, 129.1, 128.0, 126.7, 126.0, 124.9, 124.4, 121.1, 79.2, 36.6, 29.2, 28.2.

^{13}C NMR- DEPT 135 (125 MHz, $CDCl_3$) δ 192.0, 135.0, 131.9, 131.4, 129.9, 129.1, 128.0, 126.8, 126.0, 125.0, 124.4, 36.0, 29.3, 28.2.

HRMS (m/z): $[M]^+$ calcd for $C_{25}H_{22}O_3Br$, 449.0752; found 449.0752.

HPLC separation conditions: CHIRALPAK IC column (250 mm × 4.6 mm), hexane:*i*-PrOH = 70:30, 1.0 mL/min, T = 25 °C, λ = 254 nm, t_R (minor) = 9.9 min, t_R (major) = 23.0 min.

*Chiral HPLC spectrum of racemic **3d***

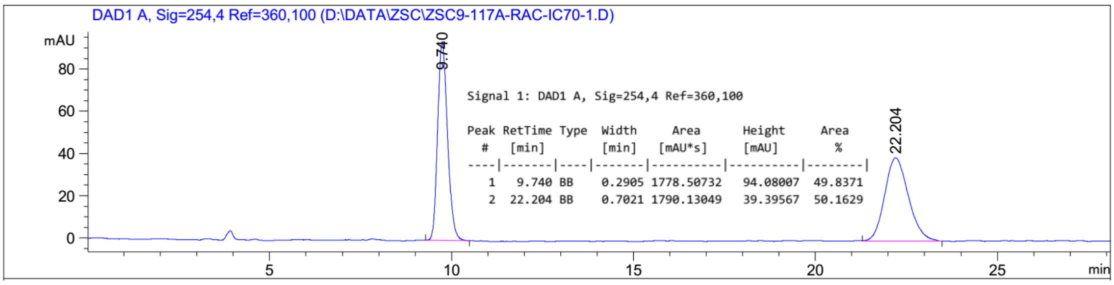

*Chiral HPLC spectrum of **3d***

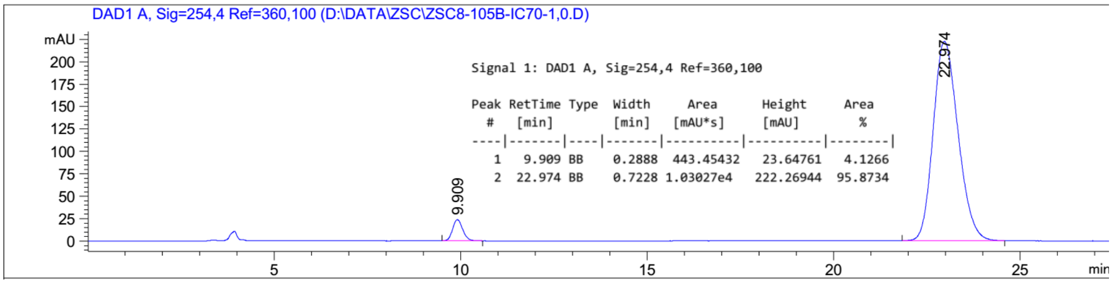

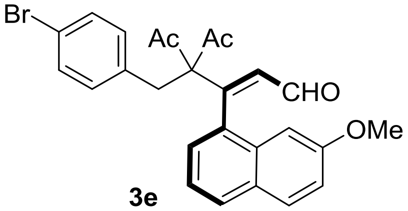

3e

(S_a)-(E)-4-acetyl-4-(4-bromobenzyl)-3-(7-methoxynaphthalen-1-yl)-5-oxohex-2-enal

White solid, m.p.: 141.6-142.7 °C, 35.9 mg, 75% yield, 87% ee.

^{1}H NMR (500 MHz, $CDCl_3$): δ 9.06 (d, J = 7.5 Hz, 1H), 7.85 (d, J = 8.5 Hz, 1H), 7.82 (d, J = 9.0 Hz, 1H), 7.35 (t, J = 7.5 Hz, 1H), 7.28 – 7.21 (m, 3H), 7.17 (d, J = 2.5 Hz, 1H), 7.01 (d, J = 7.0 Hz, 1H), 6.83 (d, J = 8.5 Hz, 2H), 6.41 (d, J = 7.5 Hz, 1H), 3.90 (s, 3H), 3.37 (d, J = 14.5 Hz, 1H), 2.85 (d, J = 14.5 Hz, 1H), 2.40 (s, 3H), 2.21 (s, 3H).

^{13}C NMR (125 MHz, $CDCl_3$): δ 204.3, 203.0, 192.3, 159.0, 157.0, 135.1, 134.4, 133.8, 131.9, 131.3, 130.5, 129.9, 129.6, 128.9, 126.5, 122.6, 121.1, 119.3, 103.2, 79.1, 55.4, 36.7, 29.2, 28.2.

^{13}C NMR- DEPT 135 (125 MHz, $CDCl_3$) δ 192.3, 135.2, 131.9, 131.4, 130.5, 129.6, 126.5, 122.7, 119.3, 103.3, 55.4, 36.8, 29.3, 28.3.

HRMS (m/z): $[M]^+$ calcd for $C_{26}H_{24}O_4Br$, 479.0852; found 479.0842

HPLC separation conditions: CHIRALPAK IA column (250 mm × 4.6 mm), hexane:*i*-PrOH = 80:20, 1.0 mL/min, T = 25 °C, λ = 254 nm, t_R (major) = 7.5 min, t_R (minor) = 9.3 min.

*Chiral HPLC spectrum of racemic **3e***

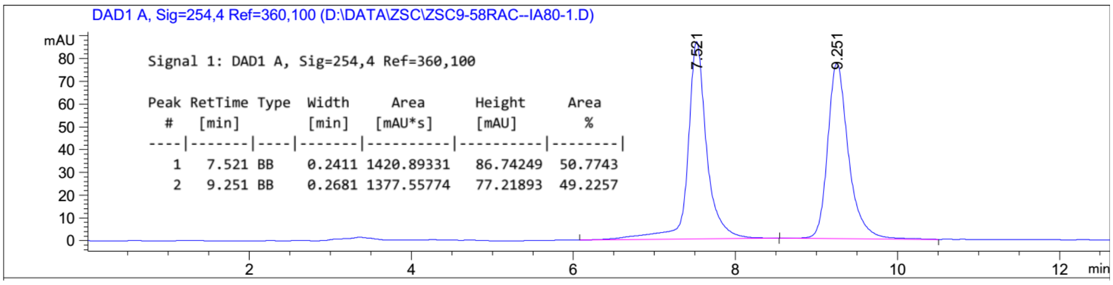

*Chiral HPLC spectrum of **3e***

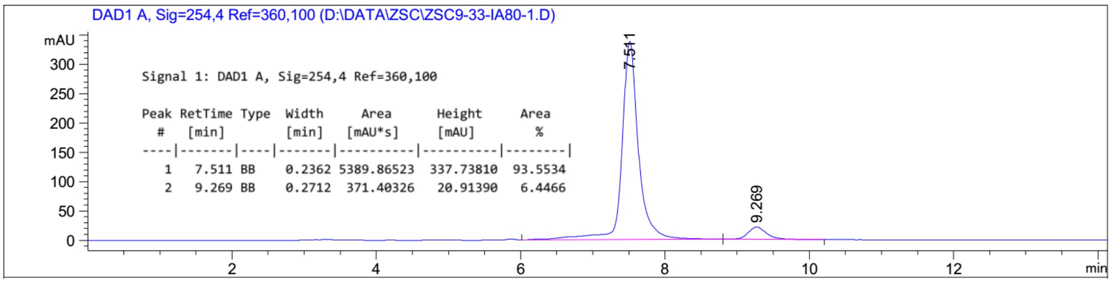

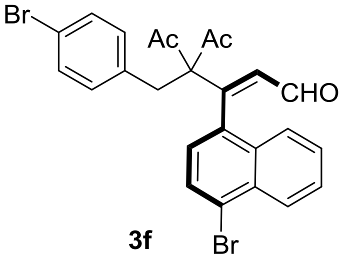

3f

(S_a)-(E)-4-acetyl-4-(4-bromobenzyl)-3-(4-bromonaphthalen-1-yl)-5-oxohex-2-enal

White solid, m.p.:156.4-158.3 °C, 52.4 mg, 99% yield, 88% ee

^{1}H NMR (500 MHz, $CDCl_3$): δ 9.05 (d, J = 7.5 Hz, 1H), 8.39 (d, J = 8.5 Hz, 1H), 7.95 (d, J = 8.0 Hz, 1H), 7.83 (d, J = 7.5 Hz, 1H), 7.73 (t, J = 7.0 Hz, 1H), 7.69 (t, J = 7.0 Hz, 1H), 7.26 (d, J = 8.5 Hz, 2H), 6.90 (d, J = 7.5 Hz, 1H), 6.83 (d, J = 8.5 Hz, 2H), 6.45 (d, J = 7.5 Hz, 1H), 3.36 (d, J = 14.5 Hz, 1H), 2.76 (d, J = 14.5 Hz, 1H), 2.43 (s, 3H), 2.25 (s, 3H).

^{13}C NMR (125 MHz, $CDCl_3$): δ 204.1, 202.7, 191.5, 155.2, 135.4, 134.1, 133.7, 132.0, 131.8, 131.4, 131.0, 129.1, 128.7, 128.4, 128.2, 126.1, 125.1, 124.9, 121.2, 79.0, 36.7, 29.3, 28.2.

HRMS (m/z): $[M]^+$ calcd for $C_{25}H_{21}O_3Br^{81}Br$, 528.9832; found 528.9836.

HPLC separation conditions: CHIRALPAK IC column (250 mm × 4.6 mm), hexane:*i*-PrOH = 70:30, 1.0 mL/min, T = 25 °C, λ = 254 nm, t_R (minor) = 11.0 min, t_R (major) = 27.9 min.

*Chiral HPLC spectrum of racemic **3f***

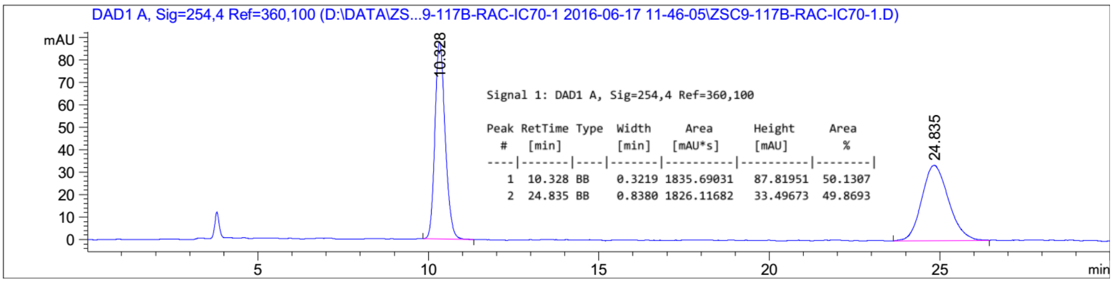

*Chiral HPLC spectrum of **3f***

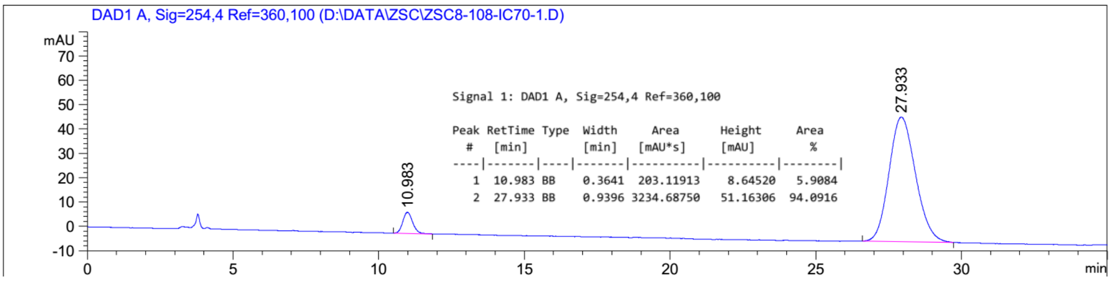

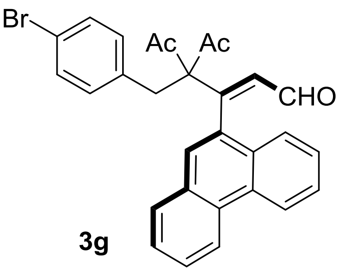

3g

(S_a)-(E)-4-acetyl-4-(4-bromobenzyl)-5-oxo-3-(phenanthren-9-yl)hex-2-enal

White solid, m.p.: 174.5-176.7 °C, 47.8 mg, 96% yield, 84% ee

^{1}H NMR (400 MHz, $CDCl_3$): δ 9.16 (d, J = 7.6 Hz, 1H), 8.79 (d, J = 8.0 Hz, 1H), 8.71 (d, J = 8.0 Hz, 1H), 7.99 (dd, J = 8.0, 1.6 Hz, 1H), 7.85 (d, J = 7.6 Hz, 1H), 7.81 – 7.61 (m, 4H), 7.23 (d, J = 8.4 Hz, 2H), 6.84 (d, J = 8.4 Hz, 2H), 6.48 (d, J = 7.6 Hz, 1H), 3.45 (d, J = 14.4 Hz, 1H), 2.85 (d, J = 14.4 Hz, 1H), 2.46 (s, 3H), 2.27 (s, 3H).

^{13}C NMR (100 MHz, $CDCl_3$): δ 204.3, 202.8, 191.9, 156.3, 135.5, 134.4, 131.9, 131.3, 131.3, 130.4, 130.3, 130.0, 129.8, 129.2, 128.1, 127.9, 127.5, 127.5, 127.4, 125.6, 123.6, 122.6, 121.1, 79.3, 36.6,

29.2, 28.2.

^{13}C NMR- DEPT 135 (100 MHz, $CDCl_3$) δ 192.0, 135.5, 132.0, 131.4, 129.2, 128.2, 127.9, 127.6, 127.6, 127.5, 125.6, 123.7, 122.6, 36.7, 29.3, 28.3.

HRMS (m/z): $[M]^+$ calcd for $C_{29}H_{24}O_3Br$, 499.0903, found 499.0906.

HPLC separation conditions: CHIRALPAK IC column (250 mm × 4.6 mm), hexane:*i*-PrOH = 70:30, 1.0 mL/min, T = 25 °C, λ = 254 nm, t_R (minor) = 11.2 min, t_R (major) = 34.4 min.

*Chiral HPLC spectrum of racemic **3g***

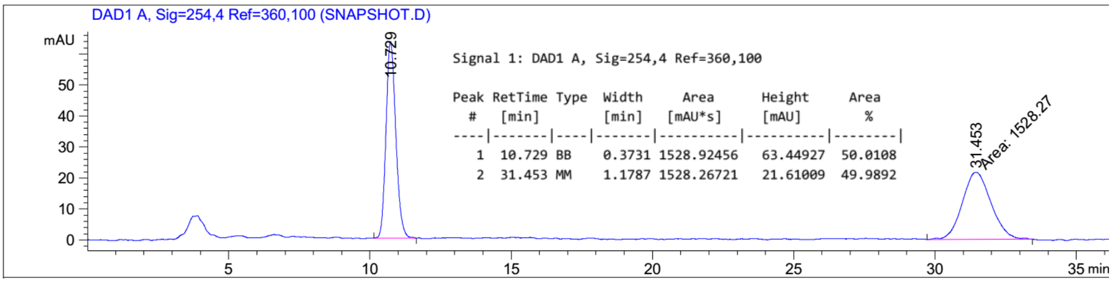

*Chiral HPLC spectrum of **3g***

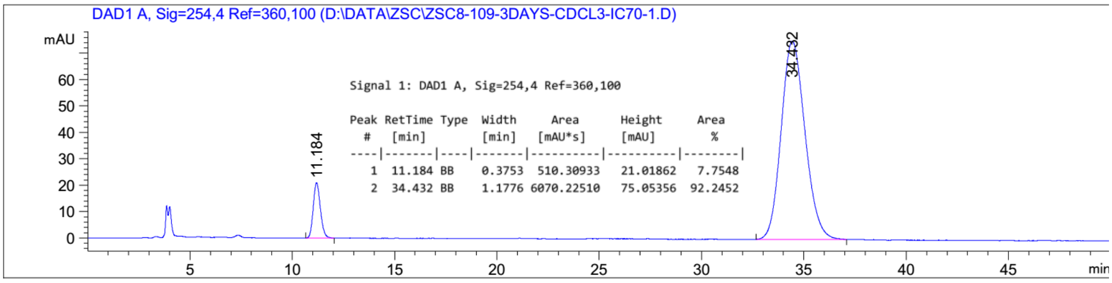

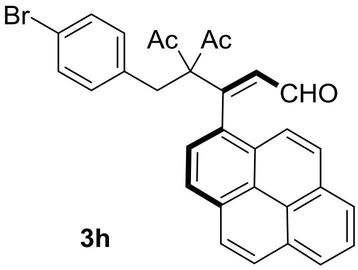

3h

(S_a)-(*E*)-4-acetyl-4-(4-bromobenzyl)-5-oxo-3-(pyren-1-yl)hex-2-enal

Yellow solid, m.p.: 206.7-207.7 °C, 51.8 mg, 99% yield, 87% ee

^{1}H NMR (400 MHz, $CDCl_3$): δ 9.03 (d, J = 7.6 Hz, 1H), 8.36 – 8.02 (m, 8H), 7.53 (d, J = 7.6 Hz, 1H), 7.21 (d, J = 8.0 Hz, 2H), 6.81 (d, *J* = 8.0 Hz, 2H), 6.56 (d, J = 7.6 Hz, 1H), 3.34 (d, J = 14.4 Hz, 1H), 2.80 (d, J = 14.4 Hz, 1H), 2.48 (s, 3H), 2.30 (s, 3H).

^{13}C NMR (100 MHz, $CDCl_3$): δ 204.3, 203.2, 192.0, 156.8, 135.3, 134.4, 131.9, 131.8, 131.3, 130.5, 130.4, 129.9, 128.8, 127.9, 127.1, 126.8, 126.4, 126.0, 125.1, 124.6, 124.5, 124.4, 123.1, 121.1, 79.4, 37.1, 29.3, 28.3.

HRMS (m/z): $[M]^+$ calcd for $C_{31}H_{24}O_3Br$, 523.0903; found 523.0911.

HPLC separation conditions: CHIRALPAK IC column (250 mm × 4.6 mm), hexane:*i*-PrOH = 70:30, 1.0 mL/min, T = 25 °C, λ = 254 nm, t_R (minor) = 14.9 min, t_R (major) = 29.6 min.

Chiral HPLC spectrum of racemic ***3h***

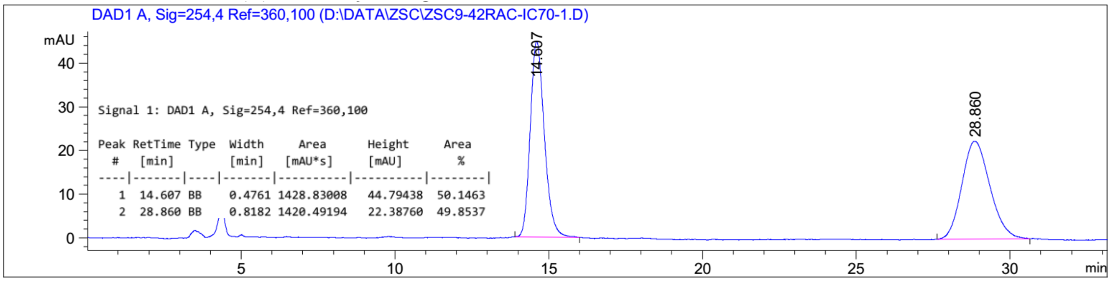

Chiral HPLC spectrum of ***3h***

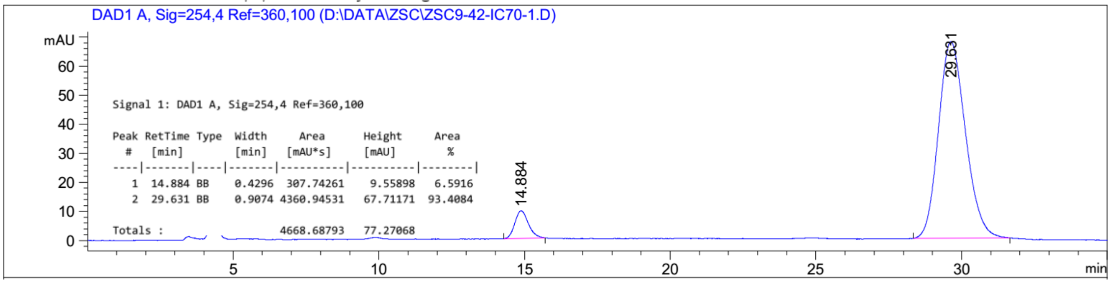

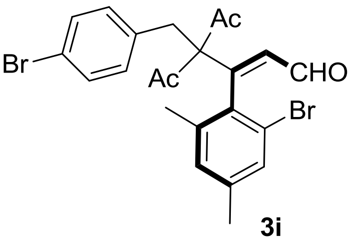

3i

(S_a) (Z)-4-acetyl-3-(2-bromo-4,6-dimethylphenyl)-4-(4-bromobenzyl)-5-oxohex-2-enal

Pale yellow solid, m.p.: 128.6-130.8 °C, 44.9 mg, 89% yield, 54% ee

^{1}H NMR (500 MHz, $CDCl_3$): δ 9.22 (d, J = 7.5 Hz, 1H), 7.39 (s, 1H), 7.30 (d, J = 8.5 Hz, 2H), 7.06 (s, 1H), 6.96 (d, J = 8.5 Hz, 2H), 6.36 (d, J = 7.5 Hz, 1H), 3.27 (d, J = 14.5 Hz, 1H), 3.21 (d, J = 14.5 Hz, 1H), 2.37(s, 3H), 2.34(s, 3H), 2.32(s, 3H), 2.15(s, 3H).

^{13}C NMR (125 MHz, $CDCl_3$): δ 204.4, 203.7, 191.5, 157.5, 140.9, 138.7, 135.6, 134.6, 132.2, 131.5, 131.3, 131.2, 131.2, 123.8, 121.2, 78.3, 36.6, 29.2, 28.4, 21.5, 20.7.

HRMS (m/z): $[M]^+$ calcd for $C_{19}H_{14}ON_2I$, 505.0008; found 505.0010.

HPLC separation conditions: CHIRALPAK IA column (250 mm × 4.6 mm), hexane:*i*-PrOH = 80:20, 1.0 mL/min, T = 25 °C, λ = 254 nm, t_R (minor) = 7.0 min, t_R (major) = 7.8 min.

Chiral HPLC spectrum of racemic ***3i***

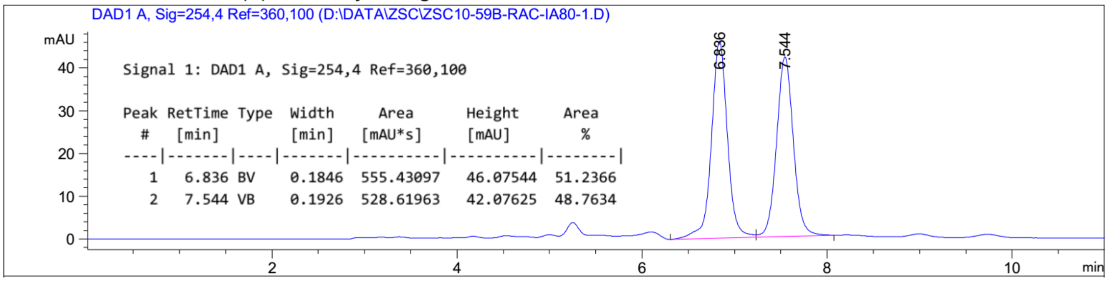

Chiral HPLC spectrum of ***3i***

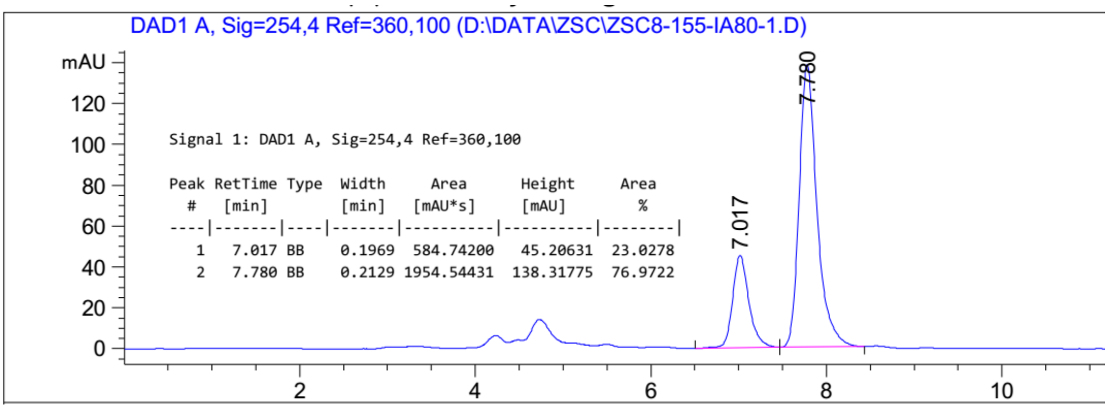

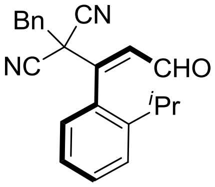

3j

(S_a) (E)-2-benzyl-2-(1-(2-isopropylphenyl)-3-oxoprop-1-en-1-yl)malononitrile

Yellow oil, 64.9 mg (0.2 mmol), 99% yield, 95% ee

^{1}H NMR (500 MHz, $CDCl_3$): δ 9.31 (d, J = 7.0 Hz, 1H), 7.59 – 7.54 (m, 2H), 7.45 – 7.42 (m, 5H), 7.35 (td, J = 7.0, 2.0 Hz, 1H), 7.15 (d, J = 7.5 Hz, 1H), 6.73 (d, J = 7.5 Hz, 1H), 3.51 (d, J = 13.5 Hz, 1H), 3.34 (d, J = 13.5 Hz, 1H), 2.90 (hept, J = 6.0 Hz, 1H), 1.43 (d, J = 6.0 Hz, 3H), 1.15 (d, J = 6.0 Hz, 3H).

^{13}C NMR (125 MHz, $CDCl_3$): δ 191.0, 153.2, 148.2, 133.0, 131.2, 130.9, 130.6, 129.4, 129.2, 129.1, 129.0, 127.0, 126.3, 113.2, 112.8, 46.7, 44.7, 31.9, 25.2, 23.0.

^{13}C NMR (126 MHz, CDCl3): δ 191.1, 133.0, 131.2, 130.6, 129.4, 129.2, 129.1, 127.0, 126.3, 44.7, 25.2, 23.0.

HRMS (m/z): $[M]^+$ calcd for $C_{22}H_{21}ON_2$, 329.1648; found 329.1646.

HPLC separation conditions: CHIRALPAK IB column (250 mm × 4.6 mm), hexane:THF = 90:10, 1.0 mL/min, T = 25 °C, λ = 254 nm, t_R (major) = 15.8 min, t_R (minor) =21.8 min.

*Chiral HPLC spectrum of racemic **3j***

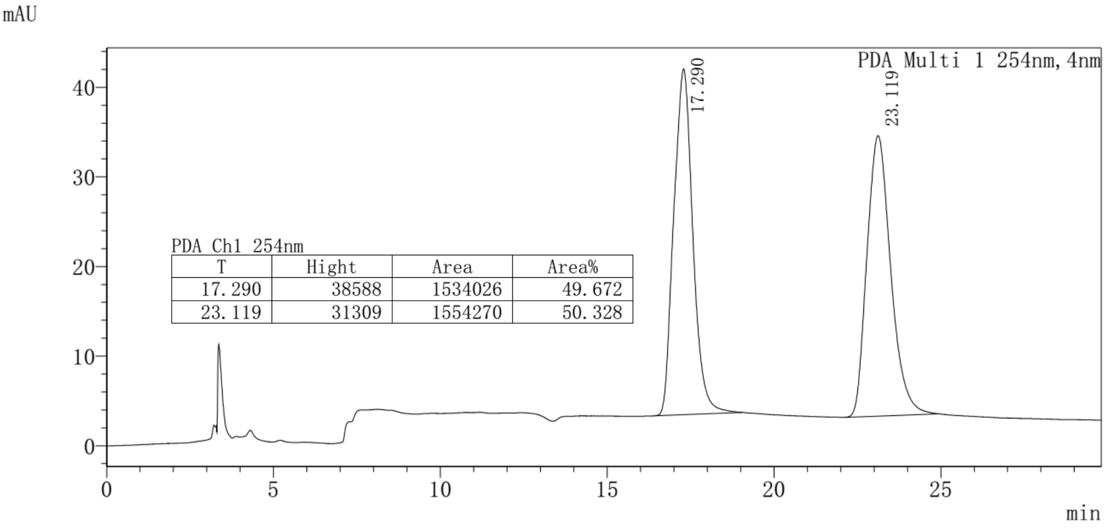

*Chiral HPLC spectrum of **3j***

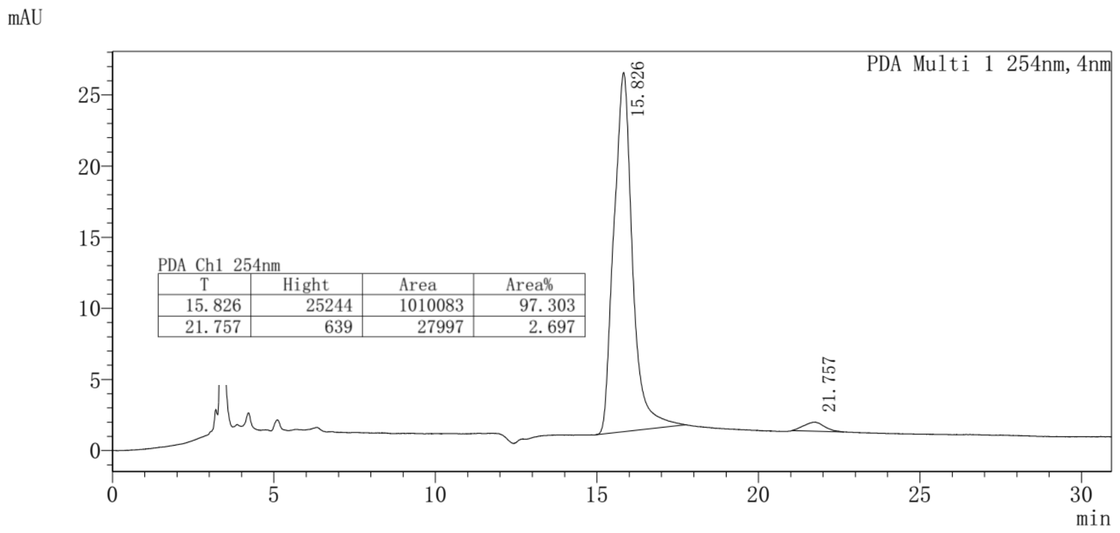

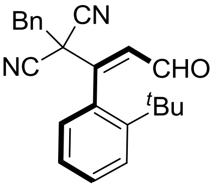

3k

(S_a) *(E)*-2-benzyl-2-(1-(2-(tert-butyl)phenyl)-3-oxoprop-1-en-1-yl)malononitrile

Yellow oil, 63.6 mg (0.2 mmol), 93% yield, 94% ee

^{1}H NMR (500 MHz, $CDCl_3$): δ 9.41 (d, J = 7.5 Hz, 1H), 7.75 (dd, J = 8.0, 1.0 Hz, 1H), 7.54 (td, J = 8.0, 1.5 Hz, 1H), 7.46 (s, 5H), 7.24 (td, J = 7.5, 1.5 Hz, 1H), 7.05 (dd, J = 7.5, 1.0 Hz, 1H), 6.52 (d, J = 7.0 Hz, 1H), 3.71 (d, J = 13.5 Hz, 1H), 3.40 (d, J = 13.5 Hz, 1H), 1.49 (s, 9H).

^{13}C NMR (125 MHz, $CDCl_3$): δ 191.1, 157.7, 149.5, 132.3, 130.9, 130.7, 130.6, 130.3, 130.1, 129.5, 129.1, 128.8, 126.2, 114.0, 113.2, 47.7, 46.7, 37.3, 33.3.

HRMS (m/z): $[M]^+$ calcd for $C_{22}H_{21}ON_2$, 343.1805; found 343.1801.

HPLC separation conditions: CHIRALPAK IB column (250 mm × 4.6 mm), hexane:THF = 90:10, 1.0 mL/min, T = 25 °C, λ = 254 nm, t_R (major) = 6.4 min, t_R (minor) = 8.9 min.

*Chiral HPLC spectrum of racemic **3k***

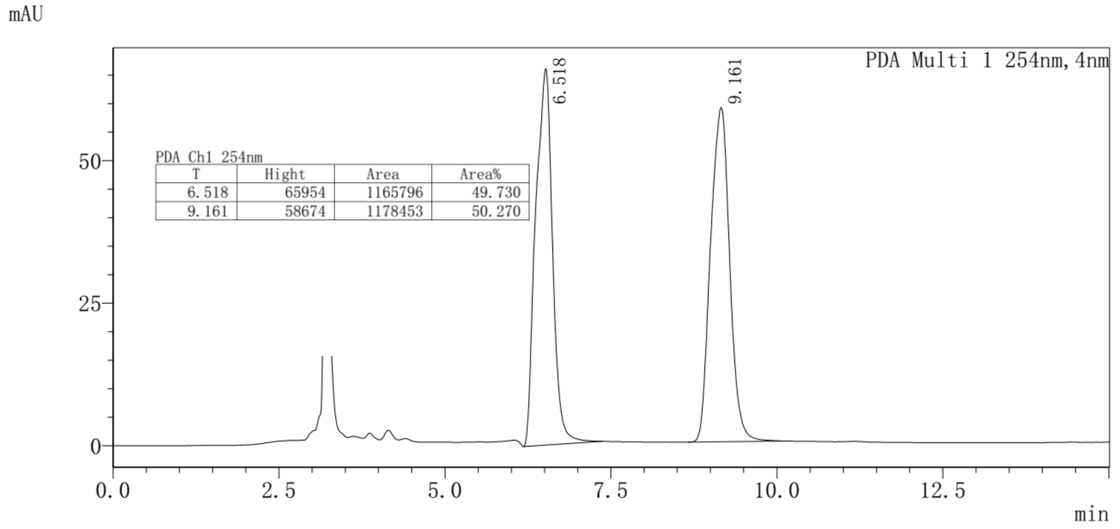

*Chiral HPLC spectrum of **3k***

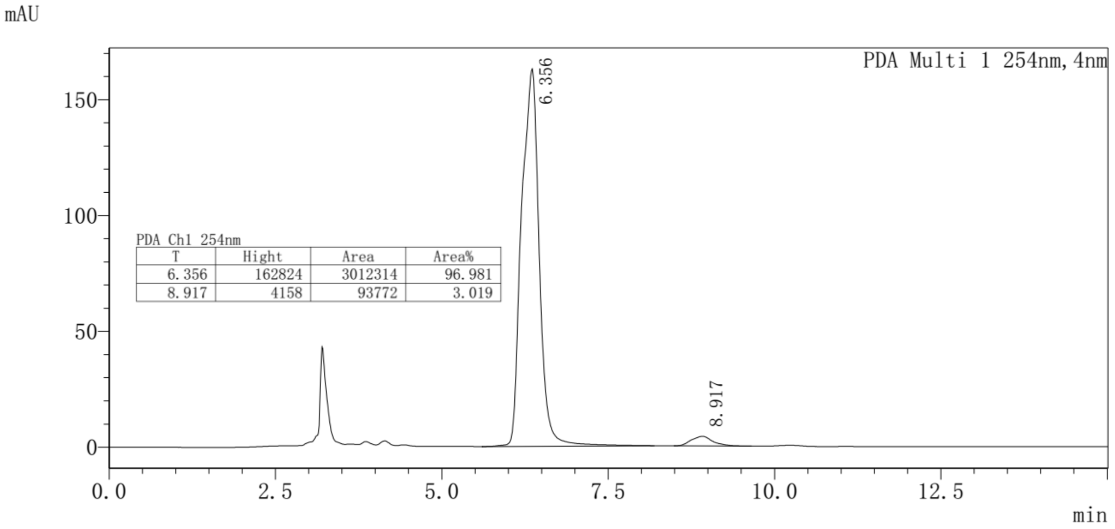

T	Hight	Area	Area%
6.356	162824	3012314	96.981
8.917	4158	93772	3.019

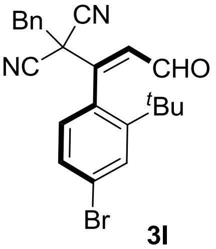

3l

(S_a) *(E)*-2-benzyl-2-(1-(4-bromo-2-(*tert*-butyl)phenyl)-3-oxoprop-1-en-1-yl)malononitrile

Yellow oil, 41.2 mg, 98% yield, 86% ee

^{1}H NMR (500 MHz, $CDCl_3$): δ 9.41 (d, J = 7.5 Hz, 1H), 7.87 (d, J = 2.0 Hz, 1H), 7.48 – 7.44 (m, 6H), 6.87 (d, J = 8.0 Hz, 1H), 6.52 (d, J = 7.5 Hz, 1H), 3.66 (d, J = 13.5 Hz, 1H), 3.40 (d, J = 13.5 Hz, 1H), 1.47 (s, 9H).

^{13}C NMR (125 MHz, $CDCl_3$): δ 190.5, 156.2, 151.8, 133.5, 132.7, 131.4, 130.7, 130.6, 129.6, 129.5, 129.2, 127.9, 125.4, 113.8, 113.0, 47.4, 46.8, 37.5, 33.0.

^{13}C NMR DEPT-135 (126 MHz, $CDCl_3$) δ = 190.5, 133.5, 132.7, 131.4, 130.7, 129.6, 129.5, 129.2, 33.03.

HRMS (m/z): $[M]^+$ calcd for $C_{23}H_{22}ON_2Br$, 421.0910; found 421.0910.

HPLC separation conditions: CHIRALPAK IB column (250 mm × 4.6 mm), hexane:THF = 80:20, 1.2 mL/min, T = 25 °C, λ = 254 nm, t_R (major) = 6.7 min, t_R (minor) = 14.6 min.

*Chiral HPLC spectrum of racemic **3l***

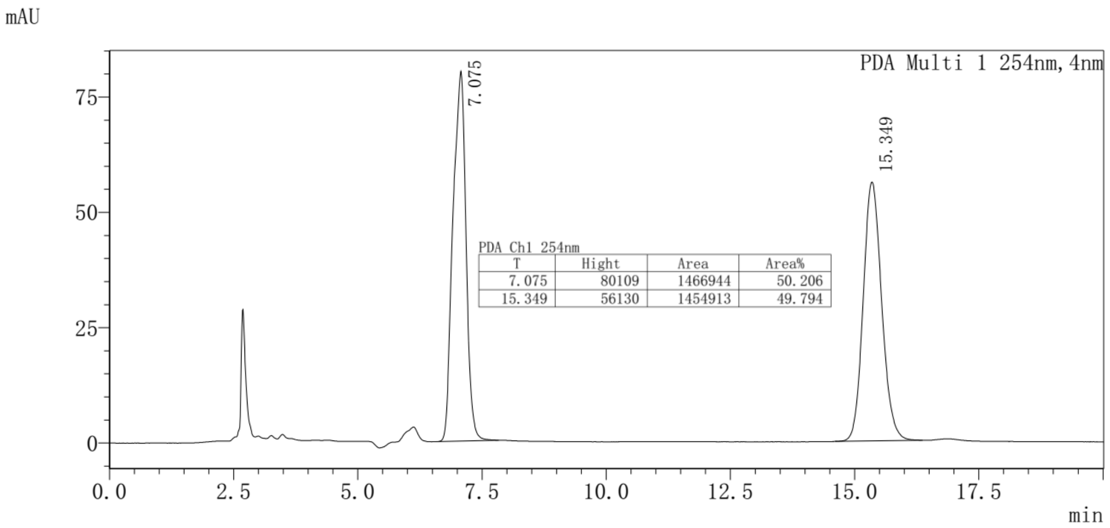

*Chiral HPLC spectrum of **3l***

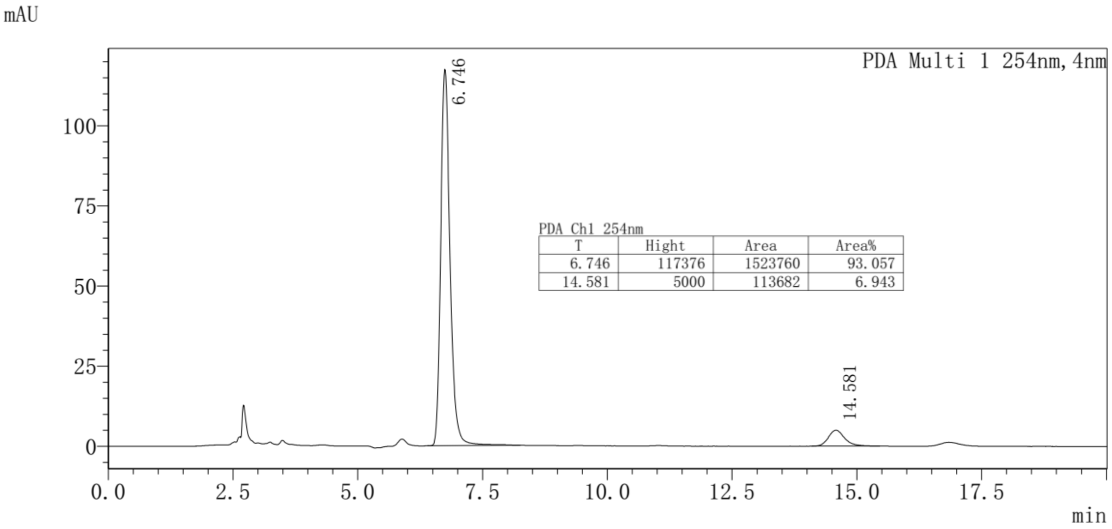

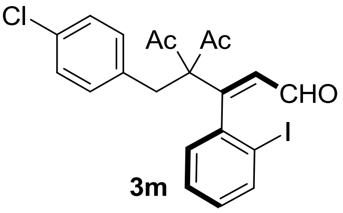

3m

(S_a)-(Z)-4-acetyl-4-(4-chlorobenzyl)-3-(2-iodophenyl)-5-oxohex-2-enal

White solid, m.p.: 120.9-121.7 °C, 44.2 mg, 92% yield, 94% ee.

^{1}H NMR (500 MHz, $CDCl_3$): δ 9.29 (d, J = 7.5 Hz, 1H), 8.01 (d, J = 8.0 Hz, 1H), 7.42 (t, J = 7.5 Hz, 1H), 7.19 – 7.12 (m, 3H), 6.99 (d, J = 8.5 Hz, 2H), 6.85 (d, J = 7.5 Hz, 1H), 6.23 (d, J = 7.5 Hz, 1H), 3.36 (d, J = 14.5 Hz, 1H), 2.90 (d, J = 14.5 Hz, 1H), 2.42 (s, 3H), 2.36 (s, 3H).

^{13}C NMR (125 MHz, $CDCl_3$): δ 204.7, 203.0, 191.3, 157.8, 140.1, 138.2, 133.9, 133.9, 132.9, 131.4, 130.8, 128.8, 128.6, 128.4, 100.5, 78.6, 35.5, 29.0, 27.6.

HRMS (m/z): $[M]^+$ calcd for $C_{21}H_{19}O_3ClI$, 481.0062; found 481.0066.

HPLC separation conditions: CHIRALPAK IA column (250 mm × 4.6 mm), hexane:*i*-PrOH = 80:20,

1.0 mL/min, T = 25 °C, λ = 254 nm, t_R (major) = 8.1 min, t_R (minor) = 10.4 min.

*Chiral HPLC spectrum of racemic **3m***

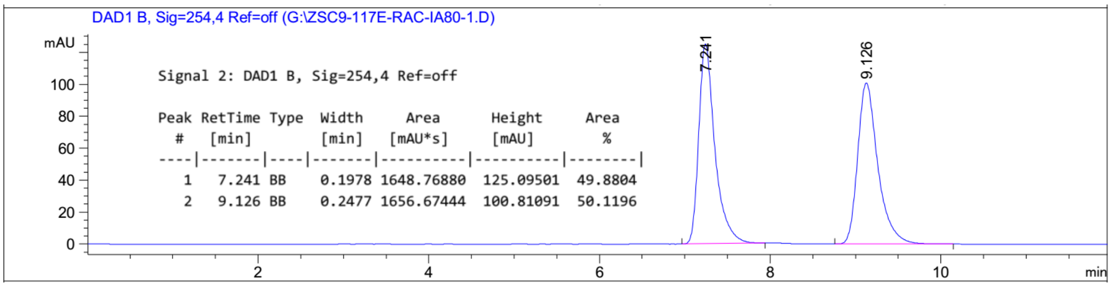

*Chiral HPLC spectrum of **3m***

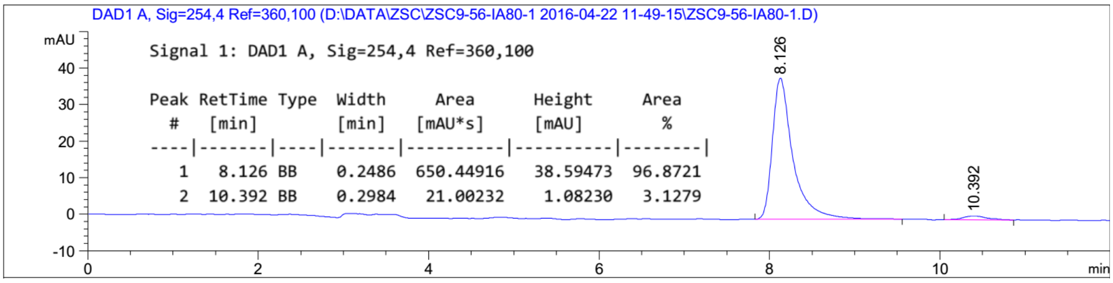

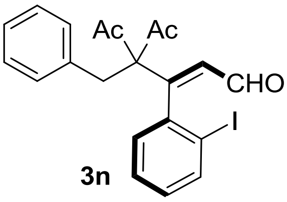

3n

(S_a)-(*Z*)-4-acetyl-4-benzyl-3-(2-iodophenyl)-5-oxohex-2-enal

White solid, m.p.: 149.5-151.1, 39.9 mg, 89% yield, 94% ee

^{1}H NMR (500 MHz, $CDCl_3$): δ 9.30 (d, J = 7.5 Hz, 1H), 8.01 (d, J = 8.0 Hz, 1H), 7.42 (t, J = 7.5 Hz, 1H), 7.22 – 7.10 (m, 4H), 7.07 – 7.00 (m, 2H), 6.87 (d, J = 7.5 Hz, 1H), 6.21 (d, J = 7.5 Hz, 1H), 3.43 (d, J = 14.5 Hz, 1H), 2.95 (d, J = 14.5 Hz, 1H), 2.43 (s, 3H), 2.34 (s, 3H).

^{13}C NMR (125 MHz, $CDCl_3$): δ 204.9, 203.1, 191.5, 158.2, 140.1, 138.4, 135.5, 133.9, 130.8, 130.0, 128.9, 128.6, 128.3, 127.1, 100.7, 78.7, 36.3, 29.1, 27.8.

HRMS (m/z): $[M]^+$ calcd for $C_{21}H_{20}O_3I$, 447.0452; found 447.0451.

HPLC separation conditions: CHIRALPAK IA column (250 mm × 4.6 mm), hexane:*i*-PrOH = 80:20, 1.0 mL/min, T = 25 °C, λ = 254 nm, t_R (major) = 6.5 min, t_R (minor) = 8.6 min.

*Chiral HPLC spectrum of racemic **3n***

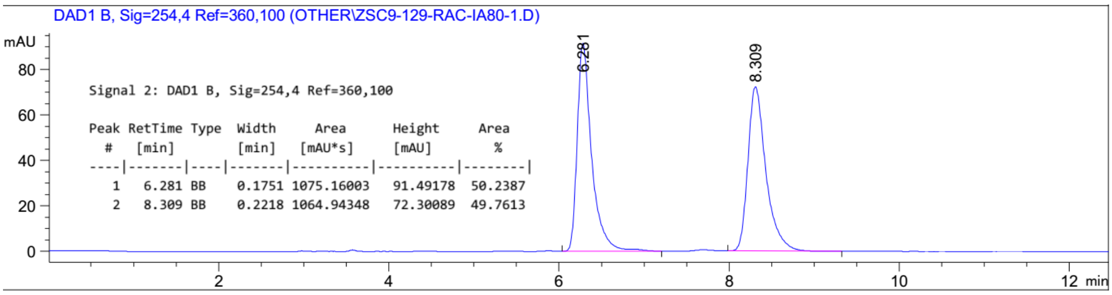

*Chiral HPLC spectrum of **3n***

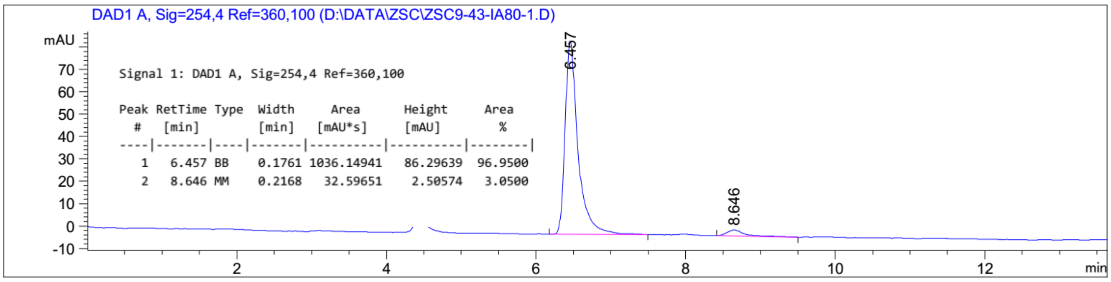

*Chiral HPLC spectrum of **3n*** (After recrystallization)

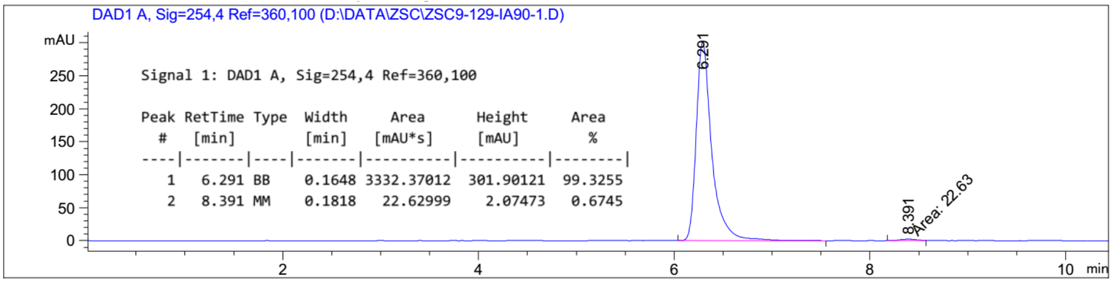

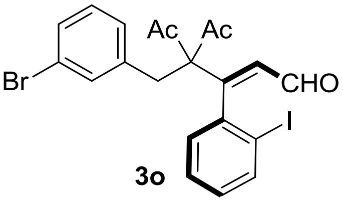

3o

(S_a)-(Z)-4-acetyl-4-(3-bromobenzyl)-3-(2-iodophenyl)-5-oxohex-2-enal

White solid, m.p.: 95.0-97.0 °C, 47.2 mg, 90% yield, 93% ee.

^{1}H NMR (400 MHz, $CDCl_3$): δ 9.29 (d, J = 7.6 Hz, 1H), 8.01 (d, J = 8.0 Hz, 1H), 7.42 (t, J = 8.0 Hz, 1H), 7.29 (d, J = 8.0 Hz, 1H), 7.21 (s, 1H), 7.15 (td, J = 8.0, 1.6 Hz, 1H), 7.05 (t, J = 8.0 Hz, 1H), 6.96 (d, J = 8.0 Hz, 1H), 6.85 (dd, J = 8.0, 1.6 Hz, 1H), 6.23 (d, J = 7.6 Hz, 1H), 3.37 (d, J = 14.8 Hz, 1H), 2.89 (d, J = 14.8 Hz, 1H), 2.43 (s, 3H), 2.38 (s, 3H).

^{13}C NMR (125 MHz, $CDCl_3$): δ 204.6, 202.9, 191.3, 157.8, 140.1, 138.1, 137.8, 134.0, 133.1, 130.8, 130.2, 129.8, 128.8, 128.6, 128.6, 122.2, 100.6, 78.5, 35.7, 29.1, 27.6.

^{13}C NMR- DEPT 135 (125 MHz, $CDCl_3$): δ 191.3, 140.1, 134.0, 133.1, 130.9, 130.3, 129.8, 128.9, 128.7, 128.6, 35.8, 29.1, 27.7.

HRMS (m/z): $[M]^+$ calcd for $C_{21}H_{19}O_3BrI$, 524.9557; found 524.9564

HPLC separation conditions: CHIRALPAK IA column (250 mm × 4.6 mm), hexane:*i*-PrOH = 80:20, 1.0 mL/min, T = 25 °C, λ = 254 nm, t_R (major) = 6.3 min, t_R (minor) = 7.6 min.

*Chiral HPLC spectrum of racemic **3o***

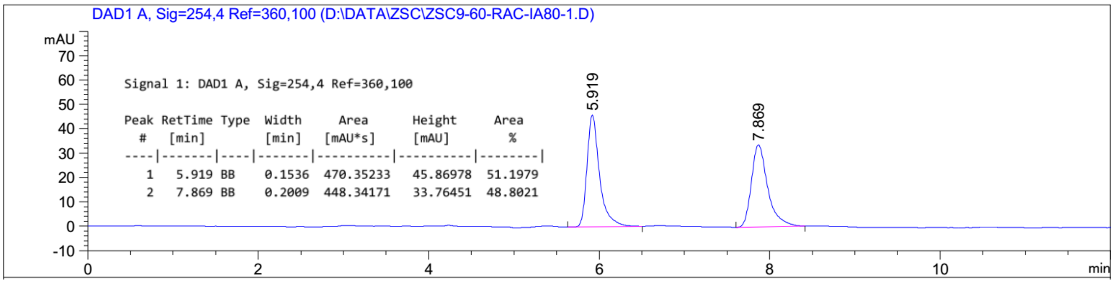

Chiral HPLC spectrum of racemic ***3o***

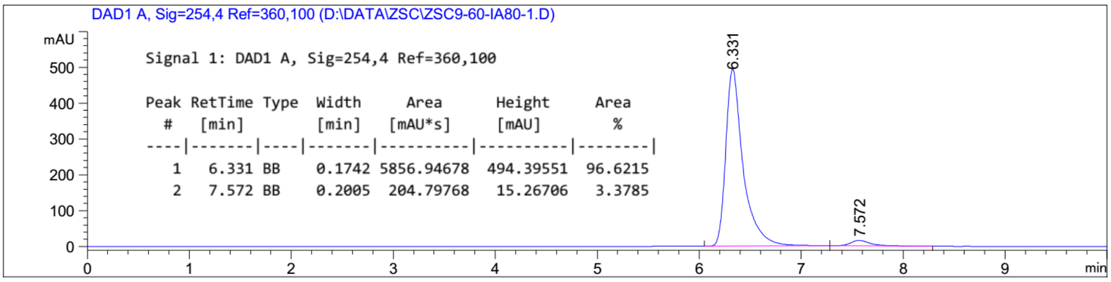

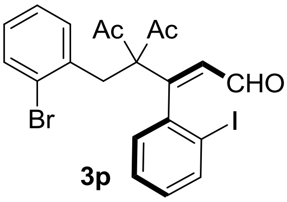

(S_a)-(Z)-4-acetyl-4-(2-bromobenzyl)-3-(2-iodophenyl)-5-oxohex-2-enal

White solid, m.p.: 125.9-131.7 °C, 49.8 mg, 95% yield, 95% ee.

^{1}H NMR (500 MHz, $CDCl_3$): δ 9.32 (d, J = 7.5 Hz, 1H), 8.04 (dd, J = 8.0, 1.5 Hz, 1H), 7.49 (dd, J = 8.0, 1.5 Hz, 1H), 7.41 (td, J = 7.5, 1.5 Hz, 1H), 7.15 (td, J = 7.5, 1.5 Hz, 1H), 7.11 (td, J = 7.5, 1.5 Hz, 1H), 7.01 (td, J = 7.5, 1.5 Hz, 1H), 6.89 – 6.85 (m, 2H), 6.24 (d, J = 7.5 Hz, 1H), 3.50 (d, J = 16.0 Hz, 1H), 3.27 (d, J = 16.0 Hz, 1H), 2.50 (s, 3H), 2.30 (s, 3H).

^{13}C NMR (125 MHz, $CDCl_3$): δ 205.1, 203.6, 191.5, 157.9, 140.2, 138.1, 135.0, 133.6, 133.0, 130.8, 130.0, 128.8, 128.5, 128.3, 127.5, 125.8, 100.8, 77.4, 35.5, 28.6, 28.2.

^{13}C NMR- DEPT 135 (125 MHz, $CDCl_3$): δ 191.5, 140.2, 133.6, 133.0, 130.8, 130.0, 128.8, 128.5, 128.3, 127.5, 35.5, 28.6, 28.2.

HRMS (m/z): $[M]^+$ calcd for $C_{21}H_{19}O_3BrI$, 524.9557, found 524.9563.

HPLC separation conditions: CHIRALPAK IA column (250 mm × 4.6 mm), hexane:*i*-PrOH = 80:20, 1.0 mL/min, T = 25 °C, λ = 254 nm, t_R (major) = 5.9 min, t_R (minor) = 7.8 min.

Chiral HPLC spectrum of racemic ***3p***

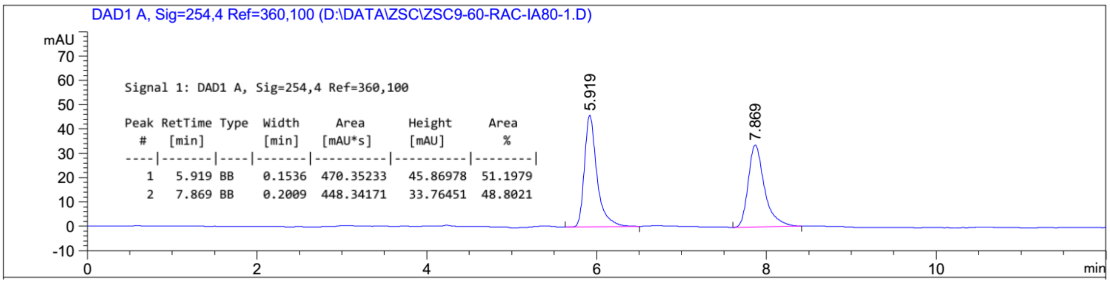

Chiral HPLC spectrum of ***3p***

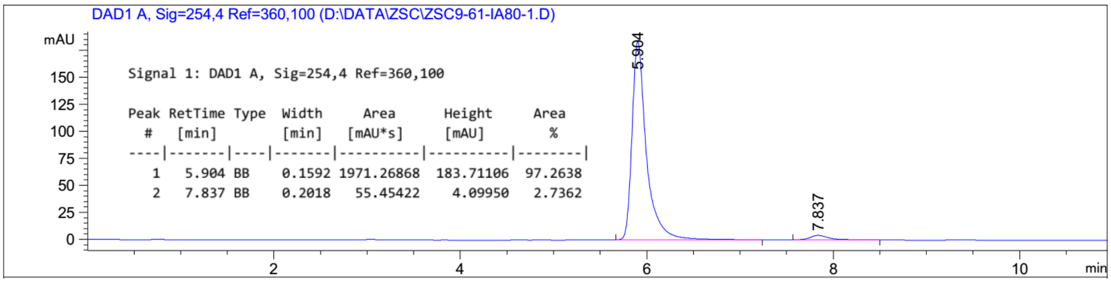

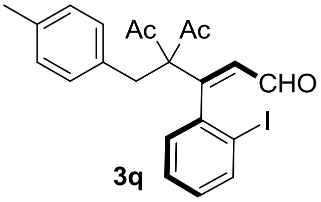

3q

(S_a)- (Z)-4-acetyl-3-(2-iodophenyl)-4-(4-methylbenzyl)-5-oxohex-2-enal

White solid, m.p.: 129.0-131.3 °C, 45.8 mg, 99% yield, 94% ee.

^{1}H NMR (400 MHz, $CDCl_3$): δ 9.29 (d, J = 7.6 Hz, 1H), 8.00 (d, J = 8.0 Hz, 1H), 7.41 (t, J = 7.6 Hz, 1H), 7.14 (t, J = 7.6 Hz, 1H), 6.98 (d, J = 8.0 Hz, 2H), 6.92 (d, J = 8.0 Hz, 2H), 6.87 (d, J = 7.6 Hz, 1H), 6.21 (d, J = 7.6 Hz, 1H), 3.38 (d, J = 14.4 Hz, 1H), 2.92 (d, J = 14.4 Hz, 1H), 2.41 (s, 3H), 2.35 (s, 3H), 2.25 (s, 3H).

^{13}C NMR (100 MHz, $CDCl_3$): δ 204.9, 203.1, 191.4, 158.2, 140.0, 138.5, 136.6, 133.8, 132.2, 130.7, 129.9, 129.0, 128.9, 128.5, 100.6, 78.7, 36.0, 29.1, 27.8, 20.9.

^{13}C NMR- DEPT 135 (100 MHz, $CDCl_3$): δ 191.5, 140.1, 133.9, 130.7, 129.9, 129.0, 129.0, 128.5, 36.0, 29.1, 27.8, 21.0.

HRMS (m/z): $[M]^+$ calcd for $C_{22}H_{22}O_3I$, 461.0608; found 461.0606.

HPLC separation conditions: CHIRALPAK IA column (250 mm × 4.6 mm), hexane:*i*-PrOH = 80:20, 1.0 mL/min, T = 25 °C, λ = 254 nm, t_R (major) = 6.3 min, t_R (minor) = 8.7 min.

Chiral HPLC spectrum of racemic ***3q***

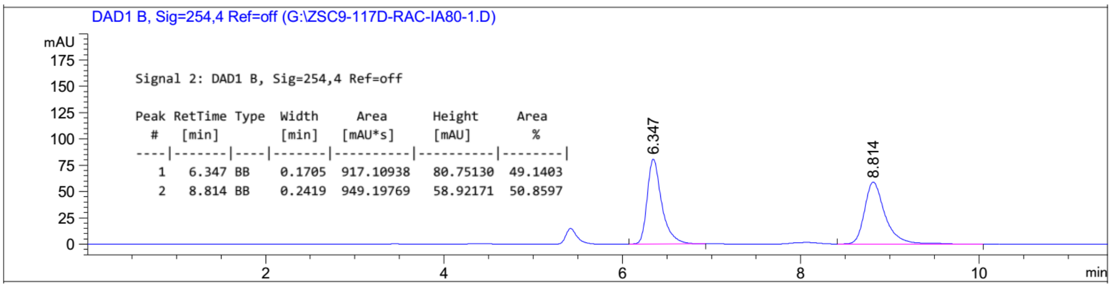

Chiral HPLC spectrum of ***3q***

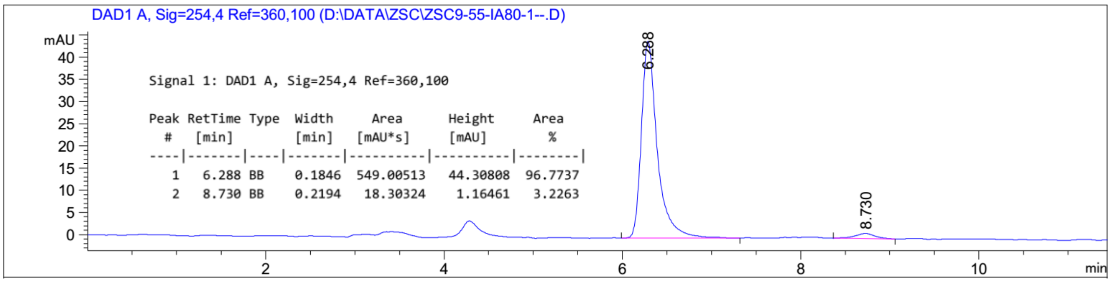

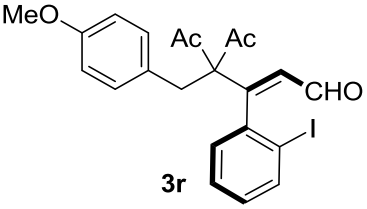

3r

(S_a)- (Z)-4-acetyl-3-(2-iodophenyl)-4-(4-methoxybenzyl)-5-oxohex-2-enal

White solid, m.p.: 111.7-112.7 °C, 45.2 mg, 95% yield, 94% ee.

^{1}H NMR (500 MHz, $CDCl_3$): δ 9.29 (d, J = 7.5 Hz, 1H), 8.00 (d, J = 8.0 Hz, 1H), 7.41 (t, J = 7.5 Hz, 1H), 7.14 (t, J = 7.5 Hz, 1H), 6.97 (d, J = 8.5 Hz, 2H), 6.86 (d, J = 7.5 Hz, 1H), 6.71 (d, J = 8.5 Hz, 2H), 6.21 (d, J = 7.5 Hz, 1H), 3.73 (s, 3H), 3.36 (d, J = 14.5 Hz, 1H), 2.90 (d, J = 14.5 Hz, 1H), 2.41 (s, 3H), 2.35 (s, 3H).
^{13}C NMR- DEPT 135 (125 MHz, $CDCl_3$): δ 205.0, 203.2, 191.4, 158.4, 158.2, 140.0, 138.4, 133.8, 131.1, 130.6, 128.8, 128.5, 127.2, 113.6, 100.6, 78.8, 55.0, 35.5, 29.0, 27.7.
HRMS (m/z): $[M]^+$ calcd for $C_{22}H_{22}O_4I$, 477.0557; found 477.0560.
HPLC separation conditions: CHIRALPAK IA column (250 mm × 4.6 mm), hexane:*i*-PrOH = 80:20, 1.0 mL/min, T = 25 °C, λ = 254 nm, t_R (major) = 8.4 min, t_R (minor) = 11.8 min.

*Chiral HPLC spectrum of racemic **3r***

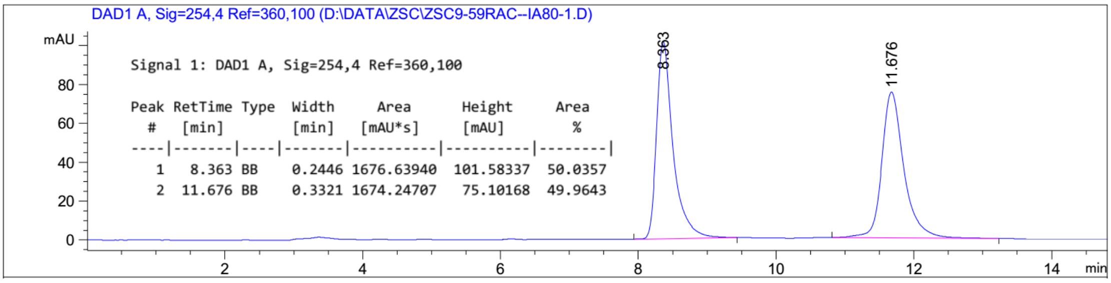

*Chiral HPLC spectrum of **3r***

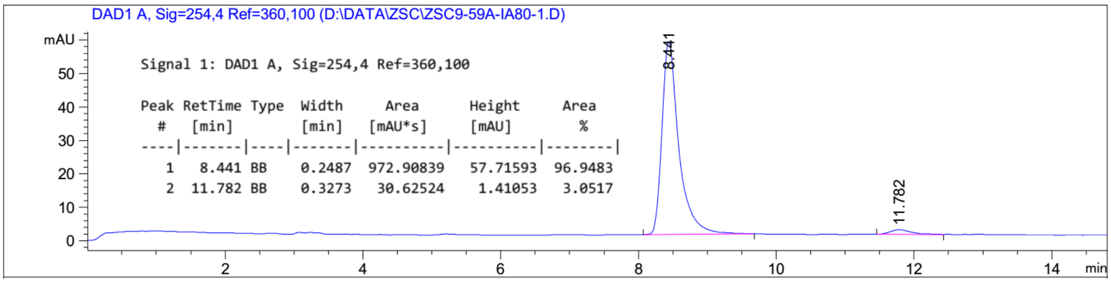

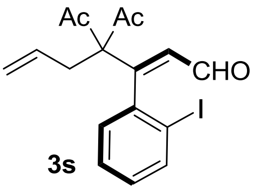

(S_a)- (Z)-4,4-diacetyl-3-(2-iodophenyl)hepta-2,6-dienal
Colorless oil, 34.5 mg, 87% yield, 94% ee.
^{1}H NMR (500 MHz, $CDCl_3$): δ 9.29 (d, J = 7.5 Hz, 1H), 7.98 (d, J = 8.0 Hz, 1H), 7.39 (t, J = 7.5 Hz, 1H), 7.13 (td, J = 8.0, 1.5 Hz, 1H), 6.86 (dd, J = 7.5, 1.5 Hz, 1H), 6.20 (d, J = 8.0 Hz, 1H), 5.53 (dddd, J = 17.0, 10.5, 9.0, 5.0 Hz, 1H), 5.05 (d, J = 17.0 Hz, 1H), 5.01 (d, J = 10.5 Hz, 1H), 2.62 – 2.54 (m, 1H), 2.47 (s, 3H), 2.42 (dd, J = 14.5, 9.0 Hz, 1H), 2.36 (s, 3H).
^{13}C NMR (125 MHz, $CDCl_3$): δ 205.2, 202.8, 191.4, 158.3, 140.0, 138.5, 133.9, 132.5, 130.7, 128.8, 128.5, 119.1, 100.1, 77.2, 35.4, 28.2, 27.6.
HRMS (m/z): $[M]^+$ calcd for $C_{17}H_{18}O_3I$, 397.0295; found 397.0291
HPLC separation conditions: CHIRALPAK IA column (250 mm × 4.6 mm), hexane:*i*-PrOH = 90:10, 1.0 mL/min, T = 25 °C, λ = 254 nm, t_R (major) = 7.1 min, t_R (minor) = 8.0 min.

Chiral HPLC spectrum of racemic ***3s***

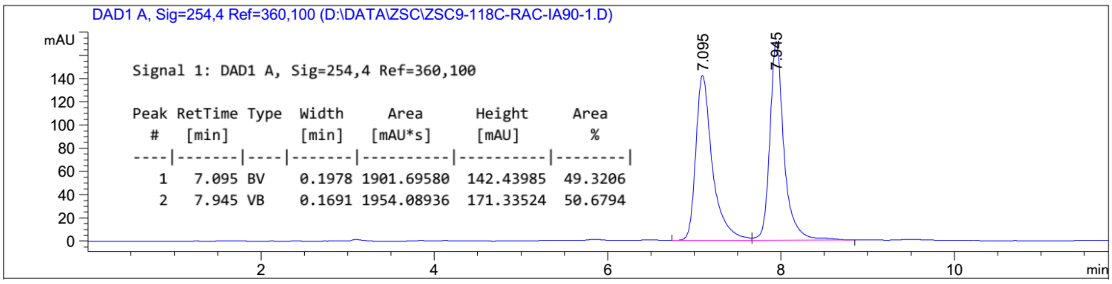

Chiral HPLC spectrum of ***3s***

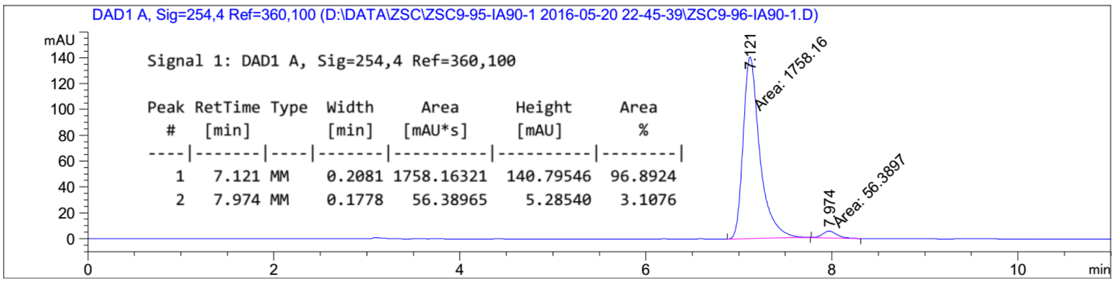

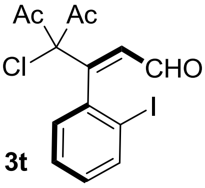

3t

(S_a)- (E)-4-acetyl-4-chloro-3-(2-iodophenyl)-5-oxohex-2-enal

Colorless oil, 19.1 mg, 49% yield, 90% ee.

^{1}H NMR (400 MHz, $CDCl_3$): δ 9.28 (d, J = 7.6 Hz, 1H), 7.96 (dd, J = 8.0, 1.2 Hz, 1H), 7.39 (td, J = 7.6, 1.2 Hz, 1H), 7.17 (dd, J = 8.0, 1.6 Hz, 1H), 7.12 (td, J = 7.6, 1.6 Hz, 1H), 6.10 (d, J = 7.6 Hz, 1H), 2.68 (s, 3H), 2.36 (s, 3H).

^{13}C NMR (100 MHz, $CDCl_3$): δ 199.4, 197.4, 191.3, 153.9, 139.5, 138.3, 133.8, 130.6, 129.8, 128.4, 101.3, 81.8, 27.0, 26.7.

HRMS (m/z): $[M]^+$ calcd for $C_{14}H_{13}O_3ClI$, 390.9592; found 390.9587.

HPLC separation conditions: CHIRALPAK IA column (250 mm × 4.6 mm), hexane:*i*-PrOH = 90:10, 1.0 mL/min, T = 25 °C, λ = 254 nm, t_R (major) = 7.0 min, t_R (minor) = 7.9 min.

Chiral HPLC spectrum of racemic ***3t***

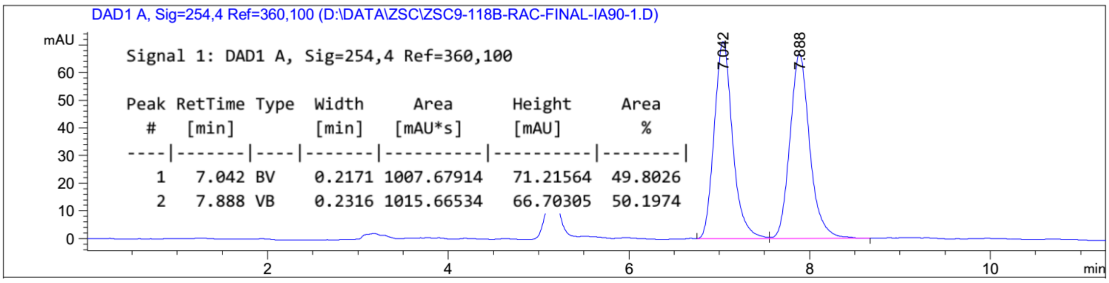

Chiral HPLC spectrum of ***3t***

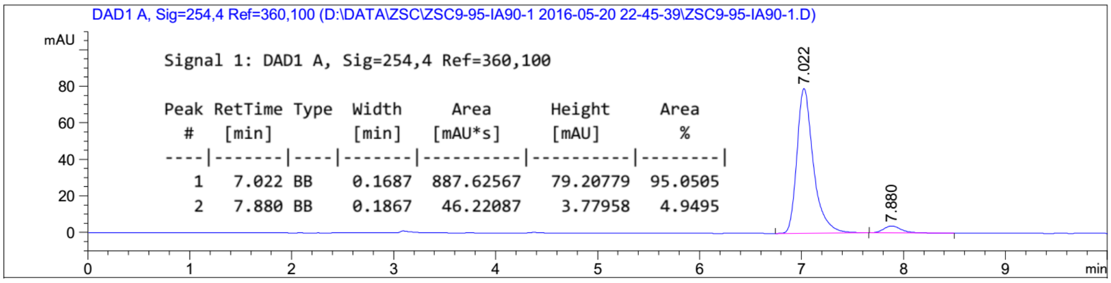

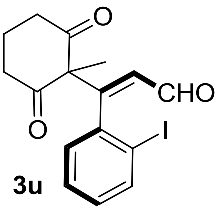

3u

(*Sa*)-(*Z*)-3-(2-iodophenyl)-3-(1-methyl-2,6-dioxocyclohexyl)acrylaldehyde

Colorless oil, 21.7 mg, 57% yield (Z/E = 90/10), 90% ee.

^{1}H NMR (400 MHz, $CDCl_3$): δ 9.26 (d, J = 7.6 Hz, 1H), 7.96 (d, J = 8.0 Hz, 1H), 7.41 (t, J = 7.6 Hz, 1H), 7.20 (dd, J = 8.0, 1.6 Hz, 1H), 7.10 (td, J = 8.0, 1.6 Hz, 1H), 6.14 (d, J = 8.0 Hz, 1H), 3.10 (ddd, J = 15.4, 11.6, 6.0 Hz, 1H), 2.71 (dt, J = 15.4, 4.4 Hz, 1H), 2.64 – 2.49 (m, 2H), 2.14 – 2.05 (m, 1H), 1.83 – 1.71 (m, 1H), 1.62 (s, 3H).

^{13}C NMR (100 MHz, $CDCl_3$): δ 206.2, 205.1, 191.9, 160.5, 140.4, 138.9, 131.8, 130.8, 129.4, 128.4, 100.9, 72.8, 40.1, 39.1, 21.5, 17.7.

HRMS (m/z): $[M]^+$ calcd for $C_{16}H_{16}O_3I$, 383.0139; found 383.0134.

HPLC separation conditions: CHIRALPAK IC column (250 mm × 4.6 mm), hexane:*i*-PrOH = 80:20, 1.0 mL/min, T = 25 °C, λ = 254 nm, t_R (minor) = 18.1 min, t_R (major) = 19.4 min.

Chiral HPLC spectrum of racemic ***3u***

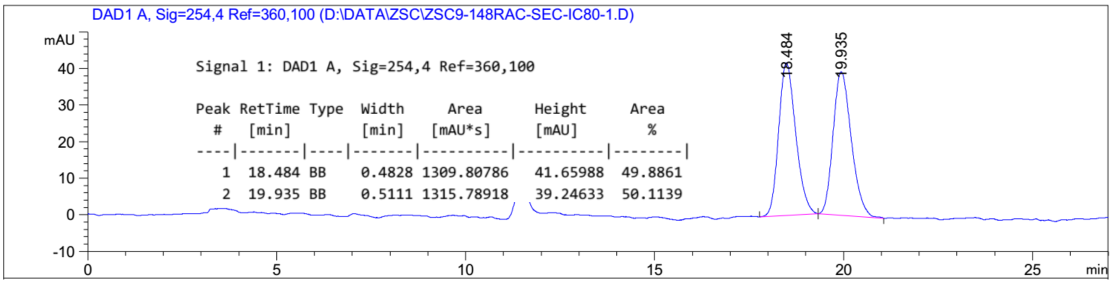

Chiral HPLC spectrum of ***3u***

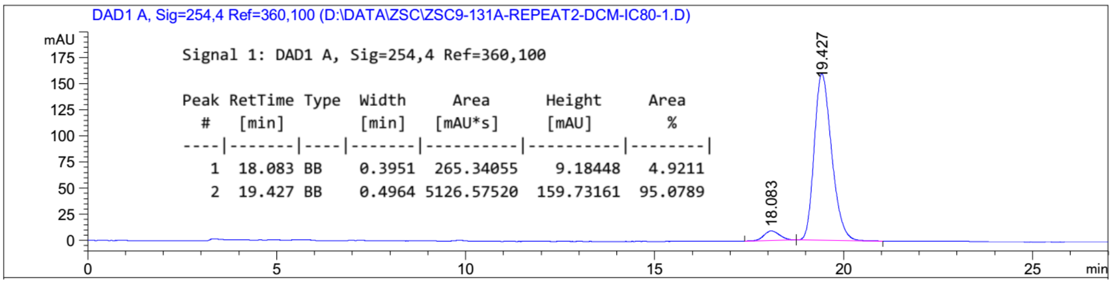

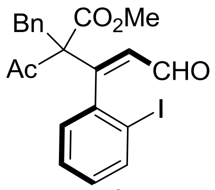

3v

Methyl (*Z*)-2-acetyl-2-benzyl-3-(2-iodophenyl)-5-oxopent-3-enoate

Colorless oil, 43.0 mg, 93% yield, 2.1:1 dr, 85% ee (major), 96% ee (minor).

^{1}H NMR (400 MHz, $CDCl_3$): δ (major) 9.25 (d, J = 7.6 Hz, 1H), 7.98 (d, J = 8.0 Hz, 1H), 7.42 (dt, J = 7.6, 1.2 Hz, 1H), 7.25 – 7.09 (m, 6H), 7.02 (dd, J = 8.0, 1.6 Hz, 1H), 6.28 (d, J = 7.6 Hz, 1H), 3.60 (s, 3H), 3.32 (d, J = 13.2 Hz, 1H), 3.23 (d, J = 13.2 Hz, 1H), 2.32 (s, 3H). δ (minor) 9.28 (d, J = 7.6 Hz, 1H), 7.98 (d, J = 8.0 Hz, 1H), 7.41 (dt, J = 7.6, 0.8 Hz, 1H), 7.25 – 7.09 (m, 7H), 6.23 (d, J = 7.6 Hz, 1H), 3.62 (s, 3H), 3.51 (d, J = 13.6 Hz, 1H), 3.46 (d, J = 13.6 Hz, 1H), 2.17 (s, 3H).

^{13}C NMR (100 MHz, $CDCl_3$): δ (major) 201.5, 192.1, 169.8, 158.8, 139.9, 135.6, 134.0, 130.6, 130.4, 129.2, 128.3, 128.2, 127.2, 100.8, 72.6, 52.4, 39.1, 28.9. δ (minor) 202.2, 192.1, 169.7, 158.6, 139.3, 135.5, 134.0, 130.5, 130.3, 129.3, 128.3, 128.2, 127.3, 100.7, 72.3, 52.6, 40.7, 30.2.

HRMS (m/z): $[M]^+$ calcd for $C_{21}H_{20}O_4I$, 463.0401; found 463.0398.

HPLC separation conditions: CHIRALPAK AD-3 column (250 mm × 4.6 mm), hexane:*i*-PrOH = 90:10, 1.0 mL/min, T = 25 °C, λ = 254 nm, t_R (major$_1$) = 8.2 min, t_R (minor$_1$) = 8.7 min; t_R (major$_2$) = 9.3 min, t_R (minor$_1$) = 10.6 min

*Chiral HPLC spectrum of racemic **3v***

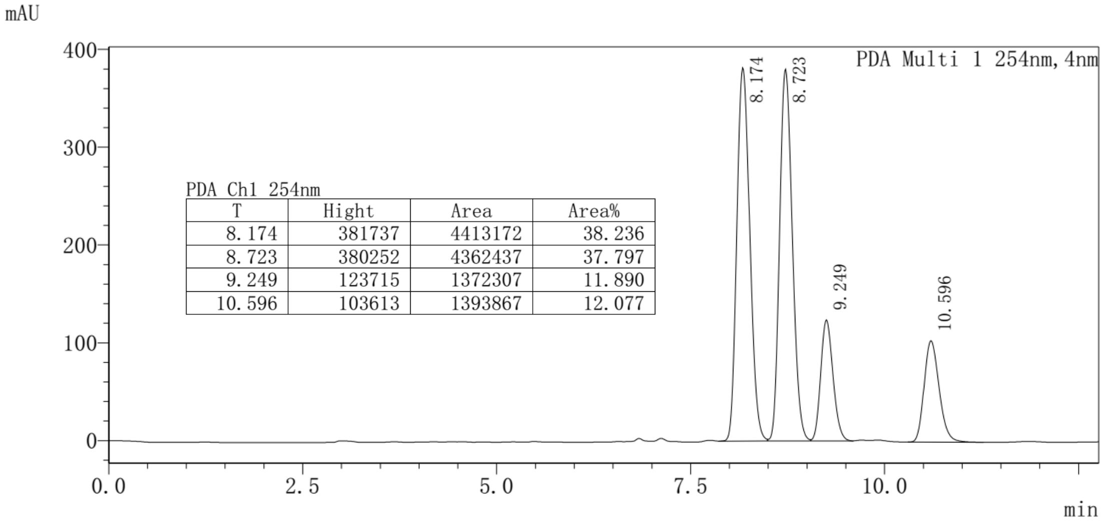

*Chiral HPLC spectrum of **3v***

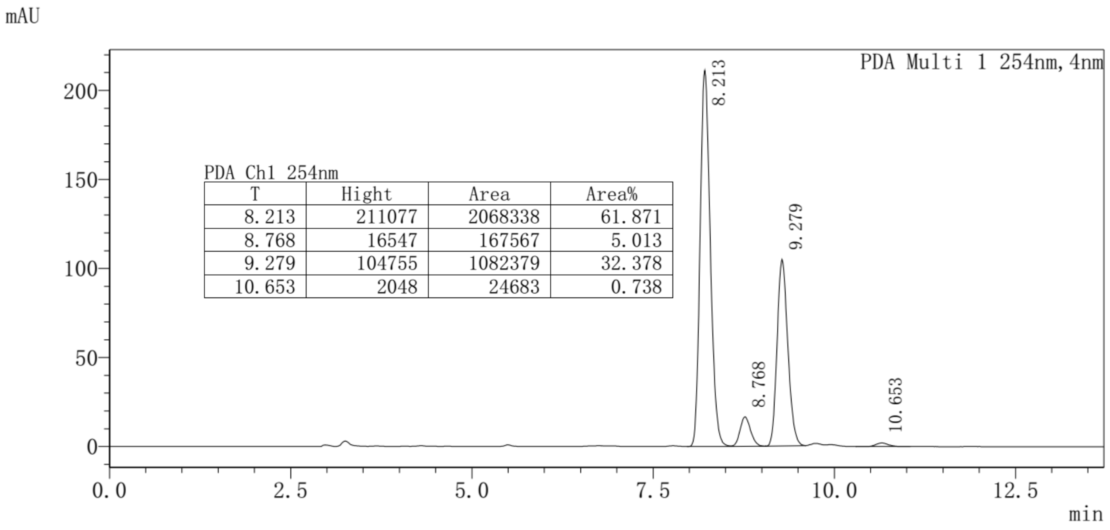

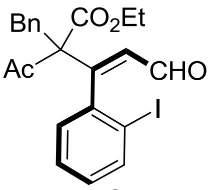

3w

Ethyl (*Z*)-2-acetyl-2-benzyl-3-(2-iodophenyl)-5-oxopent-3-enoate

Colorless oil, 46.2 mg, 97% yield, 2.3:1 dr, 86% ee (major), 94% ee (minor).

^{1}H NMR (500 MHz, $CDCl_3$): δ (major) 9.26 (d, J = 8.0 Hz, 1H), 7.98 (d, J = 8.0 Hz, 1H), 7.41 (t, J = 8.0 Hz, 1H), 7.24 – 7.10 (m, 6H), 7.01 (d, J = 7.5 Hz, 1H), 6.27 (d, J = 7.7 Hz, 1H), 3.98 (q, J = 7.5 Hz, 2H), 3.35 (d, J = 13.5 Hz, 1H), 3.24 (d, J = 13.5 Hz, 1H), 2.34 (s, 3H). 1.12 (t, J = 7.0 Hz, 1H) δ (minor) 9.28 (d, J = 8.0 Hz, 1H), 7.98 (d, *J* = 8.0 Hz, 1H), 7.41 (t, J = 8.0 Hz, 1H), 7.24 – 7.10 (m, 7H), 6.22 (d, J = 8.0 Hz, 1H), 4.03 (q, J = 7.5 Hz, 2H), 3.54 (d, J = 13.5 Hz, 1H), 3.50 (d, J = 13.5 Hz, 1H), 2.19 (s, 3H), 1.13 (t, J = 7.0 Hz, 1H).

^{13}C NMR (125 MHz, $CDCl_3$): δ (major) 201.5, 192.1, 169.2, 158.9, 139.9, 135.7, 134.1, 130.7, 130.3 129.1, 128.3, 128.12, 127.2 100.9, 72.6, 62.0, 39.1, 28.8, 13.7. δ (minor) 202.1, 192.1, 169.1, 140.2, 139.4, 135.7, 134.1, 130.6, 130.3, 129.2, 128.3, 128.14, 127.3, 100.8, 72.3, 62.1, 40.8, 30.1, 13.7.

HRMS (m/z): $[M]^+$ calcd for $C_{22}H_{22}O_4I$, 477.0557; found 477.0556.

HPLC separation conditions: CHIRALPAK AD-3 column (250 mm × 4.6 mm), hexane:*i*-PrOH = 98:2, 1.0 mL/min, T = 25 °C, λ = 254 nm, t_R (major$_1$) = 18.4 min, t_R (minor$_1$) = 21.5 min; t_R (major$_2$) = 23.4 min, t_R (minor$_1$) = 28.2 min

*Chiral HPLC spectrum of racemic **3w***

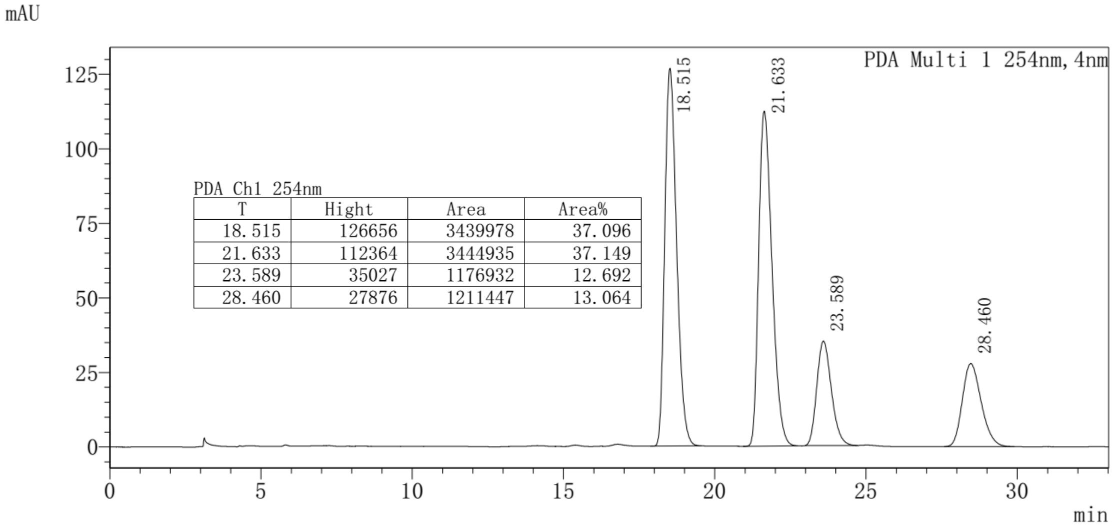

Chiral HPLC spectrum of ***3w***

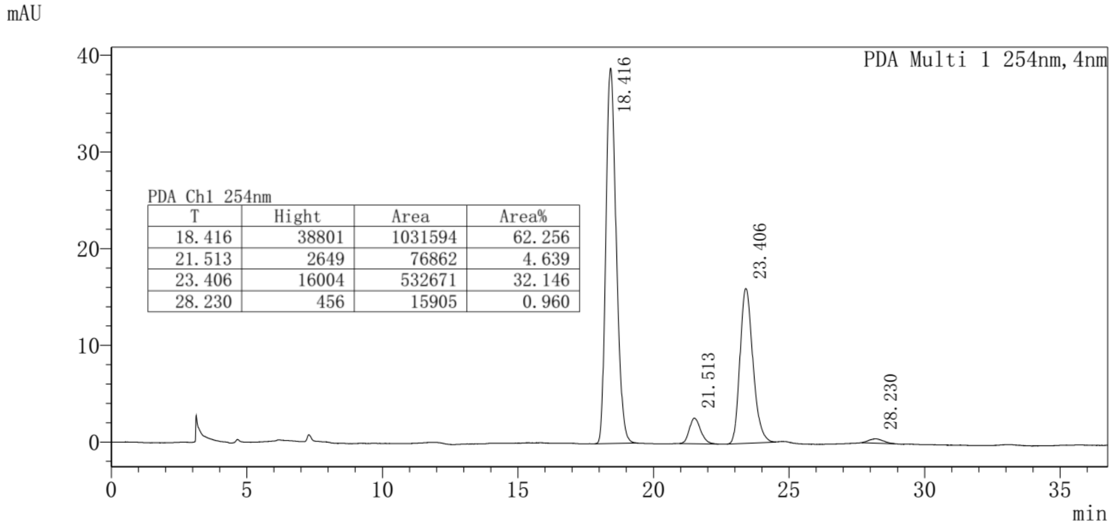

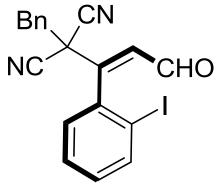

3x

(S_a) (Z)-2-benzyl-2-(1-(2-iodophenyl)-3-oxoprop-1-en-1-yl)malononitrile

Yellow oil, 82.0 mg (0.2 mmol), 99% yield, 81% ee

^{1}H NMR (500 MHz, $CDCl_3$): δ 9.33 (d, J = 7.5 Hz, 1H), 8.08 (d, J = 8.0 Hz, 1H), 7.56 (t, J = 7.5 Hz, 1H), 7.45 – 7.42 (m, 5H), 7.34 (d, J = 8.0 Hz, 1H), 7.28 (t, J = 7.5 Hz, 1H), 6.78 (d, J = 7.5 Hz, 1H), 3.61 (d, J = 13.5 Hz, 1H), 3.33 (d, J = 13.5 Hz, 1H).

^{13}C NMR (125 MHz, $CDCl_3$): δ 190.4, 152.9, 140.4, 136.2, 133.2, 132.0, 130.9, 130.6, 130.2, 129.4, 129.1, 128.9, 113.0, 112.6, 100.5, 46.2, 44.2.

^{13}C NMR-DEPT135 (125 MHz, $CDCl_3$) δ 190.4, 140.3, 133.2, 132.0, 130.6, 130.2, 129.4, 129.1, 128.9, 44.2.

HRMS (m/z): $[M]^+$ calcd for $C_{19}H_{14}ON_2I$, 413.0145; found 413.0144.

HPLC separation conditions: CHIRALPAK IA column (250 mm × 4.6 mm), hexane:THF = 95:5, 1.2 mL/min, T = 25 °C, λ = 254 nm, t_R (major) = 30.8 min, t_R (minor) = 36.1 min.

*Chiral HPLC spectrum of racemic **3x***

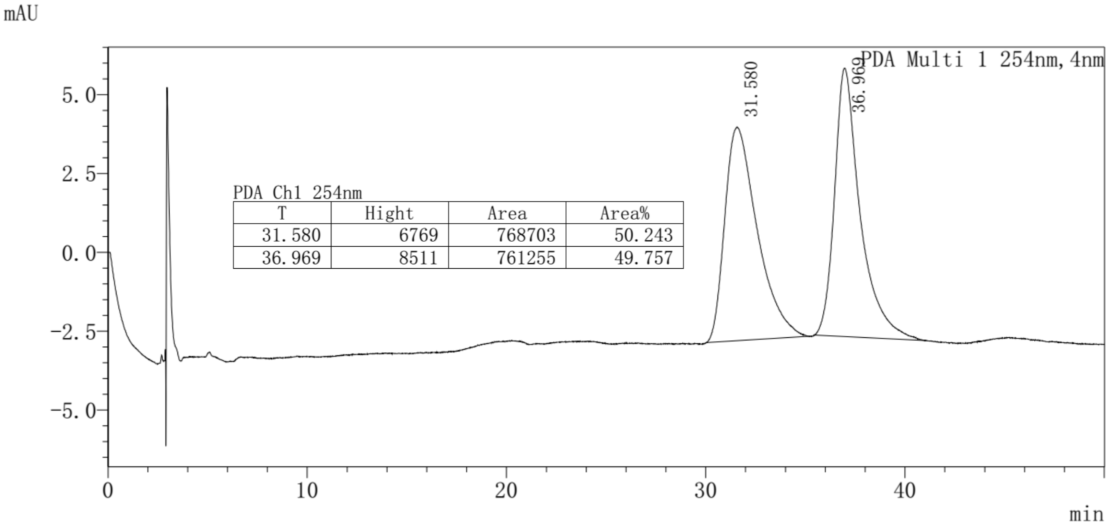

*Chiral HPLC spectrum of **3x***

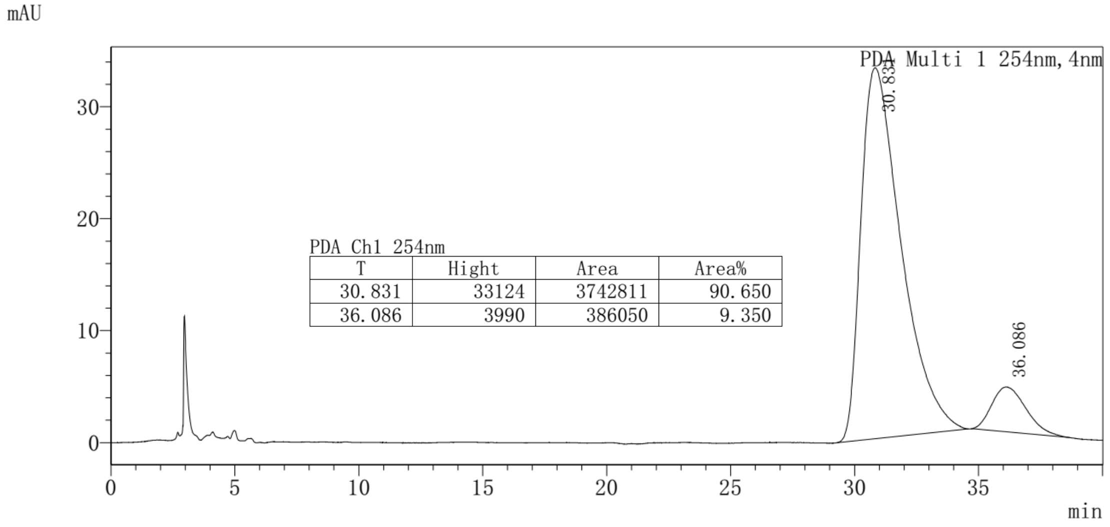

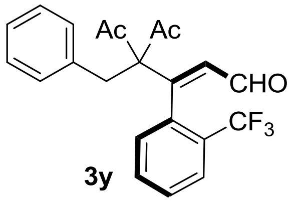

3y

(S_a)-(E)-4-acetyl-4-benzyl-5-oxo-3-(2-(trifluoromethyl)phenyl)hex-2-enal

White solid, m.p.: 133.0-133.7 °C, 330.1 mg (1 mmol), 85% yield, 89% ee. 99% ee after recrystallization.

^{1}H NMR (400 MHz, $CDCl_3$): δ 9.17 (d, J = 7.6 Hz, 1H), 7.87 (d, J = 4.0 Hz, 1H), 7.61 (d, J = 4.0 Hz, 2H), 7.21 – 7.10 (m, 3H), 7.08 – 6.91 (m, 3H), 6.26 (d, J = 7.6 Hz, 1H), 3.36 (d, J = 14.4 Hz, 1H), 2.88 (d, J = 14.4 Hz, 1H), 2.41 (s, 3H), 2.20 (s, 3H).

^{13}C NMR (100 MHz, $CDCl_3$): δ 204.7, 203.2, 190.6, 154.3, 135.3, 134.6, 132.2, 131.9, 130.1, 130.0, 129.7, 129.3 (q, J = 29.3 Hz), 128.3, 127.3 (q, J = 5.0 Hz), 127.1, 123.9 (q, J = 272.0 Hz), 78.6, 36.6, 28.1(q, J = 1.7 Hz), 27.6.

^{19}F NMR (376 MHz, $CDCl_3$): δ -55.95.

HRMS (m/z): $[M]^+$ calcd for $C_{22}H_{20}O_3F_3$, 389.1359; found 389.1348.

HPLC separation conditions: CHIRALPAK IA column (250 mm × 4.6 mm), hexane:*i*-PrOH = 80:20, 1.0 mL/min, T = 25 °C, λ = 214 nm, t_R (major) = 5.2 min, t_R (minor) = 6.1 min.

Chiral HPLC spectrum of racemic ***3y***

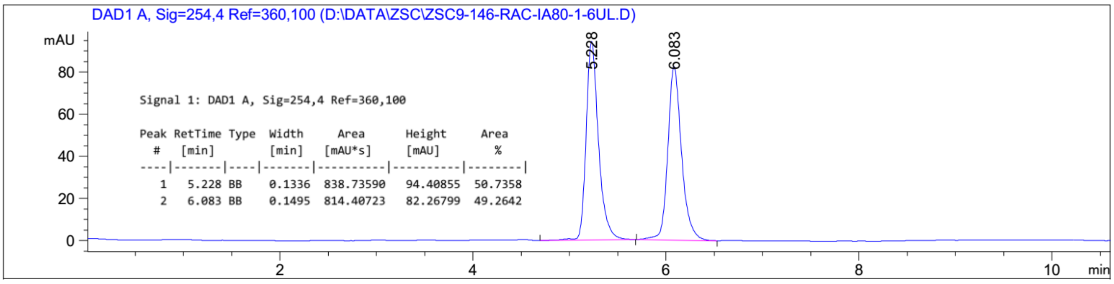

Chiral HPLC spectrum of ***3y***

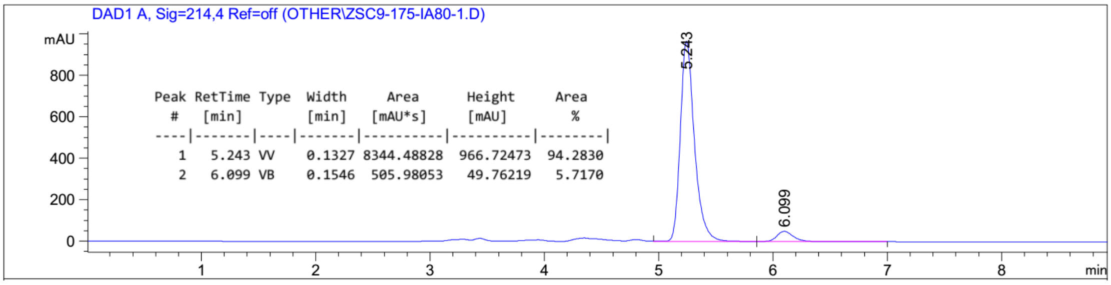

Supplementary Note 4

Versatile transformations of the compounds 3n and 3y

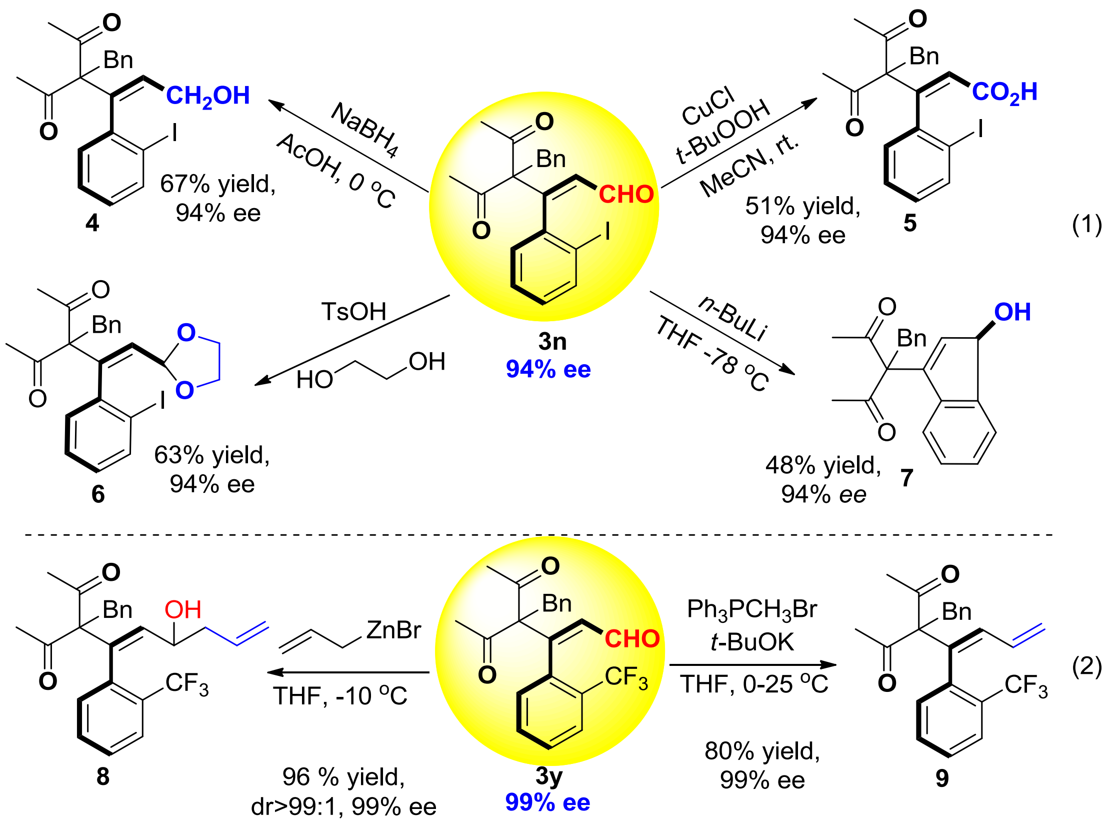

a) Reduction of **3n**

Reduction of **3n** was commenced according to the literature[4]. To a solution of **3n** (44.8 mg, 0.1 mmol) in 2 mL of acetic acid was added sodium borohydride (7.6 mg, 0.2 mmol) in portions at 0 °C. The reaction was continued for another hour (monitored by TLC). Water (5 mL) was added, and then the mixture was neutralized with a saturated solution of potassium bicarbonate (5 mL). The aqueous solution was extracted twice with 10 mL of ethyl acetate. The combined organic phase was washed with brine and dried over sodium sulfate. The solvent was removed under reduced pressure to give a crude residue. Further purification by flash column chromatography on silica gel and eluted with PE/EA (5/1) gave compound **4** as a white solid (29.6 mg, 67% yield).

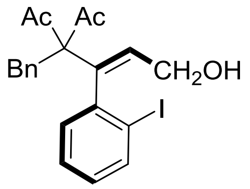

4

(*S*$_a$)-(*Z*)-3-benzyl-3-(3-hydroxy-1-(2-iodophenyl)prop-1-en-1-yl)pentane-2,4-dione

Pale solid, m.p.:131.4-132.4 °C, 29.6 mg, 67% yield

^{1}H NMR (400 MHz, CDCl$_3$): δ 7.97 (dd, J = 8.0, 0.8 Hz, 1H), 7.35 (td, J = 7.6, 1.0 Hz, 1H), 7.23 – 7.12 (m, 3H), 7.10 – 7.01 (m, 3H), 6.76 (dd, J = 7.6, 1.2 Hz, 1H), 5.97 (t, J = 6.4 Hz, 1H), 4.13 (dd, J = 13.6,

6.4 Hz, 1H), 3.86 (dd, J = 13.6, 6.4 Hz, 1H), 3.34 (d, J = 14.8 Hz, 1H), 2.91 (d, J = 14.8 Hz, 1H), 2.47 (s, 3H), 2.40 (s, 3H), 1.70 (s, 1H).

^{13}C NMR (100 MHz,): δ 206.8, 205.2, 140.7, 139.8, 139.3, 136.4, 135.6, 130.0, 129.7, 129.3, 128.6, 128.1, 126.7, 101.1, 77.8, 60.9, 36.2, 28.8, 27.5.

^{13}C NMR –DEPT 135 (100 MHz, $CDCl_3$): δ 139.8, 135.6, 130.0, 129.7, 129.3, 128.6, 128.1, 126.7, 60.9, 36.1, 28.8, 27.5.

HRMS (m/z): $[M]^+$ calcd for $C_{21}H_{22}O_3I$, 449.0608; found 449.0602.

HPLC separation conditions: CHIRALPAK IA column (250 mm × 4.6 mm), hexane:*i*-PrOH = 80:20, 1.0 mL/min, T = 25 °C, λ = 254 nm, t_R (major) = 7.2 min, t_R (minor) = 12.6 min.

*Chiral HPLC spectrum of racemic **4***

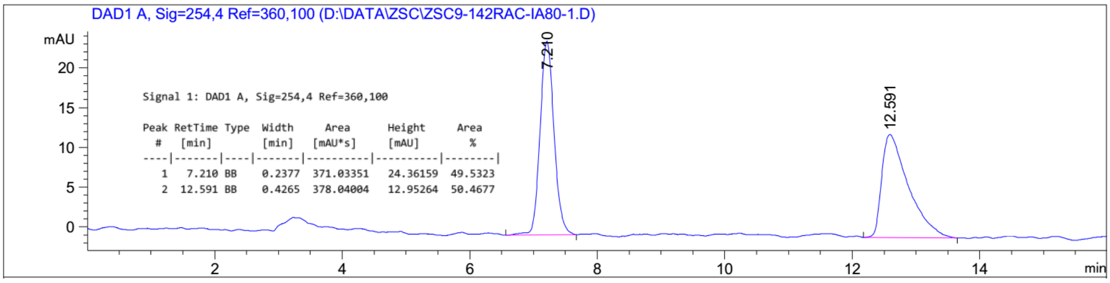

*Chiral HPLC spectrum of **4***

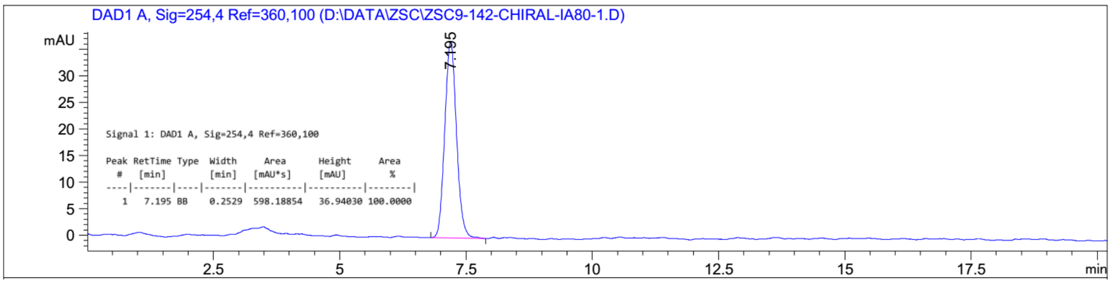

b) Oxidation of **3n**

Oxidation of **3n** was commenced according to the literature[5]. To a solution of **3n** (44.6 mg, 0.1 mmol) in 1 mL of MeCN was added CuCl (1.2 mg, 5 mol%) and *t*-BuOOH (70% in water, 0.15 mmol) successively. The reaction mixture was stirred for 5 hours (monitored by TLC). The solvent was removed under reduced pressure to give a crude residue. And further purification by flash column chromatography on silica gel and eluted with PE/EA (3/1) gave compound **5** as a white solid (23.5 mg, 51% yield).

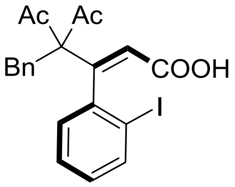

5

(S_a)-(Z)-4-acetyl-4-benzyl-3-(2-iodophenyl)-5-oxohex-2-enoic acid

White solid, m.p.: 118.6-120.1 °C, 23.5 mg, 51% yield.

^{1}H NMR (500 MHz, $CDCl_3$): δ 7.93 (d, J = 8.0 Hz, 1H), 7.36 (t, J = 7.5 Hz, 1H), 7.23 – 7.14 (m, 3H),

7.10 – 7.02 (m, 3H), 6.70 (d, J = 8.0 Hz, 1H), 6.13 (s, 1H), 3.39 (d, J = 15.0 Hz, 1H), 2.98 (d, J = 15.0 Hz, 1H), 2.44 (s, 3H), 2.39 (s, 3H).

^{13}C NMR (125 MHz, $CDCl_3$): δ 205.6, 203.2, 167.0, 154.9, 140.1, 139.5, 135.8, 130.0, 129.7, 128.3, 128.1, 127.3, 126.9, 125.1, 99.2, 78.9, 36.1, 29.1, 27.6.

HRMS (m/z): $[M]^-$ calcd for $C_{21}H_{18}O_4I$, 461.0255; found 461.0258.

HPLC separation conditions: CHIRALPAK IA column (250 mm × 4.6 mm), hexane:*i*-PrOH:TFA = 80:20:0.1, 1.0 mL/min, T = 25 °C, λ = 254 nm, t_R (major) = 5.6 min, t_R (minor) = 6.5 min.

Chiral HPLC spectrum of racemic **5**

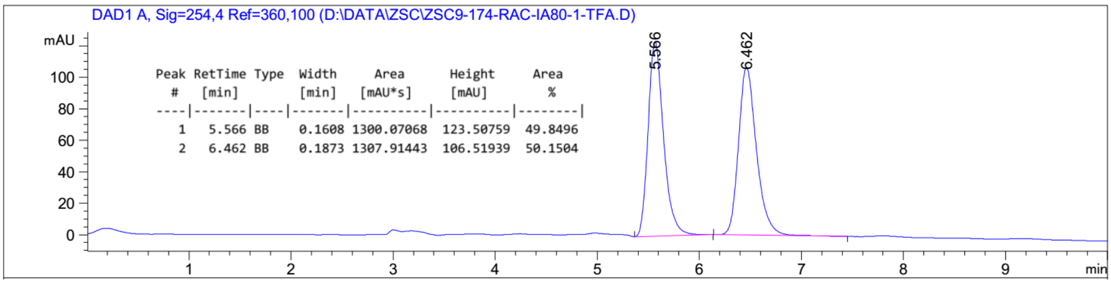

Chiral HPLC spectrum of **5**

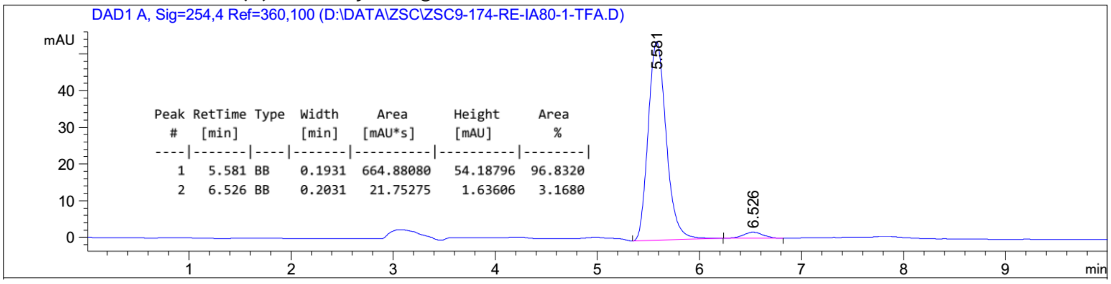

c) Compound **6** was prepared according to the literature[6]. Under Argon atomersphere, TrimethylOrthoformate (0.5 mmol, 5 equiv) was added a solution of **3n** (44.5 mg, 0.1 mmol) in 1 mL of ethylene glycol, and A little amount of dry CH_2Cl_2 was allowed to add into the mixture for a better solubility. Later, TsOH (1 mg, 1 mol%) was added to the mixture as a catalyst. The reaction mixture was stirred for 8 hours (monitored by TLC). Then the reaction was quenched with Ammonium Hydroxide (1 mL) and diluted with water (10 mL). The aqueous solution was extracted three times with 10 ml of ethyl acetate. The combined organic phase was washed with brine and dried over sodium sulfate. The solvent was removed under reduced pressure to give a crude residue. And further purification by flash column chromatography on neutral Al_2O_3 eluted with PE/EA (5/1) gave compound **6** as a white solid (30.9 mg, 63% yield).

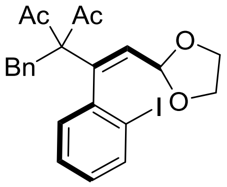

6

(*S*$_a$)-(*Z*)-3-(2-(1,3-dioxolan-2-yl)-1-(2-iodophenyl)vinyl)-3-benzylpentane-2,4-dione

White solid, m.p.: 116.3-128.0 °C, 30.9 mg, 63% yield.

^{1}H NMR (400 MHz, $CDCl_3$): δ 7.98 (dd, J = 8.0, 0.8 Hz, 1H), 7.37 (td, J = 7.6, 1.2 Hz, 1H), 7.22 – 7.12 (m, 3H), 7.11 – 7.02 (m, 3H), 6.80 (dd, J = 7.6, 1.6 Hz, 1H), 5.74 (d, J = 7.6 Hz, 1H), 4.95 (d, J = 7.6 Hz, 1H), 4.03 (q, J = 6.4 Hz, 1H), 3.99 – 3.85 (m, 2H), 3.75 (q, J = 6.4, Hz, 1H), 3.33 (d, J = 14.8 Hz, 1H), 2.98 (d, J = 14.8 Hz, 1H), 2.49 (s, 3H), 2.43 (s, 3H).

^{13}C NMR (100 MHz, $CDCl_3$): δ 206.7, 204.8, 143.6, 139.8, 139.7, 136.3, 132.9, 130.0, 129.8, 129.8, 128.3, 128.1, 126.7, 100.6, 100.3, 77.9, 65.5, 65.0, 35.8, 28.8, 27.4.

^{13}C NMR DEPT 135 (100 MHz, $CDCl_3$): δ 139.7, 132.9, 130.0, 128.1, 126.7, 100.3, 65.5, 65.0, 35.8, 28.8, 27.4.

HRMS (m/z): $[M]^+$ calcd for $C_{23}H_{24}O_4I$, 491.0714; found 491.0707.

HPLC separation conditions: CHIRALPAK IA column (250 mm × 4.6 mm), hexane:*i*-PrOH = 80:20, 1.0 mL/min, T = 25 °C, λ = 254 nm, t_R (major) = 5.6 min, t_R (minor) = 8.9 min.

*Chiral HPLC spectrum of racemic **6***

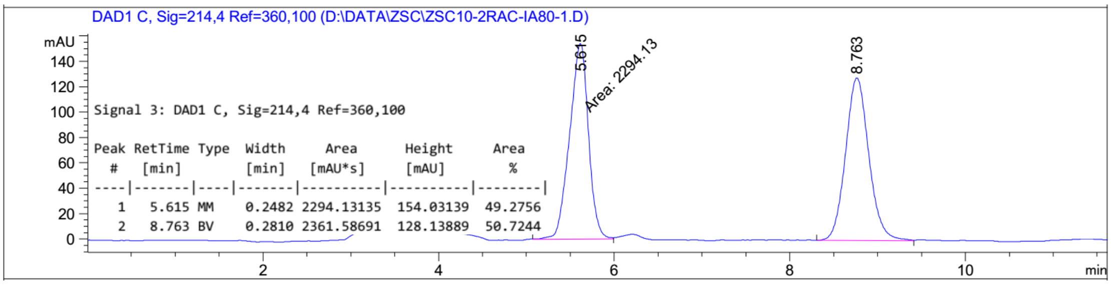

*Chiral HPLC spectrum of **6***

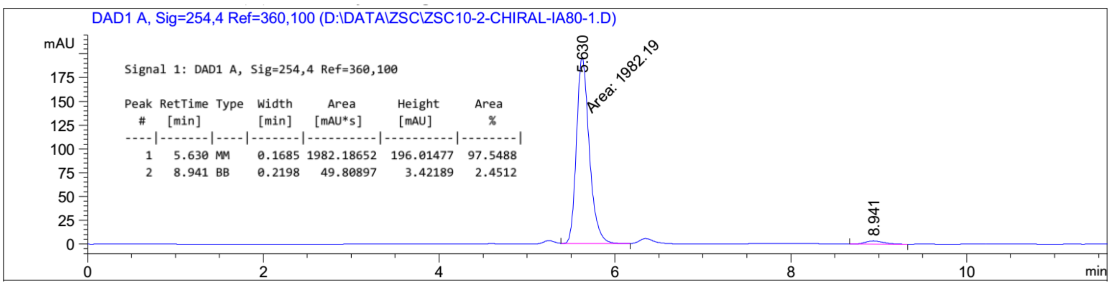

d) Compound **7** was prepared according to the literature[7]. ^{n}BuLi (2.4 M in Hexane, 62.5 μL, 0.15 mmol) was added by microsyringe to a solution of **3n** (44.6 mg, 0.10 mmol) in dry THF (2 mL) at -78 °C. The reaction mixture was stirred for 4h then quenched slowly with sat. NH_4Cl (0.5 mL) at the same temperature. After 10 minutes, the mixture was diluted with water (10 mL). The aqueous solution was extracted three times with 10 mL of ethyl acetate. The combined organic phase was washed with brine and dried over sodium sulfate. The solvent was removed under reduced pressure to give a crude residue. And further purification by flash column chromatography on silica gel and eluted with PE/EA (5/1) gave compound **7** as a white solid (15.3 mg, 48% yield).

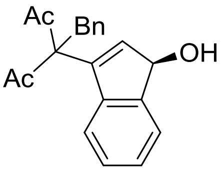

7

(*R*)-3-benzyl-3-(1-hydroxy-1H-inden-3-yl)pentane-2,4-dione

Colorless oil, 15.3 mg, 48% yield

^{1}H NMR (400 MHz, $CDCl_3$): δ 7.62 – 7.52 (m, 1H), 7.35 – 7.25 (m, 3H), 7.23 – 7.11 (m, 3H), 7.08 – 6.99 (m, 1H), 6.98 – 6.86 (m, 2H), 6.60 (d, J = 2.0 Hz, 1H), 5.13 (d, J = 7.2 Hz, 1H), 3.72 (d, J = 14.8 Hz, 1H), 3.65 (d, J = 14.8 Hz, 1H), 2.23 (s, 3H), 2.20 (s, 3H), 1.73 (d, J = 9.2 Hz, 1H).

^{13}C NMR (125 MHz, $CDCl_3$): δ 204.9, 204.6, 145.8, 140.8, 138.2, 136.1, 129.8, 128.5, 128.1, 127.0, 126.6, 123.9, 121.1, 76.0, 73.6, 38.1, 28.6, 28.4.

^{13}C NMR – DEPT 135 (125 MHz, $CDCl_3$): δ 138.2, 129.8, 128.5, 128.1, 127.0, 126.6, 123.9, 121.1, 76.0, 38.1, 28.6, 28.4.

HRMS (m/z): $[M]^+$ calcd for $C_{21}H_{21}O_3$, 321.1485; found 321.1476.

HPLC separation conditions: CHIRALPAK IC column (250 mm × 4.6 mm), hexane:*i*-PrOH = 80:20, 1.0 mL/min, T = 25 °C, λ = 230 nm, t_R (major) = 6.5 min, t_R (minor) =8.0 min.

*Chiral HPLC spectrum of racemic **7***

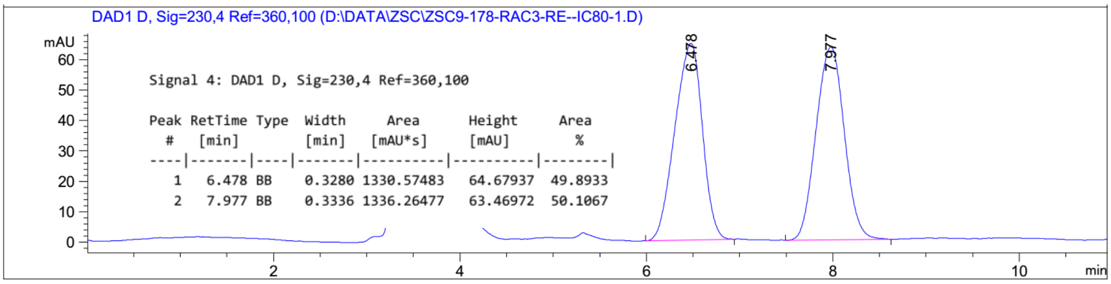

*Chiral HPLC spectrum of **7***

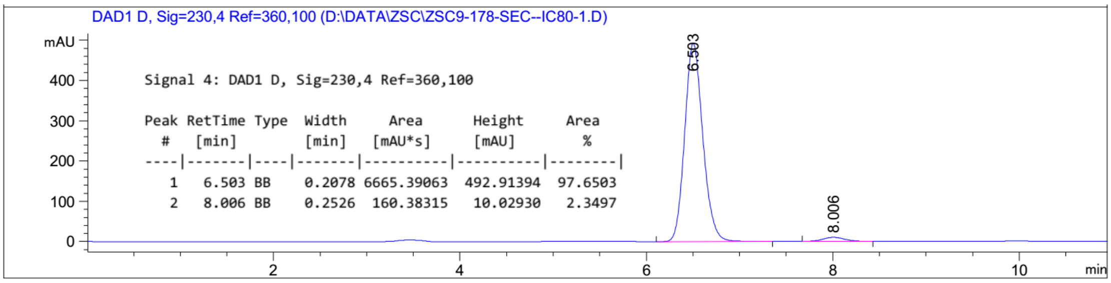

e) Compound **8** was prepared according to the literature [8]. At -10 °C, allylzinc bromide (0.5 M in THF, 0.15 mmol) was added to a solution of **3y** (38.6 mg, 0.10 mmol) in 2 mL THF. The reaction mixture was stirred for another 1h (monitored by TLC). The solvent was removed under reduced pressure to give a crude residue. And further purification by flash column chromatography on silica gel and eluted with PE/EA (3/1) gave compound **8** as thick oil (41.3 mg, 96% yield).

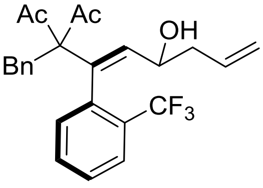

8

(*E*)-3-benzyl-3-(3-hydroxy-1-(2-(trifluoromethyl)phenyl)hexa-1,5-dien-1-yl)pentane-2,4-dione

White solid, m.p.: 98.8-101.1 °C, 41.3 mg, 96% yield

^{1}H NMR (500 MHz, $CDCl_3$): δ 7.83 (d, J = 7.5 Hz, 1H), 7.62 – 7.49 (m, 3H), 7.21 – 7.09 (m, 3H), 7.01

(d, J = 7.0 Hz, 2H), 6.83 (d, J = 7.5 Hz, 1H), 5.83 (d, J = 9.0 Hz, 1H), 5.66 – 5.50 (m, 1H), 5.07 (dd, J = 16.5, 10.0 Hz, 2H), 3.89 (q, J = 6.5 Hz, 1H), 3.23 (d, J = 14.5 Hz, 1H), 2.80 (d, J = 14.5 Hz, 1H), 2.45 (s, 3H), 2.28 (s, 3H), 2.24 (dt, J = 13.5, 6.5 Hz, 1H), 2.18 (dt, J = 13.5, 6.5 Hz, 1H), 1.86 (s, 1H).

^{13}C NMR (125 MHz, $CDCl_3$): δ 206.8, 205.1, 139.5, 136.2, 134.6, 133.8 (d, J = 1.9 Hz), 133.0, 131.9, 131.4, 130.0, 128.6, 128.0 (q, J = 28.9 Hz), 128.1, 127.3 (q, J = 5.2 Hz), 126.7, 124.3 (q, J = 273.9 Hz), 118.8, 77.5, 69.0, 40.7, 36.2, 27.8 (q, J = 1.8 Hz), 27.6.

^{19}F NMR (376 MHz, $CDCl_3$): δ -56.7.

HRMS (m/z): $[M]^+$ calcd for $C_{25}H_{29}O_3NF_3$ (M + NH^+) 448.2094; found 448.2089. Calcd for $C_{25}H_{24}O_2F_3$ (M – H_2O+ H^+)413.1723; found 413.1718.

HPLC separation conditions: CHIRALCEL OD-3 column (250 mm × 4.6 mm), hexane:*i*-PrOH = 90:10, 1.0 mL/min, T = 25 °C, λ = 214 nm, t_R (minor) = 8.5 min, t_R (major) = 9.5 min.

*Chiral HPLC spectrum of racemic **8***

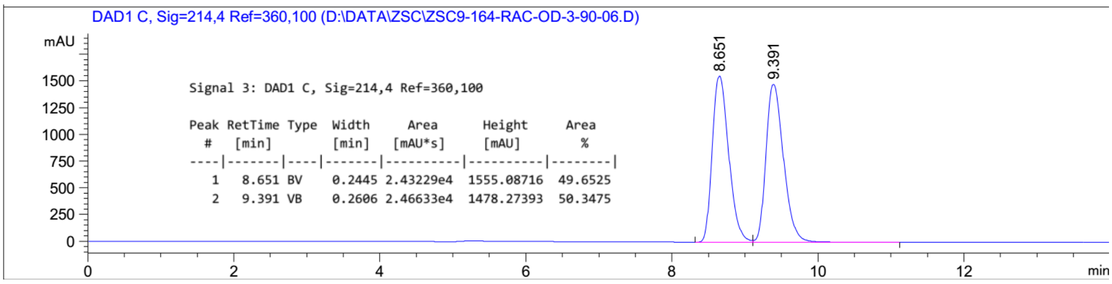

*Chiral HPLC spectrum of **8***

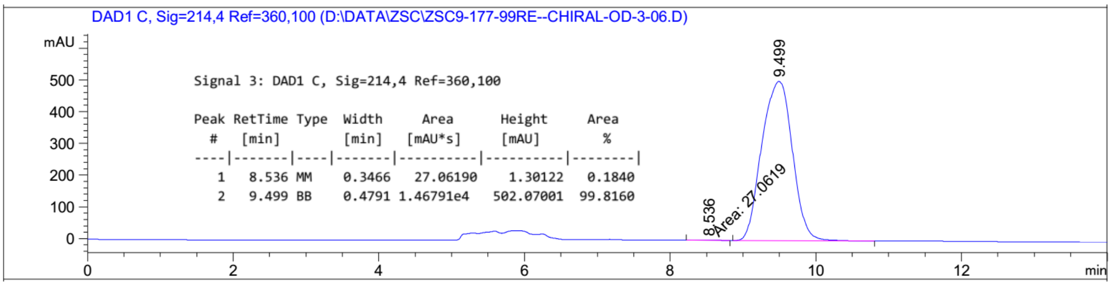

f) Compound **9** was prepared according to the literature[9]. KO^{t}Bu (14.0 mg, 0.12 mmol) was added to a solution of Methyltriphenylphosphonium bromide (39.3 mg, 0.11 mmol) in dry THF (1 mL) at 0 °C. The reaction mixture was stirred for 1h until **3y** (38.5 mg, 0.10 mmol) was added and then allowed to move to ambient temperature (25 °C) for another 5h (monitored by TLC). Then the reaction was quenched with sat. NH_4Cl (1 mL) and diluted with water (10 mL) was added. The aqueous solution was extracted three times with 10 ml of ethyl acetate. The combined organic phase was washed with brine and dried over sodium sulfate. The solvent was removed under reduced pressure to give a crude residue. And further purification by flash column chromatography on silica gel and eluted with PE/EA (8/1) gave compound **9** as a white solid (30.9 mg, 80% yield).

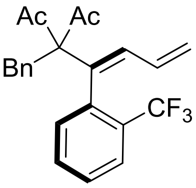

9

(*Sa*)-(*E*)-3-benzyl-3-(1-(2-(trifluoromethyl)phenyl)buta-1,3-dien-1-yl)pentane-2,4-dione

White solid, m.p.: 115.3-117.6 °C, 30.9 mg, 80% yield

^{1}H NMR (500 MHz, $CDCl_3$): δ 7.82 (d, J = 7.5 Hz, 1H), 7.56 (t, J = 7.5 Hz, 1H), 7.52 (t, J = 7.5 Hz, 1H), 7.23 – 7.10 (m, 3H), 7.04 (d, J = 7.1 Hz, 2H), 6.88 (d, *J* = 7.5 Hz, 1H), 6.34 (d, J = 10.5 Hz, 1H), 5.88 (dt, J = 17.0, 10.5 Hz, 1H), 5.36 (d, J = 17.0 Hz, 1H), 5.19 (d, J = 10.0 Hz, 1H), 3.31 (d, J = 14.5 Hz, 1H), 2.83 (d, J = 14.5 Hz, 1H), 2.45 (s, 3H), 2.27 (s, 3H).

^{13}C NMR (125 MHz, $CDCl_3$): δ 207.1, 205.2, 136.4, 134.6, 133.4, 132.0, 130.9, 130.0, 129.4, (q, J = 29.4 Hz), 128.5, 128.1, 127.12 (q, J = 5.2 Hz), 126.7, 124.1(q, J = 274.0 Hz), 121.2, 77.7, 36.5, 27.9 (q, J = 1.7 Hz), 27.5.

^{19}F NMR (376 MHz, $CDCl_3$): δ -57.2.

HRMS (m/z): $[M]^+$ calcd for $C_{23}H_{22}O_2F_3$, 387.1566; found 387.1562.

HPLC separation conditions: CHIRALPAK IA column (250 mm × 4.6 mm), hexane:*i*-PrOH = 80:20, 1.0 mL/min, T = 25 °C, λ = 254 nm, t_R (major) = 4.6 min, t_R (minor) = 7.0 min.

Chiral HPLC spectrum of racemic ***9***

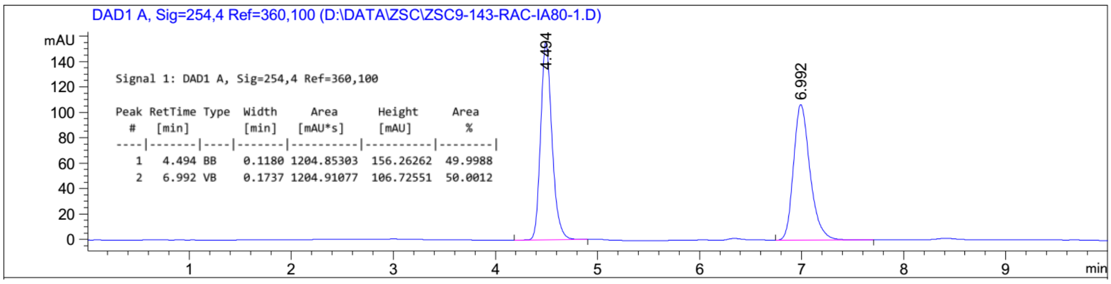

Chiral HPLC spectrum of ***9***

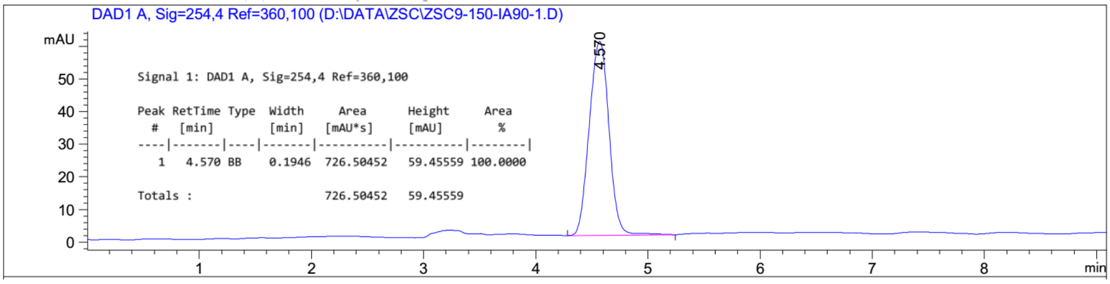

Supplementary References

1. Stiller, J.; Marqués-López, E.; Herrera, R. P.; Fröhlich, R.; Strohmann, C. & Christmann, M. Enantioselective α- and γ-alkylation of α,β-unsaturated aldehydes using dienamine activation. *Org. Lett.* **13**, 70-73 (2011).

2. Kenny, M.; Christensen, J.; Coles, S. J. & Franckevicius, V. Regioswitchable palladium-catalyzed decarboxylative coupling of 1,3-dicarbonyl compounds *Org. Lett.* **17**, 3926-3929 (2015).

3. Kaiser, J.; van Esseveldt, B. C. J.; Segers, M. J. A.; van Delft, F. L.; Smits, J. M. M.; Butterworth, S. & Rutjes, F. P. J. T. Synthesis and aromatisation of cyclic enediyne-containing amino acids *Org. Biomol. Chem.* **7**, 695-705 (2009).

4. Liu, J. *et al*. A convenient synthesis of (*R*)-salmeterol via Rh-catalyzed asymmetric transfer hydrogenation. *Tetrahedron: Asymmetry* **19**, 1824-1828 (2008).

5. Mannam, S. & Sekar, G. CuCl catalyzed oxidation of aldehydes to carboxylic acids with aqueous *tert*-butyl hydroperoxide under mild conditions. *Tetrahedron Lett.* **49**, 1083-1086 (2008).

6. Xie, Y. & Floreancig, P. E. Stereoselective heterocycle synthesis through a reversible allylic alcohol transposition and nucleophilic addition sequence. *Chem. Sci.* **2**, 2423-2427 (2011).

7. Garcia-Yebra, C., Janssen, J. P., Rominger, F. & Helmchen, G. Asymmetric iridium(I)-catalyzed allylic alkylation of monosubstituted allylic substrates with phosphinooxazolines as Ligands. Isolation, characterization, and reactivity of chiral (allyl)iridium(III) complexes. *Organometallics*, **23**, 5459-5470 (2004).

8. Kahnberg, P., Lee, C. W., Grubbs, R. H. & Sterner, O. Alternative routes to pterulone. *Tetrahedron* **58**, 5203-5208 (2002).

9. Sada, M., Ueno, S., Asano, K., Nomura, K. & Matsubara, S. Stereoselective preparation of 3-alkanoylprop-2-en-1-ol derivatives. *Synlett.* 724-726 (2009).
